# Quantifying the ability of the CF_2_H group as a hydrogen bond donor

**DOI:** 10.3762/bjoc.21.11

**Published:** 2025-01-20

**Authors:** Matthew E Paolella, Daniel S Honeycutt, Bradley M Lipka, Jacob M Goldberg, Fang Wang

**Affiliations:** 1 Department of Chemistry, University of Rhode Island, 140 Flagg Rd, Kingston, RI 02881, USA; 2 Department of Chemistry, Colgate University, 13 Oak Drive, Hamilton, NY 13346, USA

**Keywords:** bioisostere, difluoromethyl (CF_2_H), fluorine, hydrogen bond donors, hydrogen bond strength

## Abstract

The CF_2_H group can act as a hydrogen bond donor, serving as a potential surrogate for OH or SH groups but with a weaker hydrogen bond donation ability. Here, we describe a series of CF_2_H group-containing moieties that facilitate hydrogen bond interactions. We survey hydrogen bond donation ability using several established methods, including ^1^H NMR-based hydrogen bond acidity determination, UV–vis spectroscopy titration with Reichardt's dye, and ^1^H NMR titration using tri-*n*-butylphosphine oxide as a hydrogen bond acceptor. Our experiments reveal that the direct attachment of the CF_2_H group to cationic aromatic systems significantly enhances its hydrogen bond donation ability, a result consistent with theoretical calculations. We anticipate that this chemistry will be valuable for designing functional molecules for chemical biology and medicinal chemistry applications.

## Introduction

**Figure 1:**
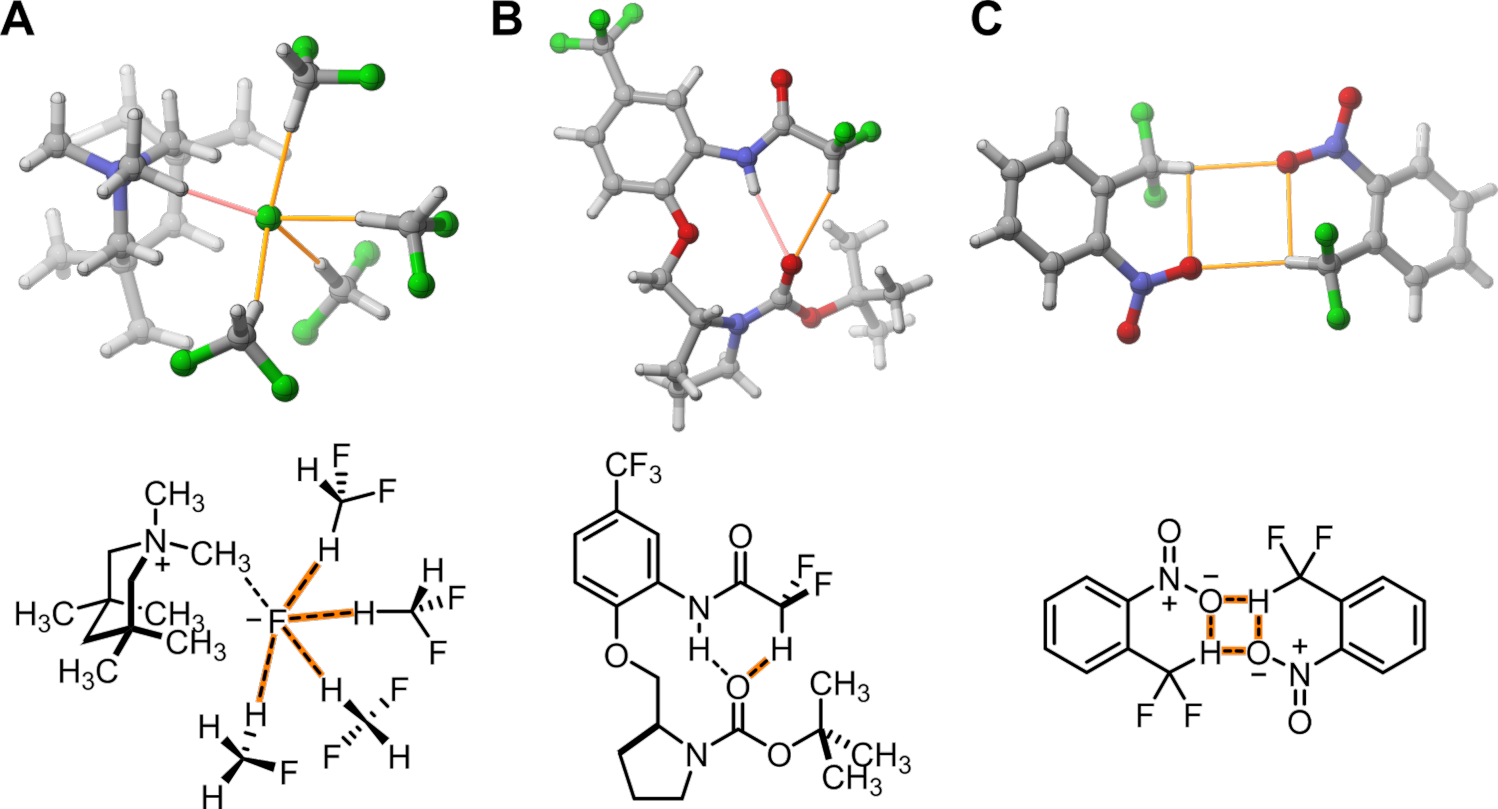
Examples of solid state structures exhibiting CF_2_H group-mediated hydrogen bond interactions [16,18,21]. Hydrogen bonding interactions involving the CF_2_H group are highlighted in orange.

Hydrogen bonding interactions are ubiquitous non-covalent forces in chemistry and biology [1–4]. In canonical hydrogen bond (HB) donor–acceptor pairs, the donor typically comprises an electronegative heteroatom, such as oxygen, nitrogen, or sulfur, and a positively charged hydrogen atom, which interacts with a lone pair on the acceptor. Apart from these common heteroatom-containing hydrogen bond donors, certain carbon–hydrogen moieties can also act in this way, although in a substantially weaker capacity [5–14]. Of particular interest is the difluoromethyl group, CF_2_H, which exhibits hydrogen bond donating character due to the highly polarized F_2_C–H bond (Figure 1) [14–24]. This functional group is often used to mimic hydroxy or thiol groups but exhibits slower acid dissociation [25] and different lipophilicity [19–20,26–28]. For these reasons, it is an attractive synthetic target [29–43] and an important bioisostere in drug design and biochemical studies [30,44–46]. Despite the value of these applications, few experimental studies have been conducted to quantify the thermodynamics of CF_2_H group-mediated hydrogen bond interactions [19–20]. Here, we present a series of CF_2_H-containing constructs and a detailed assessment of the corresponding hydrogen bond donation energetics. We expect this information to be useful for the rational application of the CF_2_H group in drug development and molecular design.

**Figure 2:**
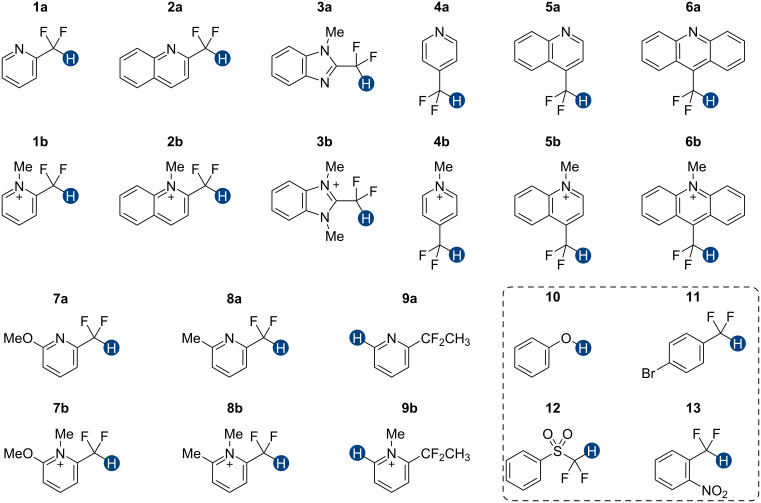
Hydrogen bond donors investigated in this study. For all cationic species, the counteranion is BF_4_^−^. The reference HB donors are in the dashed line box.

Previous quantum mechanical calculations revealed that the CF_2_H···O binding energy (Δ*E*) ranges from 1.0 kcal/mol to 5.5 kcal/mol [14–15,18,21]. In addition, as measured by hydrogen bond acidity [47–48] which is derived from the ^1^H NMR chemical shift difference of a given proton in DMSO-*d*_6_ and CDCl_3_, the CF_2_H group is generally a stronger donor than the methyl group but substantially weaker than the OH or amide NH groups [19–20]. These results collectively indicate that, although the CF_2_H group mimics hydroxy or thiol groups, it is a generally less effective hydrogen bond donor. Given that the HB donation ability of a particular functional group usually increases with increasing Brønsted acidity [49] we chose to incorporate the CF_2_H group into the backbone of *N*-methylpyridinium cations and related analogs (Figure 2). We anticipated that such cationic constructs would enhance the Brønsted acidity of the CF_2_–H bond by stabilizing the conjugate base of the CF_2_H group, in turn, increasing the hydrogen bond donation ability. Additionally, to minimize the effects of counterions, such as the bromide and fluoride anions [50], on HB interactions, all ionic compounds were synthesized with tetrafluoroborate, a classical weakly coordinating anion.

## Results and Discussion

We first assessed the hydrogen bond acidity, *A*, of these CF_2_H-containing compounds using an established method [19–20,47–48]. This convenient approach relies on comparing the ^1^H NMR chemical shift of a hydrogen bond donor in DMSO-*d*_6_ to that of it in CDCl_3_. The HB donor presumably interacts strongly with hydrogen-accepting DMSO [51], but barely with CDCl_3_, which has a weak hydrogen bond acceptance ability [51], so the magnitude of the solvent-induced chemical shift difference, Δδ_DMSO–CDCl3_ = δ_DMSO_ − δ_CDCl3_ should positively correlate with the HB donation ability. Accordingly, the *A* value can be defined as *A* = 0.0065 + 0.133Δδ_DMSO–CDCl3_. We determined the Δδ_DMSO–CDCl3_ values for a series of hydrogen bond donors. Our experiment with neutral HB donors reproduced literature results (Table 1, compounds **10**, **11**, and **12**) [20,22,47] and revealed an expected trend in HB donation ability; for example, compound **1a** is a weaker HB donor than **3a**. However, due to limited solubility, the ^1^H NMR spectroscopic studies of organic salts in CDCl_3_, including **1b** and **3b**, did not produce observable signals. To solubilize the salts better, we substituted deuterated nitromethane (CD_3_NO_2_) for CDCl_3_. Because of the nearly identical hydrogen donation and acceptance abilities of nitromethane (α = 0.22 and β = 0.06, respectively) and chloroform (α = 0.20 and β = 0.10) [51], Δδ_DMSO–CD3NO2_ and Δδ_DMSO–CDCl3_ should follow a similar trend. Our ^1^H NMR experiments showed a strong linear correlation between Δδ_DMSO–CD3NO2_ and Δδ_DMSO–CDCl3_ for neutral HB donors (R^2^ = 0.985, Figure S15 in Supporting Information File 1), confirming that CD_3_NO_2_ can be used to determine HB acidity (Table 1 and Figures S1–S13 in Supporting Information File 1). Based on the Δδ_DMSO–CD3NO2_ values, we can rank the relative HB donation ability of the CF_2_H-containing salts as **3b** > **1b** > **4b**, a result consistent with the expected Brønsted acidity. Even so, the Δδ_DMSO–CD3NO2_ values of *N*-methylated CF_2_H-containing organic salts are generally smaller than those of the corresponding neutral precursors. This observation contradicts our initial prediction that introducing a quaternary nitrogen would enhance the HB donation ability of the CF_2_H group. It is also at odds with the experimental and theoretical results described below. We tentatively attributed the discrepancies to the involvement of other possible solute–solvent interactions, such as solute dipolarity, polarizability, and dispersion [47]. Ostensibly, these interactions can vary significantly as the charge of the solute changes, complicating the Δδ-based direct assessment of HB acidity.

**Table 1:** Summary of Δδ_DMSO–CDCl3_, Δδ_DMSO–CD3NO2_, and *A* values of select HB donors.^a^

		Δδ_DMSO–CDCl3_ (ppm)	*A*	Δδ_DMSO–MeNO2_ (ppm)

**1a**	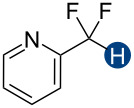	0.31	0.047	0.26
**1b**	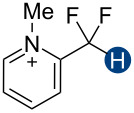	–	–	0.32
**2a**	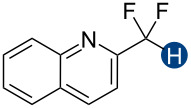	0.31	0.048	–
**3a**	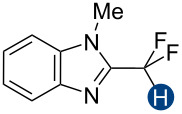	0.47	0.069	0.39
**3b**	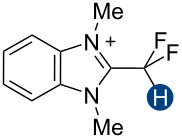	–	–	0.37
**4a**	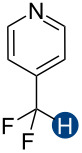	0.30	0.046	–
**4b**	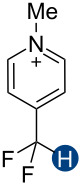	–	–	0.27
**5a**	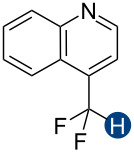	0.53	0.077	–
**6a**	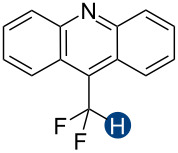	0.54	0.078	0.35
**10**	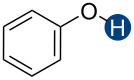	4.66 (4.69)^b^	0.63 (0.63)^b^	3.90
**11**	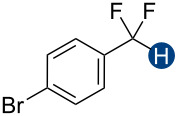	0.44 (0.43)^b^	0.065 (0.064)^b^	0.29
**12**	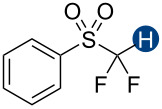	1.13	0.16 (0.16)^b^	0.84
**13**	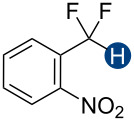	0.08	0.017	0.09

^a^For all cationic species, the counteranion is BF_4_^−^. ^b^Literature values are shown in parentheses.

**Figure 3:**
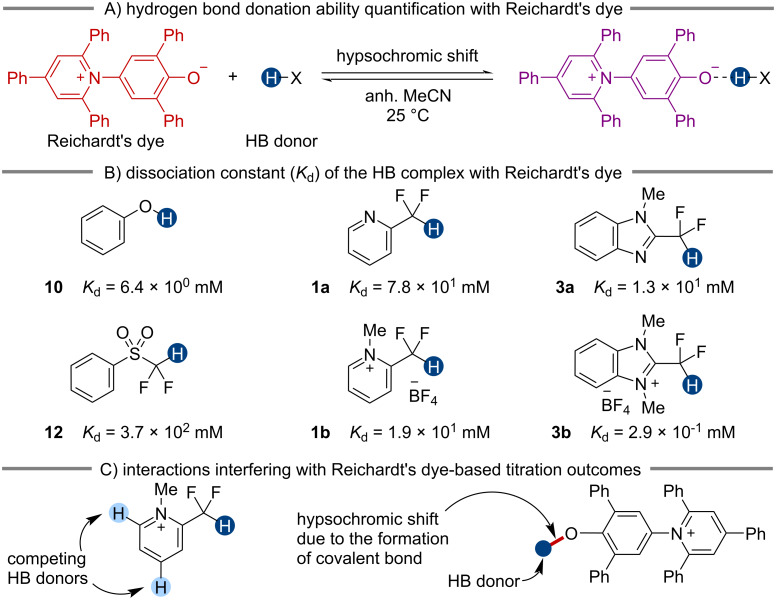
Hydrogen bond donation ability determined by UV–vis spectroscopy titration. A) Formation of HB complexes of Reichardt's dye and HB donors. B) *K*_d_ values of Reichardt's dye–hydrogen bond donor complexes. For all cationic species, the counteranion is BF_4_^−^. C) Possible interactions that interfere with the titration outcomes.

To quantify the HB donation ability of both neutral and cationic species on a single scale, we chose an alternative strategy based on an established UV–vis spectroscopy titration method [52] with Reichardt's dye [53–55] as an indicator. These experiments measure the blue shift of Reichardt's dye upon complexation with an HB donor (Figure 3A, and Figures S13–S18 in Supporting Information File 1), from which the dissociation constant (*K*_d_) of the HB complex can be determined. A smaller *K*_d_ value corresponds to a more stable complex, indicating a stronger HB donor. We employed this protocol to investigate a series of HB donors in anhydrous acetonitrile (Figure 3B). Acetonitrile is weakly HB accepting (α = 0.19) [51] and was thus chosen to attenuate the competition between the solvent and the dye with the HB donor. As shown in Figure 3B, in our hands, the *K*_d_ of the phenol–Reichardt's dye HB adduct determined is consistent with the reported value [52]. Some of our other results, however, were puzzling. For example, according to our titration data, **1a** is a better HB donor than **12**. This observation is inconsistent with the corresponding *A* values (Table 1), which typically provide reliable measurements of the HB donation ability of neutral compounds. We attribute the inconsistency to several factors. First, because the binding affinity is determined solely by the absorbance change of Reichardt's dye, the apparent *K*_d_ value only represents the overall ability of a compound to serve as an HB donor. For compounds bearing multiple HB donating sites, such as **1b**, the HB interactions involving individual functional groups cannot be quantified separately, leading to potentially ambiguous results (Figure 3C). Reports in the literature show that the UV–vis absorption of the Lewis basic Reichardt’s dye disappears in the presence of some cationic HB donors [52]. We found similar results with **3b** and likewise ascribe the unexpectedly small *K*_d_ to such limitations of this assay (Figure 3C and Figure S19 in Supporting Information File 1). Overall, despite the convenience, this UV–vis titration method may not be broadly applicable for quantifying the HB donation ability of some CF_2_H group-containing substrates.

**Figure 4:**
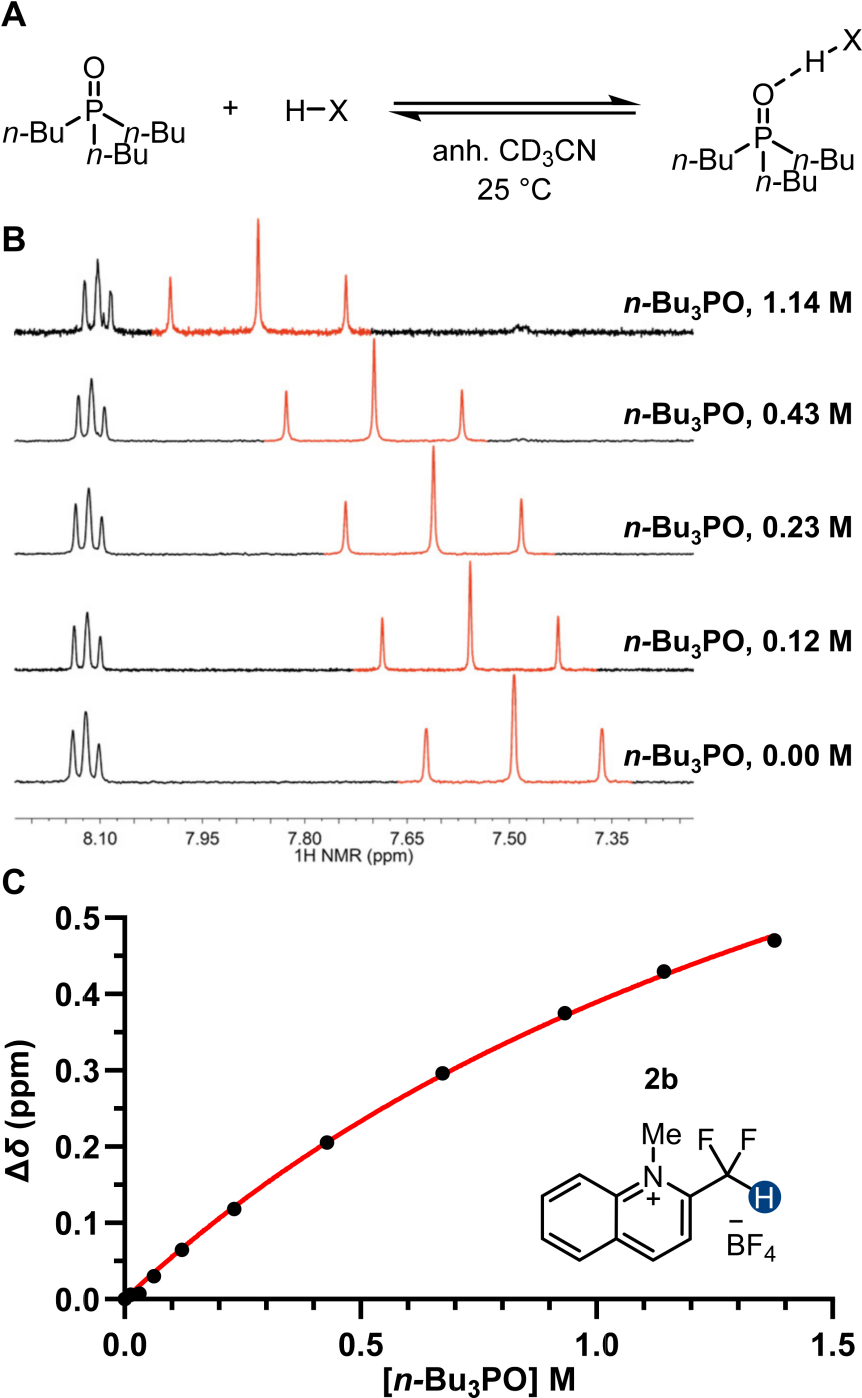
A) HB complex formation between a donor and tri-*n*-butylphosphine oxide. B) ^1^H NMR spectra of **2b** (5.0 mM) in the presence of different concentrations of tri-*n*-butylphosphine oxide in anhydrous CD_3_CN at 298 K. The ^1^H NMR signals of the CF_2_H group are indicated in red. C) Determination of the dissociation constant (*K*_d_) of the *n-*Bu_3_PO···**2b** HB complex by fitting the data to a single-site binding model.

To quantify better the thermodynamics of CF_2_H group-mediated hydrogen bond interactions, we investigated the HB donation ability of the CF_2_H group by ^1^H NMR titration with tri-*n*-butylphosphine oxide (*n-*Bu_3_PO) as a reference HB acceptor (Figure 4A and Figures S20–S40 in Supporting Information File 1). Unlike a previous method that relied on ^31^P NMR spectroscopy [52], our titration monitors the HB complex formation by ^1^H NMR chemical shift change, thereby allowing the interactions of individual HB donating moieties with *n-*Bu_3_PO to be probed (Figure 4B and C). Moreover, we used anhydrous deuterated acetonitrile (CD_3_CN) as the solvent, in which both neutral and ionic compounds exhibited appreciable solubility. In this way, we were able to determine the HB donation ability of CF_2_H-containing compounds on a single scale.

**Figure 5:**
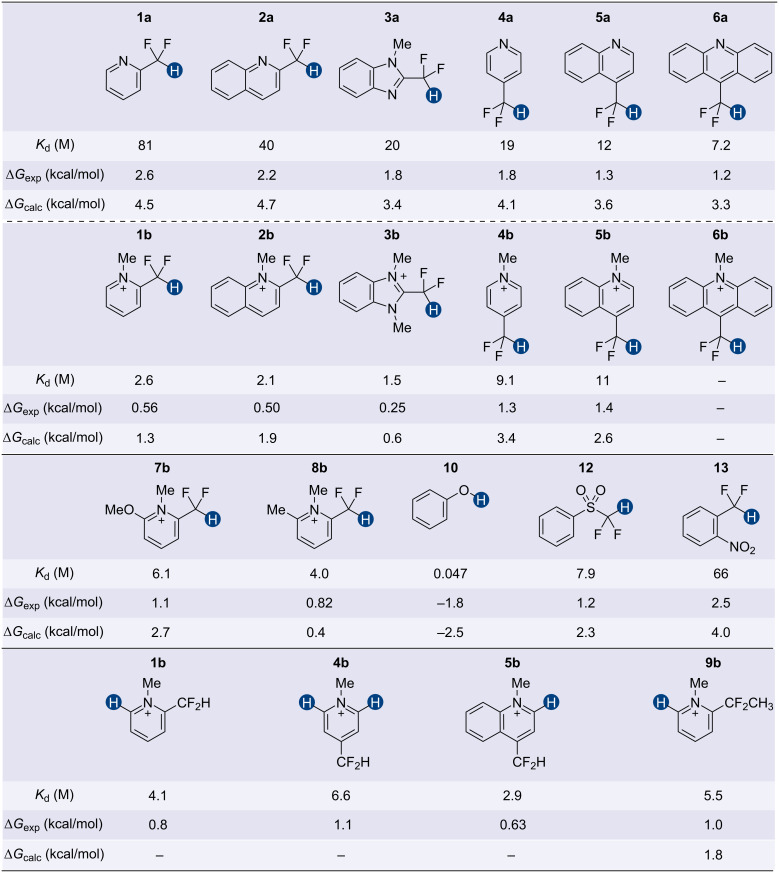
Hydrogen bond donation ability of various donors as quantified by the dissociation constant (*K*_d_) of the HB complex with tri-*n*-butylphosphine oxide at 298 K in anhydrous CD_3_CN. The *K*_d_ for **6b** was not determined due to the formation of non-HB-mediated adducts (Figure S34 in Supporting Information File 1). The corresponding experimental Gibbs free energy of binding (Δ*G*_exp_) is calculated based on the *K*_d_ values. The predicted Gibbs free energy of binding (Δ*G*_calc_) was calculated at the PCM(MeCN)-M06-2X/6-31+G(d,p) level of theory. The counteranion for all cationic species is BF_4_^−^.

As shown in Figure 5, we determined dissociation constants (*K*_d_) of *n-*Bu_3_PO···HB donor complexes, revealing several general trends. First, we found that CF_2_H groups attached to an extended aromatic system are stronger HB donors (**2a** > **1a**, **2b > 1b**, and **6a** > **5a** > **4a**), likely due to the increased Brønsted acidity of the CF_2_–H bond. Similarly, cationic donors generally exhibited substantially higher HB donation ability than the neutral precursors, as indicated by ten to thirty-fold decreases in *K*_d_ values (Figure 5, **1**–**4**). These two enhancing effects are, however, not strictly additive. For example, comparing **1a** and **2a**, a two-fold decrease in the *K*_d_ value was observed. Between **1a** and **1b**, there is a 31-fold change; between **2a** and **2b**, the difference is 17-fold. In contrast, the HB interactions involving **2b** are marginally stronger than those involving **1b**. Similar trends were also seen with **4** and **5**. These observations suggest that the delocalization of the positive charge in an extended π system reduces its ability to facilitate CF_2_H-mediated HB interactions. Analogously, cationic CF_2_H-containing molecules bearing electron-donating methoxy groups are also weak HB donors (**7b** vs **1b**). Furthermore, the cationic activation of HB donors is negligible when the quaternary nitrogen is *para* rather than *ortho* to the CF_2_H group (**4** vs **5**). These findings indicate that the presence of either a quaternary nitrogen or an extended aromatic system can enhance the HB donation ability of the CF_2_H group, but the effects are more pronounced when they are close to the CF_2_H group.

We also compared the HB donation ability of different classes of compounds. In neutral CF_2_H-containing HB donors, the phenylsulfonyl group (**12**) is generally a stronger activator than heteroaryl (**1a**–**6a**) or electron-deficient aryl groups (**13**). In contrast, pyridinium and benzimidazolium (**1b**–**5b**) systems show substantially higher capacities to enhance the HB donation ability of the CF_2_H group, underscoring the distinct nature of these constructs. Although many of the CF_2_H HB donors studied here can promote relatively strong hydrogen bonding interactions with *n-*Bu_3_PO, even the strongest CF_2_H HB donor (**3b**) is still 30 times weaker than phenol (**10**), corresponding to about a 2 kcal/mol reduction in binding energy at 25 °C. These results reveal the fundamental differences between the C–H bond and the O–H bond as HB donors and provide important quantitative information for applying the CF_2_H group as an OH group mimic.

We next attempted to establish correlations of experimentally determined HB donation ability, in terms of *K*_d_ or Δ*G*_exp_, with other easily accessible parameters. We first calculated the Gibbs free energy of formation (Δ*G*_calc_) of the HB complexes of HB donors with trimethylphosphine oxide (Me_3_PO), which models *n-*Bu_3_PO as a hydrogen bond acceptor, and compared these values with experimental data. We realized that such an analysis oversimplified the system by neglecting to account for potential contributions from different conformers possibly involved in HB interactions. To rectify this problem, we searched for two possible structures for each Me_3_PO–HB donor pair, where the HB donor adopts a different conformation in each HB complex. Values for Δ*G*_calc_ were then calculated as the weighted average of the free energy of each HB complex as


**Figure 6:**
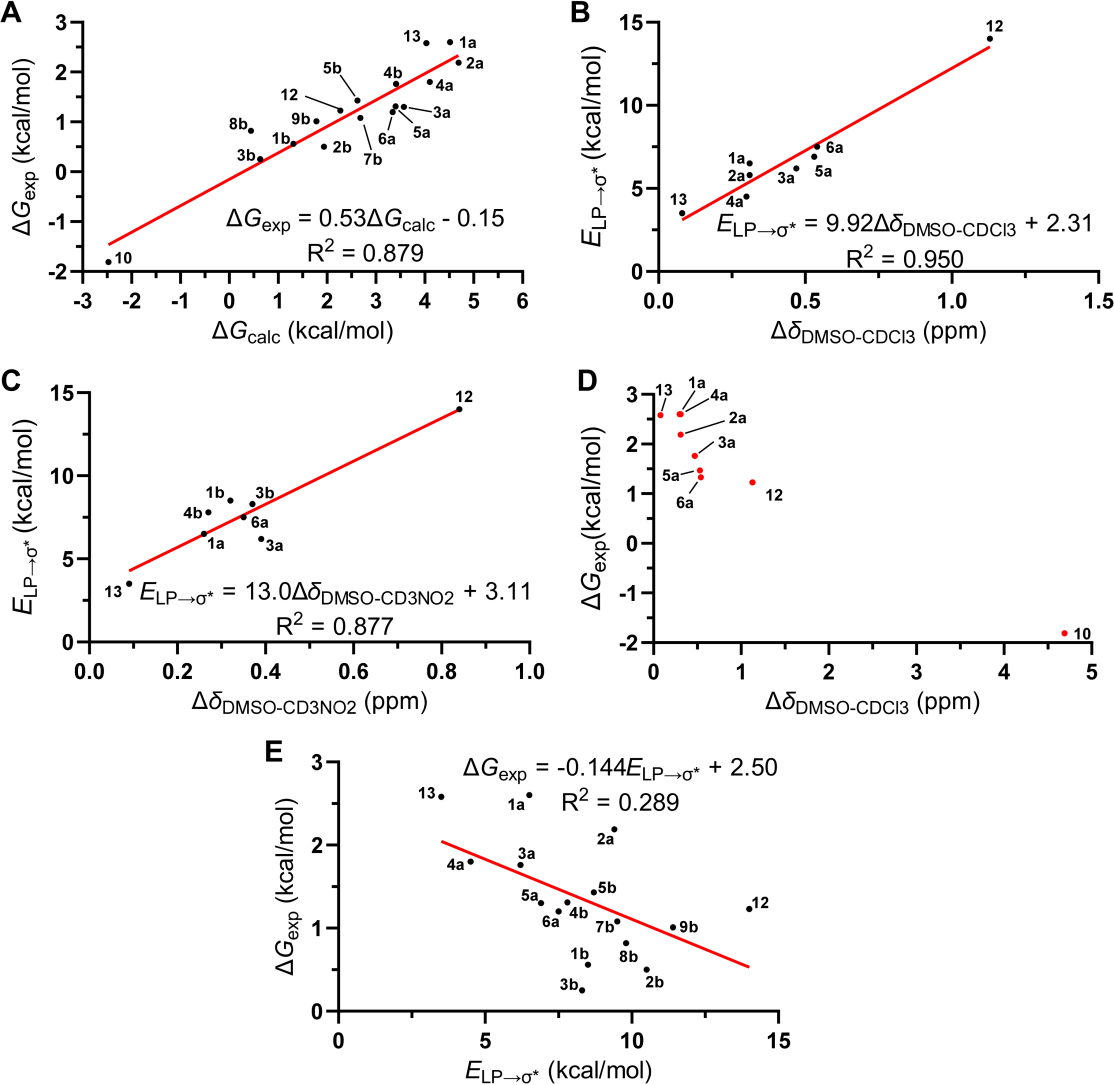
A) Linear correlation between Δ*G*_exp_ and Δ*G*_calc_. Δ*G*_exp_ and Δ*G*_calc_ values are shown in Figure 5. B) Linear correlation between *E*_LP→σ*_ and Δδ_DMSO−CDCl3_. *E*_LP→σ*_ values can be found in Supporting Information File 1. Δδ_DMSO−CDCl3_ values are listed in Table 1. C) Linear correlation between *E*_LP→σ*_ and Δδ_DMSO−CD3NO2_. Δδ_DMSO−CDCl3_ values are listed in Table 1. D) Inverse relationship between Δ*G*_exp_ and Δδ_DMSO−CDCl3_. E) Weak correlation between Δ*G*_exp_ and *E*_LP→σ*_.

[1] $$\Delta G_{\text{calc}}=\;-RT\;ln\;\left[\left(P_{{\text{Me}}_{3}\text{PO···HB,a}}+P_{{\text{Me}}_{3}\text{PO···HB,b}}\right)/\left(P_{{\text{Me}}_{3}\text{PO | HB,a}}+P_{{\text{Me}}_{3}\text{PO | HB,b}}\right)\right]$$

in which *P*_Me3PO···HB,a_ and *P*_Me3PO···HB,b_ are the percent populations of the HB complex of Me_3_PO with the donor conformer a and b, respectively; *P*_Me3PO | HB,a_ and *P*_Me3PO | HB,b_ are the percent populations of Me_3_PO and the corresponding HB donor conformer as two non-interacting molecules (see Supporting Information File 1 for details). We found a strong linear correlation between Δ*G*_exp_ and Δ*G*_calc_ obtained at the PCM(MeCN)-M06-2X/6-31+G(d,p) level of theory (Figure 5 and Figure 6A). These results demonstrate the reliability of this relatively efficient computational approach for predicting the HB donation ability of CF_2_H-containing molecules.

We further conducted natural bond orbital (NBO) [56] second-order perturbation analysis [57] to estimate the interaction energies (*E*_LP→σ_*) of the oxygen lone pairs (LPs) of Me_3_PO with the H–CF_2_Ar antibonding orbital (σ*). Such hyperconjugative interactions indicate the magnitudes of the charge transfer from the LPs to the σ* orbitals and are considered the major contributors to hydrogen bonding [57]. Using this analysis, strong linear correlations were found between *E*_LP→σ*_ and Δδ_DMSO–CDCl3_ or Δδ_DMSO–CD3NO2_ values (Figure 6B,C and Supporting Information File 1, Figures S44 and S45), implicating specific orbital interactions between the HB donating and accepting motifs that are responsible for chemical shift differences. In contrast, a relatively weak inverse association was observed between Δ*G*_exp_ and Δδ_DMSO–CDCl3_ values for neutral hydrogen bond donors (Figure 6D). This result suggests that the CF_2_H···O interactions are likely to be a predominant contributor to the binding between HB donating and accepting molecules but other weak intermolecular forces, collectively, may also play a role. This proposal is further supported by the weaker linear relationship between Δ*G*_exp_ and *E*_LP→σ*_ (Figure 6E and Figure S46 in Supporting Information File 1).

Collectively, these results indicate that the Δδ_DMSO–CDCl3_ or Δδ_DMSO–CD3NO2_ measurement can discriminate CF_2_H HB interactions from other non-covalent forces. In this way, it is possible to parse the HB donating contribution of the CF_2_H functional group within a given class of compounds, such as neutral or cationic donors, as shown here. One limitation of this approach is that it does not directly provide information about binding affinity or energy, particularly between HB donors and acceptors as molecular entities rather than as a collection of separate functional groups. In contrast, NMR titration experiments quantify the binding affinities and energies between CF_2_H-containing molecules and *n-*Bu_3_PO as the concatenation of many non-covalent forces. For example, our experiments showed that some C_Ar_–H bonds, such as those of the pyridinium ring, can serve as good HB donors (Figure 5). Because C_Ar_–H bonds and the CF_2_H group have comparable HB donation abilities, care needs to be taken when assigning specific contributions of each to the observed binding affinities. Even so, ^1^H NMR titration experiments with phosphine oxides still allow us to partially resolve these two forces by monitoring the proton of the CF_2_H group. Such issues are particularly salient when quantification methods that rely only on acceptor readouts, such as the Reichardt’s dye-based UV–vis titration, rendering results that are difficult to interpret (Figure 3). Overall, to survey the HB donating ability of the CF_2_H-containing molecules systematically, a combination of NMR titration and Δδ_DMSO–CDCl3_ or Δδ_DMSO–CD3NO2_ measurements is desirable.

## Conclusion

In conclusion, we have identified a series of CF_2_H-containing compounds that can serve as HB donors. We employed several experimental methods to quantify HB donation ability, including (i) ^1^H NMR chemical shift-based hydrogen bond acidity, *A* value, measurements, (ii) UV–vis spectroscopic titrations with Reichardt’s dye, and (iii) ^1^H NMR titrations using *n-*Bu_3_PO as a reference HB acceptor. Our studies revealed that the ^1^H NMR titrations, although tedious, offered reliable binding affinity data for HB complexes involving neutral and cationic donor molecules. This technique can be employed as a general approach for quantifying the energetics of HB interaction-enabled binding processes. Additionally, the free energies of HB complexation calculated at the PCM(MeCN)-M06-2X/6-31+G(d,p) level correlate well with our experimental data, allowing for binding affinity predictions. Lastly, we found a linear relationship between Δδ_DMSO–CDCl3_ or Δδ_DMSO–CD3NO2_ and hyperconjugative Me_3_PO(LP)→σ*_H–CF2Ar_ interaction energies, providing a quick and feasible estimation of the intrinsic HB donation ability of the CF_2_H moiety. Further studies of the nature of hydrogen bonding interactions involving the CF_2_H group are underway.

## Supporting Information

File 1 Supplementary figures and schemes, materials, experimental procedures; characterization data (1D and 2D NMR, MS, HRMS) for all compounds; titration studies; DFT calculations.

## Data Availability

All data that supports the findings of this study is available in the published article and/or the supporting information of this article.

## References

[1] Wittkopp A, Schreiner P R (2003). Chem – Eur J.

[2] Doyle A G, Jacobsen E N (2007). Chem Rev.

[3] Jeffrey G A (1997). An Introduction to Hydrogen Bonding.

[4] Anslyn E V, Dougherty D A (2006). Modern Physical Organic Chemistry.

[5] Nepal B, Scheiner S (2015). Chem – Eur J.

[6] Struble M D, Strull J, Patel K, Siegler M A, Lectka T (2014). J Org Chem.

[7] Hobza P, Havlas Z (2000). Chem Rev.

[8] Castellano R K (2004). Curr Org Chem.

[9] Ammer J, Nolte C, Karaghiosoff K, Thallmair S, Mayer P, de Vivie‐Riedle R, Mayr H (2013). Chem – Eur J.

[10] Thalladi V R, Weiss H-C, Bläser D, Boese R, Nangia A, Desiraju G R (1998). J Am Chem Soc.

[11] Cai J, Sessler J L (2014). Chem Soc Rev.

[12] Allerhand A, Von Rague Schleyer P (1963). J Am Chem Soc.

[13] Desiraju G R (1996). Acc Chem Res.

[14] Kryachko E, Scheiner S (2004). J Phys Chem A.

[15] Erickson J A, McLoughlin J I (1995). J Org Chem.

[16] Mahjoub A R, Zhang X, Seppelt K (1995). Chem – Eur J.

[17] Caminati W, Melandri S, Moreschini P, Favero P G (1999). Angew Chem, Int Ed.

[18] Jones C R, Baruah P K, Thompson A L, Scheiner S, Smith M D (2012). J Am Chem Soc.

[19] Zafrani Y, Yeffet D, Sod-Moriah G, Berliner A, Amir D, Marciano D, Gershonov E, Saphier S (2017). J Med Chem.

[20] Zafrani Y, Sod-Moriah G, Yeffet D, Berliner A, Amir D, Marciano D, Elias S, Katalan S, Ashkenazi N, Madmon M (2019). J Med Chem.

[21] Sessler C D, Rahm M, Becker S, Goldberg J M, Wang F, Lippard S J (2017). J Am Chem Soc.

[22] Zafrani Y, Parvari G, Amir D, Ghindes-Azaria L, Elias S, Pevzner A, Fridkin G, Berliner A, Gershonov E, Eichen Y (2021). J Med Chem.

[23] Saphier S, Zafrani Y (2024). Future Med Chem.

[24] Columbus I, Ghindes-Azaria L, Chen R, Yehezkel L, Redy-Keisar O, Fridkin G, Amir D, Marciano D, Drug E, Gershonov E (2022). J Med Chem.

[25] Streitwieser A, Mares F (1968). J Am Chem Soc.

[26] Linclau B, Wang Z, Compain G, Paumelle V, Fontenelle C Q, Wells N, Weymouth‐Wilson A (2016). Angew Chem, Int Ed.

[27] O'Hagan D, Young R J (2016). Angew Chem, Int Ed.

[28] Jeffries B, Wang Z, Felstead H R, Le Questel J-Y, Scott J S, Chiarparin E, Graton J, Linclau B (2020). J Med Chem.

[29] Josephson B, Fehl C, Isenegger P G, Nadal S, Wright T H, Poh A W J, Bower B J, Giltrap A M, Chen L, Batchelor-McAuley C (2020). Nature.

[30] Meanwell N A (2018). J Med Chem.

[31] Trifonov A L, Levin V V, Struchkova M I, Dilman A D (2017). Org Lett.

[32] Prakash G K S, Ganesh S K, Jones J-P, Kulkarni A, Masood K, Swabeck J K, Olah G A (2012). Angew Chem, Int Ed.

[33] Fujikawa K, Fujioka Y, Kobayashi A, Amii H (2011). Org Lett.

[34] O'Hagan D (2008). Chem Soc Rev.

[35] Okusu S, Tokunaga E, Shibata N (2015). Org Lett.

[36] Zhang X-Y, Sun S-P, Sang Y-Q, Xue X-S, Min Q-Q, Zhang X (2023). Angew Chem, Int Ed.

[37] Lin D, de los Rios J P, Surya Prakash G K (2023). Angew Chem, Int Ed.

[38] Jia R, Wang X, Hu J (2021). Tetrahedron Lett.

[39] Sap J B I, Meyer C F, Straathof N J W, Iwumene N, am Ende C W, Trabanco A A, Gouverneur V (2021). Chem Soc Rev.

[40] Wei Z, Miao W, Ni C, Hu J (2021). Angew Chem, Int Ed.

[41] Fujiwara Y, Dixon J A, Rodriguez R A, Baxter R D, Dixon D D, Collins M R, Blackmond D G, Baran P S (2012). J Am Chem Soc.

[42] Fier P S, Hartwig J F (2012). J Am Chem Soc.

[43] Surya Prakash G K, Hu J, Olah G A (2001). J Fluorine Chem.

[44] Thompson S, McMahon S A, Naismith J H, O’Hagan D (2016). Bioorg Chem.

[45] Flick A C, Leverett C A, Ding H X, McInturff E, Fink S J, Helal C J, O’Donnell C J (2019). J Med Chem.

[46] Gillis E P, Eastman K J, Hill M D, Donnelly D J, Meanwell N A (2015). J Med Chem.

[47] Abraham M H, Abraham R J, Byrne J, Griffiths L (2006). J Org Chem.

[48] Abraham M H, Abraham R J, Acree W E, Aliev A E, Leo A J, Whaley W L (2014). J Org Chem.

[49] Gilli P, Pretto L, Bertolasi V, Gilli G (2009). Acc Chem Res.

[50] Zafrani Y, Amir D, Yehezkel L, Madmon M, Saphier S, Karton-Lifshin N, Gershonov E (2016). J Org Chem.

[51] Marcus Y (1993). Chem Soc Rev.

[52] Pike S J, Lavagnini E, Varley L M, Cook J L, Hunter C A (2019). Chem Sci.

[53] Machado V G, Stock R I, Reichardt C (2014). Chem Rev.

[54] Reichardt C (1994). Chem Rev.

[55] Reichardt C, Welton T (2010). Solvents and Solvent Effects in Organic Chemistry.

[56] (2001). NBO.

[57] Reed A E, Curtiss L A, Weinhold F (1988). Chem Rev.

