# Supplementary material for: Quantifying the ability of the CF2H group as a hydrogen bond donor
Source: Beilstein J Org Chem. 2025 Jan 20;21:189–99. doi: 10.3762/bjoc.21.11 (PMC11773185; doi:10.3762/bjoc.21.11)
Supplement: File 1 — Supplementary figures and schemes, materials, experimental procedures; characterization data (1D and 2D NMR, MS, HRMS) for all compounds; titration studies; DFT calculations. [file Beilstein_J_Org_Chem-21-189-s001.pdf]

BEILSTEIN JOURNAL OF ORGANIC CHEMISTRY

Supporting Information

for

Quantifying the ability of the CF_2H group as a hydrogen bond donor

Matthew E. Paolella, Daniel S. Honeycutt, Bradley M. Lipka, Jacob M. Goldberg and Fang Wang

Beilstein J. Org. Chem. **2025,** *21,* 189–199. doi:10.3762/bjoc.21.11

Supplementary figures and schemes, materials, experimental procedures; characterization data (1D and 2D NMR, MS, HRMS) for all compounds; titration studies; DFT calculations

License and Terms: This is a supporting information file under the terms of the Creative Commons Attribution License (https://creativecommons.org/licenses/by/4.0). Please note that the reuse, redistribution and reproduction in particular requires that the author(s) and source are credited and that individual graphics may be subject to special legal provisions.
The license is subject to the *Beilstein Journal of Organic Chemistry* terms and conditions: (https://www.beilstein-journals.org/bjoc/terms)

Table of contents

General. S2

Chemical synthesis. S2

NMR chemical shift of hydrogen bond donating moieties in different solvents. S7

UV–vis titration with Reichardt's dye. S11

NMR titration with tri-n-butylphosphine oxide (n-Bu_3PO). S14

Theoretical calculations. S22

NMR spectra. S51

General.

Reagents, including reference compounds **10–13**, were purchased from commercial sources and used as received. Anhydrous solvents were saturated with argon, purified by passaging through two columns of activated alumina, and stored over 3 Å molecular sieves in a dry box. Deuterated solvents for NMR titration experiments were stored over 3 Å molecular sieves in a dry box. Reaction mixtures were monitored by thin-layer chromatography (TLC) on pre-coated, aluminum-backed silica gel 60 F254 plates. Column chromatography was performed on silica gel 60 (230–400 mesh). NMR spectra were recorded on a 400 MHz Bruker Avance NMR spectrometer. ^{1}H and ^{13}C chemical shifts are reported in ppm relative to $SiMe_4$ (δ = 0.00 ppm). ^{1}H and ^{13}C NMR spectra were referenced internally to residual solvent peaks. ^{19}F NMR spectra were referenced externally to $CFCl_3$ (0.0 ppm). Low-resolution electrospray mass spectra were acquired on a Shimadzu LCMS-2020 spectrometer or an Agilent InfinityLab LC/MSD iQ system. High-resolution mass spectra and MS/MS analyses were acquired on an AB SCIEX TripleTOF 4600 mass spectrometer equipped with a DuoSpray™ ion source. UV–visible spectra were recorded on an Agilent Cary 60 UV-visible spectrophotometer. Cuvettes with 1.00 cm path lengths were used for all spectroscopic measurements.

Chemical synthesis.

2-(Difluoromethyl)-*N*-methylpyridinium tetrafluoroborate (1b)

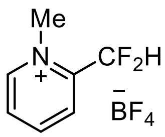

A portion of 2-(difluoromethyl)pyridine (64.6 mg, 0.50 mmol, commercially available) was dissolved in anh. nitromethane (500 μL) in a vial in a glovebox. Trimethyloxonium tetrafluoroborate (81.3 mg, 0.55 mmol) was added to this mixture in one portion. The reaction was stirred at rt for 12 h. The reaction mixture was diluted with anh. MeCN (3 mL) and filtered through a cotton plug. The mixture was layered with anh. Et_2O (20 mL). A white solid formed after three days. The solid was collected and washed with anh. Et_2O. The solid was dried under vacuum (107.8 mg, 93% yield). ^{1}H NMR (400 MHz, CD_3CN) δ 8.81 (d, *J* = 5.7 Hz, 1H), 8.68 (t, *J* = 7.9 Hz, 1H), 8.30 (d, *J* = 7.9 Hz, 1H), 8.17 (t, *J* = 6.9 Hz, 1H), 7.25 (t, *J* = 51.6 Hz, 1H), 4.36 (s, 3H). ^{13}C{^{1}H} NMR (101 MHz, CD_3CN) δ 149.87, 148.48, 146.93 (t, *J* = 28.4 Hz), 131.08, 127.42 (t, *J* = 6.7 Hz), 110.13 (t, *J* = 242.0 Hz), 47.19. ^{19}F NMR (377 MHz, CD_3CN) δ –122.23 (d, *J* = 51.6 Hz, 2F), –151.68 and –151.78 (s, 4F). ESI-MS(+) m/z calcd for $[M]^+$ 144.1, found 144.1. ESI-HRMS(+) m/z calcd for $C_7H_8F_2N^+$ $[M]^+$ 144.0620, found 144.0629.

2-(Difluoromethyl)quinoline (2a)

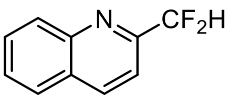

A portion of DAST (330 μL, 403 mg, 2.50 mmol) was added dropwise to a PTFE vial containing quinoline-2-carbaldehyde (157 mg, 1.00 mmol) and CH_2Cl_2 (1.0 mL). A small portion of methanol (10 μL) was added to this mixture. The reaction was stirred overnight and quenched by dropwise addition of sat. $NaHCO_3$ aq. solution (10 mL). The mixture was extracted with CH_2Cl_2 (10 mL × 4). The combined organic phase was dried over $MgSO_4$ and dried under vacuum. The crude product was purified by column chromatography (100% CH_2Cl_2) on silica gel to afford a slightly yellow oil (107.0 mg, 59% yield). ^{1}H NMR (400 MHz, $CDCl_3$) δ 8.33 (d, *J* = 8.5 Hz, 1H), 8.15 (d, *J* = 8.5 Hz, 1H), 7.89 (d, *J* = 8.2 Hz, 1H), 7.79 (t, *J* = 7.7 Hz, 1H), 7.74 (d, *J* = 8.5 Hz, 1H), 7.64 (t, *J* = 7.5 Hz, 1H), 6.79 (t, *J* = 55.3 Hz, 1H). ^{19}F NMR (377 MHz, $CDCl_3$) δ -114.18 (d, *J* = 55.6 Hz). ESI-MS(+) m/z calcd for [M+H]+ 180.1, found 180.0. Spectroscopic data were consistent with reported values.[1]

2-(Difluoromethyl)-*N*-methylquinolinium tetrafluoroborate (2b)

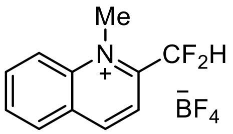

A portion of 2-(difluoromethyl)quinoline (44.8 mg, 0.25 mmol) was suspended in anh. nitromethane (250 μL) in a vial in a glovebox. Trimethyloxonium tetrafluoroborate (38.8 mg, 0.26 mmol) was added to this mixture in one portion. The reaction was stirred at rt for 24 h. The reaction mixture was diluted with anh. MeCN (0.5 mL) and filtered through a cotton plug. Anh. Et_2O was diffused into this mixture. The solid was collected after 3 days, washed with anh. Et_2O, and dried under vacuum. Colorless sheets were obtained (58.2 mg, 83%). ^1H NMR (400 MHz, CD_3CN) δ 9.31 (d, *J* = 8.6 Hz, 1H), 8.53 (d, *J* = 9.1 Hz, 1H), 8.45 (d, *J* = 8.3 Hz, 1H), 8.37 (t, *J* = 7.4 Hz, 1H), 8.34 (d, *J* = 8.8 Hz, 1H), 8.12 (t, *J* = 7.6 Hz, 1H), 7.52 (t, *J* = 51.6 Hz, 1H), 4.57 (s, 3H). $^{13}C\{^1H\}$ NMR (101 MHz, CD_3CN) δ 149.49, 149.30 (t, *J* = 26.7 Hz), 140.52, 137.68, 131.29, 130.90, 130.59, 119.56 (t, *J* = 7.5 Hz), 119.02, 109.55 (t, *J* = 243.2 Hz), 40.61. ^{19}F NMR (377 MHz, CD_3CN) δ −120.61 (d, *J* = 51.6 Hz, 2F), −151.68 and −151.86 (s, 4F). ESI-MS(+) m/z calcd for $[M]^+$ 194.1, found 194.0. ESI-HRMS(+) m/z calcd for $C_{11}H_{10}F_2N^+$ $[M]^+$ 194.0776, found 194.0796.

2-(Difluoromethyl)-1-methyl-benzoimidazole (3a)

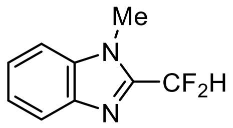

A portion of 2-(difluoromethyl)benzimidazole (168 mg, 1.0 mmol, commercially available) was mixed with K_2CO_3 (276 mg, 2.0 mmol) and CH_3I (187 μL, 3.0 mmol) in anh. MeCN (2.0 mL) in a Schlenk flask under N_2. The mixture was stirred at 65 °C. The reaction was monitored by TLC. After 4 h, the mixture was cooled to rt and filtered. The filtrate was concentrated under vacuum. The crude product was purified by column chromatography (hexanes:ethyl acetate 5:1) on silica gel to afford a white solid (139 mg, 76% yield). ^1H NMR (400 MHz, $CDCl_3$) δ 7.83 (d, *J* = 8.1 Hz, 1H), 7.51 – 7.31 (m, 3H), 6.94 (t, *J* = 52.5 Hz, 1H), 3.99 (s, 3H). ^{19}F NMR (377 MHz, $CDCl_3$) δ −113.80 (d, *J* = 52.5 Hz, 2F). Spectroscopic data were consistent with reported values.[1]

2-(Difluoromethyl)-1,3-dimethyl-benzoimidazolium tetrafluoroborate (3b)

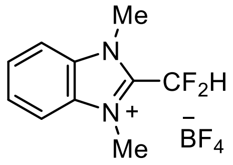

A portion of 2-(difluoromethyl)-1-methyl-benzoimidazole (36.4 mg, 0.20 mmol) was dissolved in anh. nitromethane (200 μL) in a vial in a glovebox. Trimethyloxonium tetrafluoroborate (32.5 mg, 0.22 mmol) was added to this mixture in one portion. The reaction was stirred at rt for 12 h. The reaction mixture was diluted with anh. MeCN (1 mL) and filtered through a cotton plug. Anh. Et_2O was diffused into this mixture. The solid was collected after 24 h, washed with anh. Et_2O, and dried under vacuum. Colorless crystals were obtained (56.5 mg, 99%). ^1H NMR (400 MHz, CD_3CN) δ 8.01 – 7.91 (m, 2H), 7.87 – 7.77 (m, 2H), 7.51 (t, *J* = 49.3 Hz, 1H), 4.17 (s, 6H). $^{13}C\{^1H\}$ NMR (101 MHz, CD_3CN) δ 141.31 (t, *J* = 29.3 Hz), 133.06, 129.49, 114.67, 107.20 (t, *J* = 241.8 Hz), 34.03 (t, *J* = 2.4 Hz). ^{19}F NMR (377 MHz, CD_3CN) δ −120.20 (d, *J* = 49.3 Hz, 2F), −150.87 and −150.92 (s, 4F). ESI-MS(+) m/z calcd for $[M]^+$ 144.1, found 144.1. ESI-HRMS(+) m/z calcd for $C_7H_8F_2N^+$ $[M]^+$ 144.0620, found 144.0621.

4-(Difluoromethyl)-*N*-methylpyridinium tetrafluoroborate (4b)

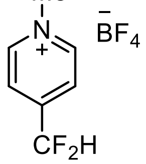

A portion of 4-(difluoromethyl)pyridine (64.6 mg, 0.50 mmol, commercially available) was dissolved in anh. nitromethane (500 μL) in a vial in a glovebox. Trimethyloxonium tetrafluoroborate (81.3 mg, 0.55 mmol) was added to this mixture in one portion. The reaction was stirred at rt for 12 h. The reaction mixture was diluted with anh. MeCN (3 mL) and filtered through a cotton plug. The mixture was layered with anh. Et_2O (20 mL) and stored at −40 °C. A white solid formed after three days. The solid was collected and washed with anh. Et_2O. The solid was dried under vacuum (89.1 mg, 77% yield). ^1H NMR (400 MHz, CD_3CN) δ 8.79 (d, *J* = 6.4 Hz, 2H), 8.15 (br, 2H), 7.05 (t, *J* = 54.1 Hz, 1H), 4.36 (s, 3H). $^{13}C\{^1H\}$ NMR (101 MHz, CD_3CN) δ 151.05 (t, *J* = 24.8 Hz),

147.67 (t, J = 9.3 Hz), 126.01 (t, J = 5.9 Hz), 112.65 (t, J = 241.1 Hz), 49.68 (t, J = 5.0 Hz). ^{19}F NMR (377 MHz, CD_3CN) δ −119.51 (d, J = 54.1 Hz, 2F), −151.67 and −151.72 (s, 4F). ESI-MS(+) m/z calcd for $[M]^+$ 144.1, found 144.1. ESI-HRMS(+) m/z calcd for $C_7H_8F_2N^+$ $[M]^+$ 144.0620, found 144.0621.

4-(Difluoromethyl)quinoline (5a)

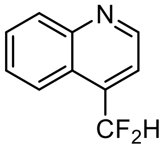

A portion of DAST (495 μL, 605 mg, 3.75 mmol) was added dropwise to a PTFE vial containing 4-quinolinecarboxaldehyde (236 mg, 1.50 mmol) and CH_2Cl_2 (1.5 mL). A small portion of methanol (15 μL) was added to this mixture. The reaction was stirred overnight and quenched by dropwise addition of sat. $NaHCO_3$ aq. solution (10 mL). The mixture was extracted with CH_2Cl_2 (10 mL × 4). The combined organic phase was dried over $MgSO_4$ and dried under vacuum. The crude product was purified by column chromatography (ethyl acetate:hexanes 1:3) on silica gel to afford a white solid (159 mg, 59% yield). ^{1}H NMR (400 MHz, $CDCl_3$) δ 9.03 (d, J = 3.4 Hz, 1H), 8.22 (d, J = 8.5 Hz, 1H), 8.10 (dq, J = 8.4, 0.9 Hz, 1H), 7.81 (ddd, J = 8.4, 7.0, 1.2 Hz, 1H), 7.67 (ddd, J = 8.2, 7.0, 1.2 Hz, 1H), 7.61 (d, J = 4.2 Hz, 1H), 7.17 (t, J = 54.5 Hz, 1H). ^{19}F NMR (377 MHz, $CDCl_3$) δ −115.12 (d, J = 54.5 Hz, 2F). ESI-MS(+) m/z calcd for $[M+H]^+$ 180.1, found 180.0. Spectroscopic data were consistent with reported values.[2]

4-(Difluoromethyl)-*N*-methylquinolinium tetrafluoroborate (5b)

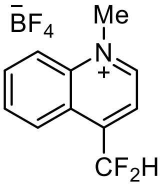

A portion of 4-(difluoromethyl)quinoline (44.8 mg, 0.25 mmol) was suspended in anh. nitromethane (250 μL) in a vial in a glovebox. Trimethyloxonium tetrafluoroborate (38.8 mg, 0.26 mmol) was added to this mixture in one portion. The reaction was stirred at rt for 24 h. The reaction mixture was diluted with anh. MeCN (0.5 mL) and filtered through a cotton plug. Anh. Et_2O was diffused into this mixture. The solid was collected after 3 d, washed with anh. Et_2O, and dried under vacuum. Colorless sheets were obtained (67.0 mg, 95%). ^{1}H NMR (400 MHz, CD_3CN) δ 9.25 (d, J = 5.9 Hz, 1H), 8.50 (d, J = 8.3 Hz, 1H), 8.48 (d, J = 8.9 Hz, 1H), 8.33 (t, J = 8.0 Hz, 1H), 8.23 (d, J = 5.8 Hz, 1H), 8.14 (t, J = 7.8 Hz, 1H), 7.61 (t, J = 52.9 Hz, 1H), 4.63 (s, 3H). ^{19}F NMR (377 MHz, CD_3CN) δ −118.59 (d, J = 52.9 Hz, 2F), −151.64 and −151.75 (s, 4F). $^{13}C\{^1H\}$ NMR (101 MHz, CD_3CN) δ 150.38, 147.78 (t, J = 23.3 Hz), 139.33, 136.09, 131.43, 126.20 (t, J = 3.1 Hz), 125.85, 119.74, 119.15 (t, J = 8.5 Hz), 111.80 (t, J = 241.0 Hz), 46.48. ESI-MS(+) m/z calcd for $[M]^+$ 194.1, found 194.0. ESI-HRMS(+) m/z calcd for $C_{11}H_{10}F_2N^+$ $[M]^+$ 194.0776, found 194.0808.

9-(Difluoromethyl)acridine (6a)

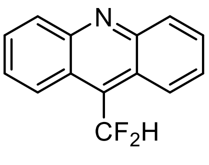

A portion of DAST (165 μL, 202 mg, 1.25 mmol) was added dropwise to a PTFE vial containing acridin-4-carbaldehyde (104 mg, 0.50 mmol) and CH_2Cl_2 (0.5 mL). A small portion of methanol (5 μL) was added to this mixture. The reaction was stirred overnight and quenched by dropwise addition of sat. $NaHCO_3$ aq. solution (5 mL). The mixture was extracted with CH_2Cl_2 (5 mL × 4). The combined organic phase was dried over $MgSO_4$ and dried under vacuum. The crude product was purified by column chromatography (ethyl acetate:hexanes 1:4) on silica gel to afford a white solid (85 mg, 74% yield). ^{1}H NMR (400 MHz, $CDCl_3$) δ 8.45 (d, J = 8.8 Hz, 2H), 8.35 (d, J = 8.6 Hz, 2H), 7.95 (t, J = 53.3 Hz, 1H), 7.85 (pseudo t, J = 7.5 Hz, 2H), 7.68 (ddd, J = 7.7, 6.6, 0.9 Hz, 2H). ^{19}F NMR (377 MHz, $CDCl_3$) δ −108.49 (d, J = 53.5 Hz, 2F). ESI-MS(+) m/z calcd for [M+H]+ 230.1, found 230.0 Spectroscopic data were consistent with reported values.[2]

***N*-Methyl-9-(difluoromethyl)acridinium (6b)**

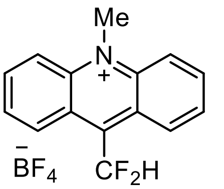

A portion of 9-(difluoromethyl)acridine (34.4 mg, 0.15 mmol) was suspended in anh. nitromethane (150 µL) in a vial in a glovebox. Trimethyloxonium tetrafluoroborate (23.3 mg, 0.16 mmol) was added to this mixture in one portion. The reaction was stirred at rt for 24 h. The reaction mixture was diluted with anh. MeCN (0.5 mL) and filtered through a cotton plug. Anh. Et_2O was diffused into this mixture. The solid was collected after three days, washed with anh. Et_2O, and dried under vacuum. Bright yellow blocks were obtained (43.9 mg, 88%). ^{1}H NMR (400 MHz, CD_3CN) δ 8.90 (d, J = 8.9 Hz, 2H), 8.68 (d, J = 9.4 Hz, 2H), 8.47 (t, J = 8.1 Hz, 2H), 8.30 (t, J = 51.6 Hz, 1H), 8.10 (t, J = 8.1 Hz, 2H), 4.87 (s, 3H). ^{13}C{^{1}H} NMR (101 MHz, CD_3CN) δ 146.18 (t, J = 22.5 Hz), 143.25, 140.01, 130.27, 127.39 (t, J = 3.1 Hz), 125.30 (t, J = 2.6 Hz), 120.37, 112.61 (t, J = 241.4 Hz), 41.21. ^{19}F NMR (377 MHz, CD_3CN) δ −109.68 (d, J = 51.6 Hz), −151.74 and −151.80 (m, 4F). ESI-MS(+) m/z calcd for $[M]^+$ 244.1, found 244.0. ESI-HRMS(+) m/z calcd for $C_{15}H_{12}F_2N^+$ $[M]^+$ 244.0933, found 244.0934.

2-(Difluoromethyl)-6-methoxy-pyridine (7a)

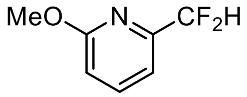

DAST (495 µL, 3.75 mmol) was added dropwise to a PTFE vial containing 6-methoxypicolinaldehyde (206 mg, 1.50 mmol) in CH_2Cl_2 (1.5 mL, partially soluble) dropwise. A dark black solution formed. A portion of MeOH (15 µL) was added to this mixture. After 10 min, the reaction turned dark yellow. The reaction was stirred overnight and generated a dark black mixture. The mixture was added dropwise to sat. $NaHCO_3$ aq. solution and extracted with CH_2Cl_2 (10 mL × 4). The combined organic phase was dried over $MgSO_4$. The crude product was purified by column chromatography (CH_2Cl_2:hexanes 10:1) on silica gel to afford a white solid (71.0 mg, 30% yield). ^{1}H NMR (400 MHz, $CDCl_3$) δ 7.67 (t, J = 7.8 Hz, 1H), 7.19 (d, J = 7.3 Hz, 1H), 6.83 (d, J = 8.3 Hz, 1H), 6.51 (t, J = 55.7 Hz, 1H), 3.95 (s, 3H). ^{19}F NMR (377 MHz, $CDCl_3$) δ −116.25 (d, J = 55.7 Hz, 2F). ^{13}C{^{1}H} NMR (101 MHz, $CDCl_3$) δ 164.05, 150.34 (t, J = 25.6 Hz), 139.47, 113.79 (t, J = 240.0 Hz), 113.21 (t, J = 1.4 Hz), 112.87 (t, J = 3.8 Hz), 53.74. ESI-MS(+) m/z calcd for $[M+H]^+$ 160.1, found 160.1.

2-(Difluoromethyl)-6-methoxy-*N*-methylpyridinium tetrafluoroborate (7b)

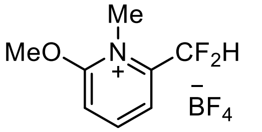

A portion of 2-(difluoromethyl)-6-methoxy-pyridine (58.3 mg, 0.37 mmol) was dissolved in anh. nitromethane (370 µL) in a vial in a glovebox. Trimethyloxonium tetrafluoroborate (62.1 mg, 0.42 mmol) was added to this mixture in one portion. The reaction was stirred at rt for 12 h. The reaction mixture was diluted with anh. MeCN (200 µL) and filtered through a cotton plug. Anh. Et_2O was diffused into the mixture. White needles formed after three days. The solid was collected and washed with anh. Et_2O. The solid was dried under vacuum (79.6 mg, 91% yield). ^{1}H NMR (400 MHz, CD_3CN) δ 8.51 (psuedo t, J = 8.3 Hz, 1H), 7.75 (d, J = 7.6 Hz, 1H), 7.71 (d, J = 9.1 Hz, 1H), 7.16 (t, J = 51.8 Hz, 1H), 4.31 (s, 3H), 3.96 (s, 3H). ^{13}C{^{1}H} NMR (101 MHz, CD_3CN) δ 163.23, 148.95, 144.21 (t, J = 25.5 Hz), 118.62 (t, J = 7.8 Hz), 115.13 (br), 110.65 (t, J = 241.5 Hz), 61.18, 37.29 (t, J = 2.7 Hz). ^{19}F NMR (376 MHz, CD_3CN) δ −120.58 (d, J = 51.8 Hz, 2F), −151.89 and −151.95 (s, 4F). ESI-MS(+) m/z calcd for $[M]^+$ 174.1, found 174.0. ESI-HRMS(+) m/z calcd for $C_8H_{10}F_2NO^+$ $[M]^+$ 174.0725, found 174.0746.

2-(Difluoromethyl)-6-methyl-pyridinium (8a)

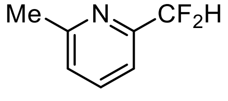

A portion of DAST (495 µL, 3.75 mmol) was added dropwise to a PTFE vial containing 2-acetylpyridine (182 mg, 1.50 mmol) in CH_2Cl_2 (1.5 mL) dropwise. A dark black solution formed. A portion of MeOH (15 µL) was added to this mixture. The reaction was stirred overnight. The mixture was added dropwise to sat. $NaHCO_3$ aq. solution and extracted with CH_2Cl_2 (10 mL×4). The combined organic phase was

dried over $MgSO_4$. The crude product was purified by column chromatography (CH_2Cl_2:hexanes 10:1) on silica gel to afford a volatile yellow oil (48 mg, 22% yield). ^{1}H NMR (400 MHz, $CDCl_3$) δ 7.72 (t, *J* = 7.7 Hz, 1H), 7.44 (d, *J* = 7.7 Hz, 1H), 7.26 (d, *J* = 7.7 Hz, 1H), 6.60 (t, *J* = 55.6 Hz, 1H), 2.60 (s, 3H). ^{19}F NMR (377 MHz, $CDCl_3$) δ −115.70 (d, *J* = 55.7 Hz, 2F). ESI-MS(+) m/z calcd for $[M]^+$ 144.1, found 144.1.

2-(Difluoromethyl)-1,6-dimethyl-pyridinium tetrafluoroborate (8b)

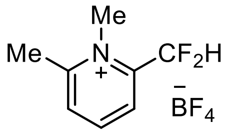

A portion of 2-(difluoromethyl)-6-methyl-pyridine (36.1 mg, 0.25 mmol, commercially available) was dissolved in anh. nitromethane (250 μL) in a vial in a glovebox. Trimethyloxonium tetrafluoroborate (44.4 mg, 0.30 mmol) was added to this mixture in one portion. The reaction was stirred at rt for 12 h. The reaction mixture was diluted with anh. MeCN (150 μL) and filtered through a cotton plug. Anh. Et_2O was diffused into the mixture. A white solid formed after three days. The solid was collected and washed with anh. Et_2O. The solid was dried under vacuum (35.2 mg, 57% yield). ^{1}H NMR (400 MHz, CD_3CN) δ 8.50 (t, *J* = 8.0 Hz, 1H), 8.12 (d, *J* = 7.8 Hz, 1H), 8.06 (d, *J* = 8.1 Hz, 1H), 7.25 (t, *J* = 51.7 Hz, 1H), 4.13 (s, 3H), 2.83 (s, 3H). ^{19}F NMR (376 MHz, CD_3CN) δ −120.78 (d, *J* = 51.7 Hz, 2F), −151.85 and −151.91 (s, 4F). ^{13}C{^{1}H} NMR (101 MHz, CD_3CN) δ 160.92, 147.00 (two overlapping carbon signals), 133.11, 125.38 (t, *J* = 7.5 Hz), 110.62 (t, *J* = 242.0 Hz), 42.00, 22.10. ESI-MS(+) m/z calcd for $[M]^+$ 158.1, found 158.1. ESI-HRMS(+) m/z calcd for $C_8H_{10}F_2N^+$ $[M]^+$ 158.0776, found 158.0796.

2-(1,1-Difluoroethyl)pyridine (9a)

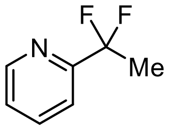

A portion of DAST (495 μL, 3.75 mmol) was added dropwise to a PTFE vial containing 2-acetylpyridine (182 mg, 1.50 mmol) in CH_2Cl_2 (1.5 mL) dropwise. A dark yellow solution formed. A portion of MeOH (15 μL) was added to this mixture. The reaction was stirred overnight and generated a dark red mixture. The mixture was added dropwise to sat. $NaHCO_3$ aq. solution and extracted with CH_2Cl_2 (10 mL × 4). The combined organic phase was dried over $MgSO_4$. The crude product was purified by column chromatography (CH_2Cl_2) on silica gel to afford a volatile yellow oil (130 mg, 60% yield). ^{1}H NMR (400 MHz, $CDCl_3$) δ 8.65 (d, *J* = 4.8 Hz, 1H), 7.80 (td, *J* = 7.8, 1.6 Hz, 1H), 7.65 (dt, *J* = 7.9, 0.9 Hz, 1H), 7.36 (dd, *J* = 7.1, 5.2 Hz, 1H), 2.02 (t, *J* = 18.7 Hz, 3H). ^{19}F NMR (377 MHz, $CDCl_3$) δ −90.93 (q, *J* = 18.7 Hz, 2F). These data are consistent with published results.[3] ESI-MS(+) m/z calcd for $[M+H]^+$ 144.1, found 144.1.

2-(1,1-Difluoroethyl)-*N*-methylpyridinium tetrafluoroborate (9b)

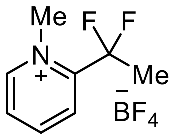

A portion of 2-(1,1-difluoroethyl)-pyridine (73.5 mg, 0.50 mmol) was dissolved in anh. nitromethane (500 μL) in a vial in a glovebox. Trimethyloxonium tetrafluoroborate (88.7 mg, 0.60 mmol) was added to this mixture in one portion. The reaction was stirred at rt for 12 h. The reaction mixture was diluted with anh. MeCN (200 μL) and filtered through a cotton plug. Anh. Et_2O was diffused into the mixture. A yellow oil formed after two days. The oil was collected and dried under vacuum (93.4 mg, 73% yield). ^{1}H NMR (400 MHz, CD_3CN) δ 8.80 (d, *J* = 6.1 Hz, 1H), 8.64 (t, *J* = 8.0 Hz, 1H), 8.28 (d, *J* = 8.1 Hz, 1H), 8.14 (t, *J* = 6.9 Hz, 1H), 4.42 (s, 3H), 2.20 (t, *J* = 19.7 Hz, 3H). ^{13}C{^{1}H} NMR (101 MHz, CD_3CN) δ 150.81, 149.01 (t, *J* = 30.4 Hz), 148.38, 130.75, 128.19 (t, *J* = 6.8 Hz), 120.16 (t, *J* = 243.3 Hz), 48.86 (t, *J* = 5.4 Hz), 24.35 (t, *J* = 26.1 Hz). ^{19}F NMR (377 MHz, CD_3CN) δ −88.33 (q, *J* = 19.6 Hz, 2F), −151.62 and −151.67 (s, 4F). ESI-MS(+) m/z calcd for $[M]^+$ 158.1, found 158.1. ESI-HRMS(+) m/z calcd for $C_8H_{10}F_2N^+$ $[M]^+$ 158.0776, found 158.0794.

NMR chemical shift of hydrogen bond donating moieties in different solvents.

All NMR samples in DMSO-d_6 and CD_3NO_2 were prepared in anhydrous solvents, which were dried over 3 Å molecular sieves in the glovebox for at least 24 h before use. All ^{1}H NMR spectra were acquired with samples at 2 mM on a 400 MHz Bruker Avance NMR spectrometer with 128 scans.

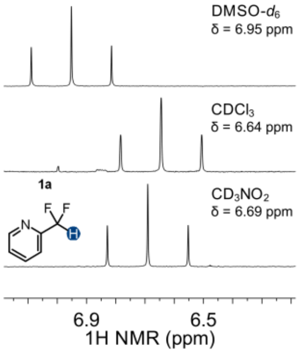

Figure S1. ^{1}H NMR spectra showing the signals of the CF_2H group of **1a** in different solvents.

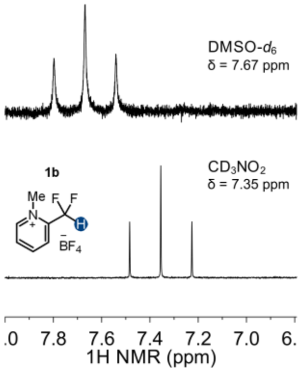

Figure S2. ^{1}H NMR spectra showing the signals of the CF_2H group of **1b** in different solvents.

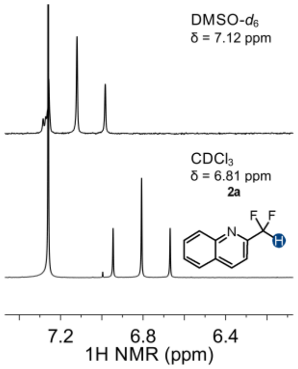

Figure S3. ^{1}H NMR spectra showing the signals of the CF_2H group of **2a** in different solvents.

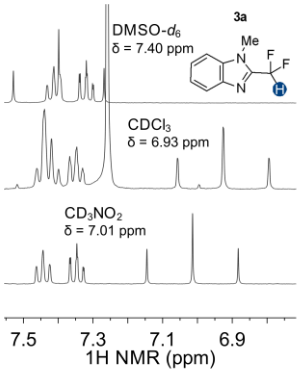

Figure S4. ^{1}H NMR spectra showing the signals of the CF_2H group of **3a** in different solvents.

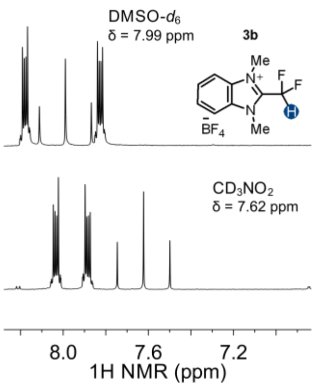

Figure S5. ^{1}H NMR spectra showing the signals of the CF_2H group of **3b** in different solvents.

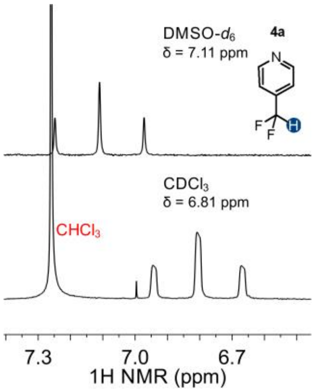

Figure S6. ^{1}H NMR spectra showing the signals of the CF_2H group of **4a** in different solvents.

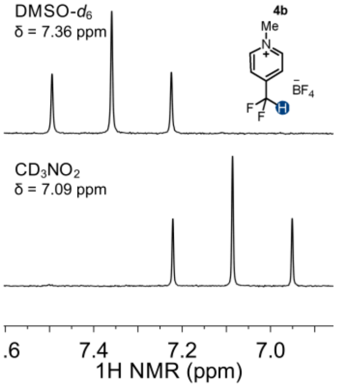

Figure S7. ^{1}H NMR spectra showing the signals of the CF_2H group of **4b** in different solvents.

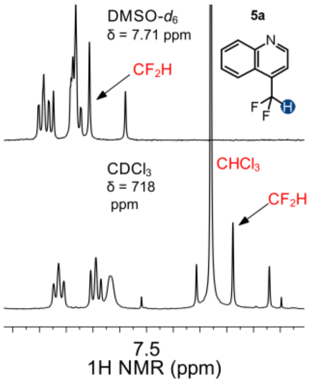

Figure S8. ^{1}H NMR spectra showing the signals of the CF_2H group of **5a** in different solvents.

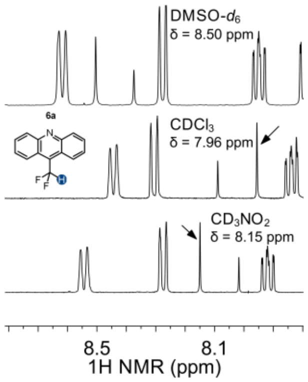

Figure S9. ^{1}H NMR spectra showing the signals of the CF_2H group of **6a** in different solvents.

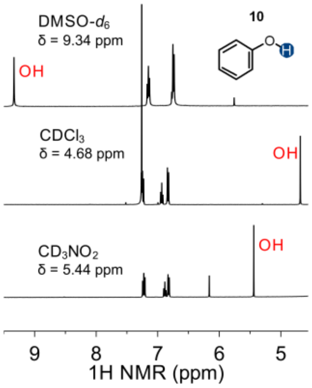

Figure S10. ^{1}H NMR spectra showing the signals of the OH group of **10** in different solvents.

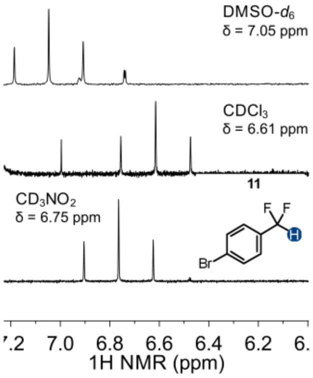

Figure S11. ^{1}H NMR spectra showing the signals of the CF_2H group of **11** in different solvents.

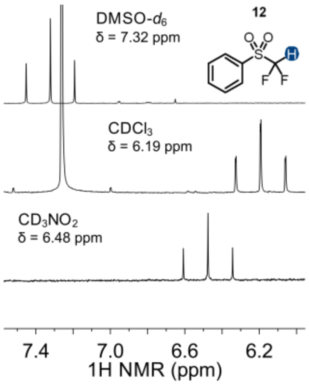

Figure S12. ^{1}H NMR spectra showing the signals of the CF_2H group of **12** in different solvents.

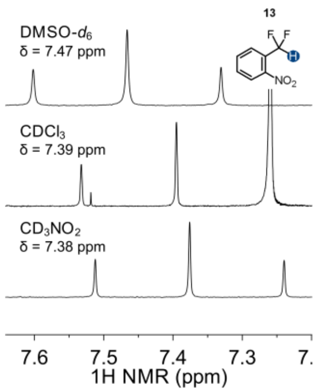

Figure S13. ^{1}H NMR spectra showing the signals of the CF_2H group of **13** in different solvents.

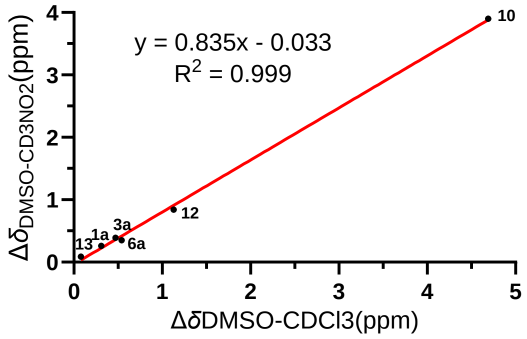

Figure S14. Linear correlation between $\Delta\delta_{DMSO\text{-}CD3NO2}$ and $\Delta\delta_{DMSO\text{-}CDCl3}$ with the data for phenol (**10**) included.

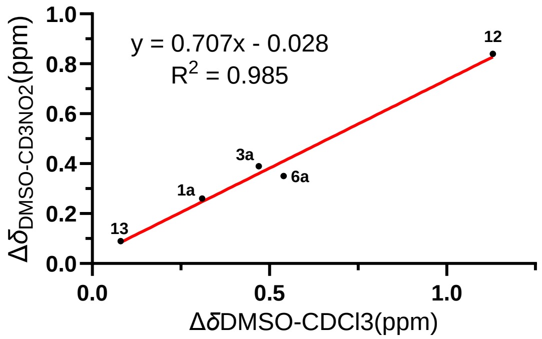

Figure S15. Linear correlation between $\Delta\delta_{DMSO\text{-}CD3NO2}$ and $\Delta\delta_{DMSO\text{-}CDCl3}$ with the data for phenol (**10**) excluded.

UV–vis titration with Reichardt's dye.

All UV–vis titrations were performed on an Agilent Cary 60 UV-visible spectrophotometer. The titration protocol was adapted from an established method.[4] A solution of Reichardt's dye in anhydrous acetonitrile (2.5 mM, 5.0 mL) was prepared in the glovebox. The stock solution of the hydrogen bond donating compound was prepared by dissolving it in anhydrous acetonitrile containing 25 μM Reichardt's dye. The concentration of hydrogen bond donating compound ranged between 0.25 M and 1 M. To determine the binding affinity between the hydrogen bond donor and Reichardt's dye, an aliquot of the Reichardt's dye solution (20 μL, 2.5 mM) was added to anhydrous acetonitrile (1980 μL, stored out of the glovebox for less than 8 h) in a cuvette equipped with a PTFE cap, giving a dye concentration of 25 μM. The UV–vis spectrum was then recorded. Aliquots of the hydrogen bond donating compound solution, as described above, were successively added to the cuvette. The UV–vis spectrum was recorded after each addition. The concentration of Reichardt's dye–hydrogen bond donor complex was calculated based on the UV–vis absorbance at 628 nm, which corresponded to the concentration of free Reichardt's dye. The data were fit to a one-site specific binding model as

$$[\text{Dye} - \text{HB donor complex}] = \frac{B_{max}[\text{HB donor}]}{K_\text{d} + [\text{HB donor}]}$$

Table 1. Summary of UV–vis titration results.

Compound	K_d (mM)	B_{max}	R^2
**1a**	7.8×10^{1}	6.54×10^{-6}	0.980
**1b**	1.9×10^{1}	4.23×10^{-5}	0.997
**3a**	1.3×10^{1}	8.30×10^{-6}	0.986
**3b**	2.9×10^{-1}	4.09×10^{-5}	0.984
**10**	6.3×10^{0}	2.34×10^{-6}	0.999
**12**	3.7×10^{2}	4.94×10^{-5}	0.999

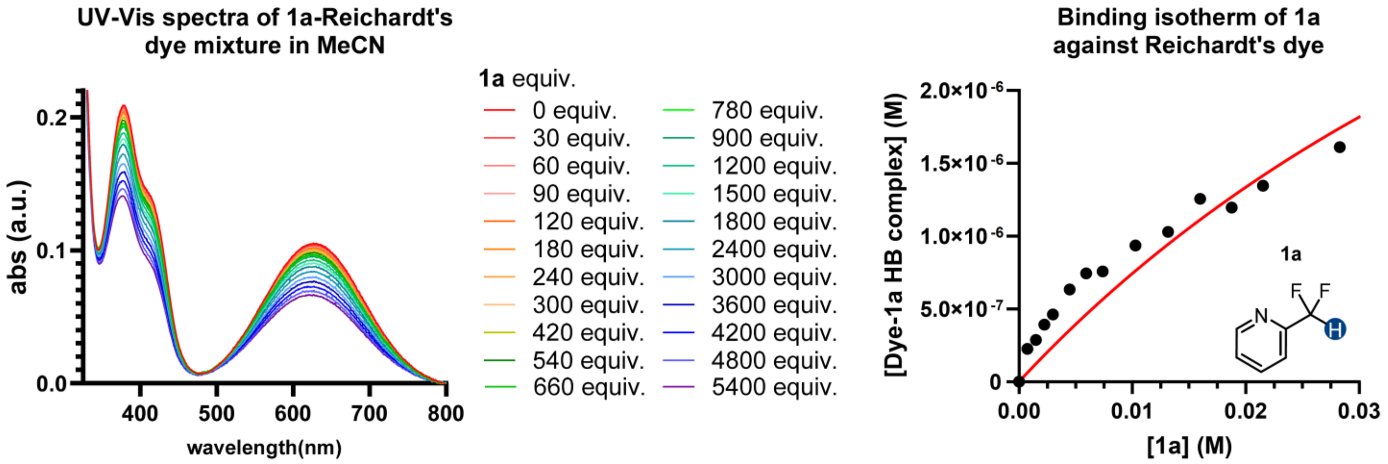

Figure S16. UV–vis titration of Reichardt's dye with 2-(difluoromethyl)pyridine (**1a**) in MeCN.

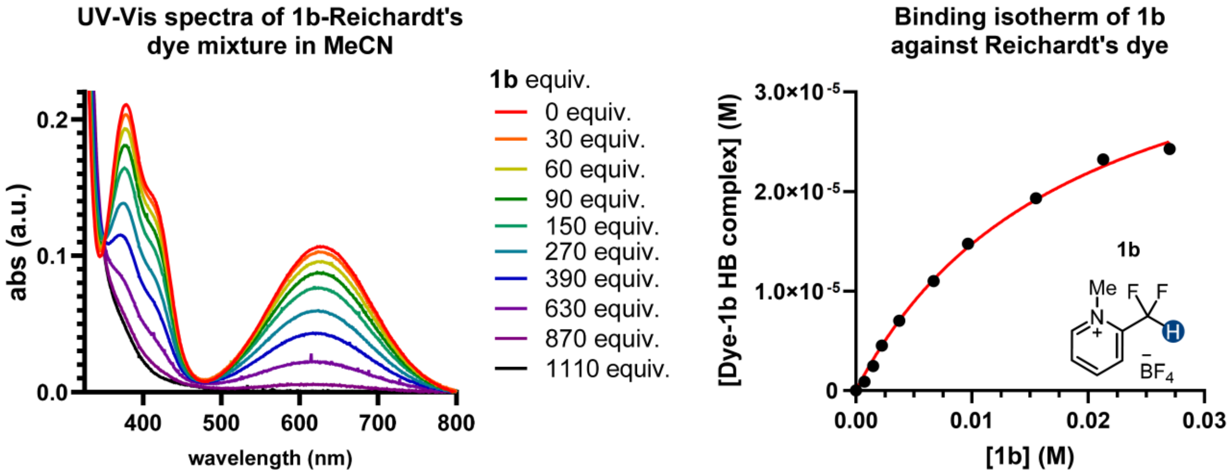

Figure S17. UV–vis titration of Reichardt's dye with 2-(difluoromethyl)-*N*-methylpyridinium tetrafluoroborate (**1b**) in MeCN.

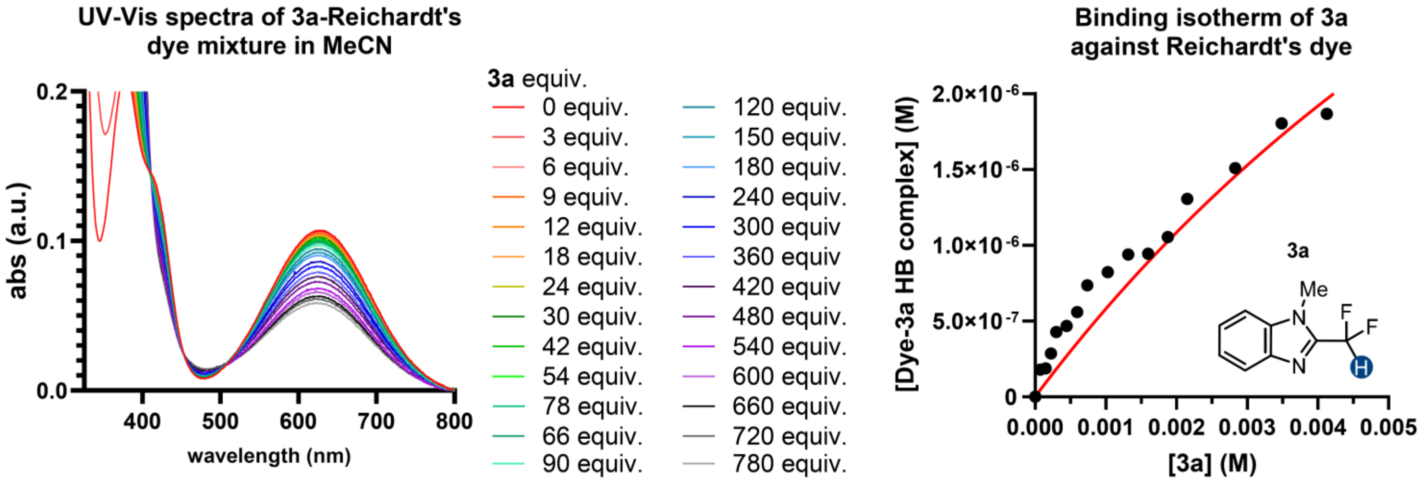

Figure S18. UV–vis titration of Reichardt's dye with 2-(difluoromethyl)-1-methyl-benzoimidazole (**3a**) in MeCN.

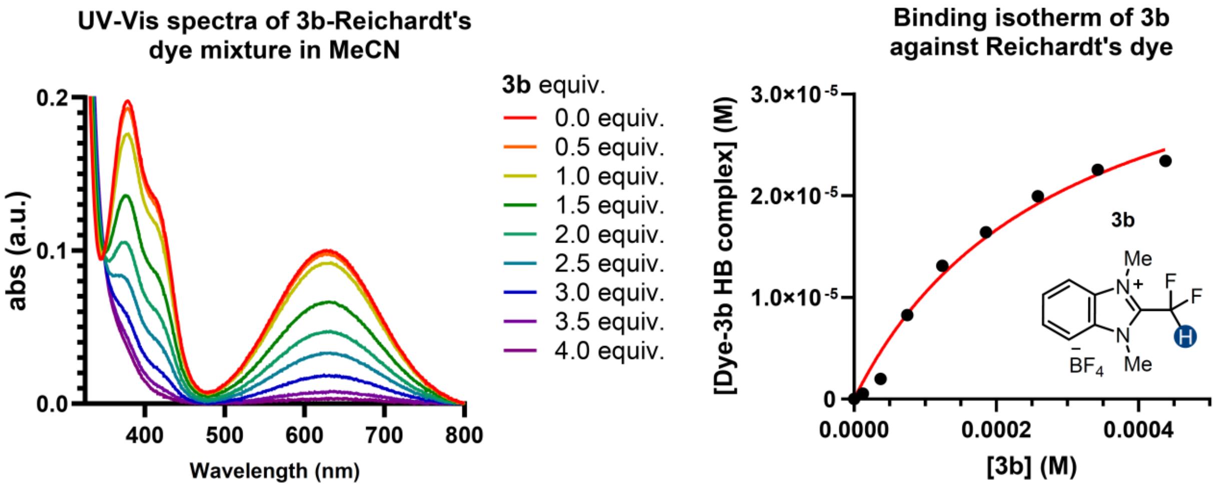

Figure S19. UV–vis titration of Reichardt's dye with 2-(difluoromethyl)-1,3-dimethyl-benzoimidazolium tetrafluoroborate (**3b**) in MeCN.

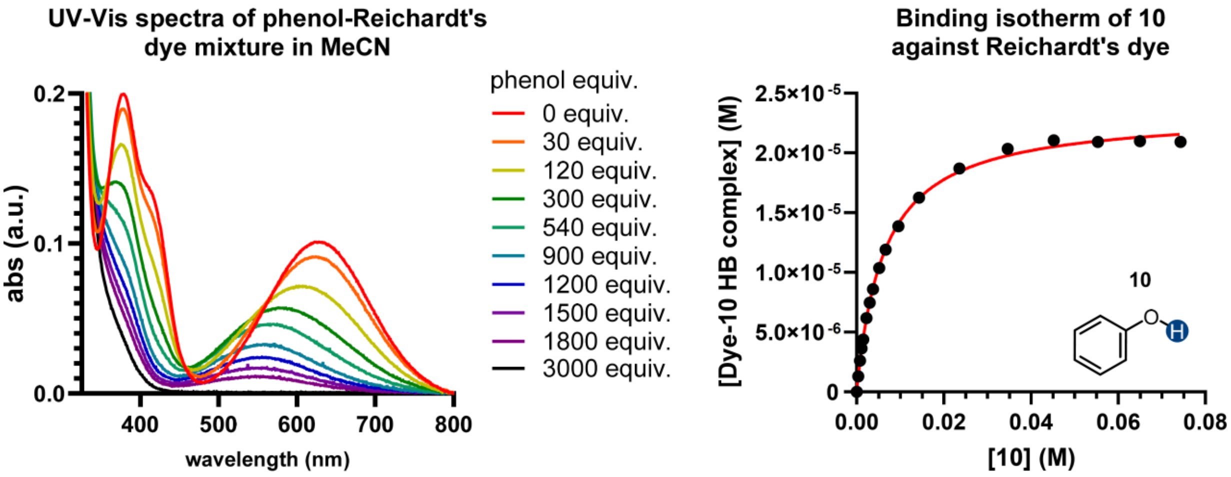

Figure S20. UV–vis titration of Reichardt's dye with phenol (**10**) in MeCN.

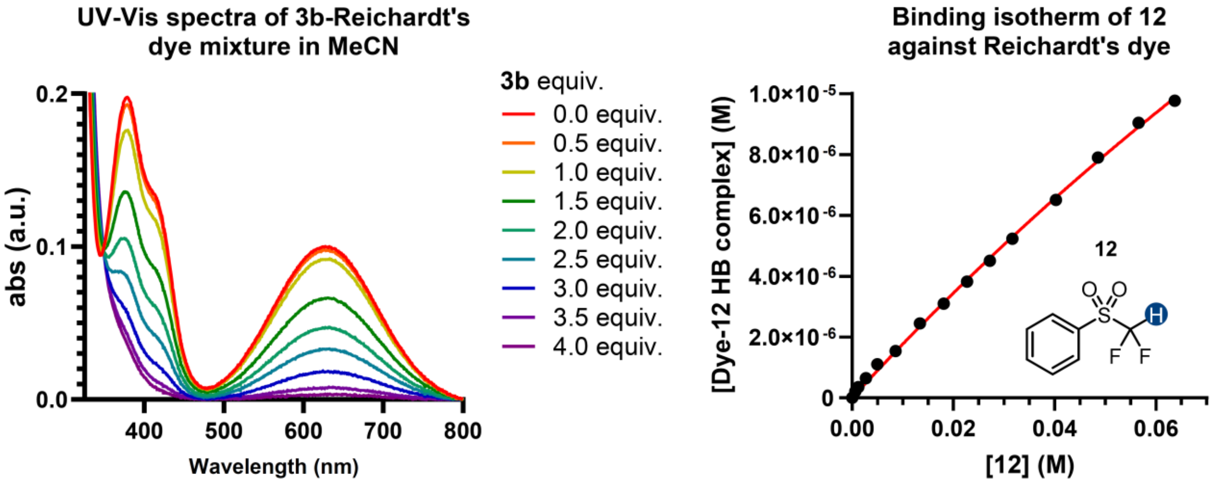

Figure S21. UV–vis titration of Reichardt's dye with difluoromethyl phenyl sulfone (**12**) in MeCN.

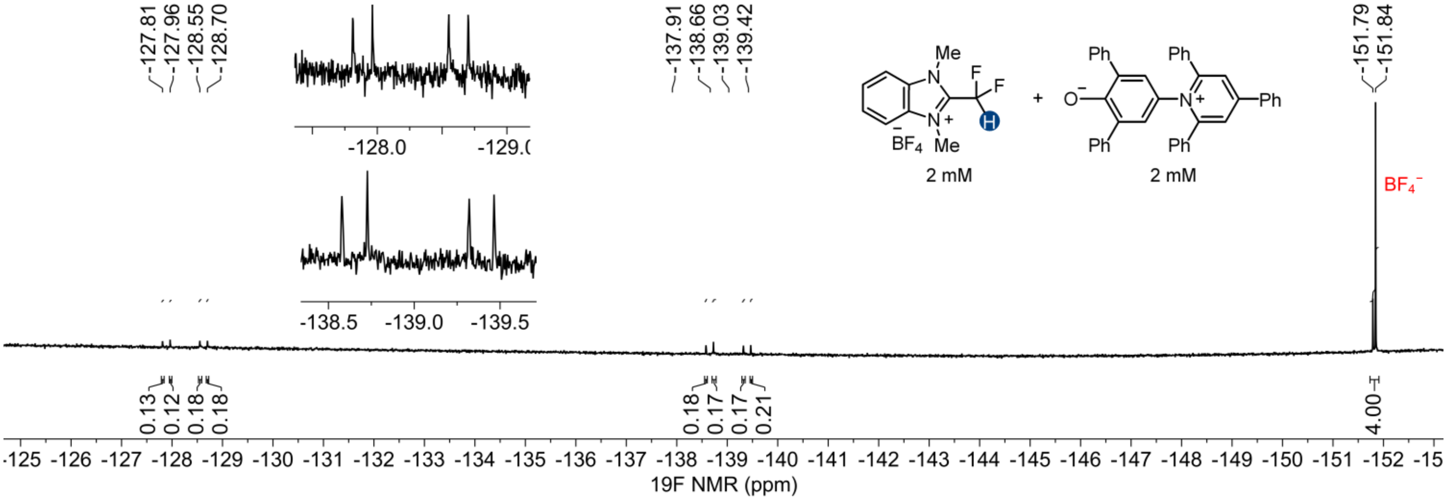

Figure S22. ^{19}F NMR spectrum showing the signals of the CF_2H group of **3b** (2 mM) in the presence of Reichardt's dye (2 mM) in anh. CD_3CN. Multiple sets of signals were observed, indicating the formation of possible covalent adducts.

NMR titration with tri-n-butylphosphine oxide (n-Bu$_3$PO).

All NMR titrations were performed on a Bruker Avance III HD 400 MHz spectrometer. Titration experiments were adapted from an established method.[4] For all experiments, CD_3CN was dried over 3 Å molecular sieves in the glovebox for at least 24 h. For each titration, a solution of the hydrogen bond donating compound in anhydrous CD_3CN (5 mM) was prepared in the glovebox. A portion of this solution (450 μL) was added to an NMR tube with a septum. A portion of *n*-Bu$_3$PO (200–300 mg) was dissolved in anhydrous CD_3CN containing the hydrogen bond donating compound (5 mM) in the glovebox to obtain a solution at a concentration of 2–3 M. This solution was added by increments to the hydrogen bond donating compound solution in the NMR tube with a microsyringe through the septum. NMR samples with *n*-Bu$_3$PO at high concentrations (>2.5 M) were prepared separately by directly adding a portion of pure HB donating compound to a stock solution of *n*-Bu$_3$PO in CD_3CN at the indicated concentrations. All ^{1}H NMR spectra were acquired after each addition with 16 scans. Changes in ^{1}H NMR chemical shift of the CF_2H group were plotted against *n*-Bu$_3$PO concentration and fitted in using the one-site specific binding model for saturation binding.

$$\Delta\delta = \frac{B_{max}[n\text{-Bu}_3\text{PO}]}{K_\text{d} + [n\text{-Bu}_3\text{PO}]}$$

Table 2. Summary of ^{1}H NMR titration results.

Compound	K_d (mM)	B_{max}	R^2
**1a** (CF_2H)	8.1×10^1	1.4×10^0 [a]	0.999
**1b** (CF_2H)	2.6×10^0	1.4×10^0	1.000
**1b** (*ortho*-H)	4.1×10^0	2.2×10^0	1.000
**2a** (CF_2H)	4.0×10^1	8.9×10^{-1}	1.000
**2b** (CF_2H)	2.1×10^0	1.2×10^0	1.000
**3a** (CF_2H)	2.0×10^1	1.4×10^0 [a]	0.994
**3b** (CF_2H)	1.5×10^0	1.3×10^0	1.000
**4a** (CF_2H)	1.9×10^1	1.4×10^0 [a]	0.991
**4b** (CF_2H)	9.1×10^1	1.4×10^0 [a]	0.997
**4b** (*ortho*-H)	6.6×10^0	2.6×10^0	1.000
**5a** (CF_2H)	1.2×10^1	1.4×10^0 [a]	0.996
**5b** (CF_2H)	1.1×10^1	3.2×10^0	1.000
**5b** (*ortho*-H)	2.9×10^0	1.9×10^0	1.000
**6a** (CF_2H)	7.2×10^0	1.4×10^0 [a]	0.951
**7b** (CF_2H)	6.1×10^0	2.4×10^0	1.000
**8b** (CF_2H)	4.0×10^0	1.8×10^0	1.000
**9b** (*ortho*-H)	5.5×10^0	2.6×10^0	1.000
**10** (OH)	4.7×10^{-2}	3.6×10^0	1.000
**12** (CF_2H)	7.9×10^0	3.9×10^0	1.000
**13** (CF_2H)	6.6×10^1	1.4×10^0 [a]	0.990

[a] A constraint of $B_{max} < 1.4\times10^0$ was applied to fit the data. The constraint value was chosen based on the B_{max} (1.3×10^0) of the fit for **1b**.

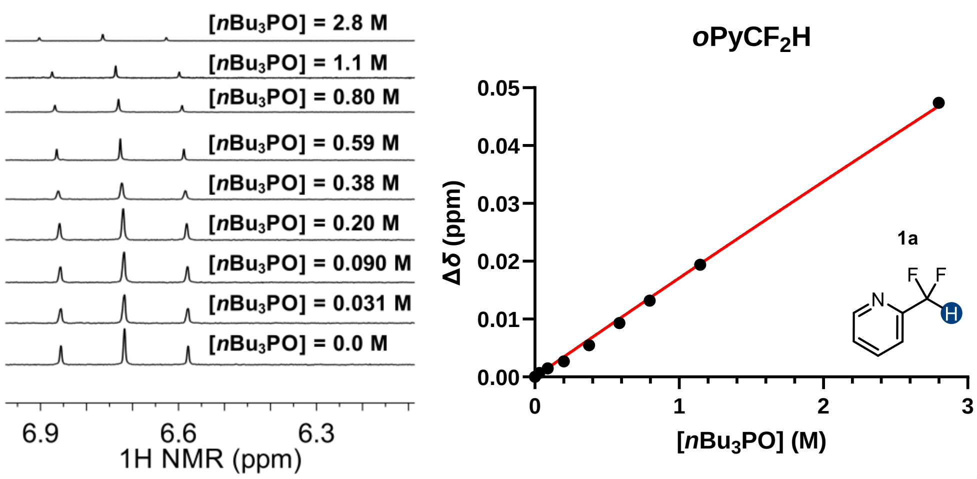

Figure S23. Left panel: ^{1}H NMR spectra showing the signals of the CF_2H group of **1a** in the presence of *n*-Bu_3PO at different concentrations. in anh. CD_3CN. Right panel: Determination of dissociation constant (K_d) of the *n*-$Bu_3PO\cdots$**1a** HB complex by fitting the data to a single-site binding model.

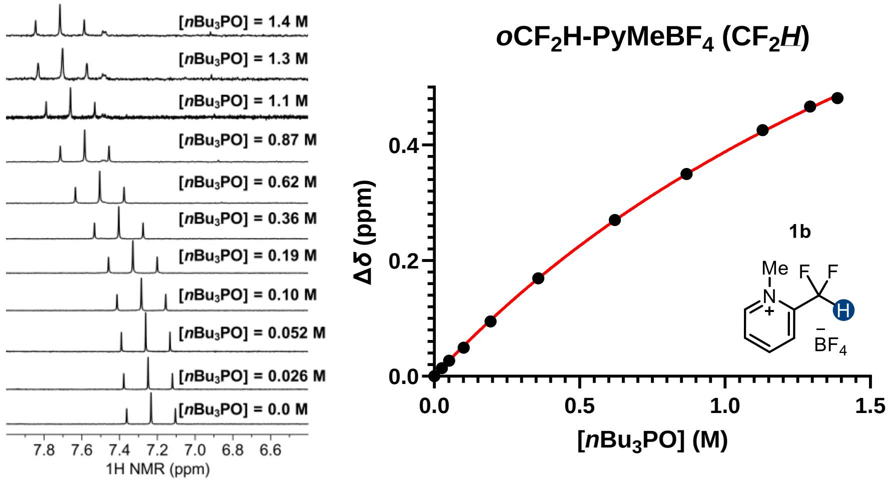

Figure S24. Left panel: ^{1}H NMR spectra showing the signals of the CF_2H group of **1b** in the presence of *n*-Bu_3PO at different concentrations. in anh. CD_3CN. Right panel: Determination of dissociation constant (K_d) of the *n*-$Bu_3PO\cdots$**1b** HB complex by fitting the data to a single-site binding model.

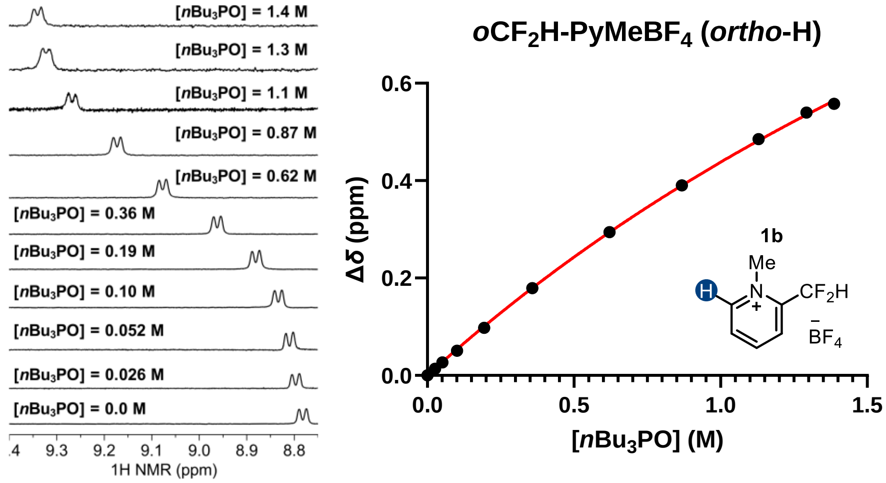

Figure S25. Left panel: ^{1}H NMR spectra showing the signals of the *ortho* hydrogen of **1b** in the presence of *n*-Bu_3PO at different concentrations. in anh. CD_3CN. Right panel: Determination of dissociation constant (K_d) of the *n*-$Bu_3PO\cdots$**1b** HB complex by fitting the data to a single-site binding model.

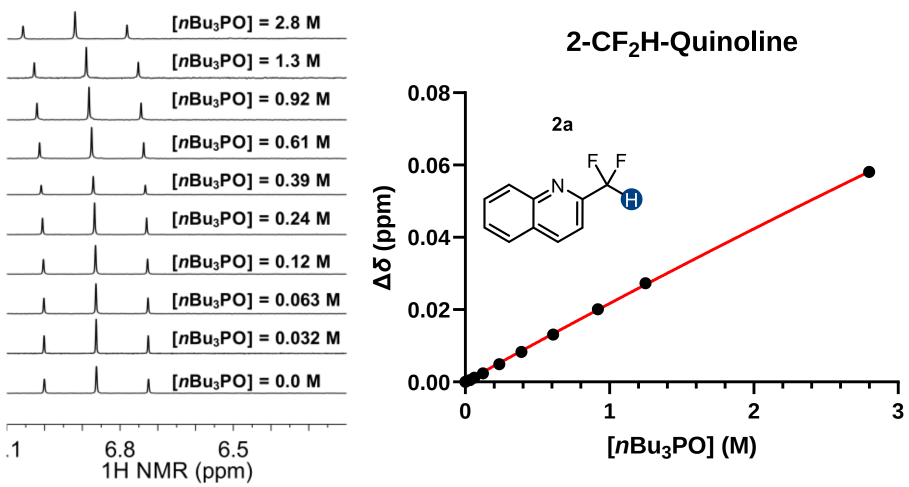

Figure S26. Left panel: ^{1}H NMR spectra showing the signals of the CF_2H group of **2a** in the presence of *n*-Bu_3PO at different concentrations. in anh. CD_3CN. Right panel: Determination of dissociation constant (K_d) of the *n*-Bu_3PO···**2a** HB complex by fitting the data to a single-site binding model.

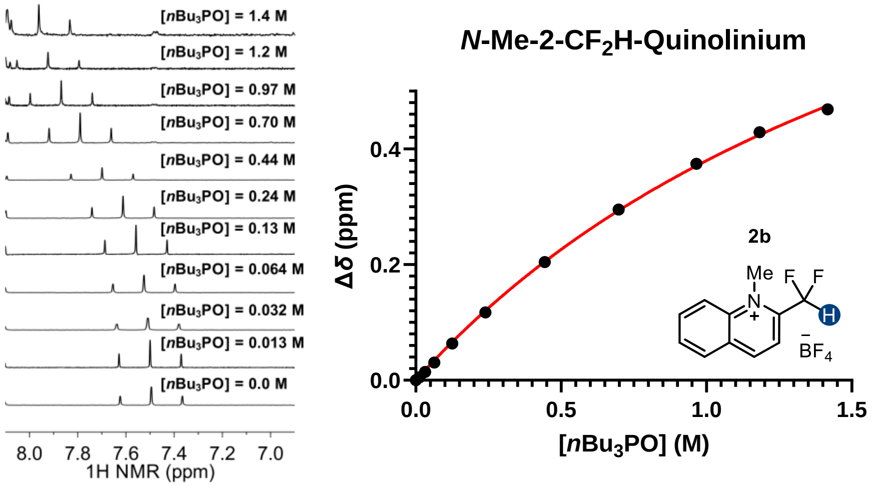

Figure S27. Left panel: ^{1}H NMR spectra showing the signals of the CF_2H group of **2b** in the presence of *n*-Bu_3PO at different concentrations. in anh. CD_3CN. Right panel: Determination of dissociation constant (K_d) of the *n*-Bu_3PO···**2b** HB complex by fitting the data to a single-site binding model.

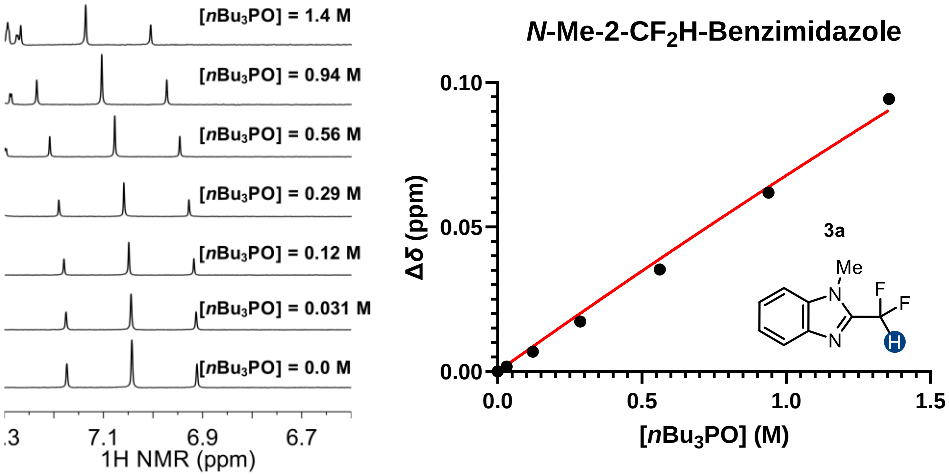

Figure S28. Left panel: ^{1}H NMR spectra showing the signals of the CF_2H group of **3a** in the presence of *n*-Bu_3PO at different concentrations. in anh. CD_3CN. Right panel: Determination of dissociation constant (K_d) of the *n*-Bu_3PO···**3a** HB complex by fitting the data to a single-site binding model.

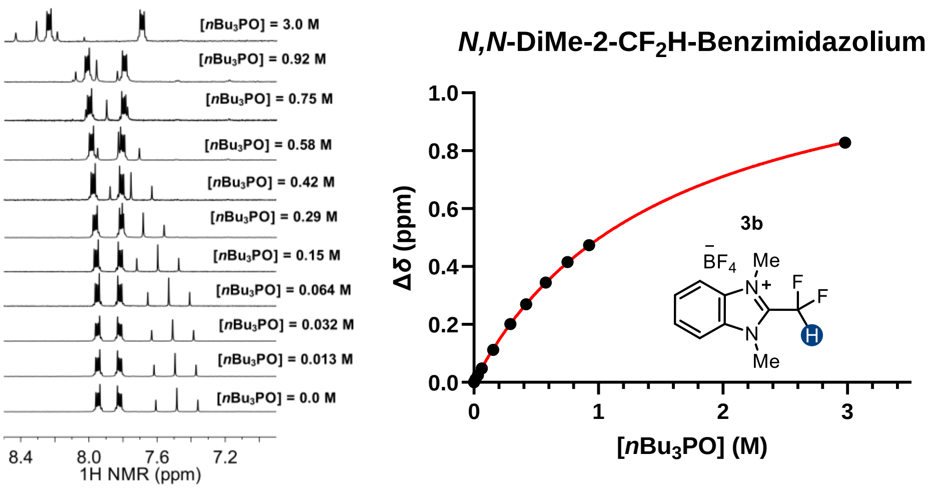

Figure S29. Left panel: ^{1}H NMR spectra showing the signals of the CF_2H group of **3b** in the presence of *n*-Bu_3PO at different concentrations. in anh. CD_3CN. Right panel: Determination of dissociation constant (K_d) of the *n*-$Bu_3PO\cdots$**3b** HB complex by fitting the data to a single-site binding model.

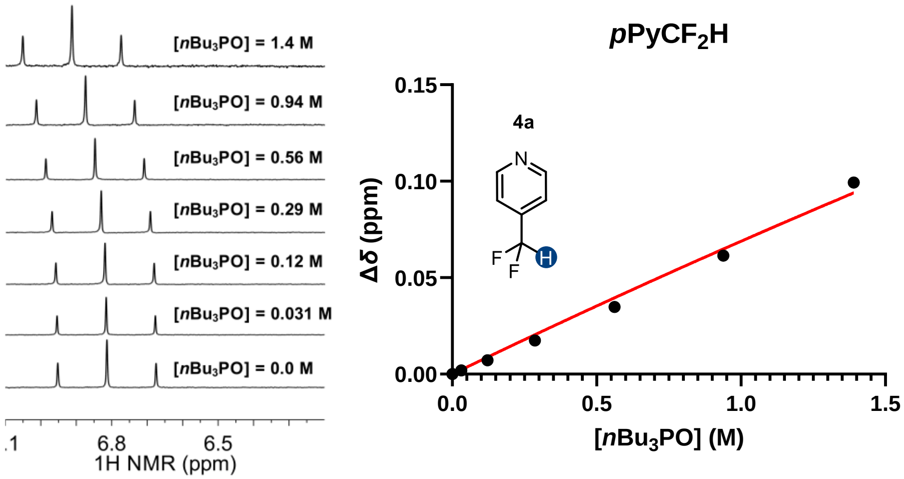

Figure S30. Left panel: ^{1}H NMR spectra showing the signals of the CF_2H group of **4a** in the presence of *n*-Bu_3PO at different concentrations. in anh. CD_3CN. Right panel: Determination of dissociation constant (K_d) of the *n*-$Bu_3PO\cdots$**4a** HB complex by fitting the data to a single-site binding model.

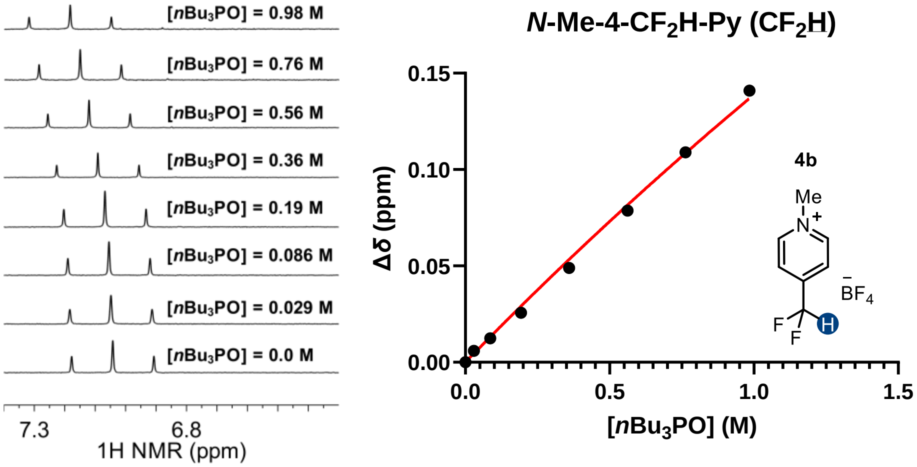

Figure S31. Left panel: ^{1}H NMR spectra showing the signals of the CF_2H group of **4b** in the presence of *n*-Bu_3PO at different concentrations. in anh. CD_3CN. Right panel: Determination of dissociation constant (K_d) of the *n*-$Bu_3PO\cdots$**4b** HB complex by fitting the data to a single-site binding model.

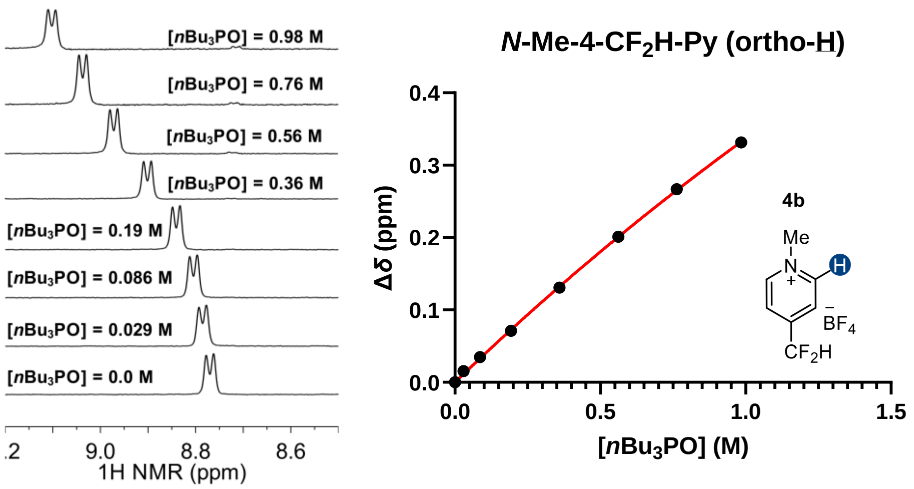

Figure S32. Left panel: ^{1}H NMR spectra showing the signals of the *ortho* hydrogen of **4b** in the presence of *n*-Bu_3PO at different concentrations. in anh. CD_3CN. Right panel: Determination of dissociation constant (K_d) of the *n*-$Bu_3PO\cdots$**4b** HB complex by fitting the data to a single-site binding model.

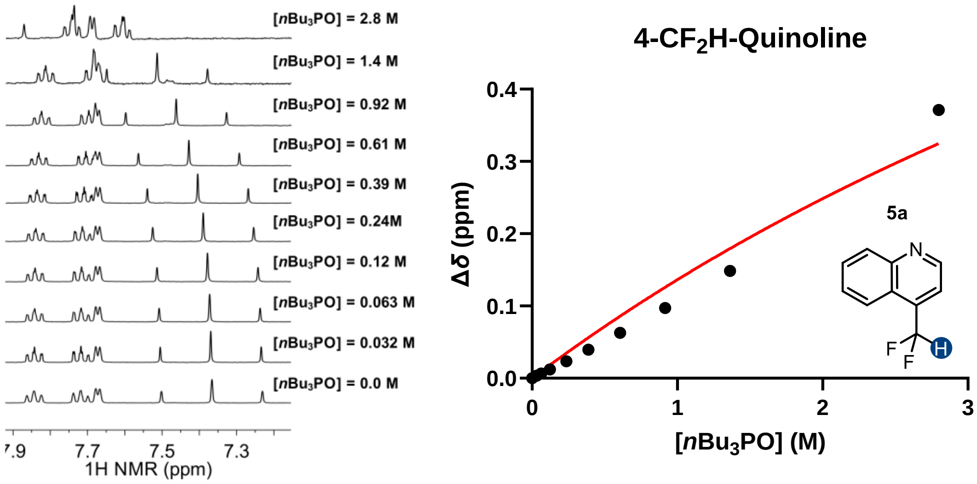

Figure S33. Left panel: ^{1}H NMR spectra showing the signals of the *ortho* hydrogen of **5a** in the presence of *n*-Bu_3PO at different concentrations. in anh. CD_3CN. Right panel: Determination of dissociation constant (K_d) of the *n*-$Bu_3PO\cdots$**5a** HB complex by fitting the data to a single-site binding model.

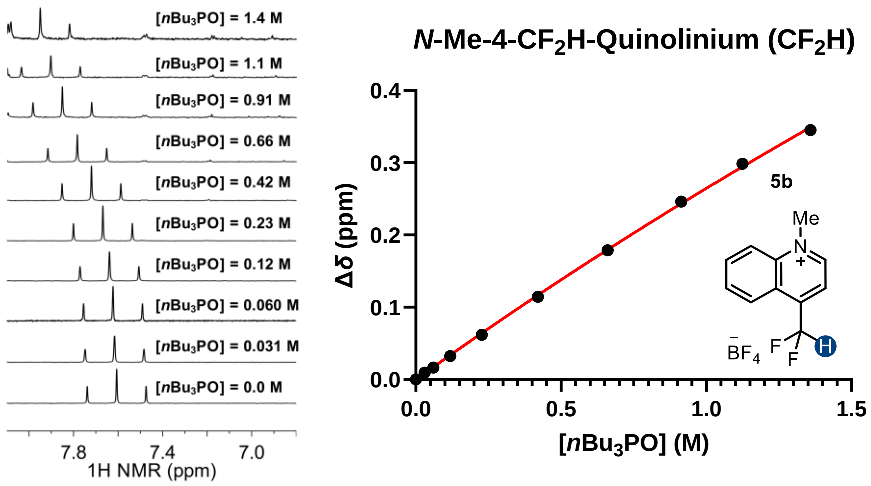

Figure S34. Left panel: ^{1}H NMR spectra showing the signals of the CF_2H group of **5b** in the presence of *n*-Bu_3PO at different concentrations. in anh. CD_3CN. Right panel: Determination of dissociation constant (K_d) of the *n*-$Bu_3PO\cdots$**5b** HB complex by fitting the data to a single-site binding model.

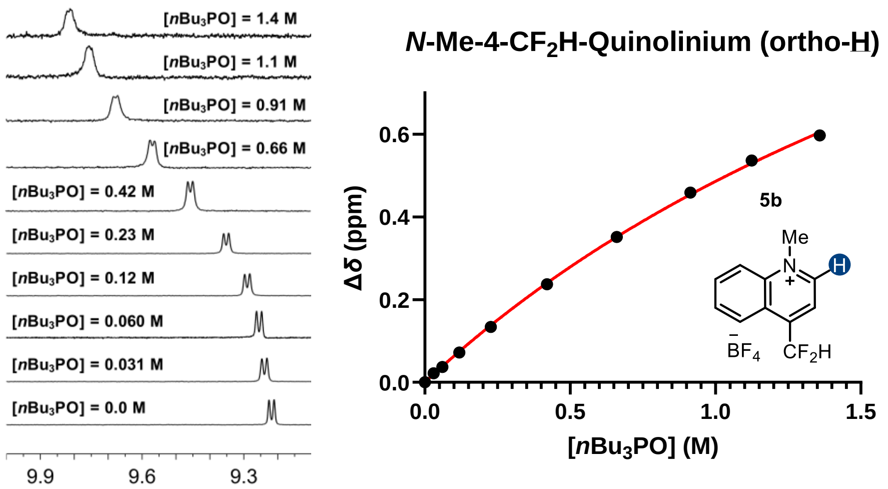

Figure S35. Left panel: ^{1}H NMR spectra showing the signals of the *ortho* hydrogen of **5b** in the presence of *n*-Bu_3PO at different concentrations. in anh. CD_3CN. Right panel: Determination of dissociation constant (K_d) of the *n*-Bu_3PO···**5b** HB complex by fitting the data to a single-site binding model.

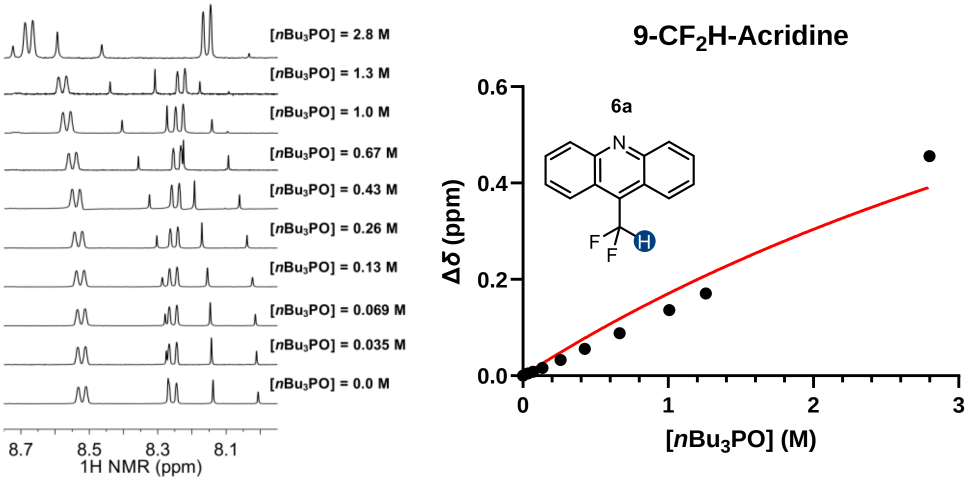

Figure S36. Left panel: ^{1}H NMR spectra showing the signals of the CF_2H group of **6a** in the presence of *n*-Bu_3PO at different concentrations. in anh. CD_3CN. Right panel: Determination of dissociation constant (K_d) of the *n*-Bu_3PO···**6a** HB complex by fitting the data to a single-site binding model.

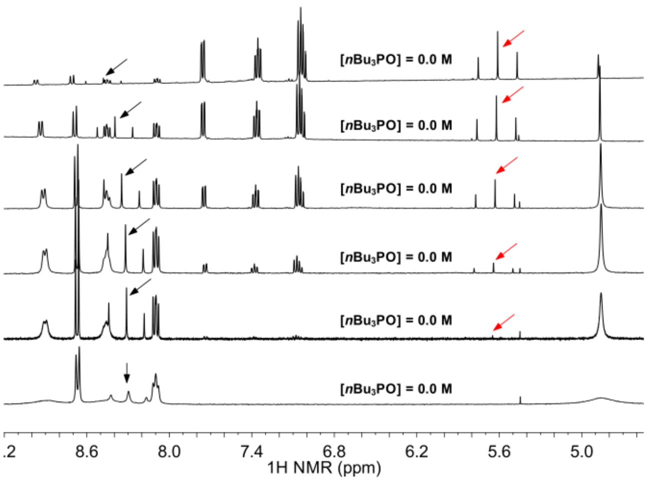

Figure S37. ^{1}H NMR spectra showing the signals of the CF_2H group of **6b** in the presence of *n*-Bu_3PO at different concentrations. in anh. CD_3CN. As indicated by the triplet at 5.61 ppm, a new species formed as *n*-Bu_3PO was added, suggesting potential covalent interactions between **6b** and *n*-Bu_3PO.

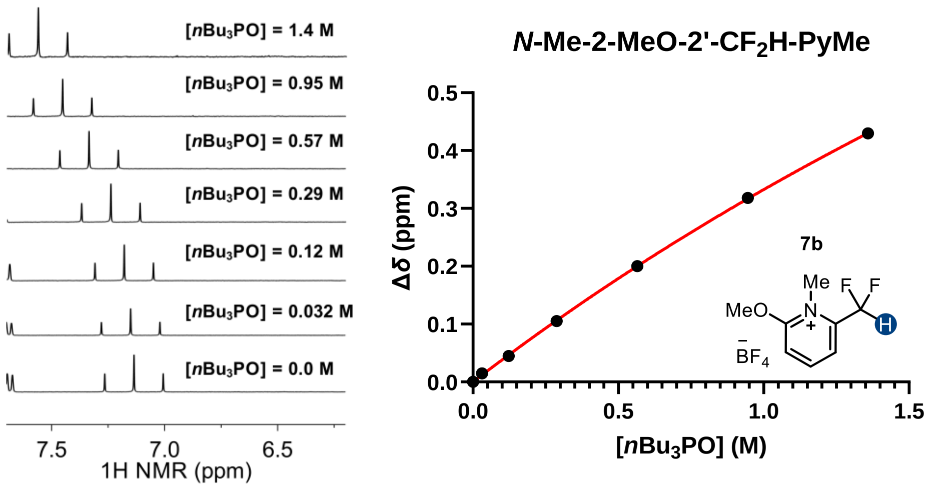

Figure S38. Left panel: ^{1}H NMR spectra showing the signals of the CF_2H group of **7b** in the presence of *n*-Bu_3PO at different concentrations. in anh. CD_3CN. Right panel: Determination of dissociation constant (K_d) of the *n*-Bu_3PO···**7b** HB complex by fitting the data to a single-site binding model.

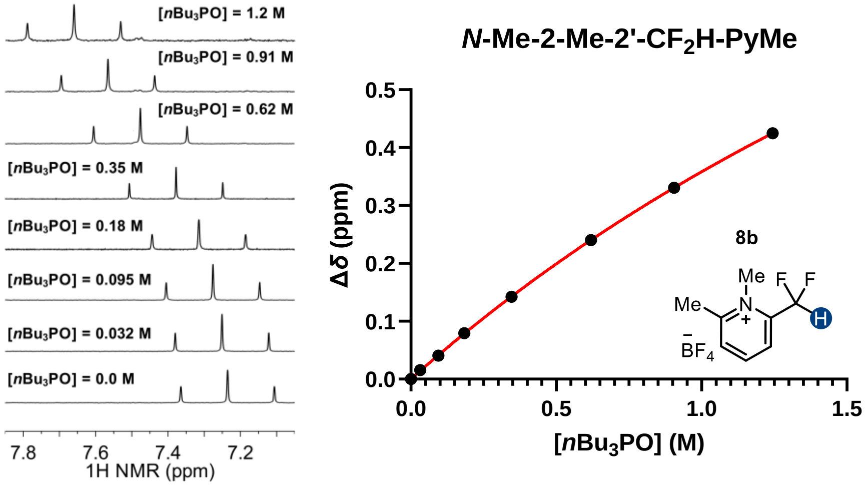

Figure S39. Left panel: ^{1}H NMR spectra showing the signals of the CF_2H group of **8b** in the presence of *n*-Bu_3PO at different concentrations. in anh. CD_3CN. Right panel: Determination of dissociation constant (K_d) of the *n*-Bu_3PO···**8b** HB complex by fitting the data to a single-site binding model.

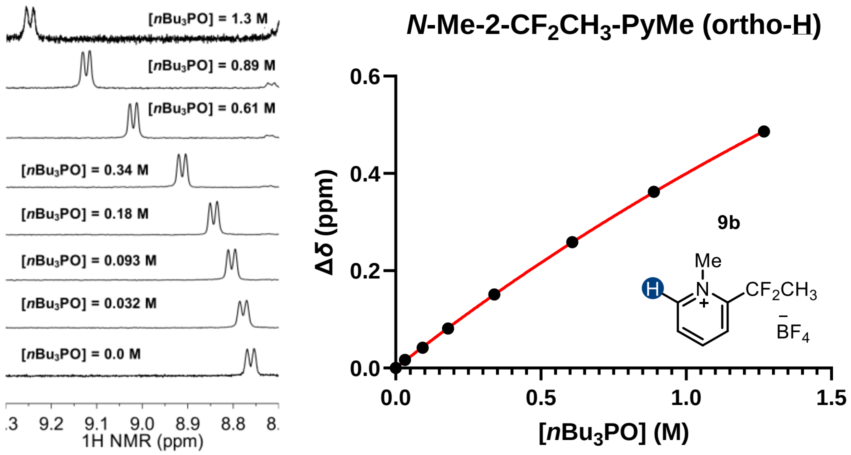

Figure S40. Left panel: ^{1}H NMR spectra showing the signals of the CF_2H group of **9b** in the presence of *n*-Bu_3PO at different concentrations. in anh. CD_3CN. Right panel: Determination of dissociation constant (K_d) of the *n*-Bu_3PO···**9b** HB complex by fitting the data to a single-site binding model.

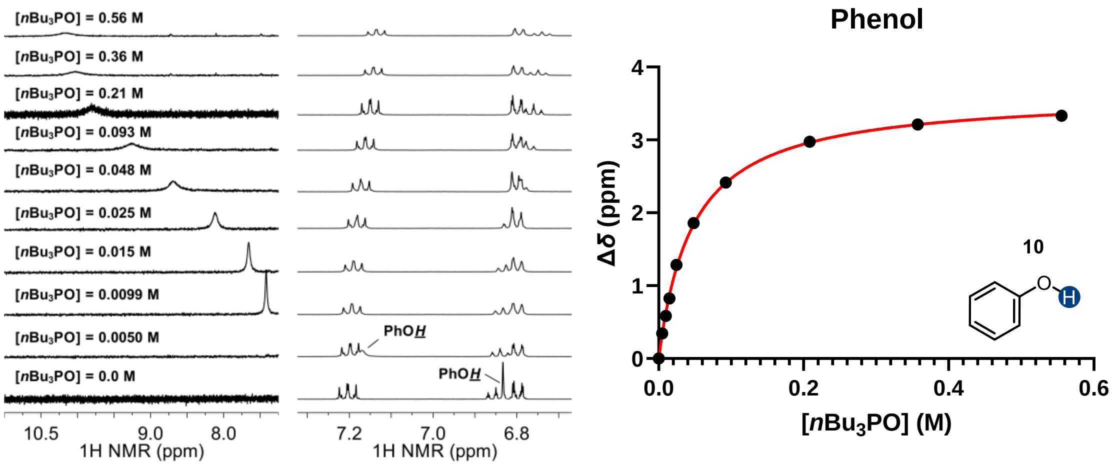

Figure S41. Left panel: ^{1}H NMR spectra showing the signals of the CF_2H group of **10** in the presence of *n*-Bu_3PO at different concentrations. in anh. CD_3CN. Right panel: Determination of dissociation constant (K_d) of the *n*-Bu_3PO···**10** HB complex by fitting the data to a single-site binding model.

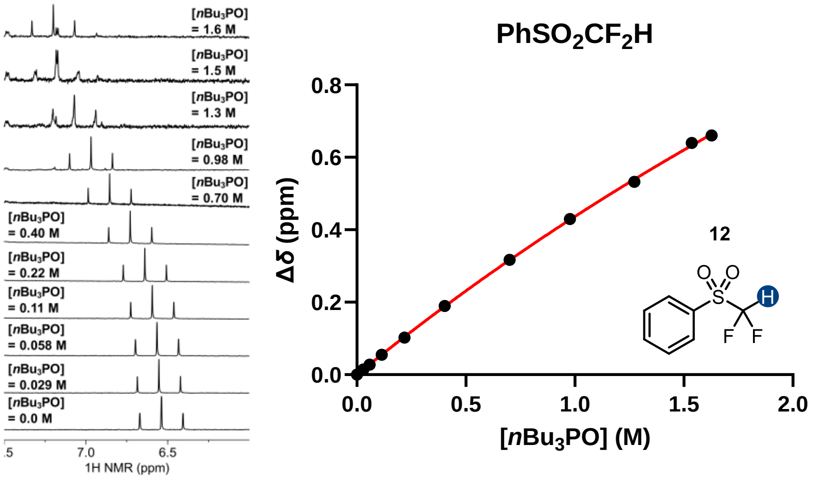

Figure S42. Left panel: ^{1}H NMR spectra showing the signals of the CF_2H group of **12** in the presence of *n*-Bu_3PO at different concentrations. in anh. CD_3CN. Right panel: Determination of dissociation constant (K_d) of the *n*-Bu_3PO···**12** HB complex by fitting the data to a single-site binding model.

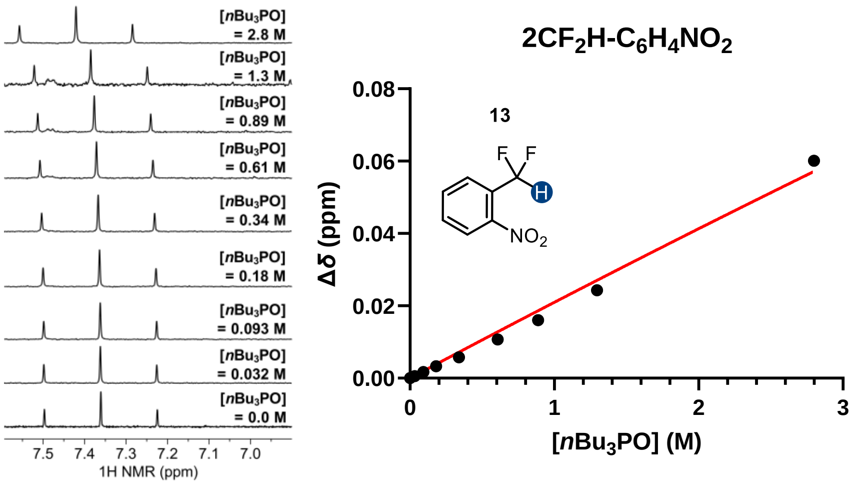

Figure S43. Left panel: ^{1}H NMR spectra showing the signals of the CF_2H group of **13** in the presence of *n*-Bu_3PO at different concentrations. in anh. CD_3CN. Right panel: Determination of dissociation constant (K_d) of the *n*-Bu_3PO···**13** HB complex by fitting the data to a single-site binding model.

Theoretical calculations.

Geometry optimization was performed at the M06-2X/6-31+G(d,p) level in acetonitrile using Gaussian 16.[5-6] The hybrid meta exchange-correlation density functional M06-2X empirically accounts for dispersive interactions and demonstrates high accuracy in main-group thermochemistry.[5] To account for the solvent effect of acetonitrile, the polarizable continuum model was applied using the integral equation formalism variant (IEFPCM).[7-8] All stationary points were characterized by frequency calculations at the same level, which confirms the local minimum. The relative populations of the different conformers at 298 K were estimated using Boltzmann statistics. The natural atomic charges were calculated using Natural Bond Orbital (NBO) analysis program version 3.[9]

To assess the impacts of basis sets on the computational results, single point energies were calculated at the PCM-M06-2X/6-311++G(2d,2p)//M06-2X/6-31+G(d,p) level of theory in acetonitrile. The free energies were calculated by combining single point PCM-M06-2X/6-311++G(2d,2p)//M06-2X/6-31+G(d,p) calculations with thermal and entropic corrections obtained at the PCM-M06-2X/6-31+G(d,p) level. As shown in Fig. S47, ΔG_{calc} values obtained at the PCM-M06-2X/6-311++G(2d,2p)//M06-2X/6-31+G(d,p) and the PCM-M06-2X/6-31+G(d,p) level show nearly identical accurary. Given the higher computational efficiency and satisfactory reliability of the PCM-M06-2X/6-31+G(d,p) calculations, we use the ΔG_{calc} values from these calculations for further discussions.

Table S3. Energy and population of **1a** and its HB complexes with Me_3PO.

PCM(MeCN)-M06-2X/6-31+G(d,p)								NBO analysis	
Conformer /species	*E* (hartree)	Δ*E* (kcal /mol)	Free Energy (hartree)	Δ*G* (kcal/ mol)[a]	Pop[b]	$Pop_{unbound}$ and $Pop_{complex}$[c]	ΔG_{total} (kcal/mol)[d]	Me_3PO→H–X bond interaction energy (kcal/mol)[e]	NBO charge on the hydrogen of of H–X bond
Me_3PO	-536.241732	-	-536.154069	-	-	-	-	-	-
**1a**-conformer A	-485.921542	-	-485.851756	-	-	-	-	-	0.224
**1a**-conformer B	-485.920701	-	-485.850361	-	-	-	-	-	0.219
Me_3PO + **1a**-conformer A	-1022.163274	-0.5	-1022.0058	0.0	1.0×10^{2}	1.0	0.0	-	-
Me_3PO + **1a**-conformer B	-1022.162433	0.0	-1021.99492	0.9	2.2×10^{1}			-	-
Me_3PO•••**1a**-conformer A HB complex	-1022.171326	-5.6	-1022.004400	6.8	9.7×10^{-4}	1.9×10^{-5}	6.4	3.7	-
Me_3PO•••**1a**-conformer B HB complex	-1022.168276	-3.7	-1021.995294	6.6	1.4×10^{-4}			6.5	-

[a] Gibbs free energy relative to the most stable species. [b] relative population calculated based on Δ*G* at 298 K. The population of the most stable species is set as 1.0×10^{2}. [c] relative population calculated as a fraction of the population of all four species (Σ*Pop*). [d] ΔG_{total} values are calculated based on $Pop_{unbound}$ and $Pop_{complex}$ at 298 K. [e] Calculated as the sum of the interaction energy of the lone pairs of the oxygen of Me_3PO with the σ* bond of the H–X moiety.

Table S4. Energy and population of **1b** and its HB complexes with Me_3PO.

PCM(MeCN)-M06-2X/6-31+G(d,p)								NBO analysis	
Conformer /species	*E* (hartree)	Δ*E* (kcal /mol)	Free Energy (hartree)	Δ*G* (kcal/ mol)[a]	Pop[b]	$Pop_{unbound}$ and $Pop_{complex}$[c]	ΔG_{total} (kcal/mol)[d]	Me_3PO→H–X bond interaction energy (kcal/mol)[e]	NBO charge on the hydrogen of of H–X bond
Me_3PO	-536.241732	-	-536.154069	-	-	-	-	-	-
**1b**-conformer A	-525.6394394	-	-525.528104	-	-	-	-	-	0.246
**1b**-conformer B	-525.6393416	-	-525.52775	-	-	-	-	-	0.249
Me_3PO + **1b**-conformer A	-1061.881172	-0.1	-1061.6822	0.0	1.0×10^{2}	1.0	0.0	-	-
Me_3PO + **1b**-conformer B	-1061.881074	0.0	-1061.6818	0.2	8.3×10^{1}			-	-
Me_3PO•••**1b**-conformer A HB complex	-1061.895577	-9.1	-1061.677472	3.0	6.9×10^{-1}	4.4×10^{-3}	3.2	2.7	-
Me_3PO•••**1b**-conformer B HB complex	-1061.891056	-6.3	-1061.675848	4.0	1.2×10^{-1}			8.5	-

[a] Gibbs free energy relative to the most stable species. [b] relative population calculated based on Δ*G* at 298 K. The population of the most stable species is set as 1.0×10^{2}. [c] relative population calculated as a fraction of the population of all four species (Σ*Pop*). [d] ΔG_{total} values are calculated based on $Pop_{unbound}$ and $Pop_{complex}$ at 298 K. [e] Calculated as the sum of the interaction energy of the lone pairs of the oxygen of Me_3PO with the σ* bond of the H–X moiety.

Table S5. Energy and population of **2a** and its HB complexes with Me_3PO.

PCM(MeCN)-M06-2X/6-31+G(d,p)								NBO analysis	
Conformer /species	*E* (hartree)	Δ*E* (kcal /mol)	Free Energy (hartree)	Δ*G* (kcal/ mol)[a]	Pop[b]	$Pop_{unbound}$ and $Pop_{complex}$[c]	ΔG_{total} (kcal/mol)[d]	Me_3PO→H–X bond interaction energy (kcal/mol)[e]	NBO charge on the hydrogen of of H–X bond
Me_3PO	-536.241732	-	-536.154069	-	-	-	-	-	-
**2a**-conformer A	-639.5123974	-	-639.399119	-	-	-	-	-	0.225
**2a**-conformer B	-639.5103922	-	-639.397745	-	-	-	-	-	0.219
Me_3PO + **2a**-conformer A	-1175.75413	-1.3	-1175.5532	0.0	1.0×10^{2}	1.0	0.0	-	-
Me_3PO + **2a**-conformer B	-1175.752124	0.0	-1175.5518	0.9	2.3×10^{1}			-	-
Me_3PO•••**2a**-conformer A HB complex	-1175.761151	-5.7	-1175.542688	6.6	1.5×10^{-3}	1.5×10^{-5}	6.6	9.4	-
Me_3PO•••**2a**-conformer B HB complex	-1175.75836	-3.9	-1175.541299	7.5	3.4×10^{-5}			5.8	-

[a] Gibbs free energy relative to the most stable species. [b] relative population calculated based on Δ*G* at 298 K. The population of the most stable species is set as 1.0×10^{2}. [c] relative population calculated as a fraction of the population of all four species (Σ*Pop*). [d] ΔG_{total} values are calculated based on $Pop_{unbound}$ and $Pop_{complex}$ at 298 K. [e] Calculated as the sum of the interaction energy of the lone pairs of the oxygen of Me_3PO with the σ* bond of the H–X moiety.

Table S6. Energy and population of **2b** and its HB complexes with Me_3PO.

PCM(MeCN)-M06-2X/6-31+G(d,p)								NBO analysis	
Conformer /species	E (hartree)	ΔE (kcal /mol)	Free Energy (hartree)	ΔG (kcal/ mol)[a]	Pop[b]	$Pop_{unbound}$ and $Pop_{complex}$[c]	ΔG_{total} (kcal/mol)[d]	Me_3PO→H–X bond interaction energy (kcal/mol)[e]	NBO charge on the hydrogen of of H–X bond
Me_3PO	-536.241732	-	-536.154069	-	-	-	-	-	-
**2b**-conformer A	-679.22579952	-	-679.070516	-	-	-	-	-	0.248
**2b**-conformer B	-679.22570530	-	-679.070308	-	-	-	-	-	0.250
Me_3PO + **2b**-conformer A	-1215.467532	-0.1	-1215.2246	0.0	1.0×10^{2}	1.0	0.0	-	-
Me_3PO + **2b**-conformer B	-1215.467438	0.0	-1215.2244	0.1	8.0×10^{1}			-	-
Me_3PO•••**2b**-conformer A HB complex	-1215.478089	-6.7	-1215.21771	4.3	6.9×10^{-2}	1.5×10^{-3}	3.8	10.5	-
Me_3PO•••**2b**-conformer B HB complex	-1215.477399	-6.3	-1215.218738	3.7	2.0×10^{-1}			9.1	-

[a] Gibbs free energy relative to the most stable species. [b] relative population calculated based on ΔG at 298 K. The population of the most stable species is set as 1.0×10^{2}. [c] relative population calculated as a fraction of the population of all four species (ΣPop). [d] ΔG_{total} values are calculated based on $Pop_{unbound}$ and $Pop_{complex}$ at 298 K. [e] Calculated as the sum of the interaction energy of the lone pairs of the oxygen of Me_3PO with the σ* bond of the H–X moiety.

Table S7. Energy and population of **3a** and its HB complexes with Me_3PO.

PCM(MeCN)-M06-2X/6-31+G(d,p)								NBO analysis	
Conformer /species	E (hartree)	ΔE (kcal /mol)	Free Energy (hartree)	ΔG (kcal/ mol)[a]	Pop[b]	$Pop_{unbound}$ and $Pop_{complex}$[c]	ΔG_{total} (kcal/mol)[d]	Me_3PO→H–X bond interaction energy (kcal/mol)[e]	NBO charge on the hydrogen of of H–X bond
Me_3PO	-536.241732	-	-536.154069	-	-	-	-	-	-
**3a**-conformer A	-656.75640271	-	-656.632652	-	-	-	-	-	0.231
**3a**-conformer B	-656.75885902	-	-656.634848	-	-	-	-	-	0.240
Me_3PO + **3a**-conformer A	-1192.998135	0.0	-1192.786721	1.4	9.8×10^{0}	1.0	0.0	-	-
Me_3PO + **3a**-conformer B	-1193.000591	-1.5	-1192.788917	0.0	1.0×10^{2}			-	-
Me_3PO•••**3a**-conformer A HB complex	-1193.0047187	-4.1	-1192.780474	5.3	4.1×10^{-3}	1.3×10^{-4}	5.3	6.2	-
Me_3PO•••**3a**-conformer B HB complex	-1193.0076084	-5.9	-1192.777917	6.9	2.7×10^{-4}			2.8	-

[a] Gibbs free energy relative to the most stable species. [b] relative population calculated based on ΔG at 298 K. The population of the most stable species is set as 1.0×10^{2}. [c] relative population calculated as a fraction of the population of all four species (ΣPop). [d] ΔG_{total} values are calculated based on $Pop_{unbound}$ and $Pop_{complex}$ at 298 K. [e] Calculated as the sum of the interaction energy of the lone pairs of the oxygen of Me_3PO with the σ* bond of the H–X moiety.

Table S8. Energy and population of **3b** and its HB complexes with Me_3PO.

PCM(MeCN)-M06-2X/6-31+G(d,p)								NBO analysis	
Conformer /species	E (hartree)	ΔE (kcal /mol)	Free Energy (hartree)	ΔG (kcal/ mol)[a]	Pop[b]	$Pop_{unbound}$ and $Pop_{complex}$[c]	ΔG_{total} (kcal/mol)[d]	Me_3PO→H–X bond interaction energy (kcal/mol)[e]	NBO charge on the hydrogen of of H–X bond
Me_3PO	-536.241732	-	-536.154069	-	-	-	-	-	-
**3b**-conformer A	-696.4809107	-	-696.317449	-	-	-	-	-	0.253
**3b**-conformer B	-696.47967382	-	-696.315167	-	-	-	-	-	0.258
Me_3PO + **3b**-conformer A	-1232.722643	-0.8	-1232.4715	0.0	1.0×10^{2}	1.0	0.0	-	-
Me_3PO + **3b**-conformer B	-1232.721406	0.0	-1232.4692	1.4	8.9×10^{0}			-	-
Me_3PO•••**3b**-conformer A HB complex	-1232.7349303	-8.5	-1232.465414	3.8	1.6×10^{-1}	1.4×10^{-2}	2.5	8.3	-
Me_3PO•••**3b**-conformer B HB complex	-1232.737985	-10.4	-1232.46746	2.6	1.4×10^{0}			1.7	-

[a] Gibbs free energy relative to the most stable species. [b] relative population calculated based on ΔG at 298 K. The population of the most stable species is set as 1.0×10^{2}. [c] relative population calculated as a fraction of the population of all four species (ΣPop). [d] ΔG_{total} values are calculated based on $Pop_{unbound}$ and $Pop_{complex}$ at 298 K. [e] Calculated as the sum of the interaction energy of the lone pairs of the oxygen of Me_3PO with the σ* bond of the H–X moiety.

Table S9. Energy and population of **4a** and its HB complexes with Me_3PO.

PCM(MeCN)-M06-2X/6-31+G(d,p)								NBO analysis	
Conformer /species	E (hartree)	ΔE (kcal /mol)	Free Energy (hartree)	ΔG (kcal/ mol)[a]	Pop[b]	$Pop_{unbound}$ and $Pop_{complex}$[c]	ΔG_{total} (kcal/mol)[d]	$Me_3PO \rightarrow H–X$ bond interaction energy (kcal/mol)[e]	NBO charge on the hydrogen of of H–X bond
Me_3PO	-536.241732	-	-536.154069	-	-	-	-	-	-
**4a**-conformer A	-485.91884425	-	-485.848801	-	-	-	-	-	0.224
**4a**-conformer B	-485.91910202	-	-485.849056	-	-	-	-	-	0.230
Me_3PO + **4a**-conformer A	-1022.160576	0.0	-1022.0029	0.2	7.6×10^{1}	1.0	0.0	-	-
Me_3PO + **4a**-conformer B	-1022.160834	-0.2	-1022.0031	0.0	1.0×10^{2}			-	-
Me_3PO•••**4a**-conformer A HB complex	-1022.1685144	-5.0	-1021.993544	6.0	3.4×10^{-3}	3.7×10^{-5}	6.0	4.1	-
Me_3PO•••**4a**-conformer B HB complex	-1022.1692436	-5.3	-1021.993152	6.3	2.3×10^{-3}			4.5	-

[a] Gibbs free energy relative to the most stable species. [b] relative population calculated based on ΔG at 298 K. The population of the most stable species is set as 1.0×10^{2}. [c] relative population calculated as a fraction of the population of all four species (ΣPop). [d] ΔG_{total} values are calculated based on $Pop_{unbound}$ and $Pop_{complex}$ at 298 K. [e] Calculated as the sum of the interaction energy of the lone pairs of the oxygen of Me_3PO with the σ* bond of the H–X moiety.

Table S10. Energy and population of **4b** and its HB complexes with Me_3PO.

PCM(MeCN)-M06-2X/6-31+G(d,p)								NBO analysis	
Conformer /species	E (hartree)	ΔE (kcal /mol)	Free Energy (hartree)	ΔG (kcal/ mol)[a]	Pop[b]	$Pop_{unbound}$ and $Pop_{complex}$[c]	ΔG_{total} (kcal/mol)[d]	$Me_3PO \rightarrow H–X$ bond interaction energy (kcal/mol)[e]	NBO charge on the hydrogen of of H–X bond
Me_3PO	-536.241732	-	-536.154069	-	-	-	-	-	-
**4b**-conformer A	-525.64518897	-	-525.535781	-	-	-	-	-	0.235
**4b**-conformer B	-525.64627504	-	-525.53685	-	-	-	-	-	0.244
Me_3PO + **4b**-conformer A	-1061.886921	0.0	-1061.6899	0.7	3.2×10^{1}	1.0	0.0	-	-
Me_3PO + **4b**-conformer B	-1061.888007	-0.7	-1061.6909	0.0	1.0×10^{2}			-	-
Me_3PO•••**4b**-conformer A HB complex	-1061.895943	-5.7	-1061.682667	5.2	1.6×10^{-2}	1.4×10^{-4}	5.3	7.8	-
Me_3PO•••**4b**-conformer B HB complex	-1061.8970048	-6.3	-1061.680874	6.2	2.4×10^{-3}			2.6	-

[a] Gibbs free energy relative to the most stable species. [b] relative population calculated based on ΔG at 298 K. The population of the most stable species is set as 1.0×10^{2}. [c] relative population calculated as a fraction of the population of all four species (ΣPop). [d] ΔG_{total} values are calculated based on $Pop_{unbound}$ and $Pop_{complex}$ at 298 K. [e] Calculated as the sum of the interaction energy of the lone pairs of the oxygen of Me_3PO with the σ* bond of the H–X moiety.

Table S11. Energy and population of **5a** and its HB complexes with Me_3PO.

PCM(MeCN)-M06-2X/6-31+G(d,p)								NBO analysis	
Conformer /species	E (hartree)	ΔE (kcal /mol)	Free Energy (hartree)	ΔG (kcal/ mol)[a]	Pop[b]	$Pop_{unbound}$ and $Pop_{complex}$[c]	ΔG_{total} (kcal/mol)[d]	$Me_3PO \rightarrow H–X$ bond interaction energy (kcal/mol)[e]	NBO charge on the hydrogen of of H–X bond
Me_3PO	-536.241732	-	-536.154069	-	-	-	-	-	-
**5a**-conformer A	-639.50839573	-	-639.393788	-	-	-	-	-	0.227
**5a**-conformer B	-639.50862216	-	-639.393976	-	-	-	-	-	0.227
Me_3PO + **5a**-conformer A	-1175.750128	0.0	-1175.5479	0.1	8.2×10^{1}	1.0	0.0	-	-
Me_3PO + **5a**-conformer B	-1175.750354	-0.1	-1175.5480	0.0	1.0×10^{2}			-	-
Me_3PO•••**5a**-conformer A HB complex	-1175.7609315	-6.8	-1175.539408	6.0	1.1×10^{-2}	9.7×10^{-5}	5.5	5.5	-
Me_3PO•••**5a**-conformer B HB complex	-1175.7565260	-4.0	-1175.539017	5.7	7.0×10^{-3}			6.9	-

[a] Gibbs free energy relative to the most stable species. [b] relative population calculated based on ΔG at 298 K. The population of the most stable species is set as 1.0×10^{2}. [c] relative population calculated as a fraction of the population of all four species (ΣPop). [d] ΔG_{total} values are calculated based on $Pop_{unbound}$ and $Pop_{complex}$ at 298 K. [e] Calculated as the sum of the interaction energy of the lone pairs of the oxygen of Me_3PO with the σ* bond of the H–X moiety.

Table S12. Energy and population of **5b** and its HB complexes with Me_3PO.

PCM(MeCN)-M06-2X/6-31+G(d,p)								NBO analysis	
Conformer /species	E (hartree)	ΔE (kcal /mol)	Free Energy (hartree)	ΔG (kcal/ mol)[a]	Pop[b]	$Pop_{unbound}$ and $Pop_{complex}$[c]	ΔG_{total} (kcal/mol)[d]	Me_3PO→H–X bond interaction energy (kcal/mol)[e]	NBO charge on the hydrogen of of H–X bond
Me_3PO	-536.241732	-	-536.154069	-	-	-	-	-	-
**5b**-conformer A	-679.23318913	-	-679.07762	-	-	-	-	-	0.237
**5b**-conformer B	-679.23364954	-	-679.078103	-	-	-	-	-	0.241
Me_3PO + **5b**-conformer A	-1215.474921	0.0	-1215.2317	0.3	7.7×10^{1}	1.0	0.0	-	-
Me_3PO + **5b**-conformer B	-1215.475382	-0.3	-1215.2322	0.0	1.0×10^{2}			-	-
Me_3PO•••**5b**-conformer A HB complex	-1215.4843150	-5.9	-1215.225394	4.3	7.6×10^{-2}	4.8×10^{-4}	4.5	8.4	-
Me_3PO•••**5b**-conformer B HB complex	-1215.4846258	-6.1	-1215.223448	5.5	1.0×10^{-2}			8.7	-

[a] Gibbs free energy relative to the most stable species. [b] relative population calculated based on ΔG at 298 K. The population of the most stable species is set as 1.0×10^{2}. [c] relative population calculated as a fraction of the population of all four species (ΣPop). [d] ΔG_{total} values are calculated based on $Pop_{unbound}$ and $Pop_{complex}$ at 298 K. [e] Calculated as the sum of the interaction energy of the lone pairs of the oxygen of Me_3PO with the σ* bond of the H–X moiety.

Table S13. Energy and population of **6a** and its HB complexes with Me_3PO.

PCM(MeCN)-M06-2X/6-31+G(d,p)								NBO analysis	
Conformer /species	E (hartree)	ΔE (kcal /mol)	Free Energy (hartree)	ΔG (kcal/ mol)[a]	Pop[b]	$Pop_{unbound}$ and $Pop_{complex}$[c]	ΔG_{total} (kcal/mol)[d]	Me_3PO→H–X bond interaction energy (kcal/mol)[e]	NBO charge on the hydrogen of of H–X bond
Me_3PO	-536.241732	-	-536.154069	-	-	-	-	-	-
**6a**-conformer A	-793.08684156	-	-792.92837	-	-	-	-	-	0.225
Me_3PO + **6a**-conformer A	-1329.328574	0.0	-1329.082439	0.0	-	-	0.0	-	-
Me_3PO•••**6a**-conformer A HB complex	-1329.3378741	-5.8	-1329.074083	5.2	-	-	5.2	7.5[f]	-

[a] Gibbs free energy relative to the most stable species. [b] relative population calculated based on ΔG at 298 K. The population of the most stable species is set as 1.0×10^{2}. [c] relative population calculated as a fraction of the population of all four species (ΣPop). [d] ΔG_{total} values are calculated based on $Pop_{unbound}$ and $Pop_{complex}$ at 298 K. [e] Calculated as the sum of the interaction energy of the lone pairs of the oxygen of Me_3PO with the σ* bond of the H–X moiety. [f] Calculated based on a single-point energy calculation at the PCM(MeCN)-M06-2X/6-31+G(2d,2p)//PCM(MeCN)-M06-2X/6-31+G(d,p) level of theory. A similar result was obtained at the PCM(MeCN)-B3LYP/6-31+G(d,p)//PCM(MeCN)-M06-2X/6-31+G(d,p) level as well (8.0 kcal/mol). The interaction energy is 196.28 kcal/mol at the PCM(MeCN)-M06-2X/6-31+G(d,p) level, a value that deviates significantly from expected values.

Table S14. Energy and population of **6b** and its HB complexes with Me_3PO.

PCM(MeCN)-M06-2X/6-31+G(d,p)								NBO analysis	
Conformer /species	E (hartree)	ΔE (kcal /mol)	Free Energy (hartree)	ΔG (kcal/ mol)[a]	Pop[b]	$Pop_{unbound}$ and $Pop_{complex}$[c]	ΔG_{total} (kcal/mol)[d]	Me_3PO→H–X bond interaction energy (kcal/mol)[e]	NBO charge on the hydrogen of of H–X bond
Me_3PO	-536.241732	-	-536.154069	-	-	-	-	-	-
**6b**-conformer A	-832.80568428	-	-832.606508	-	-	-	-	-	0.263
Me_3PO + **6b**-conformer A	-1369.047417	0.0	-1368.760577	0.0	-	-	0.0	-	-
Me_3PO•••**6b**-conformer A HB complex	-1369.0582733	-6.8	-1368.75457	3.8	-	-	3.8	16.2	-

[a] Gibbs free energy relative to the most stable species. [b] relative population calculated based on ΔG at 298 K. The population of the most stable species is set as 1.0×10^{2}. [c] relative population calculated as a fraction of the population of all four species (ΣPop). [d] ΔG_{total} values are calculated based on $Pop_{unbound}$ and $Pop_{complex}$ at 298 K. [e] Calculated as the sum of the interaction energy of the lone pairs of the oxygen of Me_3PO with the σ* bond of the H–X moiety.

Table S15. Energy and population of **7b** and its HB complexes with Me_3PO.

PCM(MeCN)-M06-2X/6-31+G(d,p)								NBO analysis	
Conformer /species	*E* (hartree)	Δ*E* (kcal /mol)	Free Energy (hartree)	Δ*G* (kcal/ mol)[a]	Pop[b]	$Pop_{unbound}$ and $Pop_{complex}$[c]	ΔG_{total} (kcal/mol)[d]	Me_3PO→H–X bond interaction energy (kcal/mol)[e]	NBO charge on the hydrogen of of H–X bond
Me_3PO	-536.241732	-	-536.154069	-	-	-	-	-	-
**7b**-conformer A	-640.12951093	-	-639.988648	-	-	-	-	-	0.245
**7b**-conformer B	-640.12988756	-	-639.989456	-	-	-	-	-	0.249
Me_3PO + **7b**-conformer A	-1176.371243	0.0	-1176.1427	0.5	4.2×10^{1}	1.0	0.0	-	-
Me_3PO + **7b**-conformer B	-1176.37162	-0.2	-1176.1435	0.0	1.0×10^{2}			-	-
Me_3PO•••**7b**-conformer A HB complex	-1176.3814383	-6.4	-1176.135357	5.1	1.8×10^{-2}	4.3×10^{-4}	4.6	9.5	-
Me_3PO•••**7b**-conformer B HB complex	-1176.3818506	-6.7	-1176.136234	4.6	4.4×10^{-1}			9.0	-

[a] Gibbs free energy relative to the most stable species. [b] relative population calculated based on Δ*G* at 298 K. The population of the most stable species is set as 1.0×10^{2}. [c] relative population calculated as a fraction of the population of all four species (Σ*Pop*). [d] ΔG_{total} values are calculated based on $Pop_{unbound}$ and $Pop_{complex}$ at 298 K. [e] Calculated as the sum of the interaction energy of the lone pairs of the oxygen of Me_3PO with the σ* bond of the H–X moiety.

Table S16. Energy and population of **8b** and its HB complexes with Me_3PO.

PCM(MeCN)-M06-2X/6-31+G(d,p)								NBO analysis	
Conformer /species	*E* (hartree)	Δ*E* (kcal /mol)	Free Energy (hartree)	Δ*G* (kcal/ mol)[a]	Pop[b]	$Pop_{unbound}$ and $Pop_{complex}$[c]	ΔG_{total} (kcal/mol)[d]	Me_3PO→H–X bond interaction energy (kcal/mol)[e]	NBO charge on the hydrogen of of H–X bond
Me_3PO	-536.241732	-	-536.154069	-	-	-	-	-	-
**8b**-conformer A	-564.94157006	-	-564.803585	-	-	-	-	-	0.246
**8b**-conformer B	-564.94039826	-	-564.801995	-	-	-	-	-	0.247
Me_3PO + **8b**-conformer A	-1101.183302	-0.7	-1100.957654	0.0	1.0×10^{2}	1.0	0.0	-	-
Me_3PO + **8b**-conformer B	-1101.182131	0.0	-1100.956064	1.0	1.9×10^{1}			-	-
Me_3PO•••**8b**-conformer A HB complex	-1101.1971504	-9.4	-1100.954013	2.3	2.1×10^{0}	1.9×10^{-2}	2.3	2.4	-
Me_3PO•••**8b**-conformer B HB complex	-1101.1939515	-7.4	-1100.951621	3.8	1.7×10^{-1}			9.8	-

[a] Gibbs free energy relative to the most stable species. [b] relative population calculated based on Δ*G* at 298 K. The population of the most stable species is set as 1.0×10^{2}. [c] relative population calculated as a fraction of the population of all four species (Σ*Pop*). [d] ΔG_{total} values are calculated based on $Pop_{unbound}$ and $Pop_{complex}$ at 298 K. [e] Calculated as the sum of the interaction energy of the lone pairs of the oxygen of Me_3PO with the σ* bond of the H–X moiety.

Table S17. Energy and population of **9b** and its HB complexes with Me_3PO.

PCM(MeCN)-M06-2X/6-31+G(d,p)								NBO analysis	
Conformer /species	*E* (hartree)	Δ*E* (kcal /mol)	Free Energy (hartree)	Δ*G* (kcal/ mol)[a]	Pop[b]	$Pop_{unbound}$ and $Pop_{complex}$[c]	ΔG_{total} (kcal/mol)[d]	Me_3PO→H–X bond interaction energy (kcal/mol)[e]	NBO charge on the hydrogen of of H–X bond
Me_3PO	-536.241732	-	-536.154069	-	-	-	-	-	-
**9b**-conformer A	-564.94470667	-	-564.806778	-	-	-	-	-	0.293
**9b**-conformer B	-564.94524776	-	-564.807247	-	-	-	-	-	0.294
Me_3PO + **9b**-conformer A	-1101.186439	0.0	-1100.960847	0.3	6.1×10^{1}	1.0	0.0	-	-
Me_3PO + **9b**-conformer B	-1101.18698	-0.3	-1100.961316	0.0	1.0×10^{2}			-	-
Me_3PO•••**9b**-conformer A HB complex	-1101.1965883	-6.4	-1100.953766	4.4	3.4×10^{-2}	2.0×10^{-3}	3.7	11.4	-
Me_3PO•••**9b**-conformer B HB complex	-1101.1967194	-6.5	-1100.955793	3.2	2.9×10^{-1}			10.0	-

[a] Gibbs free energy relative to the most stable species. [b] relative population calculated based on Δ*G* at 298 K. The population of the most stable species is set as 1.0×10^{2}. [c] relative population calculated as a fraction of the population of all four species (Σ*Pop*). [d] ΔG_{total} values are calculated based on $Pop_{unbound}$ and $Pop_{complex}$ at 298 K. [e] Calculated as the sum of the interaction energy of the lone pairs of the oxygen of Me_3PO with the σ* bond of the H–X moiety.

Table S18. Energy and population of **10** and its HB complexes with Me_3PO.

PCM(MeCN)-M06-2X/6-31+G(d,p)								NBO analysis	
Conformer /species	*E* (hartree)	Δ*E* (kcal /mol)	Free Energy (hartree)	Δ*G* (kcal/ mol)[a]	Pop[b]	$Pop_{unbound}$ and $Pop_{complex}$[c]	ΔG_{total} (kcal/mol)[d]	Me_3PO→H–X bond interaction energy (kcal/mol)[e]	NBO charge on the hydrogen of of H–X bond
Me_3PO	-536.241732	-	-536.154069	-	-	-	-	-	-
**10**-conformer A	-307.36080565	-	-307.284492	-	-	-	-	-	0.535
Me_3PO + **10**-conformer A	-843.6025379	0.0	-843.438561	0.0	-	-	0.0	-	-
Me_3PO•••**10**-conformer A HB complex	-843.6198452	-10.9	-843.439492	-0.6	-	-	-0.6	45.8	-

[a] Gibbs free energy relative to the most stable species. [b] relative population calculated based on Δ*G* at 298 K. The population of the most stable species is set as 1.0×10^2. [c] relative population calculated as a fraction of the population of all four species (Σ*Pop*). [d] ΔG_{total} values are calculated based on $Pop_{unbound}$ and $Pop_{complex}$ at 298 K. [e] Calculated as the sum of the interaction energy of the lone pairs of the oxygen of Me_3PO with the σ* bond of the H–X moiety.

Table S19. Energy and population of **12** and its HB complexes with Me_3PO.

PCM(MeCN)-M06-2X/6-31+G(d,p)								NBO analysis	
Conformer /species	*E* (hartree)	Δ*E* (kcal /mol)	Free Energy (hartree)	Δ*G* (kcal/ mol)[a]	Pop[b]	$Pop_{unbound}$ and $Pop_{complex}$[c]	ΔG_{total} (kcal/mol)[d]	Me_3PO→H–X bond interaction energy (kcal/mol)[e]	NBO charge on the hydrogen of of H–X bond
Me_3PO	-536.241732	-	-536.154069	-	-	-	-	-	-
**12**-conformer A	-1018.3630993	-	-1018.275939	-	-	-	-	-	0.250
**12**-conformer B	-1018.3616498	-	-1018.274694	-	-	-	-	-	0.245
Me_3PO + **12**-conformer A	-1554.604832	-0.9	-1554.430008	0.0	1.0×10^2	1.0	0.0	-	-
Me_3PO + **12**-conformer B	-1554.603382	0.0	-1554.428763	0.8	2.7×10^1			-	-
Me_3PO•••**12**-conformer A HB complex	-1554.615095	-7.3	-1554.423455	4.1	9.7×10^{-2}	$8.7\ x10^{-4}$	4.2	11.0	-
Me_3PO•••**12**-conformer B HB complex	-1554.6128808	-6.0	-1554.421568	5.3	1.3×10^{-2}			14.0	-

[a] Gibbs free energy relative to the most stable species. [b] relative population calculated based on Δ*G* at 298 K. The population of the most stable species is set as 1.0×10^2. [c] relative population calculated as a fraction of the population of all four species (Σ*Pop*). [d] ΔG_{total} values are calculated based on $Pop_{unbound}$ and $Pop_{complex}$ at 298 K. [e] Calculated as the sum of the interaction energy of the lone pairs of the oxygen of Me_3PO with the σ* bond of the H–X moiety.

Table S20. Energy and population of **13** and its HB complexes with Me_3PO.

PCM(MeCN)-M06-2X/6-31+G(d,p)								NBO analysis	
Conformer /species	*E* (hartree)	Δ*E* (kcal /mol)	Free Energy (hartree)	Δ*G* (kcal/ mol)[a]	Pop[b]	$Pop_{unbound}$ and $Pop_{complex}$[c]	ΔG_{total} (kcal/mol)[d]	Me_3PO→H–X bond interaction energy (kcal/mol)[e]	NBO charge on the hydrogen of of H–X bond
Me_3PO	-536.241732	-	-536.154069	-	-	-	-	-	-
**13**-conformer A	-674.31510777	-	-674.233402	-	-	-	-	-	0.237
**13**-conformer B	-674.31215286	-	-674.230149	-	-	-	-	-	0.228
Me_3PO + **13**-conformer A	-1210.55684	-1.9	-1210.3875	0.0	1.0×10^2	1.0	0.0	-	-
Me_3PO + **13**-conformer B	-1210.553885	0.0	-1210.3842	2.0	3.2×10^1			-	-
Me_3PO•••**13**-conformer A HB complex	-1210.5661581	-7.7	-1210.377903	6.0	4.0×10^{-3}	$4.5\ x10^{-5}$	5.9	3.2	-
Me_3PO•••**13**-conformer B HB complex	-1210.5628699	-5.6	-1210.376215	7.1	6.7×10^{-4}			3.5	-

[a] Gibbs free energy relative to the most stable species. [b] relative population calculated based on Δ*G* at 298 K. The population of the most stable species is set as 1.0×10^2. [c] relative population calculated as a fraction of the population of all four species (Σ*Pop*). [d] ΔG_{total} values are calculated based on $Pop_{unbound}$ and $Pop_{complex}$ at 298 K. [e] Calculated as the sum of the interaction energy of the lone pairs of the oxygen of Me_3PO with the σ* bond of the H–X moiety.

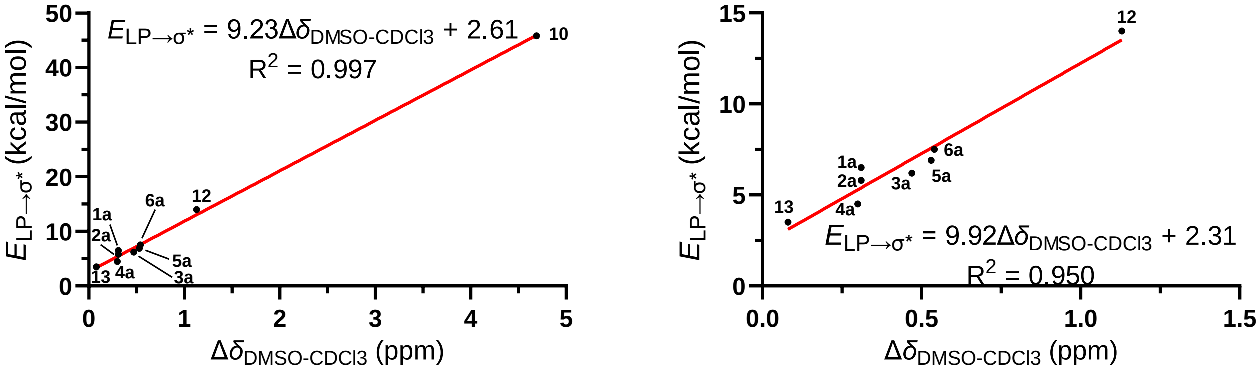

Figure S44. Linear correlation between $\Delta\delta_{DMSO\text{-}CDCl3}$ and *E* with the data for phenol (**10**) included (left) and excluded (right). The larger $E_{LP\rightarrow\sigma^*}$ value was used if two HB complexes were identified for a given HB donor–acceptor pair

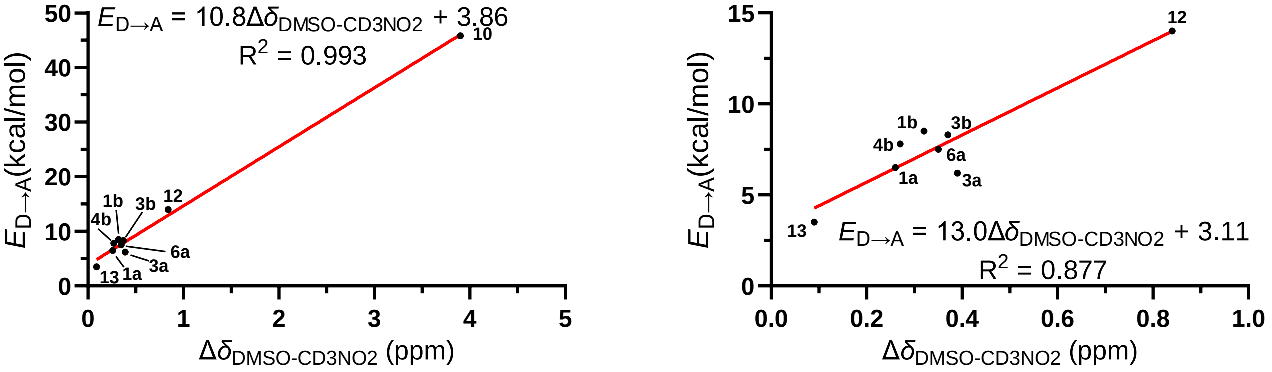

Figure S45. Linear correlation between $\Delta\delta_{DMSO\text{-}CD3NO2}$ and *E* with the data for phenol (**10**) included (left) and excluded (right). The larger $E_{LP\rightarrow\sigma^*}$ value was used if two HB complexes were identified for a given HB donor–acceptor pair

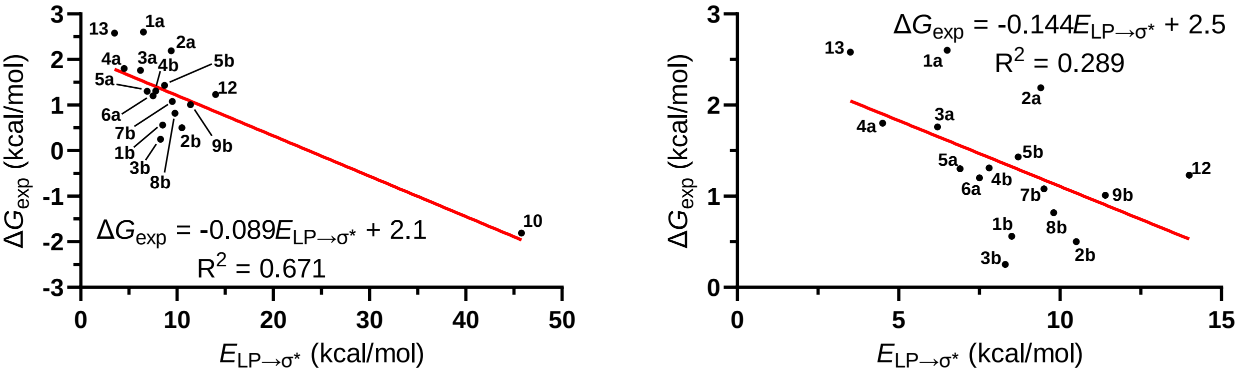

Figure S46. Linear correlation between ΔG_{exp} and *E* with the data for phenol (**10**) included (left) and excluded (right). The larger $E_{LP\rightarrow\sigma^*}$ value was used if two HB complexes were identified for a given HB donor–acceptor pair

Table S21. Energy and population of **1a** and its HB complexes with Me_3PO at the PCM(MeCN)-M06-2X/6-311++G(2d,2p)//M06-2X/6-31+G(d,p) level.

Conformer /species	E (hartree)	Thermal correction (hartree)[a]	Gibbs Free Energy (hartree)	ΔG (kcal/mol)[b]	Pop[c]	$Pop_{unbound}$ and $Pop_{complex}$[d]	ΔG_{total} (kcal/mol)[e]
Me_3PO	-536.3373497	0.087664	-536.2496857	-	-	-	-
**1a**-conformer A	-486.0568172	0.069786	-485.9870312	-	-	-	-
**1a**-conformer B	-486.0558949	0.070340	-485.9855549	-	-	-	-
Me_3PO + **1a**-conformer A	-1022.3941668	-	-1022.2367168	0.00	1.0×10^{2}	1.0	0.0
Me_3PO + **1a**-conformer B	-1022.3932446	-	-1022.2352406	0.9	2.0×10^{1}		
Me_3PO•••**1a**-conformer A HB complex	-1022.4020375	0.176406	-1022.2256315	7.0	8.0×10^{-4}	1.3×10^{-5}	6.7
Me_3PO•••**1a**-conformer B HB complex	-1022.3986142	0.172982	-1022.2256322	7.0	8.0×10^{-4}		

[a] Thermal correction to Gibbs Free Energy caluclated at the PCM(MeCN)-M06-2X/6-31+G(d,p) level. [b] Gibbs free energy relative to the most stable species. [c] relative population calculated based on ΔG at 298 K. The population of the most stable species is set as 1.0×10^{2}. [d] relative population calculated as a fraction of the population of all four species (ΣPop). [e] ΔG_{total} values are calculated based on $Pop_{unbound}$ and $Pop_{complex}$ at 298 K.

Table S22. Energy and population of **1b** and its HB complexes with Me_3PO at the PCM(MeCN)-M06-2X/6-311++G(2d,2p)//M06-2X/6-31+G(d,p) level.

Conformer /species	E (hartree)	Thermal correction (hartree)[a]	Gibbs Free Energy (hartree)	ΔG (kcal/mol)[b]	Pop[c]	$Pop_{unbound}$ and $Pop_{complex}$[d]	ΔG_{total} (kcal/mol)[e]
Me_3PO	-536.3373497	0.087664	-536.2496857	-	-	-	-
**1b**-conformer A	-525.7853681	0.111336	-525.6740321	-	-	-	-
**1b**-conformer B	-525.7850249	0.111592	-525.6734329	-	-	-	-
Me_3PO + **1b**-conformer A	-1062.1227177	-	-1061.9237177	0.0	1.0×10^{2}	1.0	0.0
Me_3PO + **1b**-conformer B	-1062.1223746	-	-1061.9231186	0.4	5.3×10^{1}		
Me_3PO•••**1b**-conformer A HB complex	-1062.1363011	0.218106	-1061.9181951	3.5	2.9×10^{-1}	2.2×10^{-3}	3.6
Me_3PO•••**1b**-conformer B HB complex	-1062.1315969	0.215208	-1061.9163889	4.6	4.3×10^{-2}		

[a] Thermal correction to Gibbs Free Energy caluclated at the PCM(MeCN)-M06-2X/6-31+G(d,p) level. [b] Gibbs free energy relative to the most stable species. [c] relative population calculated based on ΔG at 298 K. The population of the most stable species is set as 1.0×10^{2}. [d] relative population calculated as a fraction of the population of all four species (ΣPop). [e] ΔG_{total} values are calculated based on $Pop_{unbound}$ and $Pop_{complex}$ at 298 K.

Table S23. Energy and population of **2a** and its HB complexes with Me_3PO at the PCM(MeCN)-M06-2X/6-311++G(2d,2p)//M06-2X/6-31+G(d,p) level.

Conformer /species	E (hartree)	Thermal correction (hartree)[a]	Gibbs Free Energy (hartree)	ΔG (kcal/mol)[b]	Pop[c]	$Pop_{unbound}$ and $Pop_{complex}$[d]	ΔG_{total} (kcal/mol)[e]
Me_3PO	-536.3373497	0.087664	-536.2496857	-	-	-	-
**2a**-conformer A	-639.6831535	0.113278	-639.5698755	-	-	-	-
**2a**-conformer B	-639.6811222	0.112647	-639.5684752	-	-	-	-
Me_3PO + **2a**-conformer A	-1176.0205032	-	-1175.8195612	0.0	1.0×10^{2}	1.0	0.0
Me_3PO + **2a**-conformer B	-1176.0184719	-	-1175.8181609	0.9	2.3×10^{1}		
Me_3PO•••**2a**-conformer A HB complex	-1176.0269584	0.218463	-1175.8084954	6.9	8.1×10^{-4}	8.4×10^{-6}	6.9
Me_3PO•••**2a**-conformer B HB complex	-1176.0243153	0.217061	-1175.8072543	7.7	2.2×10^{-4}		

[a] Thermal correction to Gibbs Free Energy caluclated at the PCM(MeCN)-M06-2X/6-31+G(d,p) level. [b] Gibbs free energy relative to the most stable species. [c] relative population calculated based on ΔG at 298 K. The population of the most stable species is set as 1.0×10^{2}. [d] relative population calculated as a fraction of the population of all four species (ΣPop). [e] ΔG_{total} values are calculated based on $Pop_{unbound}$ and $Pop_{complex}$ at 298 K.

Table S24. Energy and population of **2b** and its HB complexes with Me_3PO at the PCM(MeCN)-M06-2X/6-311++G(2d,2p)//M06-2X/6-31+G(d,p) level.

Conformer /species	E (hartree)	Thermal correction (hartree)[a]	Gibbs Free Energy (hartree)	ΔG (kcal/mol)[b]	Pop[c]	$Pop_{unbound}$ and $Pop_{complex}$[d]	ΔG_{total} (kcal/mol)[e]
Me_3PO	-536.3373497	0.087664	-536.2496857	-	-	-	-
**2b**-conformer A	-679.4076912	0.155283	-679.2524082	-	-	-	-
**2b**-conformer B	-679.4073671	0.155398	-679.2519691	-	-	-	-
Me_3PO + **2b**-conformer A	-1215.7450409	-	-1215.5020939	0.0	1.0×10^{2}	1.0	0.0
Me_3PO + **2b**-conformer B	-1215.7447168	-	-1215.5016548	0.3	6.3×10^{1}		
Me_3PO•••**2b**-conformer A HB complex	-1215.754749	0.260378	-1215.4943706	4.9	2.8×10^{-2}	5.9×10^{-4}	4.4
Me_3PO•••**2b**-conformer B HB complex	-1215.75386	0.258661	-1215.4951988	4.3	6.7×10^{-2}		

[a] Thermal correction to Gibbs Free Energy caluclated at the PCM(MeCN)-M06-2X/6-31+G(d,p) level. [b] Gibbs free energy relative to the most stable species. [c] relative population calculated based on ΔG at 298 K. The population of the most stable species is set as 1.0×10^{2}. [d] relative population calculated as a fraction of the population of all four species (ΣPop). [e] ΔG_{total} values are calculated based on $Pop_{unbound}$ and $Pop_{complex}$ at 298 K.

Table S25. Energy and population of **3a** and its HB complexes with Me_3PO at the PCM(MeCN)-M06-2X/6-311++G(2d,2p)//M06-2X/6-31+G(d,p) level.

Conformer /species	*E* (hartree)	Thermal correction (hartree)[a]	Gibbs Free Energy (hartree)	Δ*G* (kcal/mol)[b]	Pop[c]	$Pop_{unbound}$ and $Pop_{complex}$[d]	ΔG_{total} (kcal/mol)[e]
Me_3PO	-536.3373497	0.087664	-536.2496857	-	-	-	-
**3a**-conformer A	-656.9334791	0.123751	-656.8097281	-	-	-	-
**3a**-conformer B	-656.9358025	0.124011	-656.8117915	-	-	-	-
Me_3PO + **3a**-conformer A	-1193.2708287	-	-1193.0594137	1.3	1.1×10^{1}	1.0	0.0
Me_3PO + **3a**-conformer B	-1193.2731521	-	-1193.0614771	0.0	1.0×10^{2}		
Me_3PO•••**3a**-conformer A HB complex	-1193.2768663	0.231795	-1193.0450713	10.3	2.8×10^{-6}	8.2×10^{-6}	6.9
Me_3PO•••**3a**-conformer B HB complex	-1193.2802022	0.229692	-1193.0505102	6.9	9.0×10^{-4}		

[a] Thermal correction to Gibbs Free Energy caluclated at the PCM(MeCN)-M06-2X/6-31+G(d,p) level. [b] Gibbs free energy relative to the most stable species. [c] relative population calculated based on Δ*G* at 298 K. The population of the most stable species is set as 1.0×10^{2}. [d] relative population calculated as a fraction of the population of all four species (Σ*Pop*). [e] ΔG_{total} values are calculated based on $Pop_{unbound}$ and $Pop_{complex}$ at 298 K.

Table S26. Energy and population of **3b** and its HB complexes with Me_3PO at the PCM(MeCN)-M06-2X/6-311++G(2d,2p)//M06-2X/6-31+G(d,p) level.

Conformer /species	*E* (hartree)	Thermal correction (hartree)[a]	Gibbs Free Energy (hartree)	Δ*G* (kcal/mol)[b]	Pop[c]	$Pop_{unbound}$ and $Pop_{complex}$[d]	ΔG_{total} (kcal/mol)[e]
Me_3PO	-536.3373497	0.087664	-536.2496857	-	-	-	-
**3b**-conformer A	-696.6682661	0.163462	-696.5048041	-	-	-	-
**3b**-conformer B	-696.6669450	0.164506	-696.5024390	-	-	-	-
Me_3PO + **3b**-conformer A	-1233.0056157	-	-1232.7544897	0.0	1.0×10^{2}	1.0	0.0
Me_3PO + **3b**-conformer B	-1233.0042946	-	-1232.7521246	1.5	8.2×10^{0}		
Me_3PO•••**3b**-conformer A HB complex	-1233.0172165	0.269516	-1232.7477005	4.3	7.5×10^{-2}	5.5×10^{-3}	3.1
Me_3PO•••**3b**-conformer B HB complex	-1233.0200538	0.270524	-1232.7495298	3.1	5.2×10^{-1}		

[a] Thermal correction to Gibbs Free Energy caluclated at the PCM(MeCN)-M06-2X/6-31+G(d,p) level. [b] Gibbs free energy relative to the most stable species. [c] relative population calculated based on Δ*G* at 298 K. The population of the most stable species is set as 1.0×10^{2}. [d] relative population calculated as a fraction of the population of all four species (Σ*Pop*). [e] ΔG_{total} values are calculated based on $Pop_{unbound}$ and $Pop_{complex}$ at 298 K.

Table S27. Energy and population of **4a** and its HB complexes with Me_3PO at the PCM(MeCN)-M06-2X/6-311++G(2d,2p)//M06-2X/6-31+G(d,p) level.

Conformer /species	*E* (hartree)	Thermal correction (hartree)[a]	Gibbs Free Energy (hartree)	Δ*G* (kcal/mol)[b]	Pop[c]	$Pop_{unbound}$ and $Pop_{complex}$[d]	ΔG_{total} (kcal/mol)[e]
Me_3PO	-536.3373497	0.087664	-536.2496857	-	-	-	-
**4a**-conformer A	-486.0542119	0.070044	-485.9841679	-	-	-	-
**4a**-conformer B	-486.0546571	0.070046	-485.9846111	-	-	-	-
Me_3PO + **4a**-conformer A	-1022.3915616	-	-1022.2338536	0.3	6.3×10^{1}	1.0	0.0
Me_3PO + **4a**-conformer B	-1022.3920068	-	-1022.2342968	0.0	1.0×10^{2}		
Me_3PO•••**4a**-conformer A HB complex	-1022.3993980	0.174971	-1022.2244270	6.2	2.9×10^{-3}	3.1×10^{-5}	6.2
Me_3PO•••**4a**-conformer B HB complex	-1022.4002200	0.176092	-1022.2241280	6.4	2.1×10^{-3}		

[a] Thermal correction to Gibbs Free Energy caluclated at the PCM(MeCN)-M06-2X/6-31+G(d,p) level. [b] Gibbs free energy relative to the most stable species. [c] relative population calculated based on Δ*G* at 298 K. The population of the most stable species is set as 1.0×10^{2}. [d] relative population calculated as a fraction of the population of all four species (Σ*Pop*). [e] ΔG_{total} values are calculated based on $Pop_{unbound}$ and $Pop_{complex}$ at 298 K.

Table S28. Energy and population of **4b** and its HB complexes with Me_3PO at the PCM(MeCN)-M06-2X/6-311++G(2d,2p)//M06-2X/6-31+G(d,p) level.

Conformer /species	*E* (hartree)	Thermal correction (hartree)[a]	Gibbs Free Energy (hartree)	Δ*G* (kcal/mol)[b]	Pop[c]	$Pop_{unbound}$ and $Pop_{complex}$[d]	ΔG_{total} (kcal/mol)[e]
Me_3PO	-536.3373497	0.087664	-536.2496857	-	-	-	-
**4b**-conformer A	-525.7903632	0.109408	-525.6809552	-	-	-	-
**4b**-conformer B	-525.7916076	0.109425	-525.6821826	-	-	-	-
Me_3PO + **4b**-conformer A	-1062.1277129	-	-1061.9306409	0.8	2.7×10^{1}	1.0	0.0
Me_3PO + **4b**-conformer B	-1062.1289573	-	-1061.9318683	0.0	1.0×10^{2}		
Me_3PO•••**4b**-conformer A HB complex	-1062.1359621	0.213276	-1061.9226861	5.8	6.0×10^{-3}	6.3×10^{-5}	5.7
Me_3PO•••**4b**-conformer B HB complex	-1062.1377955	0.216131	-1061.9216645	6.4	2.0×10^{-3}		

[a] Thermal correction to Gibbs Free Energy caluclated at the PCM(MeCN)-M06-2X/6-31+G(d,p) level. [b] Gibbs free energy relative to the most stable species. [c] relative population calculated based on Δ*G* at 298 K. The population of the most stable species is set as 1.0×10^{2}. [d] relative population calculated as a fraction of the population of all four species (Σ*Pop*). [e] ΔG_{total} values are calculated based on $Pop_{unbound}$ and $Pop_{complex}$ at 298 K.

Table S29. Energy and population of **5a** and its HB complexes with Me_3PO at the PCM(MeCN)-M06-2X/6-311++G(2d,2p)//M06-2X/6-31+G(d,p) level.

Conformer /species	*E* (hartree)	Thermal correction (hartree)[a]	Gibbs Free Energy (hartree)	Δ*G* (kcal/mol)[b]	Pop[c]	$Pop_{unbound}$ and $Pop_{complex}$[d]	ΔG_{total} (kcal/mol)[e]
Me_3PO	-536.3373497	0.087664	-536.2496857	-	-	-	-
**5a**-conformer A	-639.6798481	0.114607	-639.5652411	-	-	-	-
**5a**-conformer B	-639.6798705	0.114646	-639.5652245	-	-	-	-
Me_3PO + **5a**-conformer A	-1176.0171978	-	-1175.8149268	0.0	1.0×10^{2}	1.0	0.0
Me_3PO + **5a**-conformer B	-1176.0172202	-	-1175.8149102	0.0	9.9×10^{1}		
Me_3PO•••**5a**-conformer A HB complex	-1176.0276320	0.221524	-1175.8061080	5.5	8.8×10^{-3}	6.5×10^{-5}	5.7
Me_3PO•••**5a**-conformer B HB complex	-1176.0228959	0.217509	-1175.8053869	6.0	4.1×10^{-3}		

[a] Thermal correction to Gibbs Free Energy caluclated at the PCM(MeCN)-M06-2X/6-31+G(d,p) level. [b] Gibbs free energy relative to the most stable species. [c] relative population calculated based on Δ*G* at 298 K. The population of the most stable species is set as 1.0×10^{2}. [d] relative population calculated as a fraction of the population of all four species (Σ*Pop*). [e] ΔG_{total} values are calculated based on $Pop_{unbound}$ and $Pop_{complex}$ at 298 K.

Table S30. Energy and population of **5b** and its HB complexes with Me_3PO at the PCM(MeCN)-M06-2X/6-311++G(2d,2p)//M06-2X/6-31+G(d,p) level.

Conformer /species	*E* (hartree)	Thermal correction (hartree)[a]	Gibbs Free Energy (hartree)	Δ*G* (kcal/mol)[b]	Pop[c]	$Pop_{unbound}$ and $Pop_{complex}$[d]	ΔG_{total} (kcal/mol)[e]
Me_3PO	-536.3373497	0.087664	-536.2496857	-	-	-	-
**5b**-conformer A	-679.4147435	0.155569	-679.2591745	-	-	-	-
**5b**-conformer B	-679.4154077	0.155547	-679.2598607	-	-	-	-
Me_3PO + **5b**-conformer A	-1215.7520932	-	-1215.5088602	0.4	4.8×10^{1}	1.0	0.0
Me_3PO + **5b**-conformer B	-1215.7527574	-	-1215.5095464	0.0	1.0×10^{2}		
Me_3PO•••**5b**-conformer A HB complex	-1215.7607060	0.259025	-1215.5016810	4.9	2.4×10^{-2}	1.9×10^{-4}	5.1
Me_3PO•••**5b**-conformer B HB complex	-1215.7612544	0.261178	-1215.5000764	5.9	4.4×10^{-3}		

[a] Thermal correction to Gibbs Free Energy caluclated at the PCM(MeCN)-M06-2X/6-31+G(d,p) level. [b] Gibbs free energy relative to the most stable species. [c] relative population calculated based on Δ*G* at 298 K. The population of the most stable species is set as 1.0×10^{2}. [d] relative population calculated as a fraction of the population of all four species (Σ*Pop*). [e] ΔG_{total} values are calculated based on $Pop_{unbound}$ and $Pop_{complex}$ at 298 K.

Table S31. Energy and population of **6a** and its HB complexes with Me_3PO at the PCM(MeCN)-M06-2X/6-311++G(2d,2p)//M06-2X/6-31+G(d,p) level.

Conformer /species	*E* (hartree)	Thermal correction (hartree)[a]	Gibbs Free Energy (hartree)	Δ*G* (kcal/mol)[b]	Pop[c]	$Pop_{unbound}$ and $Pop_{complex}$[d]	ΔG_{total} (kcal/mol)[e]
Me_3PO	-536.3373497	0.087664	-536.2496857	-	-	-	-
**6a**-conformer A	-793.2937451	0.158472	-536.2496857	-	-	-	-
Me_3PO + **6a**-conformer A	-1329.6310947	-	-1329.3849587	0.0	-	-	0.0
Me_3PO•••**6a**-conformer A HB complex	-1329.6400500	0.263791	-1329.3762590	5.5	-	-	5.5

[a] Thermal correction to Gibbs Free Energy caluclated at the PCM(MeCN)-M06-2X/6-31+G(d,p) level. [b] Gibbs free energy relative to the most stable species. [c] relative population calculated based on Δ*G* at 298 K. The population of the most stable species is set as 1.0×10^{2}. [d] relative population calculated as a fraction of the population of all four species (Σ*Pop*). [e] ΔG_{total} values are calculated based on $Pop_{unbound}$ and $Pop_{complex}$ at 298 K.

Table S32. Energy and population of **6b** and its HB complexes with Me_3PO at the PCM(MeCN)-M06-2X/6-311++G(2d,2p)//M06-2X/6-31+G(d,p) level.

Conformer /species	*E* (hartree)	Thermal correction (hartree)[a]	Gibbs Free Energy (hartree)	Δ*G* (kcal/mol)[b]	Pop[c]	$Pop_{unbound}$ and $Pop_{complex}$[d]	ΔG_{total} (kcal/mol)[e]
Me_3PO	-536.3373497	0.087664	-536.2496857	-	-	-	-
**6b**-conformer A	-833.0232677	0.199176	-832.8240917	-	-	-	-
Me_3PO + **6b**-conformer A	-1369.3606174	-	-1369.073777	0.0	-	-	0.0
Me_3PO•••**6b**-conformer A HB complex	-1369.3705843	0.303869	-1369.0667153	4.4	-	-	4.4

[a] Thermal correction to Gibbs Free Energy caluclated at the PCM(MeCN)-M06-2X/6-31+G(d,p) level. [b] Gibbs free energy relative to the most stable species. [c] relative population calculated based on Δ*G* at 298 K. The population of the most stable species is set as 1.0×10^{2}. [d] relative population calculated as a fraction of the population of all four species (Σ*Pop*). [e] ΔG_{total} values are calculated based on $Pop_{unbound}$ and $Pop_{complex}$ at 298 K.

Table S33. Energy and population of **7b** and its HB complexes with Me_3PO at the PCM(MeCN)-M06-2X/6-311++G(2d,2p)//M06-2X/6-31+G(d,p) level.

Conformer /species	*E* (hartree)	Thermal correction (hartree)^a	Gibbs Free Energy (hartree)	Δ*G* (kcal/mol)^b	Pop^c	$Pop_{unbound}$ and $Pop_{complex}$^{d}	ΔG_{total} (kcal/mol)^e
Me_3PO	-536.3373497	0.087664	-536.2496857	-	-	-	-
**7b**-conformer A	-640.3096689	0.140863	-640.1688059	-	-	-	-
**7b**-conformer B	-640.3098576	0.140431	-640.1694266	-	-	-	-
Me_3PO + **7b**-conformer A	-1176.6470186	-	-1176.4184916	0.4	5.2×10^{1}	1.0	0.0
Me_3PO + **7b**-conformer B	-1176.6472073	-	-1176.4191123	0.0	1.0×10^{2}		
Me_3PO•••**7b**-conformer A HB complex	-1176.6564819	0.246081	-1176.4104009	5.5	1.0×10^{-2}	2.1×10^{-4}	5.0
Me_3PO•••**7b**-conformer B HB complex	-1176.6567580	0.245616	-1176.4111420	5.0	2.2×10^{-2}		

^{a} Thermal correction to Gibbs Free Energy caluclated at the PCM(MeCN)-M06-2X/6-31+G(d,p) level. ^{b} Gibbs free energy relative to the most stable species. ^{c} relative population calculated based on Δ*G* at 298 K. The population of the most stable species is set as 1.0×10^2. ^{d} relative population calculated as a fraction of the population of all four species (Σ*Pop*). ^{e} ΔG_{total} values are calculated based on $Pop_{unbound}$ and $Pop_{complex}$ at 298 K.

Table S34. Energy and population of **8b** and its HB complexes with Me_3PO at the PCM(MeCN)-M06-2X/6-311++G(2d,2p)//M06-2X/6-31+G(d,p) level.

Conformer /species	*E* (hartree)	Thermal correction (hartree)^a	Gibbs Free Energy (hartree)	Δ*G* (kcal/mol)^b	Pop^c	$Pop_{unbound}$ and $Pop_{complex}$^{d}	ΔG_{total} (kcal/mol)^e
Me_3PO	-536.3373497	0.087664	-536.2496857	-	-	-	-
**8b**-conformer A	-565.0974563	0.137985	-564.9594713	-	-	-	-
**8b**-conformer B	-565.0960896	0.138403	-564.9576866	-	-	-	-
Me_3PO + **8b**-conformer A	-1101.4348060	-	-1101.2091570	0.0	1.0×10^{2}	0.99	0.0
Me_3PO + **8b**-conformer B	-1101.4334393	-	-1101.2073723	1.1	1.5×10^{1}		
Me_3PO•••**8b**-conformer A HB complex	-1101.4478042	0.243137	-1101.2046672	2.8	8.6×10^{-1}	8.0×10^{-3}	2.9
Me_3PO•••**8b**-conformer B HB complex	-1101.4445982	0.24233	-1101.2022682	4.3	6.8×10^{-2}		

^{a} Thermal correction to Gibbs Free Energy caluclated at the PCM(MeCN)-M06-2X/6-31+G(d,p) level. ^{b} Gibbs free energy relative to the most stable species. ^{c} relative population calculated based on Δ*G* at 298 K. The population of the most stable species is set as 1.0×10^2. ^{d} relative population calculated as a fraction of the population of all four species (Σ*Pop*). ^{e} ΔG_{total} values are calculated based on $Pop_{unbound}$ and $Pop_{complex}$ at 298 K.

Table S35. Energy and population of **9b** and its HB complexes with Me_3PO at the PCM(MeCN)-M06-2X/6-311++G(2d,2p)//M06-2X/6-31+G(d,p) level.

Conformer /species	*E* (hartree)	Thermal correction (hartree)^a	Gibbs Free Energy (hartree)	Δ*G* (kcal/mol)^b	Pop^c	$Pop_{unbound}$ and $Pop_{complex}$^{d}	ΔG_{total} (kcal/mol)^e
Me_3PO	-536.3373497	0.087664	-536.2496857	-	-	-	-
**9b**-conformer A	-565.1003940	0.137929	-564.9624650	-	-	-	-
**9b**-conformer B	-565.1008325	0.138001	-564.9628315	-	-	-	-
Me_3PO + **9b**-conformer A	-1101.4377436	-	-1101.2121506	0.2	6.8×10^{1}	1.0	0.0
Me_3PO + **9b**-conformer B	-1101.4381822	-	-1101.2125172	0.0	1.0×10^{2}		
Me_3PO•••**9b**-conformer A HB complex	-1101.4469983	0.242822	-1101.2041763	5.2	1.5×10^{-2}	8.7×10^{-4}	4.2
Me_3PO•••**9b**-conformer B HB complex	-1101.4471801	0.240927	-1101.2062531	3.9	1.3×10^{-1}		

^{a} Thermal correction to Gibbs Free Energy caluclated at the PCM(MeCN)-M06-2X/6-31+G(d,p) level. ^{b} Gibbs free energy relative to the most stable species. ^{c} relative population calculated based on Δ*G* at 298 K. The population of the most stable species is set as 1.0×10^2. ^{d} relative population calculated as a fraction of the population of all four species (Σ*Pop*). ^{e} ΔG_{total} values are calculated based on $Pop_{unbound}$ and $Pop_{complex}$ at 298 K.

Table S36. Energy and population of **10** and its HB complexes with Me_3PO at the PCM(MeCN)-M06-2X/6-311++G(2d,2p)//M06-2X/6-31+G(d,p) level.

Conformer /species	*E* (hartree)	Thermal correction (hartree)^a	Gibbs Free Energy (hartree)	Δ*G* (kcal/mol)^b	Pop^c	$Pop_{unbound}$ and $Pop_{complex}$^{d}	ΔG_{total} (kcal/mol)^e
Me_3PO	-536.3373497	0.087664	-536.2496857	-	-	-	-
**10**-conformer A	-307.4430557	0.076314	-307.3667417	-	-	-	-
Me_3PO + **10**-conformer A	-843.7804054	-	-843.6164274	0.0	-	-	0.0
Me_3PO•••**10**-conformer A HB complex	-843.7968307	0.180353	-843.6164777	0.0	-	-	0.0

^{a} Thermal correction to Gibbs Free Energy caluclated at the PCM(MeCN)-M06-2X/6-31+G(d,p) level. ^{b} Gibbs free energy relative to the most stable species. ^{c} relative population calculated based on Δ*G* at 298 K. The population of the most stable species is set as 1.0×10^2. ^{d} relative population calculated as a fraction of the population of all four species (Σ*Pop*). ^{e} ΔG_{total} values are calculated based on $Pop_{unbound}$ and $Pop_{complex}$ at 298 K.

Table S37. Energy and population of **12** and its HB complexes with Me_3PO at the PCM(MeCN)-M06-2X/6-311++G(2d,2p)//M06-2X/6-31+G(d,p) level.

Conformer /species	*E* (hartree)	Thermal correction (hartree)[a]	Gibbs Free Energy (hartree)	Δ*G* (kcal/mol)[b]	Pop[c]	$Pop_{unbound}$ and $Pop_{complex}$[d]	ΔG_{total} (kcal/mol)[e]
Me_3PO	-536.3373497	0.087664	-536.2496857	-	-	-	-
**12**-conformer A	-1018.5958677	0.087160	-1018.5087077	-	-	-	-
**12**-conformer B	-1018.5944157	0.086955	-1018.5074607	-	-	-	-
Me_3PO + **12**-conformer A	-1554.9332173	-	-1554.7583933	0.0	1.0×10^{2}	1.0	0.0
Me_3PO + **12**-conformer B	-1554.9317654	-	-1554.7571464	0.8	2.7×10^{1}		
Me_3PO•••**12**-conformer A HB complex	-1554.9421694	0.191639	-1554.7505304	4.9	2.4×10^{-2}	2.2×10^{-4}	5.0
Me_3PO•••**12**-conformer B HB complex	-1554.9401112	0.191313	-1554.7487982	6.0	3.9×10^{-3}		

[a] Thermal correction to Gibbs Free Energy caluclated at the PCM(MeCN)-M06-2X/6-31+G(d,p) level. [b] Gibbs free energy relative to the most stable species. [c] relative population calculated based on Δ*G* at 298 K. The population of the most stable species is set as 1.0×10^{2}. [d] relative population calculated as a fraction of the population of all four species (Σ*Pop*). [e] ΔG_{total} values are calculated based on $Pop_{unbound}$ and $Pop_{complex}$ at 298 K.

Table S38. Energy and population of **13** and its HB complexes with Me_3PO at the PCM(MeCN)-M06-2X/6-311++G(2d,2p)//M06-2X/6-31+G(d,p) level.

Conformer /species	*E* (hartree)	Thermal correction (hartree)[a]	Gibbs Free Energy (hartree)	Δ*G* (kcal/mol)[b]	Pop[c]	$Pop_{unbound}$ and $Pop_{complex}$[d]	ΔG_{total} (kcal/mol)[e]
Me_3PO	-536.3373497	0.087664	-536.2496857	-	-	-	-
**13**-conformer A	-674.5085424	0.081706	-674.4268364	-	-	-	-
**13**-conformer B	-674.5055850	0.082004	-674.4235810	-	-	-	-
Me_3PO + **13**-conformer A	-1210.8458921	-	-1210.6765221	0.0	1.0×10^{2}	1.0	0.0
Me_3PO + **13**-conformer B	-1210.8429347	-	-1210.6732667	2.0	3.2×10^{0}		
Me_3PO•••**13**-conformer A HB complex	-1210.8547756	0.188255	-1210.6665206	6.3	2.5×10^{-3}	3.0×10^{-5}	6.2
Me_3PO•••**13**-conformer B HB complex	-1210.8518580	0.186655	-1210.6652030	7.1	6.2×10^{-4}		

[a] Thermal correction to Gibbs Free Energy caluclated at the PCM(MeCN)-M06-2X/6-31+G(d,p) level. [b] Gibbs free energy relative to the most stable species. [c] relative population calculated based on Δ*G* at 298 K. The population of the most stable species is set as 1.0×10^{2}. [d] relative population calculated as a fraction of the population of all four species (Σ*Pop*). [e] ΔG_{total} values are calculated based on $Pop_{unbound}$ and $Pop_{complex}$ at 298 K.

Table S39. Summary of ΔG_{exp} and ΔG_{calc} values obtained at the PCM(MeCN)-M06-2X/6-31+G(d,p) and the PCM(MeCN)-M06-2X/6-311++G(2d,2p)//M06-2X/6-31+G(d,p) levels.

species	ΔG_{exp} (kcal/mol)	ΔG_{calc} (kcal/mol) PCM(MeCN)-M06-2X/6-31+G(d,p)	ΔG_{calc} (kcal/mol) PCM(MeCN)-M06-2X/6-311++G(2d,2p)//M06-2X/6-31+G(d,p)
**1a**	2.6	4.5	4.8
**1b**	0.56	1.3	1.7
**2a**	2.2	4.7	5.0
**2b**	0.50	1.9	2.5
**3a**	1.8	3.4	5.0
**3b**	0.25	0.6	1.2
**4a**	1.8	4.1	4.3
**4b**	1.3	3.4	3.8
**5a**	1.3	3.6	3.8
**5b**	1.4	2.6	3.2
**6a**	1.2	3.3	3.6
**6b**	-	1.9	4.4
**7b**	1.1	3.1	3.1
**8b**	0.82	0.4	1.0
**9b**	1.0	1.8	2.3
**10**	-1.9	-2.5	-1.8
**12**	1.2	2.3	3.1
**13**	2.5	4.0	4.3

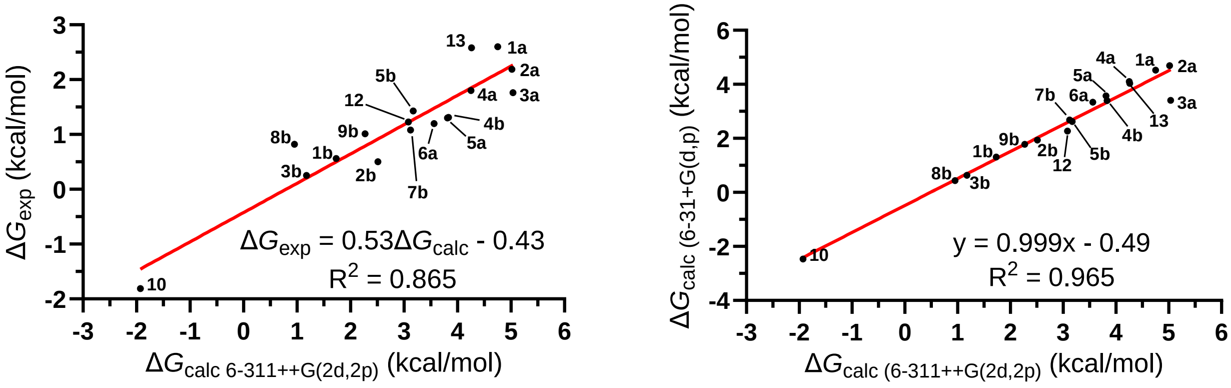

Figure S47. Left panel. Linear correlation between ΔG_{exp} and ΔG_{calc} at the the PCM(MeCN)-M06-2X/6-311++G(2d,2p)//M06-2X/6-31+G(d,p) level. Right panel. Linear correlation between ΔG_{calc} values calculated at the the PCM(MeCN)-M06-2X/6-311++G(2d,2p)//M06-2X/6-31+G(d,p) level and the PCM(MeCN)-M06-2X/6-31+G(d,p) level.

Optimized coordinates in acetonitrile

Cationic species:

2-(Difluoromethyl)-*N*-methylpyridinium Conformer A

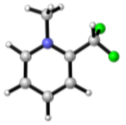

Standard orientation:

Center Number	Atomic Number	Atomic Type	X	Y	Z
1	6	0	2.577397	-0.375474	0.138967
2	6	0	1.826149	-1.542218	0.121775
3	6	0	0.440169	-1.459193	-0.015316
4	6	0	-0.158403	-0.220370	-0.127471
5	6	0	1.929355	0.842069	0.018640
6	1	0	2.305857	-2.509759	0.213614
7	1	0	-0.180108	-2.345575	-0.037020
8	1	0	2.458639	1.786040	0.023462
9	6	0	-1.663648	-0.075968	-0.256090
10	1	0	-1.984668	0.543722	-1.095863
11	9	0	-2.209744	-1.305276	-0.389231
12	9	0	-2.153292	0.462046	0.897526
13	1	0	3.654671	-0.388496	0.242480
14	7	0	0.591565	0.909594	-0.111367
15	6	0	-0.045350	2.240628	-0.257429
16	1	0	-0.460879	2.325814	-1.261503
17	1	0	-0.820416	2.354510	0.497367
18	1	0	0.719263	2.998808	-0.116086

2-(Difluoromethyl)-*N*-methylpyridinium Conformer A

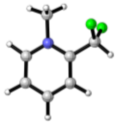

Standard orientation:

Center Number	Atomic Number	Atomic Type	X	Y	Z
1	6	0	-2.629823	0.166187	-0.018842
2	6	0	-2.245031	-1.164683	0.035114
3	6	0	-0.884108	-1.474042	0.061181
4	6	0	0.051506	-0.459784	0.032560
5	6	0	-1.652473	1.148428	-0.047194
6	1	0	-2.985156	-1.956076	0.057335
7	1	0	-0.545669	-2.502509	0.104191
8	1	0	-1.892835	2.203115	-0.086730
9	6	0	1.525246	-0.804734	0.049864
10	1	0	1.678925	-1.875831	0.180380
11	9	0	2.109264	-0.415666	-1.117230
12	9	0	2.156924	-0.140889	1.053398
13	1	0	-3.670882	0.461555	-0.040402
14	7	0	-0.344199	0.837780	-0.022846
15	6	0	0.640320	1.950281	-0.017395
16	1	0	1.014349	2.078901	0.996986
17	1	0	1.453122	1.716735	-0.699339
18	1	0	0.128033	2.848741	-0.349746

4-(Difluoromethyl)-*N*-methylpyridinium Conformer A

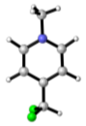

Standard orientation:

Center Number	Atomic Number	Atomic Type	X	Y	Z
1	6	0	-0.157151	1.355230	0.004541
2	6	0	0.662972	0.235533	0.002256
3	6	0	0.094378	-1.039304	-0.001002
4	6	0	-1.279024	-1.155136	-0.002355
5	6	0	-1.532063	1.179624	0.003162
6	1	0	0.704932	-1.934460	-0.002471
7	1	0	-2.227225	2.009086	0.004978
8	1	0	0.245994	2.360869	0.007338
9	7	0	-2.061663	-0.054435	-0.000454
10	6	0	-3.529988	-0.235456	-0.001785
11	1	0	-3.809559	-0.791208	-0.895668
12	1	0	-3.811869	-0.785947	0.894671
13	1	0	-4.001713	0.743499	-0.005433
14	1	0	-1.792067	-2.108775	-0.004956
15	6	0	2.166770	0.374225	0.002646
16	9	0	2.670069	-0.275420	1.089339
17	9	0	2.668514	-0.258585	-1.094925
18	1	0	2.520543	1.405724	0.010201

4-(Difluoromethyl)-*N*-methylpyridinium Conformer B

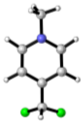

Standard orientation:

Center Number	Atomic Number	Atomic Type	X	Y	Z
1	6	0	-0.025148	1.203139	0.123663
2	6	0	0.664465	-0.002894	0.187940
3	6	0	-0.041460	-1.204468	0.150276
4	6	0	-1.416834	-1.166250	0.042881
5	6	0	-1.404192	1.180560	0.015900
6	1	0	0.462051	-2.162180	0.195542
7	1	0	-1.999827	2.082134	-0.044102
8	1	0	0.489168	2.155667	0.148030
9	7	0	-2.069244	0.012041	-0.023314
10	6	0	-3.542754	-0.003874	-0.153942
11	1	0	-3.802079	-0.483375	-1.096977
12	1	0	-3.959407	-0.559440	0.684621
13	1	0	-3.906465	1.019908	-0.141560
14	1	0	-2.030129	-2.058079	0.001870
15	6	0	2.168199	-0.014570	0.331037
16	9	0	2.677423	-1.095609	-0.320215
17	9	0	2.691290	1.097924	-0.251139
18	1	0	2.499324	-0.049620	1.371426

2-(1,1-Difluoroethyl)-*N*-methylpyridinium Conformer A

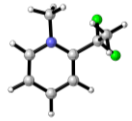

Standard orientation:

Center Number	Atomic Number	Atomic Type	X	Y	Z
1	6	0	-2.821601	-0.291798	0.055829
2	6	0	-2.123438	-1.491194	0.023944
3	6	0	-0.731920	-1.463141	-0.018754
4	6	0	-0.062163	-0.251688	-0.031376
5	6	0	-2.110305	0.892772	0.046085
6	1	0	-2.648083	-2.439558	0.031490
7	1	0	-0.154578	-2.377819	-0.046144
8	1	0	-2.597636	1.858599	0.066516
9	6	0	1.467253	-0.245517	-0.026380
10	9	0	1.865911	-1.436277	-0.562714
11	9	0	1.919736	0.710953	-0.891100
12	1	0	-3.902781	-0.255541	0.088627
13	7	0	-0.761401	0.910653	0.008188
14	6	0	-0.108410	2.245353	-0.039479
15	1	0	0.758464	2.260576	0.613958
16	1	0	0.189476	2.455158	-1.064885
17	1	0	-0.832109	2.978283	0.305380
18	6	0	2.101188	-0.087093	1.332496
19	1	0	1.792769	0.842021	1.812381
20	1	0	1.804943	-0.926686	1.963107
21	1	0	3.184883	-0.087858	1.202386

2-(1,1-Difluoroethyl)-*N*-methylpyridinium Conformer B

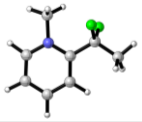

Standard orientation:

Center Number	Atomic Number	Atomic Type	X	Y	Z
1	6	0	-2.825782	-0.338070	-0.000798
2	6	0	-2.120591	-1.530924	0.003283
3	6	0	-0.726221	-1.493298	0.004780
4	6	0	-0.061379	-0.281935	0.003982
5	6	0	-2.119475	0.852269	-0.004649
6	1	0	-2.637728	-2.483353	0.004843
7	1	0	-0.157978	-2.413420	0.007293
8	1	0	-2.613729	1.814946	-0.007471
9	6	0	1.464962	-0.219533	-0.005689
10	9	0	1.848869	0.394107	-1.172237
11	9	0	1.861279	0.616797	1.004651
12	1	0	-3.907664	-0.307955	-0.002312
13	7	0	-0.773591	0.877527	-0.003331
14	6	0	-0.121673	2.214370	0.030069
15	1	0	0.211968	2.413798	1.046669
16	1	0	0.717954	2.227807	-0.657552
17	1	0	-0.861149	2.948369	-0.277313
18	6	0	2.194368	-1.527189	0.126363
19	1	0	1.933190	-2.018119	1.064677
20	1	0	1.973032	-2.180445	-0.718054
21	1	0	3.260646	-1.296596	0.126765

2-(Difluoromethyl)-1,6-dimethylpyridinium Conformer A

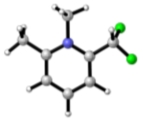

Standard orientation:

Center Number	Atomic Number	Atomic Type	Coordinates (Angstroms) X	Y	Z
1	6	0	-1.964593	1.327552	0.129463
2	6	0	-0.869913	2.171974	0.136419
3	6	0	0.408856	1.629323	0.006391
4	6	0	0.548218	0.264384	-0.107506
5	6	0	-1.795917	-0.051228	0.008315
6	1	0	-1.001124	3.243588	0.232477
7	1	0	1.291726	2.253933	-0.012448
8	6	0	1.925848	-0.357605	-0.260964
9	1	0	2.011701	-1.052653	-1.098482
10	9	0	2.834276	0.629887	-0.428782
11	9	0	2.254281	-1.010468	0.891602
12	1	0	-2.971980	1.715570	0.212980
13	7	0	-0.538670	-0.554787	-0.091467
14	6	0	-0.370747	-2.021856	-0.232756
15	1	0	-0.521146	-2.295278	-1.278646
16	1	0	0.616918	-2.315895	0.104112
17	1	0	-1.101814	-2.521590	0.395504
18	6	0	-2.969601	-0.978189	-0.016666
19	1	0	-2.937881	-1.640379	-0.884839
20	1	0	-3.000980	-1.594671	0.886915
21	1	0	-3.884640	-0.390021	-0.058860

2-(Difluoromethyl)-1,6-dimethylpyridinium Conformer B

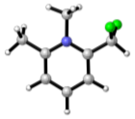

Standard orientation:

Center Number	Atomic Number	Atomic Type	Coordinates (Angstroms) X	Y	Z
1	6	0	2.236736	0.925459	-0.000012
2	6	0	1.423127	2.040154	0.000122
3	6	0	0.037861	1.862284	0.000198
4	6	0	-0.476962	0.587168	0.000103
5	6	0	1.690463	-0.361737	-0.000086
6	1	0	1.849754	3.036430	0.000185
7	1	0	-0.637621	2.708979	0.000317
8	6	0	-1.983545	0.425507	0.000177
9	1	0	-2.473834	1.399024	0.000691
10	9	0	-2.395410	-0.269509	-1.094703
11	9	0	-2.395181	-0.270578	1.094460
12	1	0	3.315101	1.022434	-0.000047
13	7	0	0.341940	-0.503974	-0.000052
14	6	0	-0.270556	-1.857836	-0.000036
15	1	0	-0.879417	-1.965907	0.894647
16	1	0	-0.881074	-1.965182	-0.893666
17	1	0	0.508943	-2.607597	-0.001092
18	6	0	2.584983	-1.563093	-0.000139
19	1	0	2.423045	-2.179103	0.887978
20	1	0	2.423264	-2.178872	-0.888455
21	1	0	3.620942	-1.229035	0.000023

2-(Difluoromethyl)-6-methoxy-*N*-methylpyridinium Conformer A

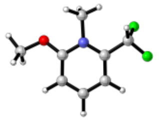

Standard orientation:

Center Number	Atomic Number	Atomic Type	Coordinates (Angstroms) X	Y	Z
1	6	0	-1.564800	1.437673	0.098777
2	6	0	-0.437718	2.228745	0.087770
3	6	0	0.831934	1.647018	-0.035060
4	6	0	0.930595	0.283836	-0.148087
5	6	0	-1.415192	0.050127	-0.020277
6	1	0	-0.533657	3.304533	0.179261
7	1	0	1.730801	2.247460	-0.039756
8	6	0	2.284503	-0.391713	-0.261615
9	1	0	2.421894	-0.972110	-1.176320
10	9	0	3.249454	0.554317	-0.208972
11	9	0	2.462948	-1.210518	0.814317
12	1	0	-2.552522	1.865321	0.198903
13	7	0	-0.183079	-0.504138	-0.153665
14	6	0	-0.126603	-1.979289	-0.291289
15	1	0	0.881698	-2.291600	-0.532593
16	1	0	-0.437287	-2.429204	0.651281
17	1	0	-0.808281	-2.273900	-1.086106
18	8	0	-2.390840	-0.830455	-0.017511
19	6	0	-3.748511	-0.366272	0.101195
20	1	0	-4.354658	-1.267111	0.073454
21	1	0	-3.882747	0.151772	1.052183
22	1	0	-3.993835	0.282497	-0.741151

2-(Difluoromethyl)-6-methoxy-*N*-methylpyridinium Conformer B

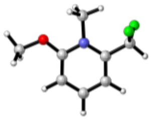

Standard orientation:

Center Number	Atomic Number	Atomic Type	Coordinates (Angstroms) X	Y	Z
1	6	0	-1.731317	1.331700	0.000001
2	6	0	-0.745053	2.290554	0.000010
3	6	0	0.604315	1.906315	0.000018
4	6	0	0.922507	0.572731	0.000006
5	6	0	-1.359915	-0.021265	-0.000009
6	1	0	-1.011432	3.341202	0.000020
7	1	0	1.396576	2.643947	0.000036
8	6	0	2.380209	0.171737	0.000044
9	1	0	3.025695	1.049765	0.000142
10	9	0	2.667428	-0.585293	1.094834
11	9	0	2.667540	-0.585126	-1.094835
12	1	0	-2.778415	1.600975	0.000006
13	7	0	-0.052498	-0.382893	-0.000023
14	6	0	0.233978	-1.838792	-0.000045
15	1	0	-0.216108	-2.274518	-0.890704
16	1	0	1.302931	-2.002057	-0.000302
17	1	0	-0.215690	-2.274476	0.890849
18	8	0	-2.187943	-1.040861	-0.000004
19	6	0	-3.606418	-0.792294	0.000000
20	1	0	-4.061482	-1.778524	-0.000003
21	1	0	-3.887791	-0.244760	0.901041
22	1	0	-3.887796	-0.244751	-0.901033

2-(Difluoromethyl)-1,3-dimethyl-benzoimidazolium Conformer A

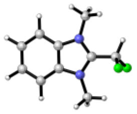

Standard orientation:

Center Number	Atomic Number	Atomic Type	Coordinates (Angstroms) X	Y	Z
1	6	0	2.075479	-1.584725	-0.010169
2	6	0	0.989144	-0.705641	-0.008492
3	6	0	3.331128	-1.001066	-0.001694
4	6	0	1.157336	0.682036	-0.001668
5	6	0	2.424831	1.271614	0.010088
6	1	0	2.560075	2.346714	0.025969
7	6	0	3.502168	0.401450	0.009390
8	1	0	4.507359	0.807518	0.019624
9	1	0	4.209200	-1.637043	-0.001512
10	1	0	1.942169	-2.660313	-0.015342
11	7	0	-0.115097	1.237632	-0.008674
12	7	0	-0.376451	-0.941859	-0.013043
13	6	0	-1.000843	0.237672	-0.005445
14	6	0	-0.375181	2.680296	0.005549
15	1	0	-0.300751	3.054599	1.026470
16	1	0	0.369799	3.158328	-0.628433
17	1	0	-1.358937	2.887892	-0.406740
18	6	0	-2.500026	0.392265	0.026940
19	1	0	-2.825793	1.429049	0.088797
20	9	0	-3.025166	-0.180280	-1.085860
21	9	0	-2.979825	-0.289857	1.097564
22	6	0	-0.979285	-2.277755	-0.021585
23	1	0	-0.732809	-2.780793	0.913685
24	1	0	-2.057131	-2.191295	-0.123451
25	1	0	-0.575940	-2.830717	-0.869866

2-(Difluoromethyl)-1,3-dimethyl-benzoimidazolium Conformer B

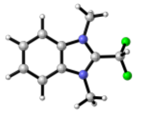

Standard orientation:

Center Number	Atomic Number	Atomic Type	Coordinates (Angstroms) X	Y	Z
1	6	0	-2.247254	-1.448015	-0.074072
2	6	0	-1.068034	-0.703719	0.028387
3	6	0	-3.425213	-0.724026	-0.149505
4	6	0	-1.080954	0.693501	0.048888
5	6	0	-2.269169	1.425411	-0.029108
6	1	0	-2.279394	2.508842	-0.021298
7	6	0	-3.436708	0.687780	-0.126867
8	1	0	-4.385923	1.207565	-0.192391
9	1	0	-4.366041	-1.256404	-0.232714
10	1	0	-2.247620	-2.530906	-0.102357
11	7	0	0.241157	1.102514	0.150828
12	7	0	0.263288	-1.094180	0.119131
13	6	0	1.011398	0.011794	0.170370
14	6	0	0.664893	2.506083	0.212223
15	1	0	0.795033	2.893989	-0.797904
16	1	0	-0.111736	3.061550	0.734590
17	1	0	1.594008	2.587965	0.770110
18	6	0	2.522412	0.059174	0.261644
19	1	0	2.888854	0.220530	1.278095
20	9	0	3.029584	-1.097900	-0.219079
21	9	0	2.962833	1.063348	-0.536094
22	6	0	0.704394	-2.496092	0.152413
23	1	0	1.020729	-2.807664	-0.842206
24	1	0	1.517020	-2.610585	0.864757
25	1	0	-0.142382	-3.093588	0.481922

2-(Difluoromethyl)-*N*-methylquinolinium Conformer A

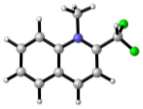

Standard orientation:

Center Number	Atomic Number	Atomic Type	Coordinates (Angstroms) X	Y	Z
1	6	0	-1.398483	0.973678	0.014967
2	6	0	-0.401422	1.967514	0.004833
3	6	0	0.925086	1.613730	-0.073582
4	6	0	1.267868	0.261898	-0.153595
5	6	0	-1.010418	-0.390786	-0.065374
6	1	0	1.712349	2.354347	-0.076401
7	7	0	0.337142	-0.700292	-0.163751
8	6	0	0.697413	-2.131620	-0.283174
9	1	0	0.163962	-2.550551	-1.134739
10	1	0	1.762113	-2.247900	-0.439607
11	1	0	0.418412	-2.642443	0.638450
12	6	0	2.736708	-0.133838	-0.229879
13	9	0	3.494356	0.984923	-0.199056
14	9	0	3.053784	-0.865628	0.874384
15	1	0	2.998297	-0.700672	-1.125580
16	6	0	-2.776672	1.302107	0.107817
17	6	0	-1.997602	-1.402196	-0.039159
18	6	0	-3.322521	-1.043853	0.055233
19	1	0	-4.075546	-1.823995	0.077852
20	6	0	-3.722816	0.311644	0.126028
21	1	0	-4.775910	0.557994	0.197774
22	1	0	-1.736050	-2.449768	-0.084177
23	1	0	-3.054265	2.349325	0.165396
24	1	0	-0.689465	3.012380	0.064645

2-(Difluoromethyl)-*N*-methylquinolinium Conformer B

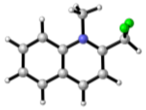

Standard orientation:

Center Number	Atomic Number	Atomic Type	Coordinates (Angstroms) X	Y	Z
1	6	0	1.494514	0.936276	-0.000057
2	6	0	0.630556	2.046347	-0.000296
3	6	0	-0.731681	1.852818	-0.000376
4	6	0	-1.242818	0.553955	-0.000242
5	6	0	0.937383	-0.372657	-0.000142
6	1	0	-1.420285	2.688300	-0.000689
7	7	0	-0.440115	-0.517900	-0.000493
8	6	0	-0.979115	-1.898215	-0.001294
9	1	0	-0.626643	-2.406321	0.895503
10	1	0	-2.059982	-1.881014	-0.002621
11	1	0	-0.624885	-2.405741	-0.897728
12	6	0	-2.754306	0.403803	0.000751
13	9	0	-3.164757	-0.288440	-1.095122
14	9	0	-3.162660	-0.290305	1.096416
15	1	0	-3.239920	1.379293	0.002167
16	6	0	2.906316	1.093873	0.000319
17	6	0	1.795564	-1.496995	-0.000029
18	6	0	3.157008	-1.302227	0.000207
19	1	0	3.809626	-2.168356	0.000123
20	6	0	3.723625	-0.004728	0.000345
21	1	0	4.801272	0.111224	0.000411
22	1	0	1.406576	-2.505340	-0.000411
23	1	0	3.312869	2.099782	0.000253
24	1	0	1.046654	3.048678	-0.000306

4-(Difluoromethyl)-*N*-methylquinolinium Conformer A

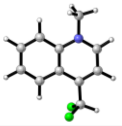

Standard orientation:

Center Number	Atomic Number	Atomic Type	Coordinates (Angstroms) X	Y	Z
1	6	0	-0.146134	0.343241	0.000126
2	6	0	-0.987905	-0.803383	0.000036
3	6	0	-0.440543	-2.060186	-0.000011
4	6	0	0.952584	-2.200198	-0.000010
5	6	0	1.262464	0.146559	0.000115
6	1	0	-1.051351	-2.954577	-0.000125
7	7	0	1.758292	-1.147994	-0.000078
8	6	0	3.218539	-1.367690	0.000023
9	1	0	3.646733	-0.919753	0.896231
10	1	0	3.402364	-2.438268	-0.000048
11	1	0	3.646877	-0.919639	-0.896004
12	1	0	1.423338	-3.175187	-0.000039
13	6	0	-2.493959	-0.674618	-0.000002
14	9	0	-2.896621	0.032001	1.094125
15	9	0	-2.896548	0.031847	-1.094283
16	1	0	-3.003100	-1.639138	0.000050
17	6	0	-0.645666	1.672587	0.000323
18	6	0	2.138953	1.252793	0.000113
19	6	0	1.614897	2.524389	-0.000174
20	1	0	2.287962	3.374486	-0.000514
21	6	0	0.218676	2.738224	-0.000079
22	1	0	-0.169814	3.750172	-0.000289
23	1	0	3.211674	1.115252	-0.000259
24	1	0	-1.715630	1.837678	0.000198

4-(Difluoromethyl)-*N*-methylquinolinium Conformer B

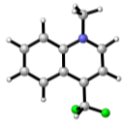

Standard orientation:

Center	Atomic	Atomic	Coordinates (Angstroms)		
Number	Number	Type	X	Y	Z
1	6	0	-0.021111	0.504374	-0.102840
2	6	0	1.111722	-0.353724	-0.142169
3	6	0	0.947831	-1.714315	-0.090099
4	6	0	-0.345089	-2.239809	0.010133
5	6	0	-1.312817	-0.081539	-0.007069
6	1	0	1.788985	-2.393443	-0.124954
7	7	0	-1.418051	-1.461530	0.048220
8	6	0	-2.752130	-2.086517	0.146457
9	1	0	-3.340551	-1.810322	-0.728168
10	1	0	-2.622645	-3.164634	0.176103
11	1	0	-3.239519	-1.748166	1.060285
12	1	0	-0.517213	-3.307567	0.059428
13	6	0	2.500336	0.236230	-0.259899
14	9	0	2.749345	1.014858	0.833677
15	9	0	3.428389	-0.753954	-0.260693
16	1	0	2.641315	0.841709	-1.158356
17	6	0	0.078798	1.919607	-0.162723
18	6	0	-2.465718	0.732061	0.028620
19	6	0	-2.325838	2.098733	-0.033543
20	1	0	-3.211663	2.723445	-0.007455
21	6	0	-1.050494	2.698237	-0.131405
22	1	0	-0.966189	3.777780	-0.179796
23	1	0	-3.453084	0.297054	0.102126
24	1	0	1.054367	2.386684	-0.226387

***N*-Methyl-9-(difluoromethyl)acridinium**

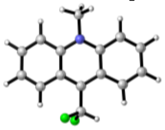

Standard orientation:

Center	Atomic	Atomic	Coordinates (Angstroms)		
Number	Number	Type	X	Y	Z
1	6	0	-1.219369	0.575832	-0.001811
2	6	0	0.115878	1.001835	0.043428
3	6	0	1.167126	0.072155	-0.008895
4	6	0	0.850686	-1.323918	-0.002290
5	6	0	-1.488268	-0.834130	0.014434
6	7	0	-0.453582	-1.720012	0.103855
7	6	0	-0.751370	-3.149181	0.329618
8	1	0	-0.877860	-3.665747	-0.623693
9	1	0	0.059753	-3.590999	0.898610
10	1	0	-1.651032	-3.230139	0.930754
11	6	0	0.448084	2.481374	0.136910
12	9	0	1.272609	2.689476	1.202466
13	9	0	1.128729	2.866832	-0.980079
14	1	0	-0.408113	3.137978	0.254659
15	6	0	-2.333657	1.475138	-0.070832
16	6	0	-2.830806	-1.291720	-0.070701
17	6	0	-3.857418	-0.389325	-0.147274
18	1	0	-4.875132	-0.754325	-0.231907
19	6	0	-3.612976	1.008203	-0.136972
20	1	0	-4.443030	1.701925	-0.199702
21	1	0	-3.055061	-2.347087	-0.122286
22	1	0	-2.172051	2.543583	-0.097458
23	6	0	1.896788	-2.278320	-0.112662
24	6	0	2.544706	0.459825	-0.076081
25	6	0	3.531269	-0.478599	-0.162251
26	1	0	4.569469	-0.173803	-0.223033
27	6	0	3.197469	-1.857041	-0.194996
28	1	0	3.984469	-2.596087	-0.299029
29	1	0	1.681986	-3.334913	-0.177608
30	1	0	2.800789	1.510154	-0.075523

Neutral species:

Trimethylphospine oxide

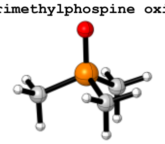

Standard orientation:

Center	Atomic	Atomic	Coordinates (Angstroms)		
Number	Number	Type	X	Y	Z
1	15	0	0.000364	-0.000274	0.166652
2	8	0	0.004075	-0.001711	1.681035
3	6	0	1.585333	-0.515986	-0.548355
4	1	0	1.819274	-1.528039	-0.209578
5	1	0	1.542247	-0.500629	-1.640300
6	1	0	2.369604	0.164205	-0.207952
7	6	0	-0.346830	1.631904	-0.542936
8	1	0	-0.345472	1.586803	-1.634860
9	1	0	-1.325452	1.971685	-0.196122
10	1	0	0.415604	2.339550	-0.208752
11	6	0	-1.242335	-1.114134	-0.543533
12	1	0	-1.214806	-1.078753	-1.635537
13	1	0	-1.042219	-2.134687	-0.209063
14	1	0	-2.233852	-0.813030	-0.196947

Phenol

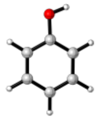

Standard orientation:

Center	Atomic	Atomic	Coordinates (Angstroms)		
Number	Number	Type	X	Y	Z
1	6	0	-0.219392	-1.222792	0.000155
2	6	0	-0.937958	-0.025409	0.000464
3	6	0	-0.267519	1.199362	0.000106
4	6	0	1.126877	1.219441	-0.000033
5	6	0	1.172643	-1.188410	-0.000101
6	1	0	-0.832847	2.127432	-0.000061
7	1	0	1.726739	-2.122001	-0.000241
8	1	0	-0.760766	-2.163342	0.000178
9	1	0	1.642649	2.174680	-0.000136
10	6	0	1.854888	0.029982	-0.000083
11	1	0	2.939350	0.051463	-0.000161
12	8	0	-2.299777	-0.113664	-0.000326
13	1	0	-2.694141	0.768044	-0.000018

2-(Difluoromethyl)pyridine Conformer A

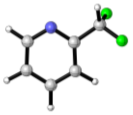

Standard orientation:

Center	Atomic	Atomic	Coordinates (Angstroms)		
Number	Number	Type	X	Y	Z
1	6	0	-2.553663	0.057618	0.103226
2	6	0	-1.811183	1.224860	0.245992
3	6	0	-0.425498	1.161676	0.109709
4	6	0	0.142963	-0.077937	-0.164814
5	6	0	-1.880270	-1.132620	-0.174867
6	1	0	-2.299208	2.170060	0.458997
7	1	0	0.196665	2.043274	0.209549
8	1	0	-2.430026	-2.061585	-0.297411
9	6	0	1.634444	-0.258257	-0.293146
10	1	0	1.918849	-0.911569	-1.119586
11	9	0	2.257032	0.946028	-0.445331
12	9	0	2.130546	-0.801796	0.864597
13	1	0	-3.633446	0.060858	0.199181
14	7	0	-0.554542	-1.207311	-0.311532

2-(Difluoromethyl)pyridine Conformer B

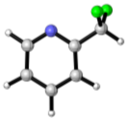

Standard orientation:

Center	Atomic	Atomic	Coordinates (Angstroms)		
Number	Number	Type	X	Y	Z
1	6	0	-2.530507	-0.284988	0.000019
2	6	0	-2.098358	1.035103	-0.000027
3	6	0	-0.726313	1.288640	-0.000050
4	6	0	0.139193	0.201064	-0.000020
5	6	0	-1.573196	-1.303362	0.000033
6	1	0	-2.810167	1.853659	-0.000042
7	1	0	-0.340465	2.302696	-0.000088
8	1	0	-1.880002	-2.345675	0.000078
9	6	0	1.630634	0.416537	-0.000032
10	1	0	1.923013	1.468077	-0.000118
11	9	0	2.192550	-0.185695	1.092143
12	9	0	2.192566	-0.185883	-1.092094
13	1	0	-3.585867	-0.532896	0.000044
14	7	0	-0.260183	-1.074233	0.000021

4-(Difluoromethyl)pyridine Conformer A

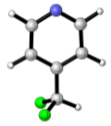

Standard orientation:

Center	Atomic	Atomic	Coordinates (Angstroms)		
Number	Number	Type	X	Y	Z
1	6	0	-0.350738	-1.094932	0.009494
2	6	0	0.160788	0.201938	-0.010667
3	6	0	-0.719798	1.276110	-0.021967
4	6	0	-2.089325	1.005723	-0.011898
5	6	0	-1.732512	-1.253898	0.017836
6	1	0	-0.363297	2.301001	-0.038283
7	1	0	-2.167786	-2.249066	0.033341
8	1	0	0.304817	-1.959382	0.018168
9	7	0	-2.595376	-0.229229	0.007632
10	1	0	-2.805992	1.822183	-0.020137
11	6	0	1.646183	0.427829	-0.015325
12	9	0	2.205375	-0.132327	1.102106
13	9	0	2.214099	-0.219393	-1.079402
14	1	0	1.937027	1.478725	-0.055690

4-(Difluoromethyl)pyridine Conformer B

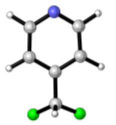

Standard orientation:

Center Number	Atomic Number	Atomic Type	Coordinates (Angstroms) X	Y	Z
1	6	0	0.534980	1.199453	0.095457
2	6	0	-0.168124	0.000000	0.164357
3	6	0	0.534979	-1.199452	0.095457
4	6	0	1.919873	-1.142547	-0.046952
5	6	0	1.919873	1.142547	-0.046950
6	1	0	0.025834	-2.155413	0.143296
7	1	0	2.497934	2.060370	-0.108947
8	1	0	0.025835	2.155413	0.143296
9	7	0	2.610763	-0.000000	-0.117610
10	1	0	2.497933	-2.060370	-0.108948
11	6	0	-1.661365	0.000001	0.356825
12	9	0	-2.212942	1.097264	-0.242166
13	9	0	-2.212942	-1.097264	-0.242163
14	1	0	-1.971213	0.000002	1.404372

2-(Difluoromethyl)nitrobenzene Conformer A

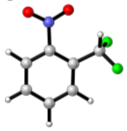

Standard orientation:

Center Number	Atomic Number	Atomic Type	Coordinates (Angstroms) X	Y	Z
1	6	0	1.955901	-1.928958	0.165197
2	6	0	0.721791	-2.570766	0.108030
3	6	0	-0.451622	-1.830705	-0.028719
4	6	0	-0.422949	-0.438039	-0.094174
5	6	0	2.015051	-0.542000	0.087137
6	1	0	0.666833	-3.652349	0.164960
7	1	0	-1.406306	-2.338708	-0.093698
8	1	0	2.959374	-0.013751	0.126434
9	6	0	-1.737076	0.302767	-0.233323
10	1	0	-1.713432	1.162784	-0.898422
11	9	0	-2.693360	-0.558434	-0.694131
12	9	0	-2.169623	0.711053	0.998239
13	1	0	2.870934	-2.500724	0.269300
14	7	0	0.961724	1.643647	-0.082412
15	8	0	2.053216	2.114760	-0.348289
16	8	0	-0.031839	2.313774	0.152525
17	6	0	0.833298	0.180262	-0.031241

2-(Difluoromethyl)nitrobenzene Conformer B

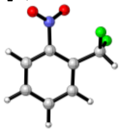

Standard orientation:

Center Number	Atomic Number	Atomic Type	Coordinates (Angstroms) X	Y	Z
1	6	0	2.794439	0.325322	0.096463
2	6	0	2.623050	-1.051809	-0.000215
3	6	0	1.340457	-1.591922	-0.100988
4	6	0	0.210131	-0.778212	-0.071945
5	6	0	1.681797	1.164080	0.100996
6	1	0	3.484901	-1.709568	-0.014186
7	1	0	1.215007	-2.664225	-0.214957
8	1	0	1.784973	2.240625	0.170483
9	6	0	-1.129004	-1.451874	-0.244006
10	1	0	-1.014603	-2.458517	-0.650375
11	9	0	-1.800624	-1.558196	0.939033
12	9	0	-1.927972	-0.743955	-1.096334
13	1	0	3.788054	0.753381	0.164276
14	7	0	-0.716764	1.537132	0.106327
15	8	0	-0.600642	2.605645	-0.470710
16	8	0	-1.687081	1.206111	0.761602
17	6	0	0.415492	0.601696	0.034534

Difluoromethyl phenyl sulfone Conformer A

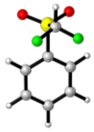

Standard orientation:

Center Number	Atomic Number	Atomic Type	Coordinates (Angstroms) X	Y	Z
1	6	0	3.299278	-0.000167	0.310496
2	6	0	2.629723	-1.213818	0.145104
3	6	0	1.278334	-1.223815	-0.189568
4	6	0	0.629455	-0.000267	-0.352643
5	6	0	2.629968	1.213438	0.143833
6	1	0	3.159220	-2.151551	0.272311
7	1	0	0.737801	-2.153797	-0.331222
8	1	0	3.159639	2.151207	0.270035
9	1	0	4.353006	-0.000140	0.569342
10	6	0	1.278597	1.223343	-0.190870
11	1	0	0.738306	2.153320	-0.333503
12	16	0	-1.093105	-0.000355	-0.752230
13	6	0	-1.921277	0.001272	0.896126
14	1	0	-3.005417	0.003015	0.761861
15	9	0	-1.516070	-1.091364	1.568609
16	9	0	-1.512713	1.093193	1.567843
17	8	0	-1.458586	1.271363	-1.374539
18	8	0	-1.458701	-1.272958	-1.372471

Difluoromethyl phenyl sulfone Conformer B

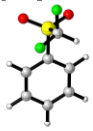

Standard orientation:

Center Number	Atomic Number	Atomic Type	Coordinates (Angstroms) X	Y	Z
1	6	0	-3.443727	-0.249974	-0.304989
2	6	0	-2.724755	-1.168001	0.462747
3	6	0	-1.375302	-0.950550	0.725943
4	6	0	-0.777951	0.197914	0.207127
5	6	0	-2.826256	0.893022	-0.815206
6	1	0	-3.214434	-2.050546	0.859183
7	1	0	-0.796897	-1.645432	1.325643
8	1	0	-3.393987	1.603661	-1.405533
9	1	0	-4.495598	-0.425647	-0.504984
10	6	0	-1.477409	1.127963	-0.561420
11	1	0	-0.978605	2.014973	-0.938192
12	16	0	0.943139	0.469963	0.508617
13	6	0	1.762870	-0.347675	-0.932708
14	1	0	1.477483	0.128640	-1.872897
15	9	0	3.087560	-0.248753	-0.723581
16	9	0	1.421526	-1.648786	-0.923008
17	8	0	1.253962	1.892857	0.372726
18	8	0	1.359190	-0.278282	1.693431

2-(Difluoromethyl)-1-methyl-benzoimidazole Conformer A

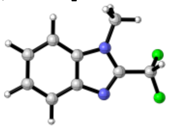

Standard orientation:

Center Number	Atomic Number	Atomic Type	Coordinates (Angstroms) X	Y	Z
1	6	0	2.178340	-1.600367	0.072052
2	6	0	0.977588	-0.883123	-0.022359
3	6	0	3.359085	-0.875367	0.129077
4	6	0	0.998351	0.525418	-0.059770
5	6	0	2.183507	1.265820	0.001059
6	1	0	2.186127	2.350601	-0.020746
7	6	0	3.360707	0.537012	0.094664
8	1	0	4.307557	1.064739	0.144514
9	1	0	4.305978	-1.400005	0.203495
10	1	0	2.173616	-2.685191	0.100227
11	7	0	-0.321981	0.910334	-0.158420
12	7	0	-0.328446	-1.338133	-0.089738
13	6	0	-1.047571	-0.247204	-0.161798
14	6	0	-0.794895	2.286327	-0.203727
15	1	0	-0.677055	2.757689	0.774357
16	1	0	-0.220155	2.836921	-0.950175
17	1	0	-1.846578	2.306959	-0.484183
18	6	0	-2.547052	-0.224231	-0.259716
19	1	0	-2.929897	0.243380	-1.170086
20	9	0	-3.027146	-1.487459	-0.187431
21	9	0	-3.056739	0.466768	0.809966

2-(Difluoromethyl)-1-methyl-benzoimidazole Conformer B

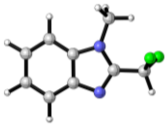

Standard orientation:

Center Number	Atomic Number	Atomic Type	Coordinates (Angstroms)		
			X	Y	Z
1	6	0	2.378687	-1.477362	-0.000032
2	6	0	1.095896	-0.910776	-0.000015
3	6	0	3.463458	-0.614330	-0.000015
4	6	0	0.942753	0.490735	0.000029
5	6	0	2.031736	1.369477	0.000051
6	1	0	1.901076	2.446585	0.000067
7	6	0	3.291437	0.788564	0.000025
8	1	0	4.168855	1.427046	0.000031
9	1	0	4.469846	-1.019919	-0.000030
10	1	0	2.507311	-2.554900	-0.000057
11	7	0	-0.416662	0.715797	0.000047
12	7	0	-0.147374	-1.518134	-0.000026
13	6	0	-0.996729	-0.519712	0.000015
14	6	0	-1.051789	2.026394	-0.000072
15	1	0	-0.750638	2.578745	0.892328
16	1	0	-0.751786	2.578094	-0.893265
17	1	0	-2.132762	1.905962	0.000678
18	6	0	-2.475893	-0.733114	-0.000007
19	1	0	-2.724213	-1.794049	-0.000023
20	9	0	-3.045393	-0.137274	1.092159
21	9	0	-3.045360	-0.137222	-1.092131

2-(Difluoromethyl)quinoline Conformer A

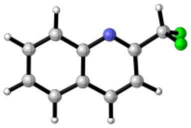

Standard orientation:

Center Number	Atomic Number	Atomic Type	Coordinates (Angstroms)		
			X	Y	Z
1	6	0	1.291210	0.811254	0.000000
2	6	0	0.230942	1.755770	-0.000000
3	6	0	-1.064408	1.312998	-0.000000
4	6	0	-1.289703	-0.088101	0.000000
5	6	0	0.950966	-0.570364	0.000000
6	1	0	-1.905515	1.997602	-0.000000
7	7	0	-0.346655	-0.998002	0.000000
8	6	0	-2.701920	-0.611611	0.000000
9	9	0	-3.377761	-0.133610	1.091171
10	9	0	-3.377761	-0.133611	-1.091171
11	1	0	-2.750152	-1.700769	0.000000
12	6	0	2.660083	1.187927	0.000000
13	6	0	1.984720	-1.542940	0.000000
14	6	0	3.300552	-1.149382	-0.000000
15	1	0	4.089616	-1.894224	-0.000000
16	6	0	3.642128	0.227631	0.000000
17	1	0	4.687316	0.518713	0.000000
18	1	0	1.702878	-2.591004	0.000000
19	1	0	2.913974	2.244133	0.000000
20	1	0	0.460736	2.817465	-0.000000

2-(Difluoromethyl)quinoline Conformer B

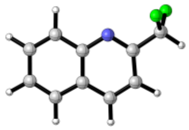

Standard orientation:

Center Number	Atomic Number	Atomic Type	Coordinates (Angstroms)		
			X	Y	Z
1	6	0	1.359212	0.833736	0.000000
2	6	0	0.467825	1.933532	0.000000
3	6	0	-0.885170	1.702721	0.000000
4	6	0	-1.333322	0.360824	0.000000
5	6	0	0.799059	-0.476400	0.000000
6	1	0	-1.600449	2.518100	0.000001
7	7	0	-0.545506	-0.689009	0.000000
8	6	0	-2.816226	0.070384	0.000000
9	9	0	-3.146097	-0.682928	-1.092219
10	9	0	-3.146097	-0.682931	1.092218
11	1	0	-3.440285	0.965695	0.000002
12	6	0	2.772806	0.985559	0.000000
13	6	0	1.666328	-1.603121	-0.000000
14	6	0	3.026630	-1.424997	-0.000000
15	1	0	3.686195	-2.286693	-0.000001
16	6	0	3.586480	-0.119033	-0.000000
17	1	0	4.664982	-0.000709	-0.000000
18	1	0	1.220080	-2.592440	-0.000001
19	1	0	3.191781	1.987769	0.000000
20	1	0	0.864255	2.944840	0.000000

4-(Difluoromethyl)quinoline Conformer A

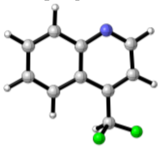

Standard orientation:

Center Number	Atomic Number	Atomic Type	Coordinates (Angstroms)		
			X	Y	Z
1	6	0	-0.375222	-0.188552	-0.089795
2	6	0	0.982957	0.244484	-0.125537
3	6	0	1.266026	1.581029	-0.042078
4	6	0	0.191561	2.499606	0.087899
5	6	0	-1.372228	0.819168	0.037568
6	1	0	2.286282	1.943699	-0.076485
7	7	0	-1.071411	2.149907	0.126970
8	1	0	0.408257	3.562688	0.159599
9	6	0	2.083718	-0.772018	-0.275400
10	9	0	3.301374	-0.162420	-0.310405
11	9	0	2.098263	-1.602199	0.816923
12	1	0	1.992885	-1.396396	-1.167269
13	6	0	-0.777452	-1.549150	-0.179226
14	6	0	-2.740787	0.443417	0.073441
15	6	0	-3.101386	-0.878042	-0.017304
16	1	0	-4.148866	-1.159792	0.009936
17	6	0	-2.109981	-1.882281	-0.146656
18	1	0	-2.407148	-2.923354	-0.217411
19	1	0	-3.480730	1.231041	0.173532
20	1	0	-0.030760	-2.331617	-0.266827

4-(Difluoromethyl)quinoline Conformer B

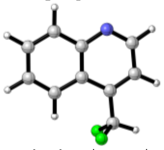

Standard orientation:

Center Number	Atomic Number	Atomic Type	Coordinates (Angstroms)		
			X	Y	Z
1	6	0	0.242274	-0.124301	0.000000
2	6	0	-1.010826	0.558343	-0.000000
3	6	0	-1.032732	1.926983	-0.000001
4	6	0	0.198131	2.634847	-0.000001
5	6	0	1.413982	0.684617	0.000001
6	1	0	-1.968524	2.476414	-0.000001
7	7	0	1.371806	2.052038	-0.000000
8	1	0	0.188368	3.721961	-0.000001
9	6	0	-2.314342	-0.188861	-0.000001
10	9	0	-2.397279	-1.010023	-1.093348
11	9	0	-2.397281	-1.010020	1.093349
12	1	0	-3.185862	0.467494	-0.000003
13	6	0	0.380976	-1.538997	0.000000
14	6	0	2.689981	0.062587	0.000001
15	6	0	2.795086	-1.306033	-0.000000
16	1	0	3.772590	-1.777076	-0.000000
17	6	0	1.629691	-2.112086	-0.000000
18	1	0	1.726019	-3.192805	-0.000001
19	1	0	3.566694	0.702438	0.000001
20	1	0	-0.504213	-2.164900	0.000000

9-(Difluoromethyl)acridine

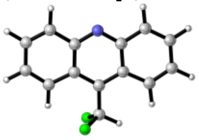

Standard orientation:

Center Number	Atomic Number	Atomic Type	Coordinates (Angstroms)		
			X	Y	Z
1	6	0	-1.294684	0.253641	0.000034
2	6	0	0.001540	0.793015	0.000031
3	6	0	1.119494	-0.060298	-0.000008
4	6	0	0.874650	-1.475810	0.000179
5	6	0	-1.411710	-1.183381	0.000056
6	7	0	-0.356404	-2.003224	0.000195
7	6	0	0.212262	2.285927	-0.000022
8	9	0	0.945316	2.660739	1.095626
9	9	0	0.945697	2.660556	-1.095481
10	1	0	-0.698040	2.879215	-0.000169
11	6	0	-2.507087	1.025153	0.000067
12	6	0	-2.713838	-1.783288	-0.000142
13	6	0	-3.836162	-1.011066	-0.000238
14	1	0	-4.819045	-1.470551	-0.000473
15	6	0	-3.726667	0.413382	-0.000027
16	1	0	-4.628528	1.016391	0.000074
17	1	0	-2.761842	-2.867321	-0.000396
18	1	0	-2.473185	2.107430	0.000315
19	6	0	1.985648	-2.379857	0.000253
20	6	0	2.480399	0.394516	-0.000274
21	6	0	3.511851	-0.500397	-0.000213
22	1	0	4.535456	-0.140577	-0.000393
23	6	0	3.264622	-1.906909	0.000050
24	1	0	4.103084	-2.595710	0.000048
25	1	0	1.765806	-3.442466	0.000280
26	1	0	2.690096	1.456737	-0.000437

Hydrogen bonding complexes with cationic donors:

2-(Difluoromethyl)-*N*-methylpyridinium Conformer A – Me_3PO Complex

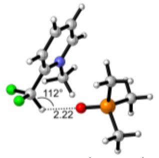

Standard orientation:

Center Number	Atomic Number	Atomic Type	Coordinates (Angstroms) X	Y	Z
1	6	0	1.425943	2.658698	-0.325010
2	6	0	1.656583	1.923970	-1.482024
3	6	0	1.729712	0.534805	-1.404183
4	6	0	1.572418	-0.086976	-0.180107
5	6	0	1.278655	1.987262	0.875825
6	1	0	1.777051	2.422778	-2.436930
7	1	0	1.901707	-0.073666	-2.282418
8	1	0	1.097421	2.499165	1.812163
9	6	0	1.681224	-1.592242	-0.034017
10	1	0	0.871529	-2.033731	0.543513
11	9	0	1.700619	-2.149329	-1.268612
12	9	0	2.881885	-1.882599	0.554293
13	1	0	1.359382	3.738970	-0.335537
14	7	0	1.355625	0.644561	0.937160
15	6	0	1.136862	-0.029827	2.236796
16	1	0	0.212024	-0.603677	2.154388
17	1	0	1.988622	-0.671093	2.457614
18	1	0	1.042599	0.733578	3.004169
19	8	0	-1.053386	-0.968358	0.229338
20	15	0	-2.343970	-0.212623	-0.036538
21	6	0	-2.182398	1.000216	-1.372677
22	1	0	-3.140863	1.488036	-1.567805
23	1	0	-1.448149	1.757929	-1.086381
24	1	0	-1.839963	0.493572	-2.278288
25	6	0	-3.708187	-1.304758	-0.508883
26	1	0	-3.875263	-2.030817	0.290224
27	1	0	-4.623113	-0.731099	-0.675193
28	1	0	-3.440494	-1.836192	-1.425196
29	6	0	-2.916040	0.716695	1.409065
30	1	0	-3.835344	1.261151	1.179171
31	1	0	-3.100912	0.022779	2.232509
32	1	0	-2.140147	1.426897	1.707404

2-(Difluoromethyl)-*N*-methylpyridinium Conformer B – Me_3PO Complex

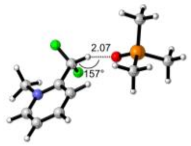

Standard orientation:

Center Number	Atomic Number	Atomic Type	Coordinates (Angstroms) X	Y	Z
1	6	0	-2.864271	2.499892	0.009011
2	6	0	-1.485580	2.350998	0.051520
3	6	0	-0.940887	1.065759	0.054484
4	6	0	-1.781372	-0.030080	0.015449
5	6	0	-3.662567	1.367771	-0.030212
6	1	0	-0.837117	3.219242	0.082374
7	1	0	0.130830	0.882080	0.086713
8	1	0	-4.743024	1.420817	-0.067380
9	6	0	-1.173332	-1.415770	0.032343
10	1	0	-0.083657	-1.361720	-0.011434
11	9	0	-1.549204	-2.073143	1.170060
12	9	0	-1.642297	-2.149967	-1.017023
13	1	0	-3.336807	3.473749	0.005660
14	7	0	-3.128458	0.133330	-0.025798
15	6	0	-4.047058	-1.029797	-0.107098
16	1	0	-4.027101	-1.419728	-1.123550
17	1	0	-3.728015	-1.790769	0.599763
18	1	0	-5.045343	-0.684571	0.147583
19	8	0	1.781669	-0.466151	0.083752
20	15	0	3.213084	0.035754	-0.011809
21	6	0	3.298203	1.827887	-0.262883
22	1	0	4.336774	2.161598	-0.327371
23	1	0	2.776617	2.084849	-1.188216
24	1	0	2.807977	2.329479	0.575369
25	6	0	4.177168	-0.309400	1.481324
26	1	0	4.202900	-1.388638	1.649160
27	1	0	5.197908	0.065949	1.374871
28	1	0	3.699501	0.174791	2.336317
29	6	0	4.128872	-0.710749	-1.383826
30	1	0	5.150520	-0.324501	-1.418988
31	1	0	4.155428	-1.794519	-1.248244
32	1	0	3.620647	-0.479593	-2.322928

4-(Difluoromethyl)-*N*-methylpyridinium Conformer A – Me_3PO Complex

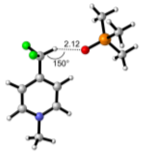

Standard orientation:

Center Number	Atomic Number	Atomic Type	Coordinates (Angstroms) X	Y	Z
1	6	0	-1.241387	-0.578200	0.029230
2	6	0	-1.689593	0.736609	0.014228
3	6	0	-3.059039	1.007155	-0.008310
4	6	0	-3.944323	-0.049070	-0.015552
5	6	0	-2.178796	-1.599242	0.020952
6	1	0	-3.439334	2.021767	-0.019969
7	1	0	-1.904069	-2.646228	0.031491
8	1	0	-0.176746	-0.800250	0.047122
9	7	0	-3.494099	-1.322643	-0.001037
10	6	0	-4.487895	-2.417739	-0.009753
11	1	0	-5.116545	-2.323689	0.874584
12	1	0	-5.088126	-2.335121	-0.914770
13	1	0	-3.961852	-3.368475	0.004799
14	1	0	-5.019634	0.078775	-0.032933
15	6	0	-0.697449	1.874907	0.017883
16	9	0	-0.894741	2.631395	-1.104275
17	9	0	-0.968388	2.692388	1.079738
18	1	0	0.342435	1.545421	0.060765
19	15	0	3.235978	-0.446929	0.005381
20	8	0	1.783211	-0.008064	0.080120
21	6	0	3.411143	-2.232781	-0.241905
22	1	0	2.916507	-2.515576	-1.174206
23	1	0	4.465792	-2.514501	-0.291174
24	1	0	2.934499	-2.757294	0.589684
25	6	0	4.164813	-0.056610	1.510535
26	1	0	5.202893	-0.385746	1.418502
27	1	0	4.139770	1.022962	1.676496
28	1	0	3.698310	-0.560424	2.360351
29	6	0	4.136987	0.339056	-1.355105
30	1	0	5.174816	-0.002457	-1.377070
31	1	0	3.651354	0.086273	-2.300608
32	1	0	4.114662	1.422964	-1.219869

4-(Difluoromethyl)-*N*-methylpyridinium Conformer B – Me_3PO Complex

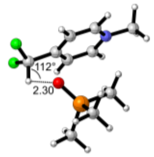

Standard orientation:

Center Number	Atomic Number	Atomic Type	Coordinates (Angstroms) X	Y	Z
1	6	0	-1.852960	0.398404	1.120966
2	6	0	-1.261494	0.919268	-0.024851
3	6	0	-1.216860	0.149766	-1.186683
4	6	0	-1.760648	-1.117012	-1.169086
5	6	0	-2.375535	-0.883196	1.084447
6	1	0	-0.750828	0.522856	-2.088787
7	1	0	-2.842635	-1.348895	1.942749
8	1	0	-1.914551	0.968559	2.039831
9	7	0	-2.317997	-1.612879	-0.043486
10	6	0	-2.836209	-2.997447	-0.061604
11	1	0	-3.486980	-3.115667	-0.926183
12	1	0	-1.989712	-3.681291	-0.123790
13	1	0	-3.396062	-3.174228	0.852938
14	1	0	-1.758254	-1.772179	-2.031762
15	6	0	-0.641256	2.294950	0.003938
16	9	0	-0.666860	2.835148	-1.245130
17	9	0	-1.385796	3.108529	0.809797
18	1	0	0.390710	2.277032	0.354647
19	15	0	2.367830	-0.391800	0.026729
20	8	0	1.644738	0.696801	-0.742671
21	6	0	1.322898	-1.839545	0.358139
22	1	0	0.977506	-2.263451	-0.588470
23	1	0	1.882019	-2.600139	0.908943
24	1	0	0.461638	-1.532235	0.959169
25	6	0	2.948276	0.167054	1.649666
26	1	0	3.462385	-0.638346	2.180000
27	1	0	3.633322	1.007372	1.513895
28	1	0	2.089034	0.499213	2.238596
29	6	0	3.825200	-1.026004	-0.841315
30	1	0	4.315553	-1.807212	-0.255421
31	1	0	3.517417	-1.434888	-1.806634
32	1	0	4.525500	-0.204277	-1.008597

2-(1,1-Difluoroethyl)-*N*-methylpyridinium Conformer A – Me_3PO Complex

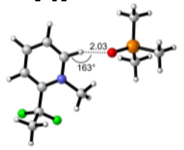

Standard orientation:

Center Number	Atomic Number	Atomic Type	Coordinates (Angstroms) X	Y	Z
1	6	0	0.773090	2.540899	-0.180797
2	6	0	2.130492	2.721047	0.041589
3	6	0	2.954886	1.602022	0.141169
4	6	0	2.415438	0.333188	0.024855
5	6	0	0.274824	1.254669	-0.297295
6	1	0	2.551522	3.715464	0.135141
7	1	0	4.018601	1.703276	0.311525
8	1	0	-0.776110	1.033526	-0.481387
9	6	0	3.344482	-0.879315	0.078946
10	9	0	4.451777	-0.496635	0.782513
11	9	0	2.765739	-1.866119	0.826847
12	1	0	0.091095	3.376951	-0.269126
13	7	0	1.085420	0.179305	-0.196634
14	6	0	0.429697	-1.152026	-0.300203
15	1	0	1.007706	-1.800673	-0.952918
16	1	0	0.365651	-1.587912	0.695300
17	1	0	-0.565431	-0.987631	-0.711807
18	6	0	3.781138	-1.408988	-1.263694
19	1	0	2.926750	-1.699415	-1.875450
20	1	0	4.343073	-0.633033	-1.785730
21	1	0	4.420710	-2.277226	-1.094746
22	15	0	-3.636214	-0.189326	-0.032162
23	8	0	-2.496155	0.092694	-0.997808
24	6	0	-4.462299	1.319850	0.532691
25	1	0	-4.863810	1.852361	-0.332766
26	1	0	-5.276181	1.081006	1.221620
27	1	0	-3.733884	1.958025	1.038738
28	6	0	-3.071270	-1.044428	1.462088
29	1	0	-3.899897	-1.210069	2.154900
30	1	0	-2.636189	-2.007247	1.182321
31	1	0	-2.306512	-0.434618	1.950910
32	6	0	-4.928596	-1.229401	-0.756184
33	1	0	-5.726885	-1.410597	-0.032273
34	1	0	-5.341977	-0.726235	-1.633454
35	1	0	-4.492662	-2.183072	-1.062698

2-(1,1-Difluoroethyl)-*N*-methylpyridinium Conformer B – Me_3PO Complex

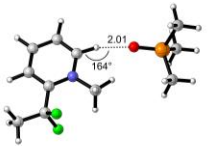

Standard orientation:

Center Number	Atomic Number	Atomic Type	Coordinates (Angstroms) X	Y	Z
1	6	0	-1.139551	2.691737	0.027569
2	6	0	-2.522463	2.729742	-0.028598
3	6	0	-3.233508	1.528523	-0.039755
4	6	0	-2.561796	0.322758	0.008633
5	6	0	-0.501335	1.461388	0.075140
6	1	0	-3.052932	3.674178	-0.066341
7	1	0	-4.313963	1.537480	-0.086242
8	1	0	0.580325	1.352660	0.115445
9	6	0	-3.318289	-1.003242	-0.023798
10	9	0	-2.922271	-1.683179	-1.149301
11	9	0	-2.896768	-1.761415	1.037398
12	1	0	-0.540009	3.592946	0.034657
13	7	0	-1.202124	0.311183	0.067755
14	6	0	-0.412987	-0.947613	0.158472
15	1	0	-0.549156	-1.371314	1.151756
16	1	0	-0.755683	-1.644579	-0.600978
17	1	0	0.630298	-0.679657	-0.001688
18	6	0	-4.819718	-0.924875	-0.003181
19	1	0	-5.165233	-0.423825	0.901820
20	1	0	-5.190575	-0.406689	-0.888136
21	1	0	-5.191940	-1.950357	-0.009479
22	15	0	3.770838	-0.121283	-0.016338
23	8	0	2.446666	0.622125	0.031525
24	6	0	4.751692	0.288544	-1.482029
25	1	0	4.953557	1.362289	-1.488342
26	1	0	5.697789	-0.258322	-1.475875
27	1	0	4.185451	0.025279	-2.378496
28	6	0	3.553569	-1.919681	-0.042579
29	1	0	4.521689	-2.425335	-0.078574
30	1	0	3.017293	-2.229273	0.858023
31	1	0	2.967814	-2.197839	-0.922272
32	6	0	4.828173	0.234910	1.409629
33	1	0	5.766656	-0.320803	1.339901
34	1	0	5.042280	1.305841	1.438841
35	1	0	4.303934	-0.050524	2.324677

2-(Difluoromethyl)-1,6-dimethylpyridinium Conformer A – Me_3PO Complex

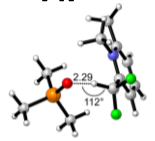

Standard orientation:

Center Number	Atomic Number	Atomic Type	Coordinates (Angstroms) X	Y	Z
1	6	0	-1.473603	1.962427	1.368312
2	6	0	-1.434967	0.859070	2.202883
3	6	0	-1.394092	-0.416102	1.641374
4	6	0	-1.419267	-0.541620	0.269943
5	6	0	-1.510429	1.803038	-0.016435
6	1	0	-1.416820	0.984279	3.279690
7	1	0	-1.322335	-1.306345	2.252175
8	6	0	-1.325830	-1.906985	-0.384670
9	1	0	-0.615847	-1.939083	-1.209400
10	9	0	-0.949949	-2.812603	0.551221
11	9	0	-2.566886	-2.282989	-0.821349
12	1	0	-1.477157	2.967767	1.770589
13	7	0	-1.516788	0.548853	-0.532307
14	6	0	-1.529602	0.358940	-2.000662
15	1	0	-0.492473	0.262142	-2.328318
16	1	0	-2.102410	-0.532178	-2.241608
17	1	0	-2.013012	1.208753	-2.469223
18	6	0	-1.530094	2.987177	-0.932126
19	1	0	-0.718067	2.934732	-1.662073
20	1	0	-2.478840	3.055366	-1.472347
21	1	0	-1.409147	3.893610	-0.341057
22	15	0	2.391566	-0.054334	-0.124282
23	8	0	1.150189	-0.531262	-0.857507
24	6	0	2.321225	-0.375778	1.657404
25	1	0	2.141845	-1.441291	1.822279
26	1	0	3.257652	-0.084726	2.139948
27	1	0	1.499080	0.197018	2.093846
28	6	0	2.658234	1.729534	-0.293561
29	1	0	3.562973	2.038674	0.235837
30	1	0	2.753387	1.980059	-1.352825
31	1	0	1.797961	2.259609	0.124038
32	6	0	3.905608	-0.850636	-0.717645
33	1	0	4.776625	-0.477988	-0.173079
34	1	0	3.821419	-1.930298	-0.572710
35	1	0	4.026101	-0.641019	-1.783076

2-(Difluoromethyl)-1,6-dimethylpyridinium Conformer B – Me_3PO Complex

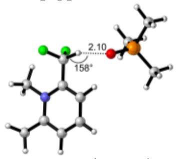

Standard orientation:

Center Number	Atomic Number	Atomic Type	Coordinates (Angstroms) X	Y	Z
1	6	0	-2.812247	2.149362	-0.110570
2	6	0	-1.464893	2.237016	-0.405279
3	6	0	-0.709038	1.065183	-0.475918
4	6	0	-1.323349	-0.144425	-0.234500
5	6	0	-3.409578	0.910172	0.126539
6	1	0	-1.002817	3.200384	-0.589082
7	1	0	0.349938	1.062322	-0.723118
8	6	0	-0.478322	-1.395022	-0.346767
9	1	0	0.530581	-1.142956	-0.683884
10	9	0	-0.398523	-2.030925	0.861553
11	9	0	-1.048657	-2.277448	-1.217935
12	1	0	-3.434502	3.034215	-0.061161
13	7	0	-2.648309	-0.212013	0.075344
14	6	0	-3.302348	-1.518865	0.329973
15	1	0	-3.857103	-1.814274	-0.561391
16	1	0	-2.554106	-2.264202	0.567239
17	1	0	-3.974998	-1.411608	1.176654
18	6	0	-4.869619	0.801143	0.433896
19	1	0	-5.363931	0.077961	-0.218881
20	1	0	-5.029286	0.492945	1.471577
21	1	0	-5.335832	1.774874	0.292973
22	15	0	3.320080	0.232027	0.013093
23	8	0	2.227386	0.038923	-1.026401
24	6	0	2.933992	-0.599474	1.575508
25	1	0	2.814069	-1.670047	1.391006
26	1	0	3.730288	-0.444564	2.307719
27	1	0	1.996243	-0.197812	1.968890
28	6	0	3.584741	1.976087	0.423584
29	1	0	4.369444	2.084250	1.176359
30	1	0	3.872472	2.516605	-0.481274
31	1	0	2.652267	2.395896	0.809370
32	6	0	4.924172	-0.412683	-0.522437
33	1	0	5.679368	-0.258372	0.252222
34	1	0	4.828982	-1.481288	-0.728542
35	1	0	5.230335	0.102364	-1.436008

2-(Difluoromethyl)-6-methoxy-*N*-methylpyridinium Conformer A – Me_3PO Complex

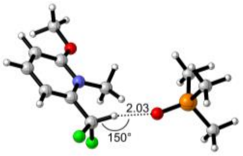

Standard orientation:

Center Number	Atomic Number	Atomic Type	Coordinates (Angstroms) X	Y	Z
1	6	0	3.858032	-0.299542	0.678971
2	6	0	3.786495	1.031741	1.026397
3	6	0	2.641736	1.779607	0.721255
4	6	0	1.594643	1.161709	0.085009
5	6	0	2.765585	-0.886823	0.028684
6	1	0	4.623751	1.502337	1.529120
7	1	0	2.567891	2.829194	0.969140
8	6	0	0.346478	1.929875	-0.305954
9	1	0	-0.583645	1.551330	0.125519
10	9	0	0.510985	3.229271	0.050596
11	9	0	0.234366	1.901934	-1.668862
12	1	0	4.737562	-0.890183	0.893741
13	7	0	1.648716	-0.162474	-0.236560
14	6	0	0.517977	-0.854713	-0.904099
15	1	0	-0.405146	-0.302617	-0.736557
16	1	0	0.736719	-0.934339	-1.969512
17	1	0	0.430807	-1.847356	-0.469723
18	8	0	2.699791	-2.136270	-0.378766
19	6	0	3.820065	-3.006410	-0.138884
20	1	0	3.523431	-3.964745	-0.555595
21	1	0	4.703757	-2.626609	-0.654523
22	1	0	3.998229	-3.098559	0.933812
23	15	0	-3.483074	-0.408108	0.211006
24	8	0	-2.317926	0.521369	-0.083889
25	6	0	-4.201036	-0.136409	1.850957
26	1	0	-4.557973	0.894035	1.918010
27	1	0	-5.034970	-0.821061	2.024779
28	1	0	-3.432841	-0.299191	2.610565
29	6	0	-3.005739	-2.154923	0.152924
30	1	0	-3.864459	-2.797527	0.361931
31	1	0	-2.615448	-2.386421	-0.841464
32	1	0	-2.226463	-2.338955	0.896686
33	6	0	-4.843200	-0.220726	-0.969247
34	1	0	-5.660026	-0.904712	-0.726051
35	1	0	-5.208982	0.808112	-0.933841
36	1	0	-4.476397	-0.435748	-1.975654

2-(Difluoromethyl)-1,3-dimethyl-benzoimidazolium Conformer A – Me_3PO Complex

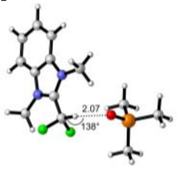

Standard orientation:

Center Number	Atomic Number	Atomic Type	Coordinates (Angstroms) X	Y	Z
1	6	0	4.092445	0.220529	-0.930673
2	6	0	2.832681	0.223135	-0.325546
3	6	0	4.806792	-0.963117	-0.846293
4	6	0	2.321449	-0.903031	0.325901
5	6	0	3.042958	-2.096857	0.413301
6	1	0	2.651270	-2.967915	0.925442
7	6	0	4.291738	-2.100901	-0.185739
8	1	0	4.893816	-3.001673	-0.145569
9	1	0	5.790959	-1.018939	-1.297829
10	1	0	4.489298	1.094637	-1.433849
11	7	0	1.065025	-0.554982	0.801014
12	7	0	1.869869	1.214502	-0.218762
13	6	0	0.835164	0.716571	0.464670
14	6	0	0.198697	-1.459925	1.564529
15	1	0	0.264405	-2.445215	1.103311
16	1	0	0.550243	-1.505187	2.595878
17	1	0	-0.835107	-1.115533	1.512693
18	6	0	-0.401024	1.504869	0.814831
19	1	0	-1.131600	0.944036	1.396894
20	9	0	-0.017696	2.630860	1.475044
21	9	0	-0.996164	1.906349	-0.344841
22	6	0	2.016549	2.567514	-0.761229
23	1	0	2.298918	2.487114	-1.810888
24	1	0	1.071435	3.096581	-0.679899
25	1	0	2.788962	3.093275	-0.199067
26	15	0	-3.494196	-0.576648	-0.253672
27	8	0	-2.749648	-0.293966	1.041670
28	6	0	-4.320022	0.888847	-0.922990
29	1	0	-3.573382	1.664306	-1.109145
30	1	0	-4.836015	0.647267	-1.855526
31	1	0	-5.043958	1.256633	-0.192015
32	6	0	-4.779762	-1.836018	-0.057639
33	1	0	-5.294035	-2.011174	-1.005769
34	1	0	-4.320442	-2.766666	0.283793
35	1	0	-5.501257	-1.497874	0.689862
36	6	0	-2.398133	-1.178747	-1.565163
37	1	0	-2.966192	-1.402378	-2.471596
38	1	0	-1.650635	-0.413336	-1.788750
39	1	0	-1.893257	-2.085261	-1.221603

2-(Difluoromethyl)-6-methoxy-*N*-methylpyridinium Conformer B – Me_3PO Complex

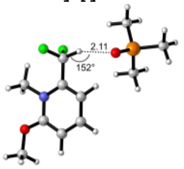

Standard orientation:

Center Number	Atomic Number	Atomic Type	Coordinates (Angstroms) X	Y	Z
1	6	0	2.622260	-1.821861	-0.370525
2	6	0	1.327735	-1.970086	-0.815269
3	6	0	0.464406	-0.864606	-0.847726
4	6	0	0.930046	0.356178	-0.427271
5	6	0	3.051816	-0.555039	0.050683
6	1	0	0.978808	-2.943004	-1.142627
7	1	0	-0.566956	-0.937448	-1.181652
8	6	0	-0.010119	1.538223	-0.485511
9	1	0	-0.974855	1.236914	-0.899440
10	9	0	-0.206912	2.051576	0.766784
11	9	0	0.529385	2.539830	-1.240427
12	1	0	3.306836	-2.658219	-0.340462
13	7	0	2.209551	0.507224	0.025359
14	6	0	2.753762	1.802760	0.499479
15	1	0	3.556845	2.105229	-0.171753
16	1	0	1.967632	2.545574	0.509247
17	1	0	3.148259	1.658458	1.503661
18	8	0	4.254673	-0.262928	0.495867
19	6	0	5.242599	-1.306014	0.577272
20	1	0	6.129701	-0.819670	0.972845
21	1	0	4.903851	-2.087786	1.259094
22	1	0	5.443109	-1.708055	-0.417283
23	15	0	-3.482497	-0.413808	0.093890
24	8	0	-2.585713	-0.106280	-1.094385
25	6	0	-2.633719	-1.390083	1.363067
26	1	0	-1.770657	-0.826210	1.727337
27	1	0	-3.302083	-1.606390	2.200303
28	1	0	-2.287262	-2.328557	0.922691
29	6	0	-4.960609	-1.352396	-0.366015
30	1	0	-5.583382	-1.547882	0.510496
31	1	0	-5.534138	-0.777948	-1.097292
32	1	0	-4.657237	-2.301061	-0.814959
33	6	0	-4.069896	1.081956	0.929098
34	1	0	-4.700875	0.825651	1.783681
35	1	0	-3.207584	1.657347	1.275352
36	1	0	-4.643041	1.686425	0.222148

2-(Difluoromethyl)-1,3-dimethyl-benzoimidazolium Conformer B – Me_3PO Complex

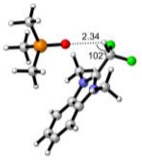

Standard orientation:

Center Number	Atomic Number	Atomic Type	Coordinates (Angstroms) X	Y	Z
1	6	0	-2.490619	-1.459788	-1.229102
2	6	0	-1.809854	-0.456773	-0.536386
3	6	0	-2.816731	-2.593530	-0.501541
4	6	0	-1.485702	-0.572482	0.819000
5	6	0	-1.819020	-1.715131	1.552096
6	1	0	-1.572965	-1.822406	2.601666
7	6	0	-2.484091	-2.718455	0.863916
8	1	0	-2.758191	-3.625165	1.391492
9	1	0	-3.340011	-3.405893	-0.993214
10	1	0	-2.744517	-1.360777	-2.278181
11	7	0	-0.807514	0.590226	1.171510
12	7	0	-1.301618	0.761705	-0.960425
13	6	0	-0.703037	1.346199	0.077894
14	6	0	-0.152975	0.808883	2.466611
15	1	0	-0.562056	1.693148	2.950373
16	1	0	-0.347108	-0.065403	3.083043
17	1	0	0.918930	0.915721	2.298305
18	6	0	0.005903	2.672506	-0.059993
19	1	0	0.847964	2.606524	-0.749514
20	9	0	-0.896498	3.588215	-0.512030
21	9	0	0.442541	3.093246	1.145733
22	6	0	-1.399599	1.267429	-2.329658
23	1	0	-2.399881	1.665666	-2.502302
24	1	0	-0.659149	2.047919	-2.488317
25	1	0	-1.197275	0.441251	-3.010810
26	15	0	2.409745	-0.816725	-0.270880
27	8	0	1.804783	0.569484	-0.122669
28	6	0	1.330987	-1.971125	-1.160394
29	1	0	1.040405	-1.529739	-2.117564
30	1	0	1.848796	-2.917153	-1.338853
31	1	0	0.433201	-2.164898	-0.565786
32	6	0	2.748835	-1.603793	1.324725
33	1	0	3.208878	-2.584464	1.179741
34	1	0	3.421466	-0.967917	1.905264
35	1	0	1.809779	-1.726832	1.871391
36	6	0	3.979884	-0.795972	-1.171891
37	1	0	4.398279	-1.802698	-1.245085
38	1	0	3.811282	-0.398622	-2.175550
39	1	0	4.683376	-0.147726	-0.644140

2-(Difluoromethyl)-*N*-methylquinolinium Conformer A – Me_3PO Complex

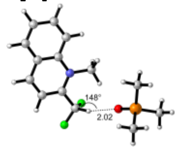

Standard orientation:

Center Number	Atomic Number	Atomic Type	Coordinates (Angstroms)		
			X	Y	Z
1	6	0	-3.459232	0.122691	-0.531070
2	6	0	-3.202259	1.437398	-0.968214
3	6	0	-1.998619	2.035706	-0.682936
4	6	0	-1.027736	1.317731	0.022404
5	6	0	-2.450282	-0.571853	0.188526
6	1	0	-1.778179	3.048616	-0.989257
7	7	0	-1.235715	0.056518	0.416916
8	6	0	-0.179098	-0.711281	1.118105
9	1	0	-0.105069	-1.689078	0.645963
10	1	0	0.785438	-0.217909	1.024949
11	1	0	-0.454802	-0.812908	2.168582
12	6	0	0.296719	1.980144	0.373335
13	9	0	0.277986	3.256440	-0.086835
14	9	0	0.389899	2.049737	1.734736
15	1	0	1.183849	1.469646	-0.011885
16	6	0	-4.701649	-0.516698	-0.782367
17	6	0	-2.704988	-1.878419	0.665136
18	6	0	-3.924040	-2.461896	0.407408
19	1	0	-4.115538	-3.462072	0.780448
20	6	0	-4.929955	-1.787769	-0.324420
21	1	0	-5.878311	-2.277880	-0.512415
22	1	0	-1.969948	-2.422716	1.240921
23	1	0	-5.459557	0.026493	-1.337171
24	1	0	-3.966420	1.974749	-1.520993
25	15	0	3.779861	-0.650286	-0.256972
26	8	0	2.793441	0.312569	0.382672
27	6	0	4.385979	-0.071569	-1.862112
28	1	0	4.887646	0.889933	-1.730005
29	1	0	5.087706	-0.791713	-2.290141
30	1	0	3.538931	0.056585	-2.540195
31	6	0	3.060622	-2.286243	-0.553279
32	1	0	3.793840	-2.951773	-1.015445
33	1	0	2.735025	-2.712385	0.398953
34	1	0	2.197026	-2.183915	-1.215260
35	6	0	5.248307	-0.926132	0.765170
36	1	0	5.932634	-1.623313	0.275489
37	1	0	5.755954	0.027619	0.927160
38	1	0	4.940749	-1.336316	1.729878

4-(Difluoromethyl)-*N*-methylquinolinium Conformer A – Me_3PO Complex

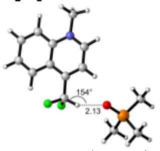

Standard orientation:

Center Number	Atomic Number	Atomic Type	Coordinates (Angstroms)		
			X	Y	Z
1	6	0	2.163032	-0.470937	-0.038133
2	6	0	0.773120	-0.313731	-0.296300
3	6	0	0.233802	0.937812	-0.459768
4	6	0	1.074606	2.053945	-0.372838
5	6	0	2.970964	0.696537	0.036908
6	1	0	-0.828746	1.066129	-0.652657
7	7	0	2.374903	1.935789	-0.137720
8	6	0	3.201271	3.156457	-0.058218
9	1	0	3.968211	3.121095	-0.831412
10	1	0	2.557036	4.016020	-0.219617
11	1	0	3.655428	3.216798	0.930451
12	1	0	0.693860	3.060222	-0.496129
13	6	0	-0.156414	-1.501097	-0.402941
14	9	0	0.290122	-2.347746	-1.379934
15	9	0	-0.127728	-2.211485	0.766306
16	1	0	-1.185152	-1.204724	-0.621108
17	6	0	2.780189	-1.736538	0.147915
18	6	0	4.356797	0.592166	0.286024
19	6	0	4.916182	-0.652224	0.458904
20	1	0	5.980494	-0.732015	0.650460
21	6	0	4.127810	-1.822541	0.391637
22	1	0	4.592355	-2.791739	0.533144
23	1	0	4.982025	1.472792	0.342939
24	1	0	2.174893	-2.632734	0.095578
25	15	0	-3.945964	0.259503	0.071388
26	8	0	-2.767408	0.203094	-0.886572
27	6	0	-3.947671	-1.117844	1.248379
28	1	0	-3.027493	-1.085842	1.837451
29	1	0	-4.808413	-1.055025	1.918774
30	1	0	-3.985321	-2.060010	0.695966
31	6	0	-5.543761	0.201210	-0.778177
32	1	0	-6.365668	0.253844	-0.060020
33	1	0	-5.611139	1.044858	-1.469151
34	1	0	-5.614983	-0.730891	-1.343601
35	6	0	-3.960733	1.771226	1.069008
36	1	0	-4.812368	1.774889	1.753732
37	1	0	-3.032790	1.827817	1.643492
38	1	0	-4.024539	2.637135	0.405746

2-(Difluoromethyl)-*N*-methylquinolinium Conformer B – Me_3PO Complex

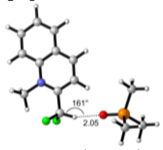

Standard orientation:

Center Number	Atomic Number	Atomic Type	Coordinates (Angstroms)		
			X	Y	Z
1	6	0	2.450927	-1.400889	-0.034561
2	6	0	1.053893	-1.570477	-0.079973
3	6	0	0.228045	-0.470267	-0.083138
4	6	0	0.791821	0.807855	-0.041491
5	6	0	2.985609	-0.083782	0.007295
6	1	0	-0.855649	-0.543975	-0.118153
7	7	0	2.117184	0.995420	0.000435
8	6	0	2.711784	2.351285	0.042451
9	1	0	3.361820	2.470191	-0.823631
10	1	0	1.934588	3.102109	0.012945
11	1	0	3.279831	2.452264	0.966854
12	6	0	-0.177249	1.977977	-0.043158
13	9	0	0.007313	2.743679	1.071194
14	9	0	0.054295	2.780905	-1.122011
15	1	0	-1.208653	1.620941	-0.070841
16	6	0	3.335667	-2.512136	-0.028458
17	6	0	4.387461	0.100291	0.056823
18	6	0	5.210375	-1.001667	0.062032
19	1	0	6.284025	-0.853750	0.100769
20	6	0	4.690519	-2.317666	0.018781
21	1	0	5.367700	-3.164036	0.023933
22	1	0	4.826858	1.087195	0.092373
23	1	0	2.910103	-3.509632	-0.061488
24	1	0	0.639215	-2.573467	-0.111070
25	15	0	-4.034027	-0.547111	0.005410
26	8	0	-2.814830	0.343247	-0.168892
27	6	0	-5.191468	-0.428620	-1.381528
28	1	0	-4.678972	-0.721027	-2.300945
29	1	0	-6.052099	-1.082791	-1.221674
30	1	0	-5.533371	0.604872	-1.474997
31	6	0	-4.987241	-0.149065	1.492383
32	1	0	-5.848458	-0.815175	1.586469
33	1	0	-4.345072	-0.259895	2.369255
34	1	0	-5.333399	0.885286	1.430425
35	6	0	-3.591985	-2.298292	0.146150
36	1	0	-4.485853	-2.915082	0.267518
37	1	0	-3.058728	-2.605968	-0.756934
38	1	0	-2.938552	-2.433847	1.011831

4-(Difluoromethyl)-*N*-methylquinolinium Conformer B – Me_3PO Complex

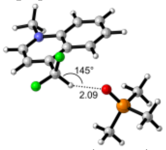

Standard orientation:

Center Number	Atomic Number	Atomic Type	Coordinates (Angstroms)		
			X	Y	Z
1	6	0	1.379346	0.153421	-0.331099
2	6	0	1.284547	-1.246916	-0.106636
3	6	0	2.321570	-1.920451	0.488633
4	6	0	3.475011	-1.214189	0.844296
5	6	0	2.576199	0.822698	0.043756
6	1	0	2.276355	-2.983656	0.682930
7	7	0	3.596901	0.088995	0.625575
8	6	0	4.849947	0.766241	1.011818
9	1	0	4.628694	1.536034	1.750601
10	1	0	5.520105	0.026721	1.440720
11	1	0	5.305846	1.205796	0.125110
12	1	0	4.319645	-1.709095	1.307211
13	6	0	0.041101	-1.988908	-0.544820
14	9	0	-0.013894	-1.960947	-1.912906
15	9	0	0.135139	-3.298612	-0.183692
16	1	0	-0.895720	-1.572199	-0.164229
17	6	0	0.324947	0.908264	-0.911707
18	6	0	2.716129	2.209977	-0.174452
19	6	0	1.677228	2.906276	-0.747845
20	1	0	1.784317	3.972332	-0.915366
21	6	0	0.476483	2.258014	-1.113053
22	1	0	-0.330469	2.833977	-1.552522
23	1	0	3.622900	2.730487	0.102639
24	1	0	-0.610058	0.416664	-1.169231
25	15	0	-3.480580	0.289805	0.338685
26	8	0	-2.585848	-0.442213	-0.646561
27	6	0	-3.537151	-0.516440	1.960169
28	1	0	-2.524794	-0.564429	2.369339
29	1	0	-4.179825	0.039380	2.647243
30	1	0	-3.923330	-1.531615	1.842084
31	6	0	-5.197055	0.407705	-0.225656
32	1	0	-5.810665	0.934748	0.509085
33	1	0	-5.225240	0.946589	-1.175681
34	1	0	-5.593502	-0.599535	-0.373987
35	6	0	-2.935364	1.990535	0.647135
36	1	0	-3.597546	2.485453	1.362035
37	1	0	-1.917822	1.974418	1.046547
38	1	0	-2.940537	2.544259	-0.295186

N-Methyl-9-(difluoromethyl)acridinium – Me_3PO Complex

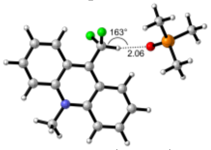

Standard orientation:

Center Number	Atomic Number	Atomic Type	Coordinates (Angstroms)		
			X	Y	Z
1	6	0	-0.686112	0.956179	0.300386
2	6	0	-0.870732	-0.432769	0.347441
3	6	0	-2.125376	-0.998122	0.062428
4	6	0	-3.233483	-0.126617	-0.182710
5	6	0	-1.824956	1.791273	0.044694
6	7	0	-3.060686	1.227414	-0.095757
7	6	0	-4.246795	2.105426	-0.141846
8	1	0	-4.465497	2.395806	-1.171139
9	1	0	-5.090250	1.579647	0.293225
10	1	0	-4.059412	2.981182	0.470861
11	6	0	0.295736	-1.345875	0.689389
12	9	0	-0.063335	-2.151439	1.734569
13	9	0	0.544201	-2.168233	-0.376945
14	1	0	1.230852	-0.852139	0.946446
15	6	0	0.590866	1.581623	0.490485
16	6	0	-1.649900	3.196481	-0.066077
17	6	0	-0.405681	3.744386	0.102275
18	1	0	-0.284190	4.816981	-0.004155
19	6	0	0.722447	2.936873	0.397909
20	1	0	1.693958	3.398438	0.534553
21	1	0	-2.477211	3.840980	-0.325163
22	1	0	1.472730	0.981168	0.691048
23	6	0	-4.496579	-0.680637	-0.522718
24	6	0	-2.343196	-2.412600	0.002966
25	6	0	-3.571784	-2.919035	-0.307392
26	1	0	-3.722717	-3.991023	-0.357805
27	6	0	-4.649971	-2.040606	-0.585362
28	1	0	-5.615075	-2.449115	-0.864880
29	1	0	-5.332761	-0.045690	-0.776898
30	1	0	-1.516875	-3.083029	0.193423
31	15	0	4.220140	-0.232038	-0.181875
32	8	0	3.196752	-0.249384	0.941748
33	6	0	5.923959	-0.345999	0.419340
34	1	0	6.046377	-1.278291	0.975650
35	1	0	6.628801	-0.326115	-0.415734
36	1	0	6.125772	0.496953	1.084408
37	6	0	4.136808	1.283932	-1.170797
38	1	0	4.891601	1.272014	-1.960905
39	1	0	3.143756	1.365847	-1.620385
40	1	0	4.306414	2.145042	-0.519495
41	6	0	4.000910	-1.601220	-1.346252
42	1	0	4.756234	-1.563089	-2.134963
43	1	0	4.086069	-2.547069	-0.805781
44	1	0	3.005342	-1.533874	-1.791443

Hydrogen bonding complexes with neutral donors:

Phenol – Me_3PO Complex

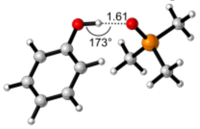

Standard orientation:

Center Number	Atomic Number	Atomic Type	Coordinates (Angstroms)		
			X	Y	Z
1	6	0	-2.856834	-1.184807	0.264893
2	6	0	-1.674409	-0.769171	-0.360052
3	6	0	-1.495421	0.587066	-0.667584
4	6	0	-2.487278	1.510429	-0.337680
5	6	0	-3.841462	-0.253171	0.583064
6	1	0	-0.584994	0.900877	-1.169943
7	1	0	-4.753603	-0.588314	1.068005
8	1	0	-2.984232	-2.237976	0.495644
9	1	0	-2.336212	2.558513	-0.579479
10	6	0	-3.664617	1.100898	0.288435
11	1	0	-4.433440	1.823479	0.540825
12	8	0	-0.742584	-1.705178	-0.652681
13	1	0	0.119161	-1.273029	-0.916404
14	15	0	2.271596	0.070171	0.028420
15	8	0	1.534746	-0.539262	-1.162877
16	6	0	3.971101	-0.529679	0.158479
17	1	0	4.512512	-0.271384	-0.754598
18	1	0	4.471461	-0.078004	1.018274
19	1	0	3.958429	-1.615841	0.273545
20	6	0	1.456352	-0.311254	1.598253
21	1	0	1.997551	0.142286	2.432207
22	1	0	0.434556	0.078632	1.572732
23	1	0	1.421604	-1.395139	1.731963
24	6	0	2.364037	1.873280	-0.077735
25	1	0	2.922724	2.279400	0.768884
26	1	0	2.862357	2.151728	-1.009148
27	1	0	1.352086	2.286191	-0.074782

2-(Difluoromethyl)pyridine Conformer A – Me_3PO Complex

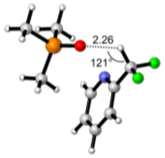

Standard orientation:

Center Number	Atomic Number	Atomic Type	Coordinates (Angstroms)		
			X	Y	Z
1	6	0	-1.906595	-2.535783	0.043377
2	6	0	-2.206262	-1.823405	-1.113613
3	6	0	-2.043295	-0.439013	-1.114029
4	6	0	-1.588094	0.163903	0.054859
5	6	0	-1.446144	-1.832516	1.157450
6	1	0	-2.559311	-2.334654	-2.003029
7	1	0	-2.261014	0.158743	-1.991061
8	1	0	-1.189977	-2.356947	2.074247
9	6	0	-1.422553	1.658711	0.174098
10	1	0	-0.448030	1.947292	0.565949
11	9	0	-1.607451	2.258852	-1.038383
12	9	0	-2.406816	2.154578	0.996662
13	1	0	-2.017125	-3.613253	0.087782
14	7	0	-1.284749	-0.506800	1.170071
15	15	0	2.258913	0.109849	-0.076019
16	8	0	1.463167	1.337594	-0.474251
17	6	0	1.685505	-1.401078	-0.897467
18	1	0	1.762722	-1.269827	-1.979685
19	1	0	2.286692	-2.261145	-0.591569
20	1	0	0.639299	-1.579896	-0.635474
21	6	0	2.188277	-0.230305	1.702340
22	1	0	2.748460	-1.136002	1.948109
23	1	0	2.613567	0.617837	2.244725
24	1	0	1.140834	-0.353313	1.991090
25	6	0	4.020295	0.243772	-0.486083
26	1	0	4.557445	-0.658611	-0.182953
27	1	0	4.127541	0.383995	-1.564429
28	1	0	4.444710	1.108313	0.029967

2-(Difluoromethyl)pyridine Conformer B – Me_3PO Complex

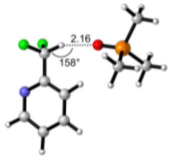

Standard orientation:

Center Number	Atomic Number	Atomic Type	Coordinates (Angstroms)		
			X	Y	Z
1	6	0	-3.276775	-2.224972	0.009085
2	6	0	-1.888802	-2.166081	-0.021684
3	6	0	-1.272193	-0.914480	-0.031633
4	6	0	-2.086773	0.212715	-0.009653
5	6	0	-3.997965	-1.027842	0.028728
6	1	0	-1.294721	-3.074036	-0.037929
7	1	0	-0.192822	-0.791633	-0.056566
8	1	0	-5.084336	-1.042527	0.052743
9	6	0	-1.457302	1.581456	-0.017562
10	1	0	-0.366129	1.552881	-0.042232
11	9	0	-1.908660	2.298625	-1.097109
12	9	0	-1.859599	2.287855	1.088318
13	1	0	-3.801725	-3.173594	0.017888
14	7	0	-3.423035	0.175102	0.019857
15	15	0	2.913294	-0.090046	-0.006401
16	8	0	1.611746	0.682689	-0.114532
17	6	0	3.163505	-1.235399	-1.388291
18	1	0	3.181013	-0.670736	-2.323501
19	1	0	4.104244	-1.779782	-1.274937
20	1	0	2.334238	-1.946854	-1.416727
21	6	0	3.002266	-1.101973	1.494678
22	1	0	3.945571	-1.652229	1.536822
23	1	0	2.920877	-0.453357	2.370170
24	1	0	2.169567	-1.810020	1.497069
25	6	0	4.374973	0.979696	0.025548
26	1	0	5.287532	0.383390	0.103182
27	1	0	4.405747	1.571788	-0.892127
28	1	0	4.307548	1.653129	0.883233

4-(Difluoromethyl)pyridine Conformer A – Me_3PO Complex

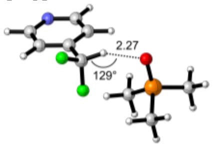

Standard orientation:

Center Number	Atomic Number	Atomic Type	Coordinates (Angstroms) X	Y	Z
1	6	0	3.000894	0.448325	-0.431303
2	6	0	1.783420	0.318540	0.233734
3	6	0	1.392287	-0.933942	0.696324
4	6	0	2.245238	-2.013297	0.465336
5	6	0	3.775959	-0.694698	-0.607598
6	1	0	0.441409	-1.057241	1.207745
7	1	0	4.731708	-0.632090	-1.120610
8	1	0	3.343841	1.409540	-0.798784
9	7	0	3.415168	-1.908197	-0.172325
10	1	0	1.973485	-3.006886	0.811675
11	6	0	0.877379	1.502697	0.427219
12	9	0	1.613949	2.632460	0.659555
13	9	0	0.192065	1.743311	-0.743760
14	1	0	0.139899	1.371464	1.218800
15	8	0	-1.634483	-0.042091	1.277335
16	15	0	-2.492419	-0.289566	0.050070
17	6	0	-3.932332	-1.334434	0.397291
18	1	0	-4.527837	-1.485797	-0.506587
19	1	0	-3.591479	-2.302180	0.772956
20	1	0	-4.548345	-0.852325	1.160138
21	6	0	-3.152466	1.235472	-0.672216
22	1	0	-2.318931	1.883736	-0.951702
23	1	0	-3.755901	1.014272	-1.556117
24	1	0	-3.771033	1.744508	0.070919
25	6	0	-1.591441	-1.121942	-1.285406
26	1	0	-2.248131	-1.295075	-2.141891
27	1	0	-0.749048	-0.496977	-1.592366
28	1	0	-1.211404	-2.079623	-0.920062

4-(Difluoromethyl)pyridine Conformer B – Me_3PO Complex

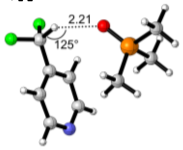

Standard orientation:

Center Number	Atomic Number	Atomic Type	Coordinates (Angstroms) X	Y	Z
1	6	0	1.195597	-0.530290	1.162092
2	6	0	1.659984	0.133655	0.029209
3	6	0	2.125074	-0.617478	-1.045946
4	6	0	2.105277	-2.007622	-0.938637
5	6	0	1.223321	-1.922907	1.168305
6	1	0	2.495182	-0.141743	-1.947295
7	1	0	0.871453	-2.471075	2.038340
8	1	0	0.819231	0.019570	2.017451
9	7	0	1.663697	-2.660273	0.141386
10	1	0	2.461360	-2.621671	-1.761275
11	6	0	1.604645	1.634740	-0.054034
12	9	0	1.765411	2.179485	1.191216
13	9	0	2.642367	2.099629	-0.819590
14	1	0	0.662860	2.001067	-0.463940
15	15	0	-2.260307	0.191447	-0.090886
16	8	0	-1.345177	1.364241	0.200914
17	6	0	-3.849685	0.698782	-0.800712
18	1	0	-3.669511	1.222675	-1.742603
19	1	0	-4.486904	-0.169723	-0.985226
20	1	0	-4.351947	1.376448	-0.106212
21	6	0	-2.668399	-0.775357	1.387636
22	1	0	-3.330223	-1.607465	1.134718
23	1	0	-1.747008	-1.167164	1.825874
24	1	0	-3.161509	-0.126998	2.115788
25	6	0	-1.558887	-0.995176	-1.270010
26	1	0	-2.288840	-1.772782	-1.509375
27	1	0	-1.276959	-0.467479	-2.184712
28	1	0	-0.668611	-1.459492	-0.837313

2-(Difluoromethyl)nitrobenzene Conformer A – Me_3PO Complex

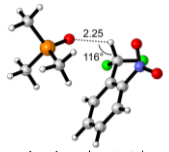

Standard orientation:

Center Number	Atomic Number	Atomic Type	Coordinates (Angstroms) X	Y	Z
1	6	0	1.307575	-2.656379	-0.897791
2	6	0	0.673745	-1.934523	-1.906483
3	6	0	0.578683	-0.544434	-1.829048
4	6	0	1.094489	0.150964	-0.737228
5	6	0	1.836803	-1.985040	0.200425
6	1	0	0.255536	-2.450667	-2.764007
7	1	0	0.104314	0.013813	-2.627416
8	1	0	2.326812	-2.515768	1.007762
9	6	0	1.042151	1.663665	-0.717906
10	1	0	0.404880	2.077765	0.056811
11	9	0	2.320373	2.149183	-0.578450
12	9	0	0.603684	2.118441	-1.928173
13	1	0	1.386541	-3.735813	-0.957731
14	8	0	-1.705478	1.314267	0.126716
15	15	0	-2.588653	0.106681	0.368566
16	6	0	-3.985546	0.462935	1.467578
17	1	0	-4.607173	-0.425298	1.605565
18	1	0	-3.603205	0.791986	2.436887
19	1	0	-4.588259	1.263293	1.031737
20	6	0	-3.314493	-0.555520	-1.155219
21	1	0	-2.509902	-0.845868	-1.835950
22	1	0	-3.938729	-1.426560	-0.940892
23	1	0	-3.921212	0.218858	-1.630624
24	6	0	-1.708553	-1.284588	1.128405
25	1	0	-2.402520	-2.094359	1.368531
26	1	0	-0.951761	-1.656607	0.432412
27	1	0	-1.217064	-0.942892	2.043222
28	7	0	2.217947	0.044200	1.486296
29	8	0	3.134463	-0.497839	2.079486
30	8	0	1.677399	1.074436	1.852724
31	6	0	1.708198	-0.603251	0.268823

2-(Difluoromethyl)nitrobenzene Conformer B – Me_3PO Complex

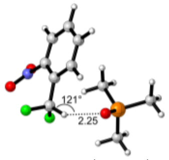

Standard orientation:

Center Number	Atomic Number	Atomic Type	Coordinates (Angstroms) X	Y	Z
1	6	0	1.553164	2.850580	0.017334
2	6	0	0.815915	2.596169	-1.136570
3	6	0	0.582027	1.282931	-1.543941
4	6	0	1.110801	0.205364	-0.836353
5	6	0	2.069552	1.790160	0.758692
6	1	0	0.400398	3.418039	-1.709226
7	1	0	-0.050443	1.081876	-2.402039
8	1	0	2.640895	1.956334	1.664702
9	6	0	0.735780	-1.180544	-1.298175
10	1	0	-0.137318	-1.152320	-1.947957
11	9	0	1.765875	-1.790583	-1.959805
12	9	0	0.432338	-1.976340	-0.223725
13	1	0	1.723900	3.868886	0.348069
14	7	0	2.461361	-0.581525	1.107516
15	8	0	2.392315	-0.484350	2.322233
16	8	0	3.015470	-1.488383	0.515370
17	6	0	1.857949	0.494639	0.308137
18	15	0	-2.679053	-0.095321	0.160618
19	8	0	-2.095175	-0.301951	-1.222745
20	6	0	-3.348760	-1.619436	0.877532
21	1	0	-2.549826	-2.363105	0.934029
22	1	0	-3.746199	-1.436254	1.878878
23	1	0	-4.145779	-1.999679	0.234170
24	6	0	-4.037889	1.104593	0.177319
25	1	0	-4.447788	1.212591	1.184494
26	1	0	-3.663950	2.071857	-0.167685
27	1	0	-4.824388	0.764067	-0.500357
28	6	0	-1.477895	0.517917	1.375304
29	1	0	-1.974543	0.729387	2.325898
30	1	0	-0.705200	-0.238665	1.535189
31	1	0	-1.012147	1.433036	0.997178

Difluoromethyl phenyl sulfone Conformer A – Me$_3$PO Complex

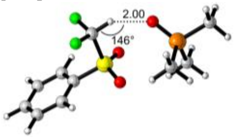

Standard orientation:

Center Number	Atomic Number	Atomic Type	X	Y	Z
1	6	0	-5.004691	0.731933	-0.049334
2	6	0	-4.527874	-0.071265	0.988007
3	6	0	-3.159457	-0.286703	1.129617
4	6	0	-2.296007	0.318496	0.217236
5	6	0	-4.123313	1.328059	-0.952912
6	1	0	-5.219510	-0.526693	1.688215
7	1	0	-2.764252	-0.900371	1.932378
8	1	0	-4.502198	1.954283	-1.753121
9	1	0	-6.072159	0.896918	-0.152848
10	6	0	-2.751853	1.123474	-0.826070
11	1	0	-2.046449	1.582158	-1.511068
12	16	0	-0.554939	0.029107	0.372253
13	6	0	-0.271124	-1.472997	-0.658867
14	1	0	0.795367	-1.713863	-0.624996
15	9	0	-1.047070	-2.459888	-0.165504
16	9	0	-0.691190	-1.193944	-1.910399
17	8	0	0.175570	1.116677	-0.283285
18	8	0	-0.247149	-0.355117	1.750230
19	15	0	3.459243	0.170377	0.034209
20	8	0	2.621793	-1.091703	-0.080068
21	6	0	3.046547	1.175103	1.483606
22	1	0	3.170474	0.568809	2.384316
23	1	0	3.701146	2.048598	1.540369
24	1	0	2.006035	1.496439	1.404188
25	6	0	3.295993	1.262369	-1.401383
26	1	0	3.922131	2.150348	-1.283035
27	1	0	3.603162	0.719621	-2.298792
28	1	0	2.248388	1.556933	-1.493685
29	6	0	5.229874	-0.192825	0.179923
30	1	0	5.808666	0.730712	0.260379
31	1	0	5.397928	-0.804202	1.069841
32	1	0	5.555718	-0.749301	-0.702139

Difluoromethyl phenyl sulfone Conformer B – Me$_3$PO Complex

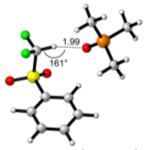

Standard orientation:

Center Number	Atomic Number	Atomic Type	X	Y	Z
1	6	0	1.682585	3.390086	0.289048
2	6	0	2.757125	2.642542	0.773409
3	6	0	2.830626	1.277469	0.508136
4	6	0	1.815992	0.695594	-0.249825
5	6	0	0.671759	2.783678	-0.458867
6	1	0	3.537948	3.120853	1.354447
7	1	0	3.656096	0.672855	0.868301
8	1	0	-0.159395	3.374822	-0.828643
9	1	0	1.631654	4.453960	0.496704
10	6	0	0.727383	1.419775	-0.737086
11	1	0	-0.060300	0.920548	-1.295678
12	16	0	1.906727	-1.038191	-0.600047
13	6	0	0.660066	-1.771635	0.546372
14	1	0	-0.346138	-1.426443	0.278231
15	9	0	0.782770	-3.109848	0.429705
16	9	0	1.001963	-1.423875	1.804794
17	8	0	1.400647	-1.307175	-1.947266
18	8	0	3.219099	-1.543295	-0.191486
19	15	0	-3.047472	0.066738	-0.037266
20	8	0	-1.823308	-0.532338	-0.708990
21	6	0	-3.360346	-0.643354	1.599652
22	1	0	-2.489487	-0.468212	2.236634
23	1	0	-4.241106	-0.187030	2.058128
24	1	0	-3.519303	-1.719736	1.499531
25	6	0	-4.563630	-0.181950	-0.995475
26	1	0	-5.422584	0.260129	-0.484589
27	1	0	-4.445854	0.283772	-1.976758
28	1	0	-4.730979	-1.253597	-1.126453
29	6	0	-2.898104	1.853862	0.217082
30	1	0	-3.794071	2.252562	0.699388
31	1	0	-2.027531	2.052089	0.847843
32	1	0	-2.759345	2.342978	-0.750575

2-(Difluoromethyl)-1-methyl-benzoimidazole Conformer A – Me$_3$PO Complex

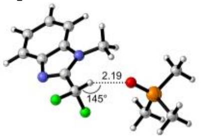

Standard orientation:

Center Number	Atomic Number	Atomic Type	X	Y	Z
1	6	0	-4.590570	0.538018	0.421604
2	6	0	-3.215591	0.522374	0.148280
3	6	0	-5.265313	-0.674398	0.409410
4	6	0	-2.565609	-0.699265	-0.122516
5	6	0	-3.237899	-1.925521	-0.142101
6	1	0	-2.725892	-2.856640	-0.362381
7	6	0	-4.598629	-1.888219	0.130323
8	1	0	-5.165728	-2.813440	0.128107
9	1	0	-6.330328	-0.695701	0.616510
10	1	0	-5.102560	1.472022	0.630243
11	7	0	-1.251888	-0.371092	-0.373560
12	7	0	-2.308543	1.563787	0.063954
13	6	0	-1.172821	0.985645	-0.250544
14	6	0	-0.226449	-1.315945	-0.788217
15	1	0	-0.101566	-2.077714	-0.014072
16	1	0	-0.530711	-1.788390	-1.725470
17	1	0	0.717989	-0.794676	-0.947405
18	6	0	0.128511	1.727846	-0.360894
19	1	0	0.876647	1.259124	-0.996174
20	9	0	-0.111107	2.997810	-0.790452
21	9	0	0.659418	1.851819	0.906298
22	15	0	3.644876	-0.474737	0.028835
23	8	0	2.781662	0.171300	-1.038514
24	6	0	5.323772	0.203923	0.089382
25	1	0	5.270000	1.274464	0.301045
26	1	0	5.912859	-0.290717	0.865616
27	1	0	5.804303	0.055052	-0.880532
28	6	0	3.842047	-2.260419	-0.216407
29	1	0	4.469125	-2.692433	0.567459
30	1	0	2.858196	-2.736184	-0.195338
31	1	0	4.303269	-2.439562	-1.190532
32	6	0	2.968364	-0.281555	1.698872
33	1	0	3.600651	-0.788376	2.432180
34	1	0	2.904378	0.781733	1.939858
35	1	0	1.962272	-0.708326	1.727957

2-(Difluoromethyl)-1-methyl-benzoimidazole Conformer B – Me$_3$PO Complex

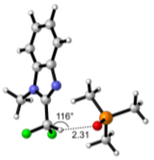

Standard orientation:

Center Number	Atomic Number	Atomic Type	X	Y	Z
1	6	0	-2.707800	-1.668755	-1.267653
2	6	0	-1.966786	-0.554261	-0.848574
3	6	0	-3.740471	-2.100647	-0.449860
4	6	0	-2.281647	0.087705	0.367062
5	6	0	-3.321640	-0.342284	1.199050
6	1	0	-3.552138	0.154670	2.135500
7	6	0	-4.042089	-1.445865	0.765730
8	1	0	-4.857555	-1.818438	1.377113
9	1	0	-4.332745	-2.960874	-0.744058
10	1	0	-2.471855	-2.169434	-2.201154
11	7	0	-1.381790	1.125439	0.479808
12	7	0	-0.890427	0.074142	-1.447262
13	6	0	-0.580718	1.046095	-0.625260
14	6	0	-1.321348	2.076702	1.581195
15	1	0	-2.337895	2.369567	1.848049
16	1	0	-0.830199	1.627451	2.447985
17	1	0	-0.770338	2.961032	1.267920
18	6	0	0.565952	1.976428	-0.864717
19	1	0	1.201093	1.629352	-1.676614
20	9	0	0.102930	3.241963	-1.124357
21	9	0	1.319961	2.088245	0.274644
22	15	0	2.725220	-0.965177	0.073053
23	8	0	2.543728	-0.192034	-1.216928
24	6	0	3.872128	-0.161060	1.224265
25	1	0	3.495412	0.838724	1.453584
26	1	0	3.963505	-0.737451	2.148356
27	1	0	4.853709	-0.073986	0.752274
28	6	0	3.385014	-2.632980	-0.196086
29	1	0	3.531629	-3.151549	0.754532
30	1	0	2.684854	-3.200034	-0.814377
31	1	0	4.341354	-2.557271	-0.719108
32	6	0	1.191846	-1.202243	1.016706
33	1	0	1.370840	-1.864241	1.868104
34	1	0	0.837542	-0.235417	1.383936
35	1	0	0.429515	-1.640119	0.366040

2-(Difluoromethyl)quinoline Conformer A – Me$_3$PO Complex

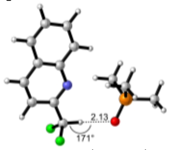

Standard orientation:

Center Number	Atomic Number	Atomic Type	Coordinates (Angstroms) X	Y	Z
1	6	0	3.026086	-0.158481	0.088597
2	6	0	3.225621	1.242131	0.210586
3	6	0	2.145908	2.082094	0.160694
4	6	0	0.858937	1.508453	-0.011540
5	6	0	1.694269	-0.627096	-0.083132
6	1	0	2.250891	3.157723	0.250494
7	7	0	0.625607	0.223485	-0.128240
8	6	0	-0.352605	2.401291	-0.087890
9	9	0	-0.346713	3.277365	0.968905
10	9	0	-0.267779	3.186482	-1.213277
11	1	0	-1.298360	1.853018	-0.096082
12	6	0	4.091593	-1.095651	0.130325
13	6	0	1.457722	-2.021015	-0.211422
14	6	0	2.508029	-2.905216	-0.168265
15	1	0	2.324546	-3.970135	-0.267252
16	6	0	3.836848	-2.439556	0.004730
17	1	0	4.653377	-3.153341	0.036718
18	1	0	0.435313	-2.361412	-0.344812
19	1	0	5.106182	-0.730752	0.262401
20	1	0	4.232289	1.628686	0.341922
21	15	0	-3.035939	-0.721467	0.068930
22	8	0	-3.118469	0.787235	0.211696
23	6	0	-4.663173	-1.520793	0.118338
24	1	0	-5.279801	-1.130142	-0.694784
25	1	0	-4.564527	-2.603889	0.009418
26	1	0	-5.145884	-1.295261	1.072368
27	6	0	-2.065964	-1.501884	1.385751
28	1	0	-2.009449	-2.583865	1.239255
29	1	0	-1.060104	-1.074337	1.371566
30	1	0	-2.537211	-1.291385	2.349086
31	6	0	-2.278901	-1.240130	-1.493199
32	1	0	-2.221586	-2.330263	-1.554693
33	1	0	-2.879833	-0.861896	-2.324018
34	1	0	-1.274046	-0.812508	-1.543534

4-(Difluoromethyl)quinoline Conformer A – Me$_3$PO Complex

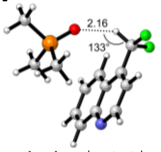

Standard orientation:

Center Number	Atomic Number	Atomic Type	Coordinates (Angstroms) X	Y	Z
1	6	0	-1.259227	0.437172	-0.218354
2	6	0	-1.364561	-0.923957	0.193724
3	6	0	-1.581956	-1.209726	1.514552
4	6	0	-1.693704	-0.136650	2.437613
5	6	0	-1.379560	1.433080	0.790740
6	1	0	-1.659825	-2.232673	1.863011
7	7	0	-1.593688	1.129189	2.107902
8	1	0	-1.867205	-0.356002	3.488496
9	6	0	-1.222876	-2.018813	-0.829655
10	9	0	-1.298890	-3.244521	-0.232127
11	9	0	-2.275667	-1.960272	-1.713332
12	1	0	-0.285699	-1.958160	-1.384299
13	6	0	-1.045761	0.840389	-1.564750
14	6	0	-1.282967	2.803947	0.430435
15	6	0	-1.086323	3.165334	-0.879754
16	1	0	-1.017871	4.213945	-1.150463
17	6	0	-0.966912	2.173602	-1.885862
18	1	0	-0.803890	2.472287	-2.916120
19	1	0	-1.375796	3.544289	1.218558
20	1	0	-0.944091	0.090282	-2.341809
21	15	0	2.479770	-0.220923	-0.020752
22	8	0	1.651235	-1.102683	-0.934133
23	6	0	1.979178	-0.313869	1.719448
24	1	0	1.976024	-1.358209	2.041295
25	1	0	2.667200	0.259339	2.346409
26	1	0	0.971246	0.096215	1.827341
27	6	0	2.391223	1.539905	-0.439260
28	1	0	3.048984	2.121005	0.212357
29	1	0	2.691436	1.681146	-1.480484
30	1	0	1.361450	1.886839	-0.315549
31	6	0	4.241394	-0.649778	-0.049749
32	1	0	4.812262	0.004763	0.613311
33	1	0	4.361984	-1.686973	0.272232
34	1	0	4.616496	-0.547791	-1.070911

2-(Difluoromethyl)quinoline Conformer B – Me$_3$PO Complex

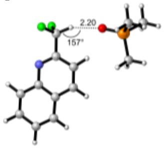

Standard orientation:

Center Number	Atomic Number	Atomic Type	Coordinates (Angstroms) X	Y	Z
1	6	0	2.583151	-1.167292	0.024817
2	6	0	1.183195	-1.379555	0.062262
3	6	0	0.339439	-0.297390	0.064173
4	6	0	0.913808	0.995609	0.027460
5	6	0	3.051865	0.178082	-0.009859
6	1	0	-0.742316	-0.388749	0.092140
7	7	0	2.202578	1.243020	-0.008106
8	6	0	-0.006609	2.193787	0.027215
9	9	0	0.281822	3.005005	1.093939
10	9	0	0.218412	2.955007	-1.090790
11	1	0	-1.063923	1.922528	0.062880
12	6	0	3.525638	-2.231713	0.020597
13	6	0	4.451794	0.424508	-0.047715
14	6	0	5.337184	-0.623567	-0.050897
15	1	0	6.404863	-0.431445	-0.079994
16	6	0	4.870995	-1.965210	-0.016452
17	1	0	5.587620	-2.779965	-0.019604
18	1	0	4.788921	1.455830	-0.073634
19	1	0	3.160034	-3.254368	0.047158
20	1	0	0.795234	-2.394446	0.089100
21	15	0	-3.820559	-0.542648	-0.005305
22	8	0	-2.804725	0.581949	0.074053
23	6	0	-4.856752	-0.457735	-1.488958
24	1	0	-4.218842	-0.504274	-2.374770
25	1	0	-5.568394	-1.286849	-1.509904
26	1	0	-5.402412	0.488846	-1.490981
27	6	0	-4.965022	-0.564586	1.398977
28	1	0	-5.675962	-1.389304	1.304447
29	1	0	-4.395052	-0.680354	2.323862
30	1	0	-5.510160	0.381747	1.431395
31	6	0	-3.044196	-2.181523	-0.037467
32	1	0	-3.801862	-2.966852	-0.096714
33	1	0	-2.385081	-2.251033	-0.906985
34	1	0	-2.453552	-2.318921	0.872234

4-(Difluoromethyl)quinoline Conformer B – Me$_3$PO Complex

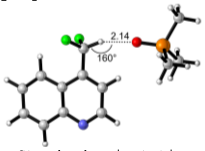

Standard orientation:

Center Number	Atomic Number	Atomic Type	Coordinates (Angstroms) X	Y	Z
1	6	0	-2.488343	-0.067203	-0.003610
2	6	0	-1.070470	-0.223429	-0.026259
3	6	0	-0.267376	0.885961	-0.043519
4	6	0	-0.874522	2.169468	-0.037839
5	6	0	-2.987658	1.266041	-0.000264
6	1	0	0.813967	0.778044	-0.061913
7	7	0	-2.171098	2.364174	-0.017227
8	1	0	-0.246463	3.057371	-0.051092
9	6	0	-0.415553	-1.576041	-0.032006
10	9	0	-0.802856	-2.297768	1.071750
11	9	0	-0.838770	-2.306185	-1.116819
12	1	0	0.674388	-1.518520	-0.049937
13	6	0	-3.410189	-1.149228	0.015155
14	6	0	-4.390493	1.483942	0.021457
15	6	0	-5.258560	0.420539	0.039247
16	1	0	-6.329896	0.592331	0.055826
17	6	0	-4.762508	-0.907014	0.036039
18	1	0	-5.459093	-1.738892	0.050137
19	1	0	-4.745240	2.509695	0.023407
20	1	0	-3.038652	-2.167696	0.012679
21	15	0	3.940539	0.106647	0.003895
22	8	0	2.638165	-0.665997	-0.097032
23	6	0	5.400798	-0.964780	0.042069
24	1	0	5.429451	-1.564089	-0.870976
25	1	0	6.314250	-0.369091	0.113731
26	1	0	5.333721	-1.631273	0.905186
27	6	0	4.032665	1.129405	1.497269
28	1	0	4.977781	1.676889	1.534552
29	1	0	3.202283	1.840150	1.494810
30	1	0	3.949917	0.487571	2.377616
31	6	0	4.189715	1.241283	-1.386795
32	1	0	5.130735	1.786183	-1.278336
33	1	0	4.206152	0.669596	-2.317733
34	1	0	3.360583	1.952686	-1.419590

9-(Difluoromethyl)acridine – Me_3PO Complex

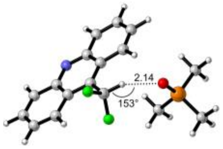

Standard orientation:

Center Number	Atomic Number	Atomic Type	Coordinates (Angstroms) X	Y	Z
1	6	0	-0.564713	1.084585	0.466503
2	6	0	-0.990570	-0.252527	0.514902
3	6	0	-2.274806	-0.596746	0.055330
4	6	0	-3.098365	0.456898	-0.469636
5	6	0	-1.482930	2.053008	-0.078242
6	7	0	-2.699650	1.734381	-0.530015
7	6	0	-0.083038	-1.323315	1.069523
8	9	0	-0.672390	-1.913135	2.161745
9	9	0	0.077807	-2.326600	0.143013
10	1	0	0.916095	-0.996114	1.352193
11	6	0	0.715252	1.550759	0.927189
12	6	0	-1.092637	3.431096	-0.144847
13	6	0	0.129115	3.830724	0.306854
14	1	0	0.417470	4.875629	0.257297
15	6	0	1.041158	2.874446	0.850518
16	1	0	2.008984	3.209960	1.210045
17	1	0	-1.808225	4.131635	-0.562863
18	1	0	1.442360	0.853158	1.330057
19	6	0	-4.410827	0.146744	-0.952333
20	6	0	-2.810537	-1.927439	0.085460
21	6	0	-4.069124	-2.180960	-0.380282
22	1	0	-4.460739	-3.192354	-0.348714
23	6	0	-4.881480	-1.132496	-0.909348
24	1	0	-5.877224	-1.361795	-1.274676
25	1	0	-5.005677	0.964926	-1.345229
26	1	0	-2.210798	-2.735750	0.484418
27	15	0	3.583170	-0.582951	-0.375594
28	8	0	3.019854	-0.812882	1.015575
29	6	0	4.833618	-1.807402	-0.841276
30	1	0	4.386011	-2.803702	-0.816490
31	1	0	5.216825	-1.606042	-1.844649
32	1	0	5.656888	-1.767391	-0.124120
33	6	0	4.377922	1.035976	-0.553091
34	1	0	4.776995	1.163231	-1.562488
35	1	0	3.639359	1.817412	-0.356342
36	1	0	5.189899	1.120064	0.173149
37	6	0	2.313051	-0.648012	-1.667539
38	1	0	2.755383	-0.474317	-2.651784
39	1	0	1.831553	-1.628419	-1.650526
40	1	0	1.560712	0.122096	-1.470795

NMR spectra.

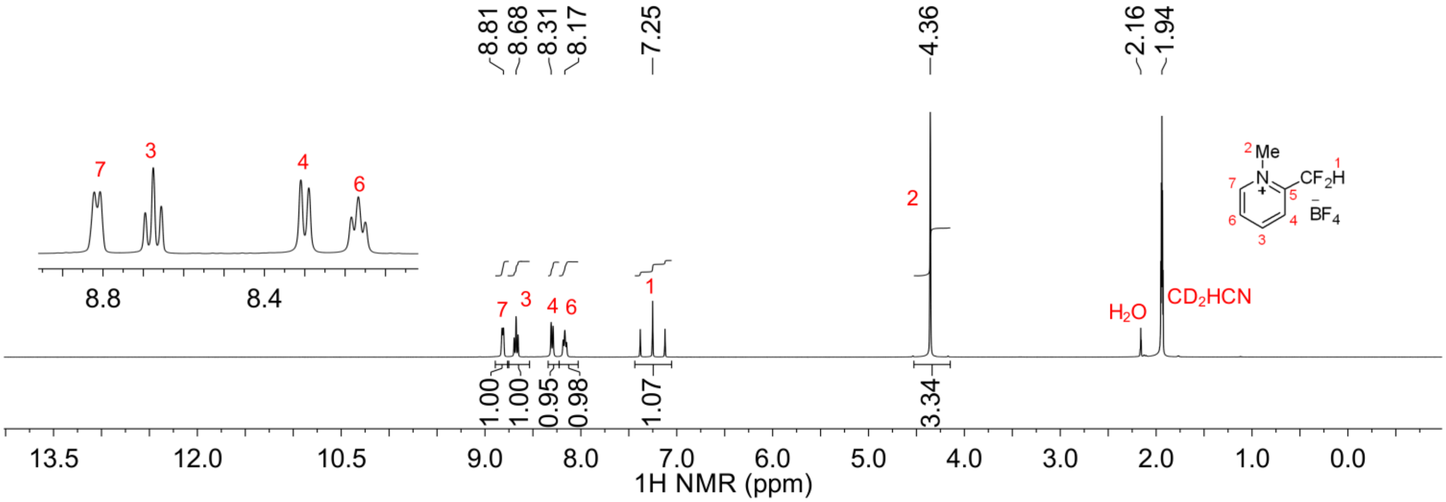

Figure S48. ^{1}H NMR spectrum of 2-(difluoromethyl)-*N*-methylpyridinium tetrafluoroborate (**1b**).

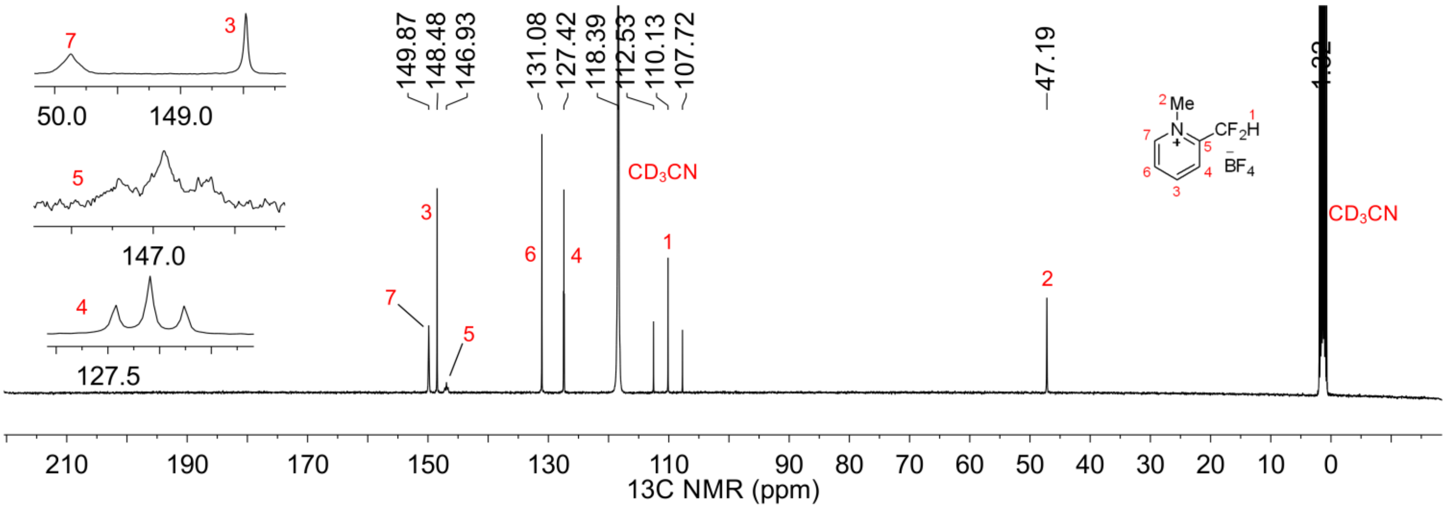

Figure S49. ^{13}C{^{1}H} NMR spectrum of 2-(difluoromethyl)-*N*-methylpyridinium tetrafluoroborate (**1b**).

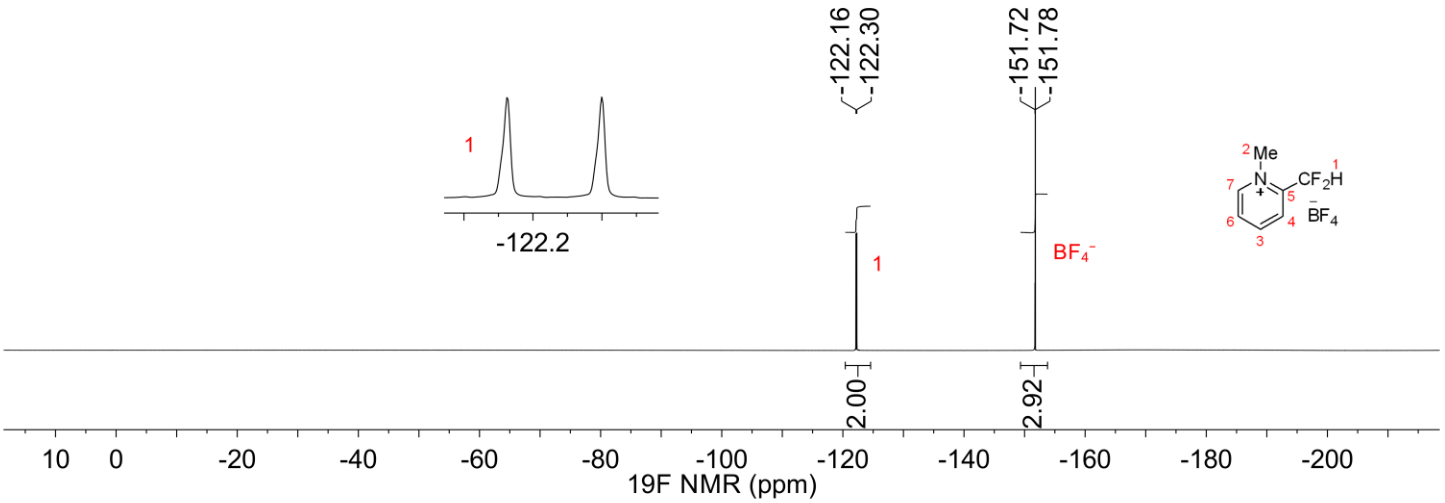

Figure S50. ^{19}F NMR spectrum of 2-(difluoromethyl)-*N*-methylpyridinium tetrafluoroborate (**1b**).

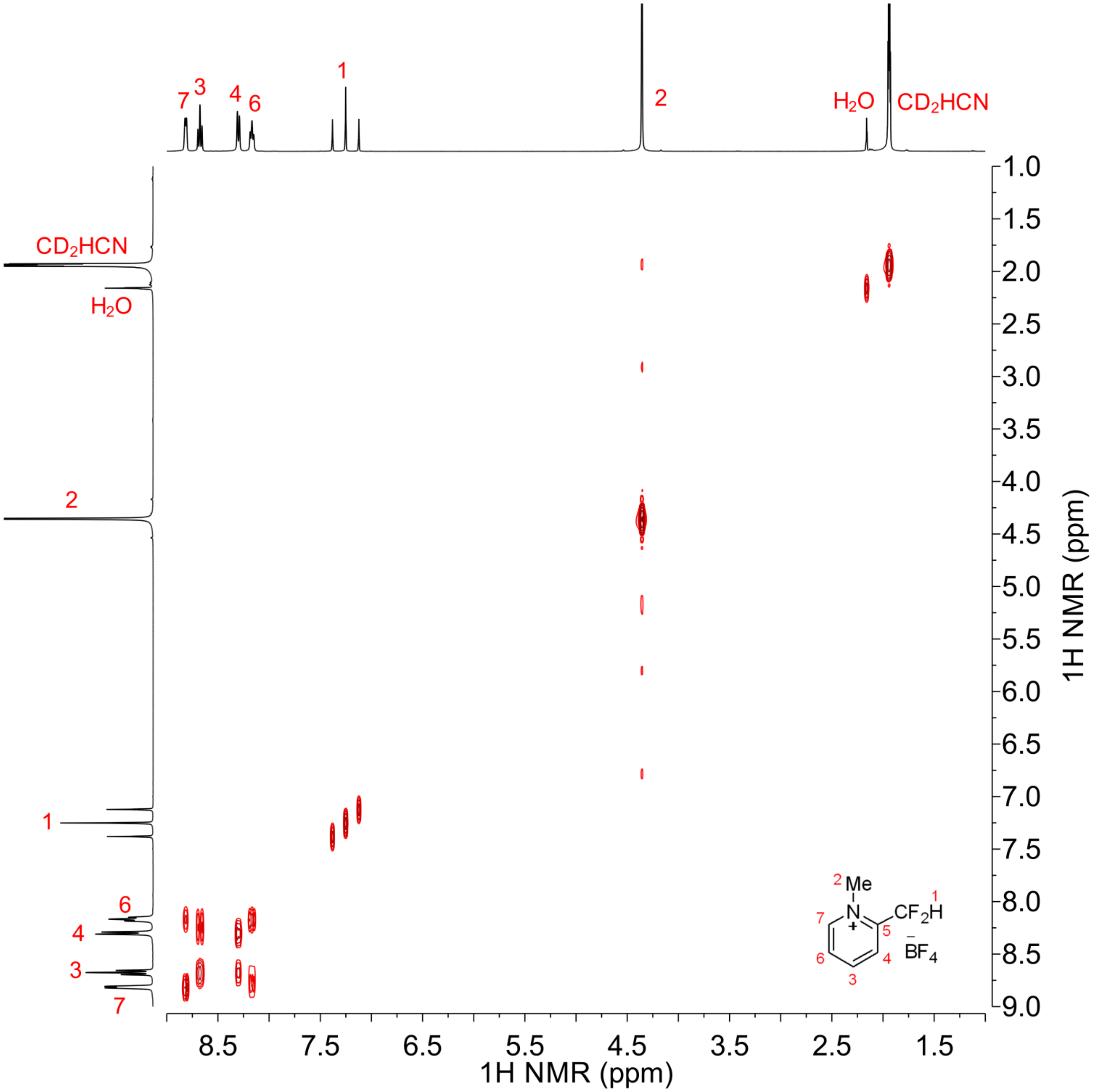

Figure S51. ^{1}H-^{1}H COSY spectrum of 2-(difluoromethyl)-*N*-methylpyridinium tetrafluoroborate (**1b**).

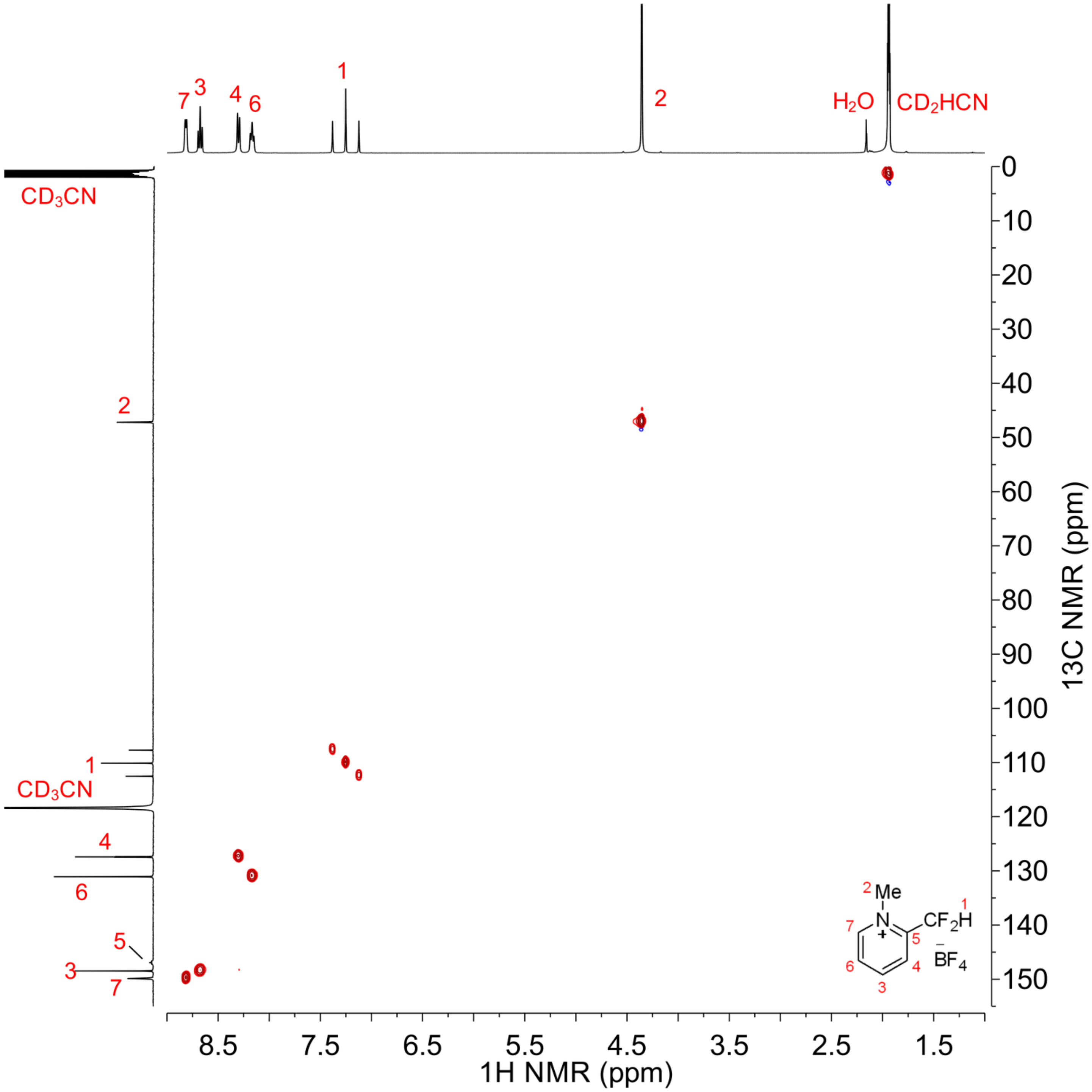

Figure S52. ^{1}H-^{13}C HSQC spectrum of 2-(difluoromethyl)-*N*-methylpyridinium tetrafluoroborate (**1b**).

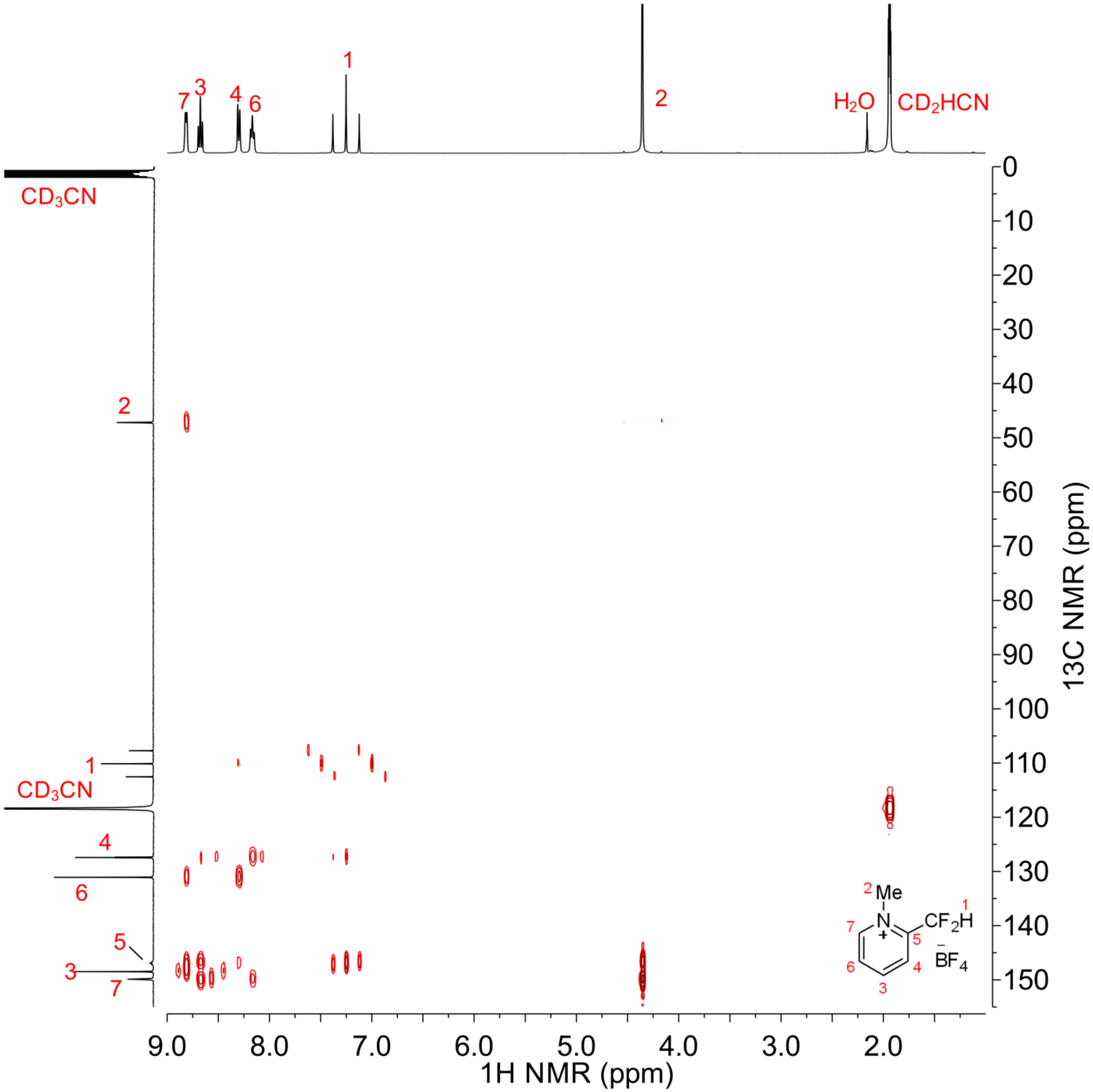

Figure S53. ^{1}H-^{13}C HMBC spectrum of 2-(difluoromethyl)-*N*-methylpyridinium tetrafluoroborate (**1b**).

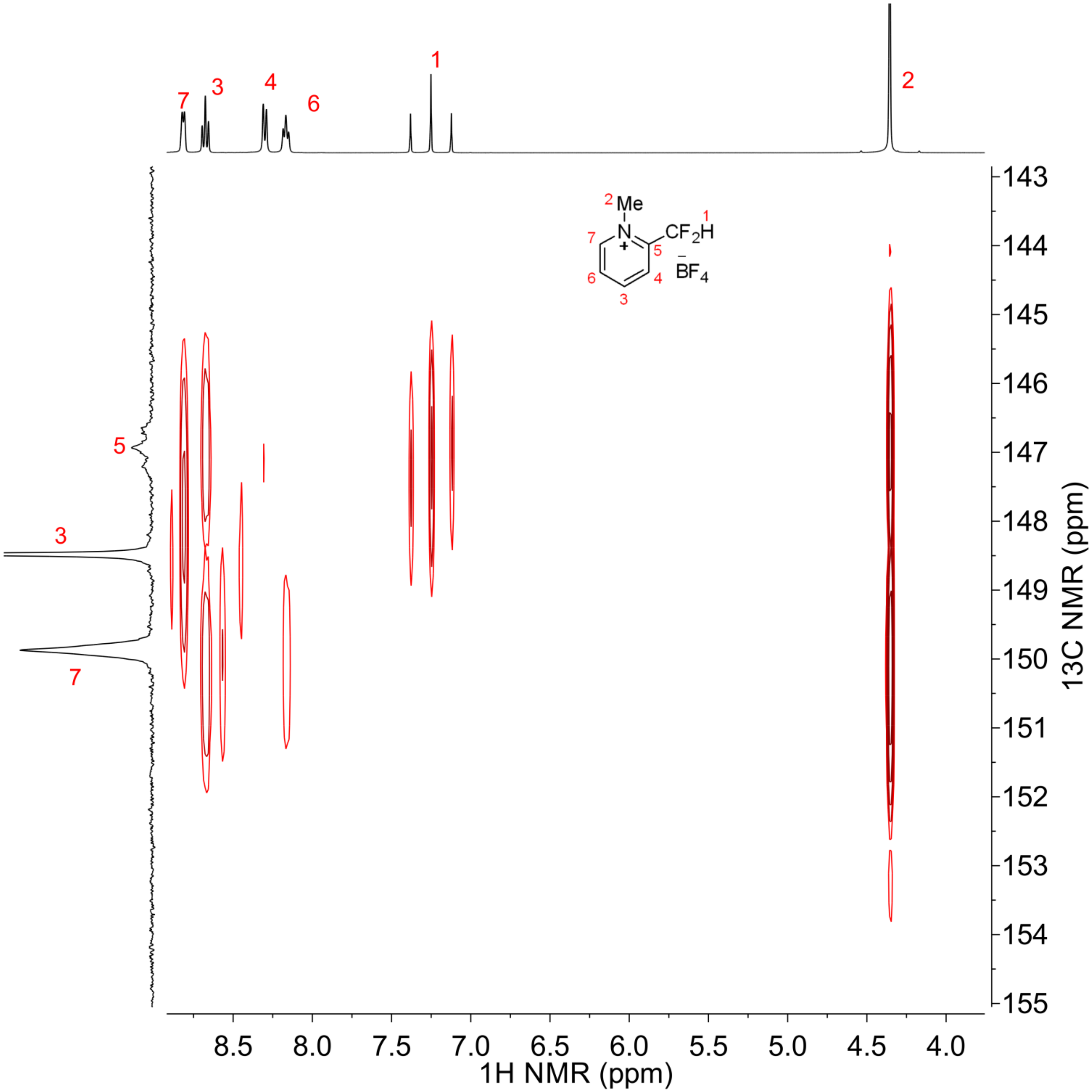

Figure S54. Expansion of ^{1}H-^{13}C HMBC spectrum of 2-(difluoromethyl)-*N*-methylpyridinium tetrafluoroborate (**1b**) from 4.2 to 8.7 ppm (^{1}H) and 143 to 155 ppm (^{13}C).

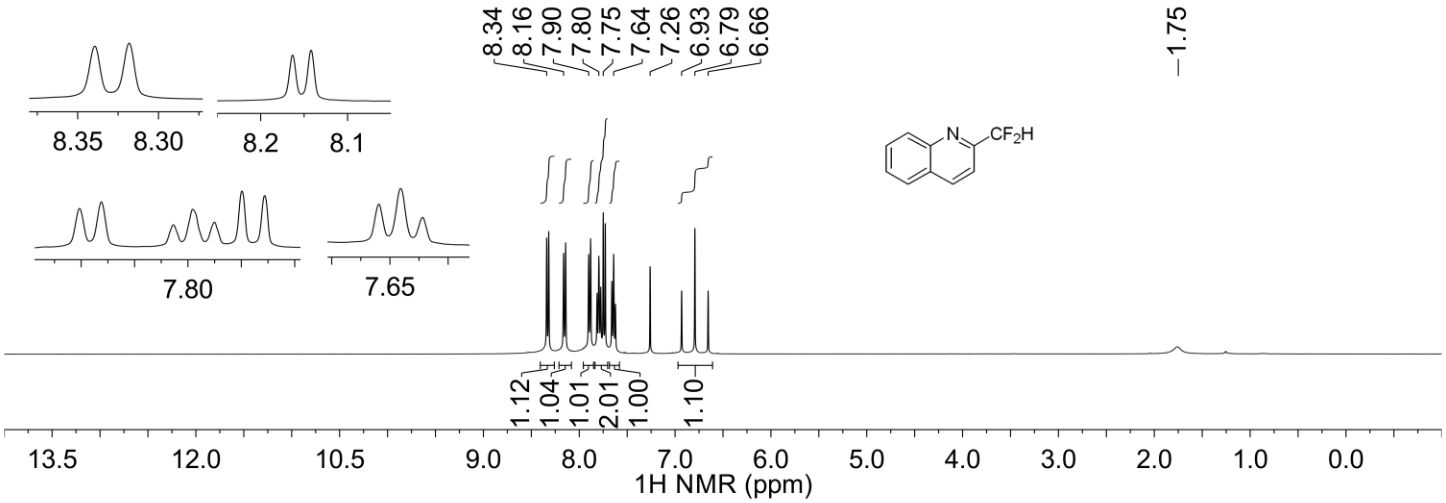

Figure S55. ^{1}H NMR spectrum of 2-(difluoromethyl)quinoline (**2a**).

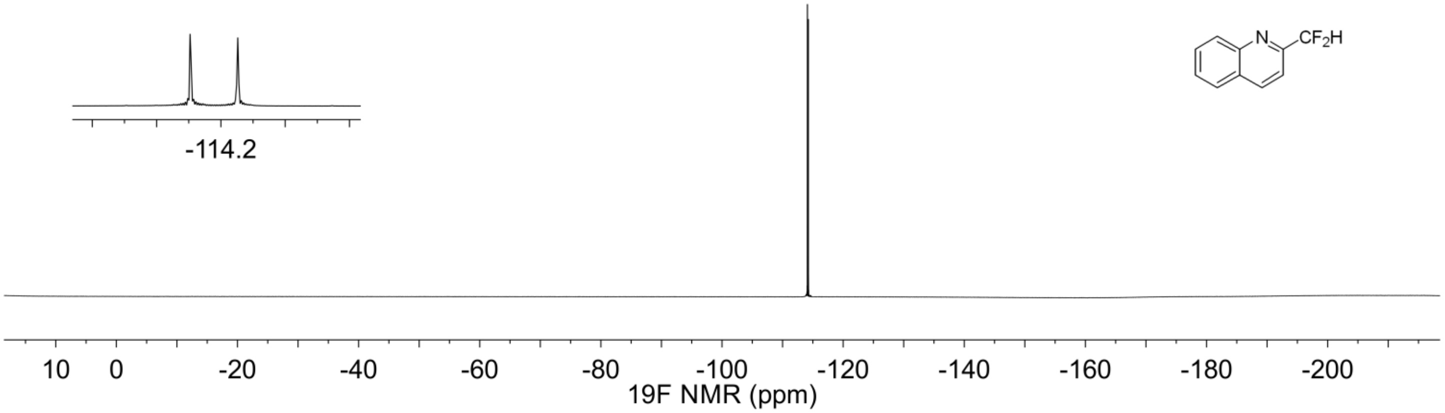

Figure S56. ^{19}F NMR spectrum of 2-(difluoromethyl)quinoline (**2a**).

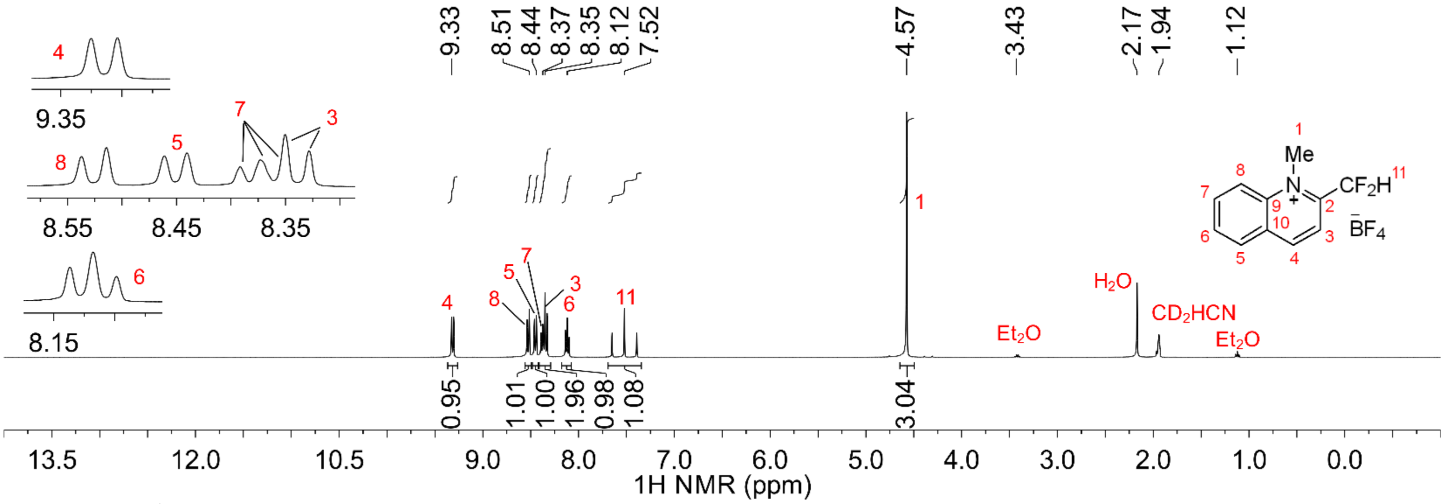

Figure S57. ^{1}H NMR spectrum of 2-(difluoromethyl)-*N*-methylquinolinium tetrafluoroborate (**2b**).

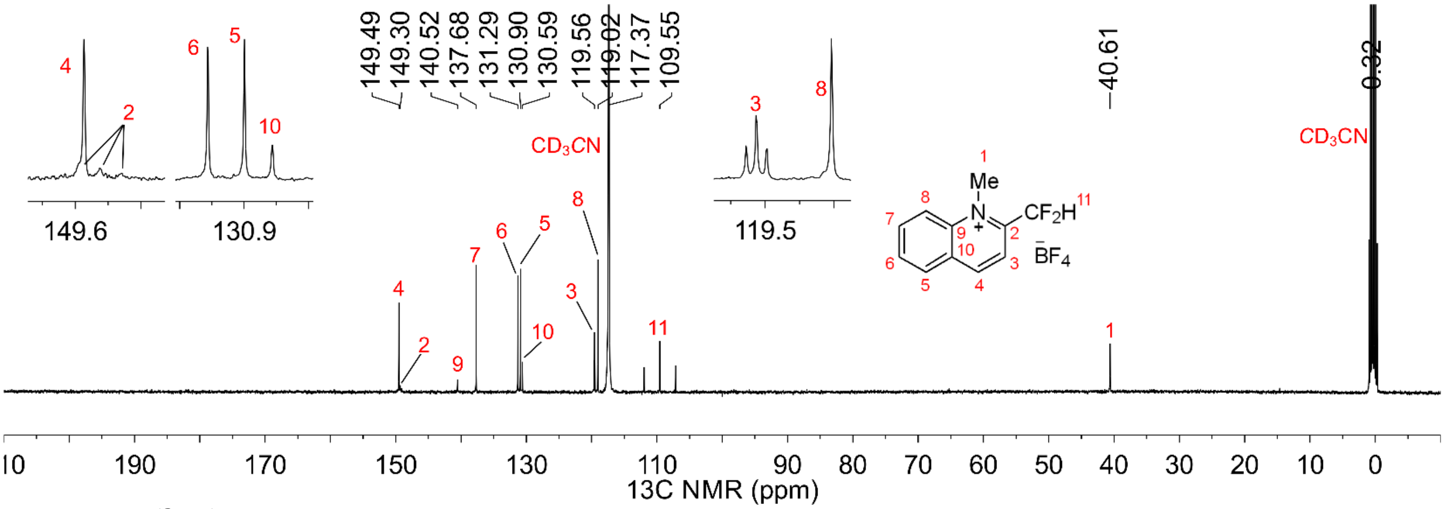

Figure S58. ^{13}C{^{1}H} NMR spectrum of 2-(difluoromethyl)-*N*-methylquinolinium tetrafluoroborate (**2b**).

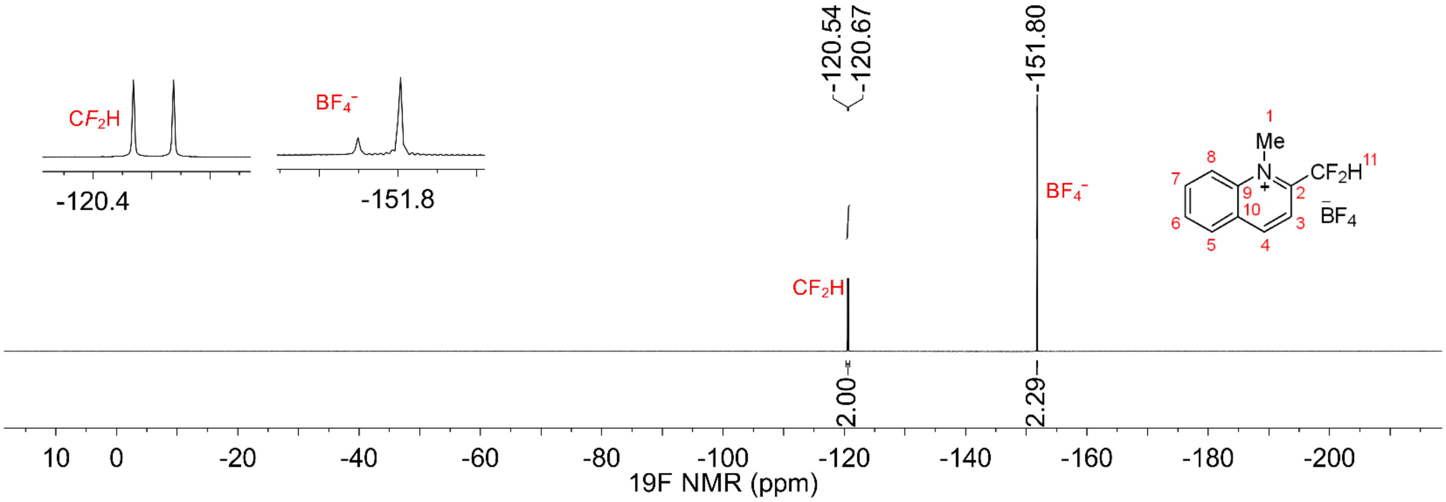

Figure S59. ^{19}F NMR spectrum of 2-(difluoromethyl)-*N*-methylquinolinium tetrafluoroborate (**2b**).

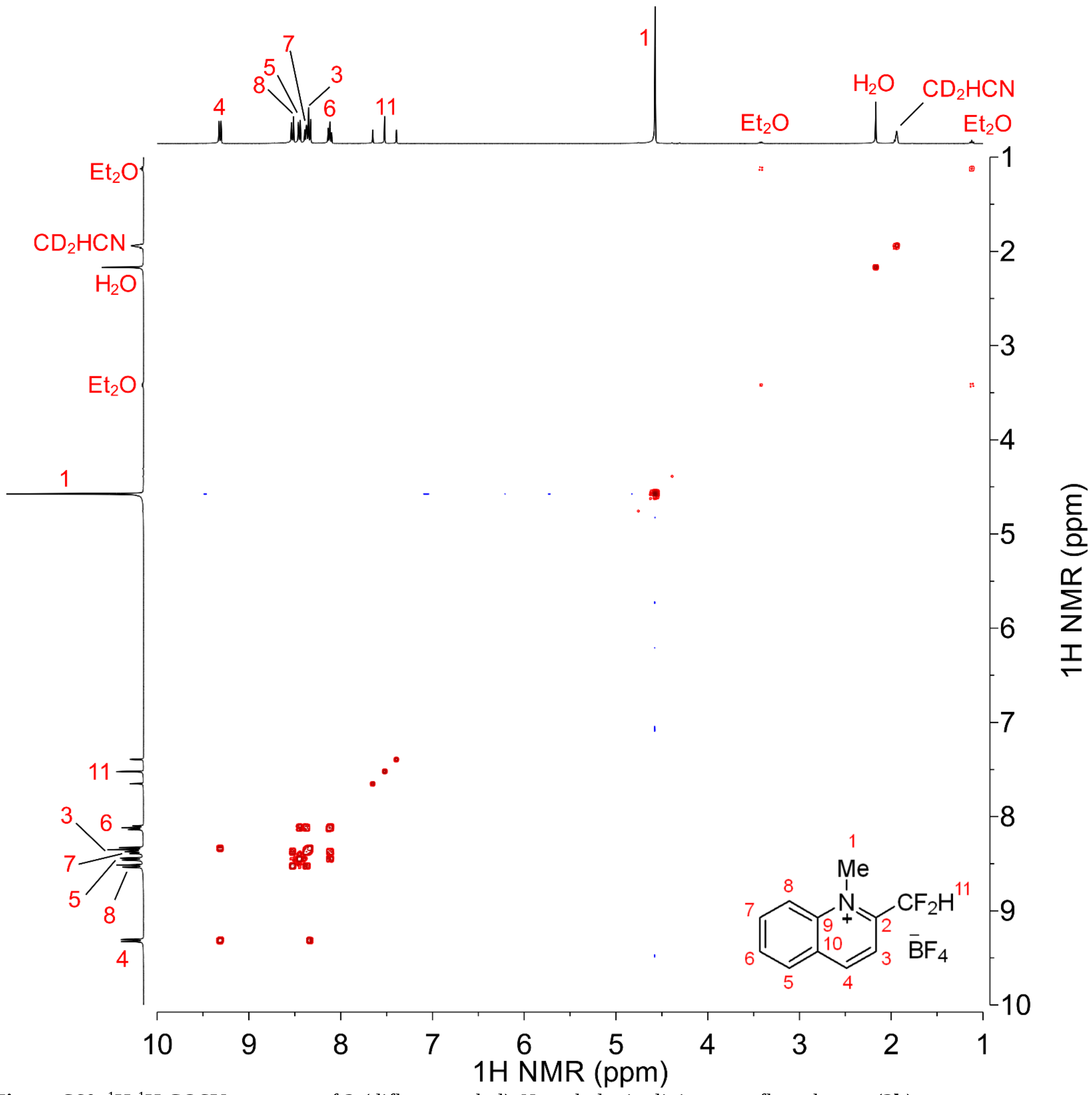

Figure S60. ^{1}H-^{1}H COSY spectrum of 2-(difluoromethyl)-*N*-methylquinolinium tetrafluoroborate (**2b**).

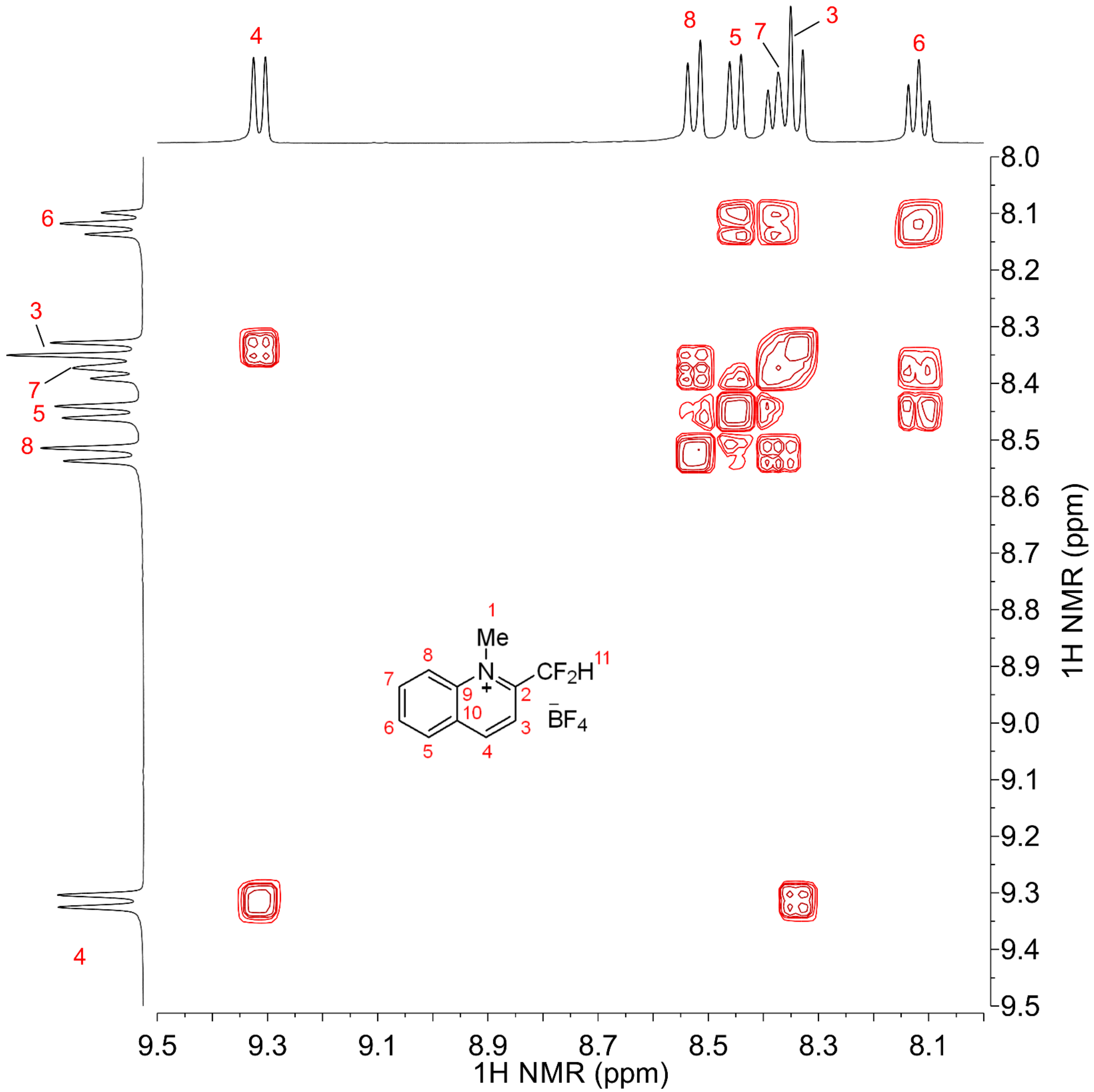

Figure S61. Expansion of ^{1}H-^{1}H COSY spectrum of 2-(difluoromethyl)-*N*-methylquinolinium tetrafluoroborate (**2b**) from 8.0 to 9.5 ppm.

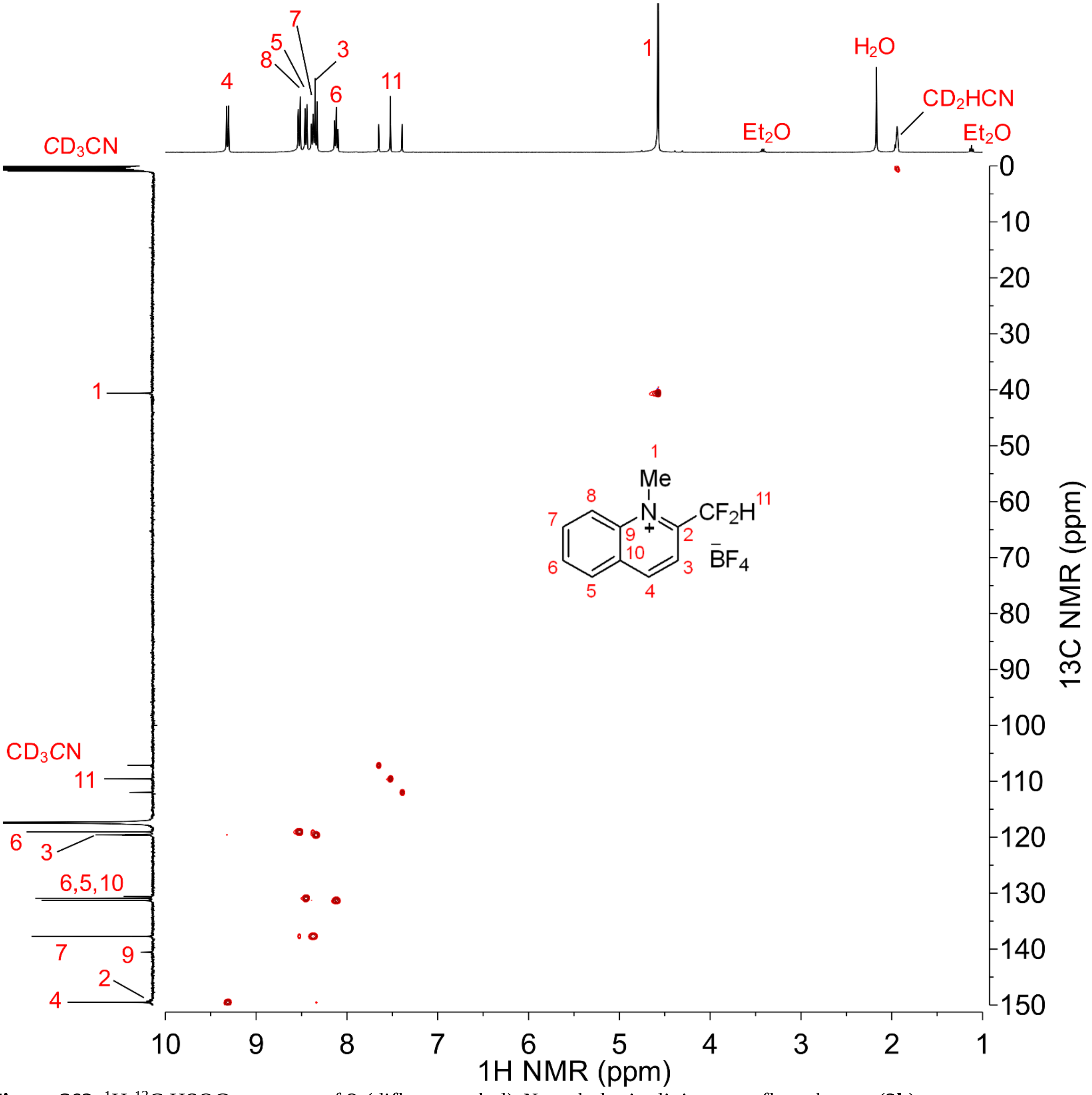

Figure S62. ^{1}H-^{13}C HSQC spectrum of 2-(difluoromethyl)-*N*-methylquinolinium tetrafluoroborate (**2b**).

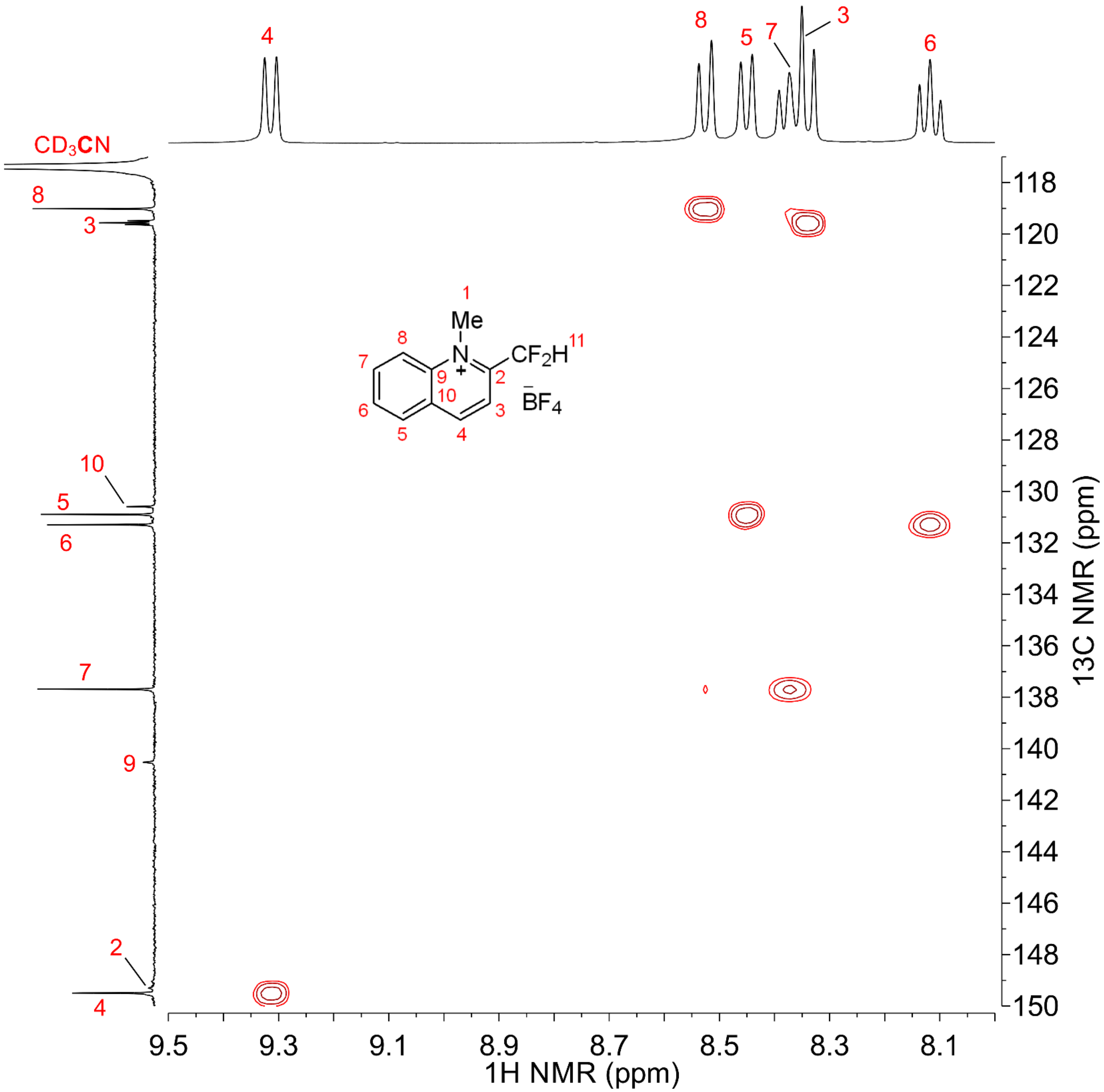

Figure S63. Expansion of ^{1}H-^{13}C HSQC spectrum of 2-(difluoromethyl)-*N*-methylquinolinium tetrafluoroborate (**2b**) from 8.0 to 9.5 ppm (^{1}H) and 117 to 150 ppm (^{13}C).

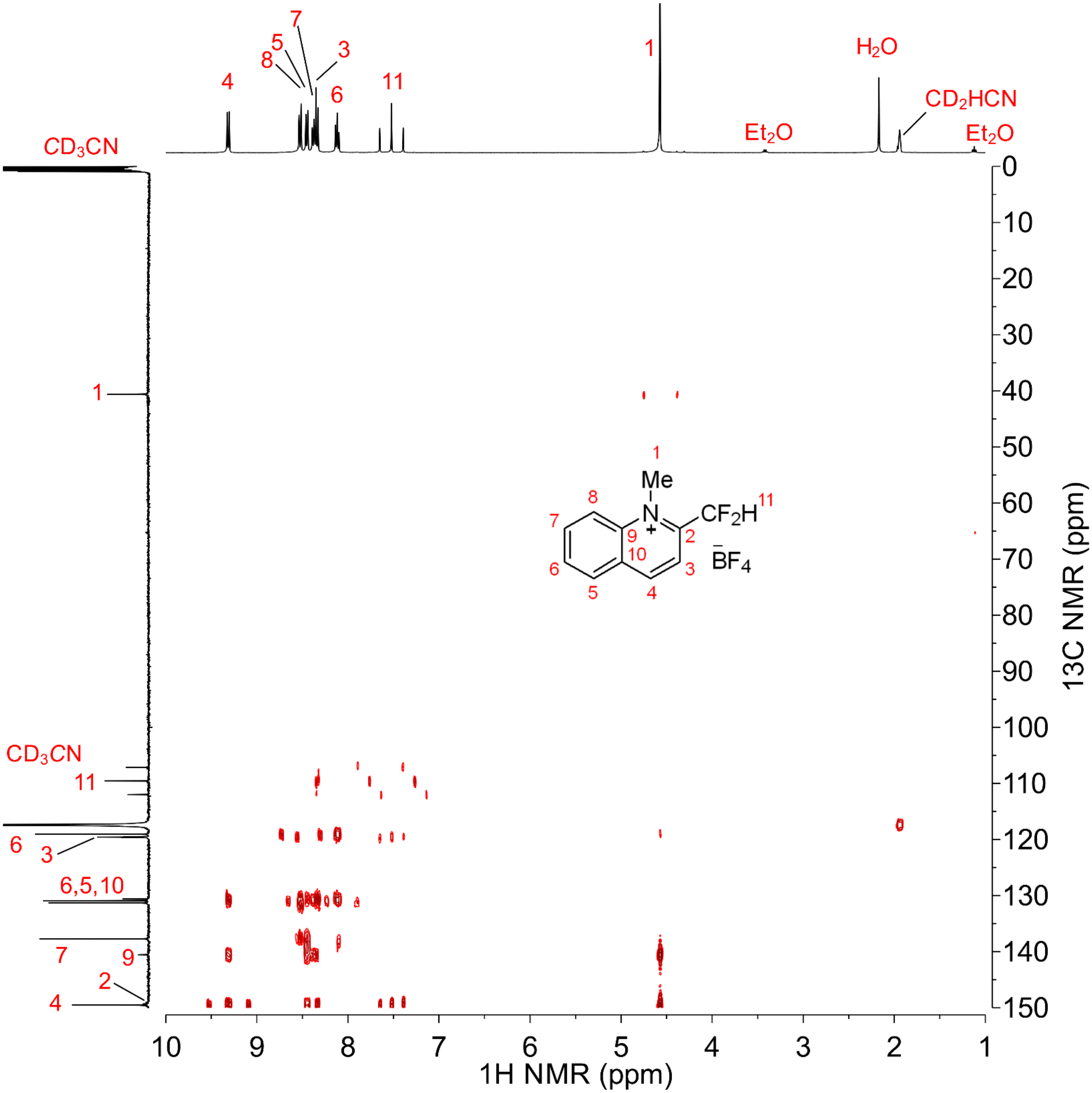

Figure S64. ^{1}H-^{13}C HMBC spectrum of 2-(difluoromethyl)-*N*-methylquinolinium tetrafluoroborate (**2b**).

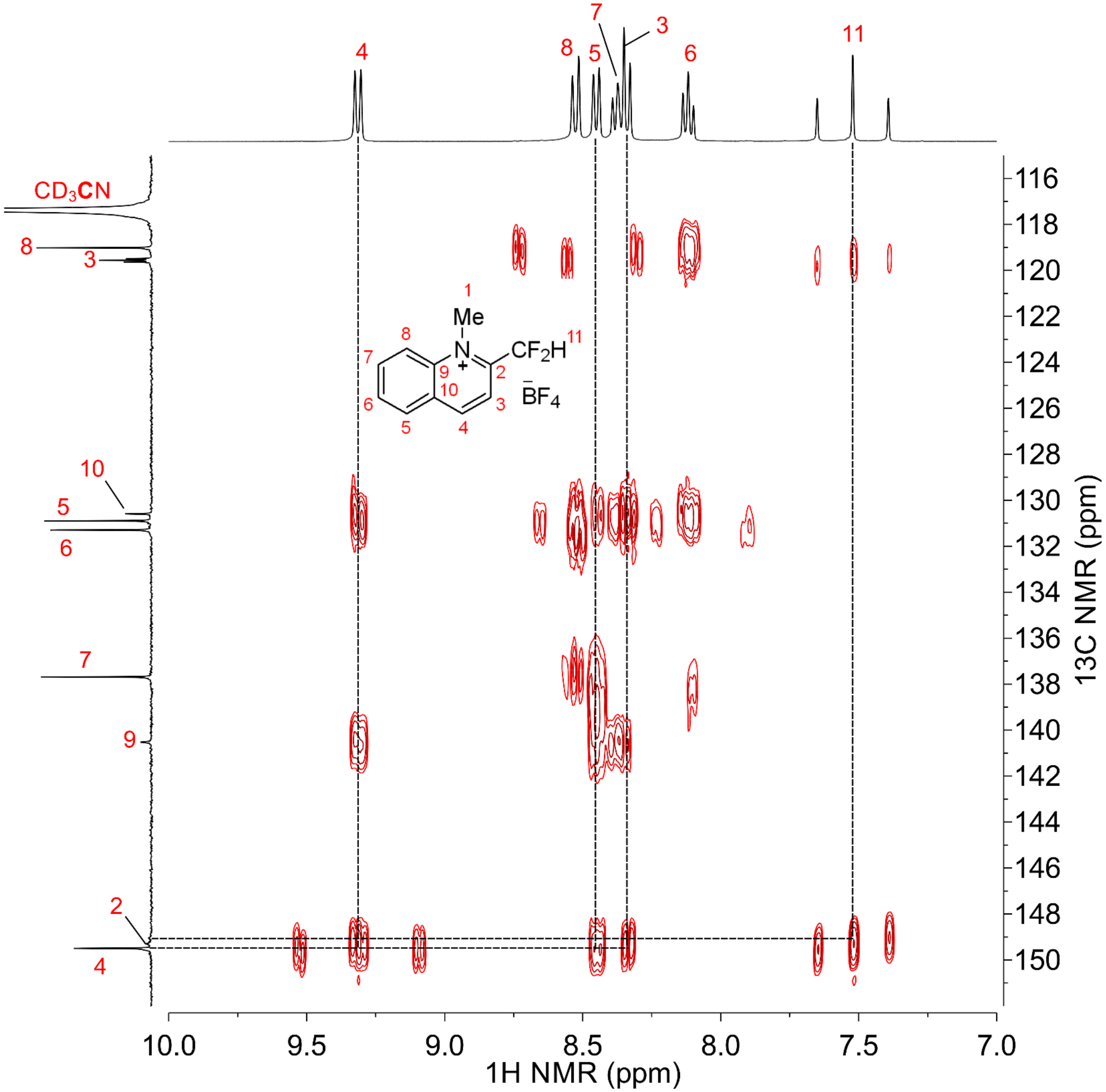

Figure S65. Expansion of ^{1}H-^{13}C HSQC spectrum of 2-(difluoromethyl)-*N*-methylquinolinium tetrafluoroborate (**2b**) from 7.5 to 10.0 ppm (^{1}H) and 115 to 160 ppm (^{13}C).

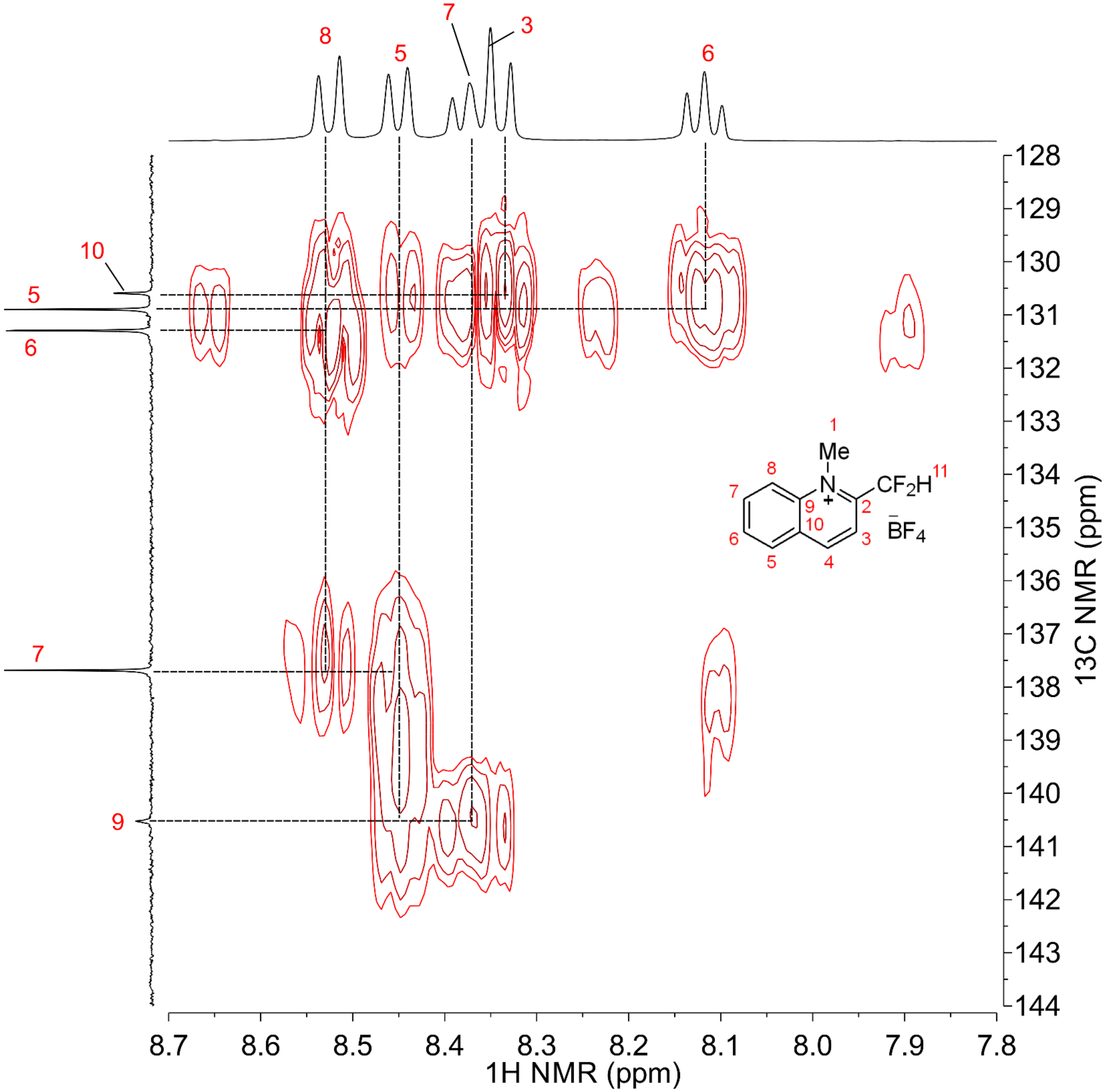

Figure S66. Expansion of ^{1}H-^{13}C HSQC spectrum of 2-(difluoromethyl)-*N*-methylquinolinium tetrafluoroborate (**2b**) from 7.8 to 8.7 ppm (^{1}H) and 128 to 144 ppm (^{13}C).

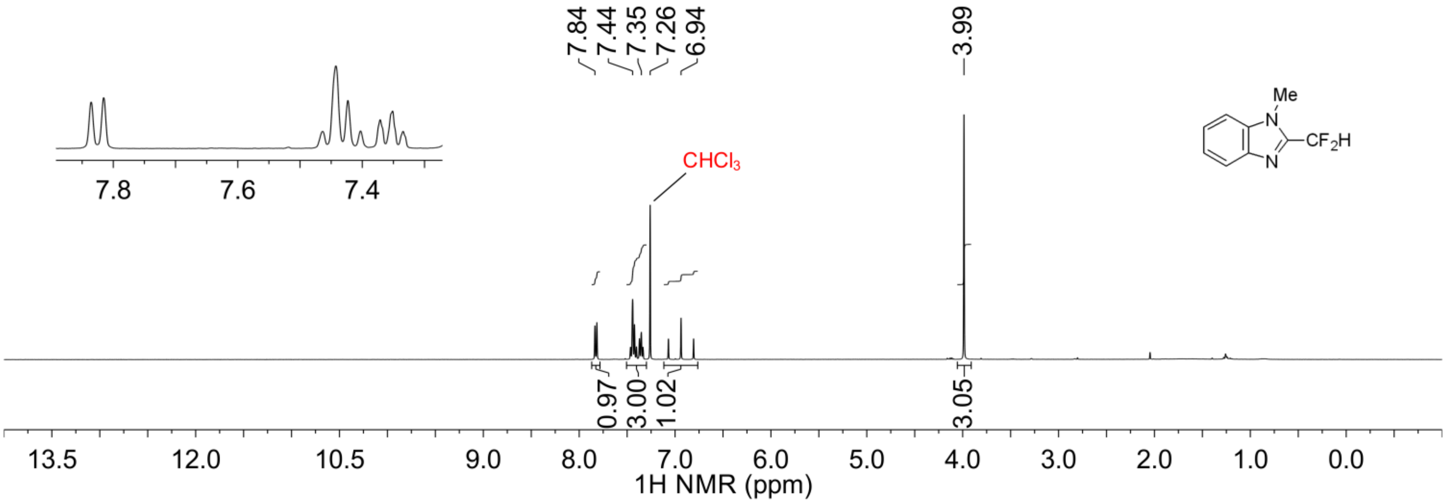

Figure S67. ^{1}H NMR spectrum of 2-(difluoromethyl)-1-methyl-benzoimidazole (**3a**).

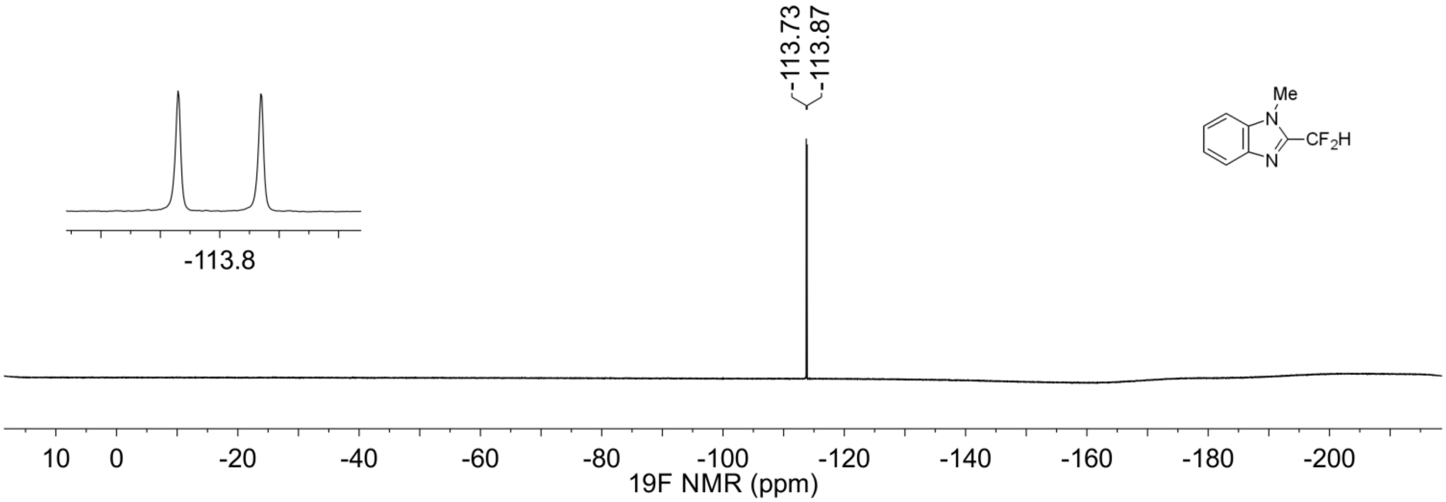

Figure S68. ^{19}F NMR spectrum of 2-(difluoromethyl)-1-methyl-benzoimidazole (**3a**).

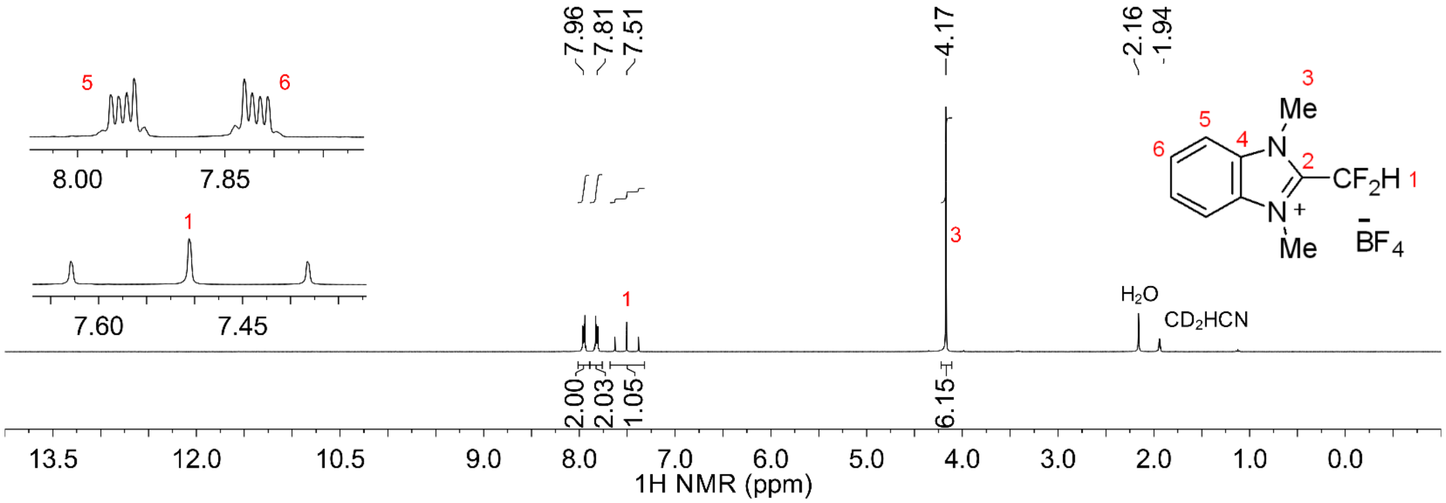

Figure S69. ^{1}H NMR spectrum of 2-(difluoromethyl)-1,3-dimethyl-benzoimidazolium tetrafluoroborate (**3b**).

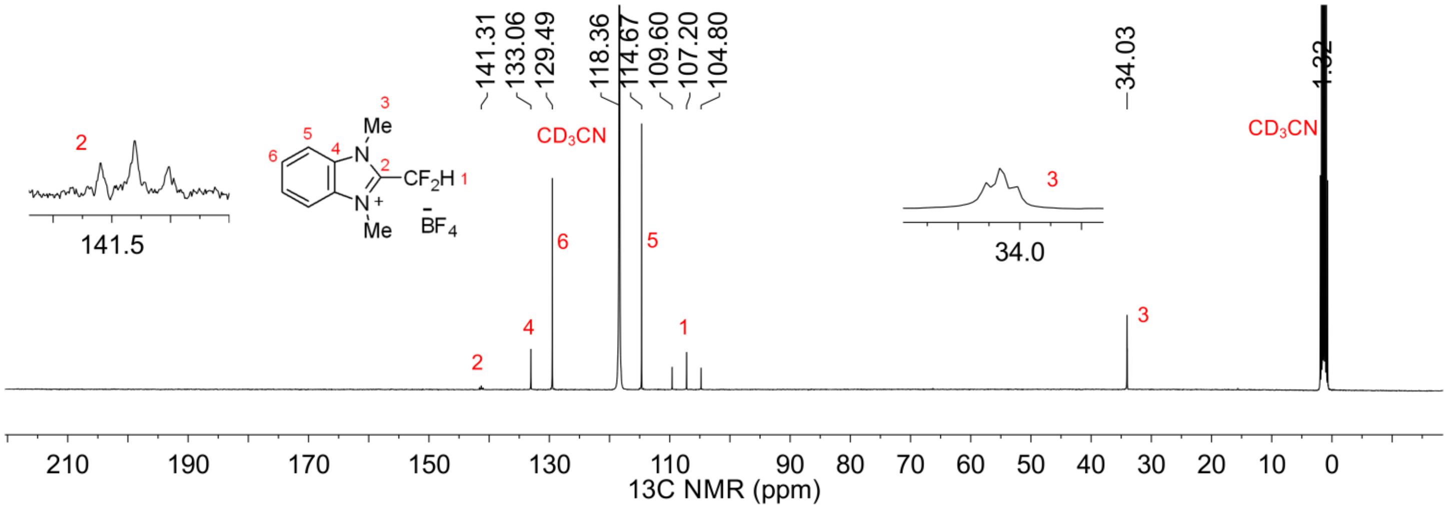

Figure S70. ^{13}C{^{1}H} NMR spectrum of 2-(difluoromethyl)-1,3-dimethyl-benzoimidazolium tetrafluoroborate (**3b**).

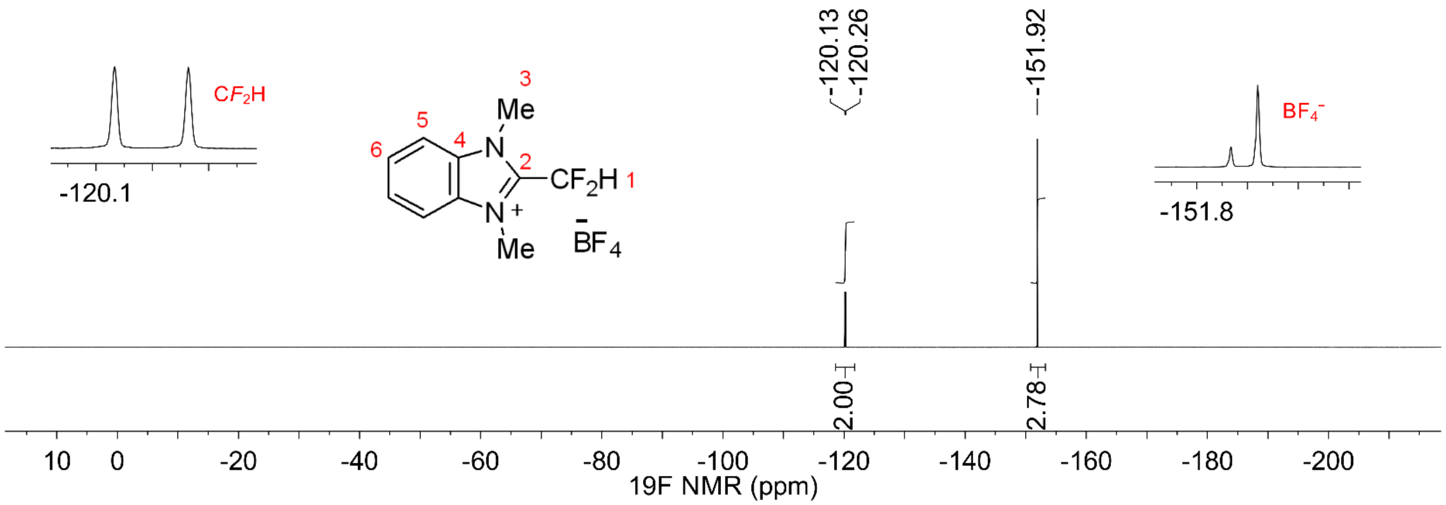

Figure S71. ^{19}F NMR spectrum of 2-(difluoromethyl)-1,3-dimethyl-benzoimidazolium tetrafluoroborate (**3b**).

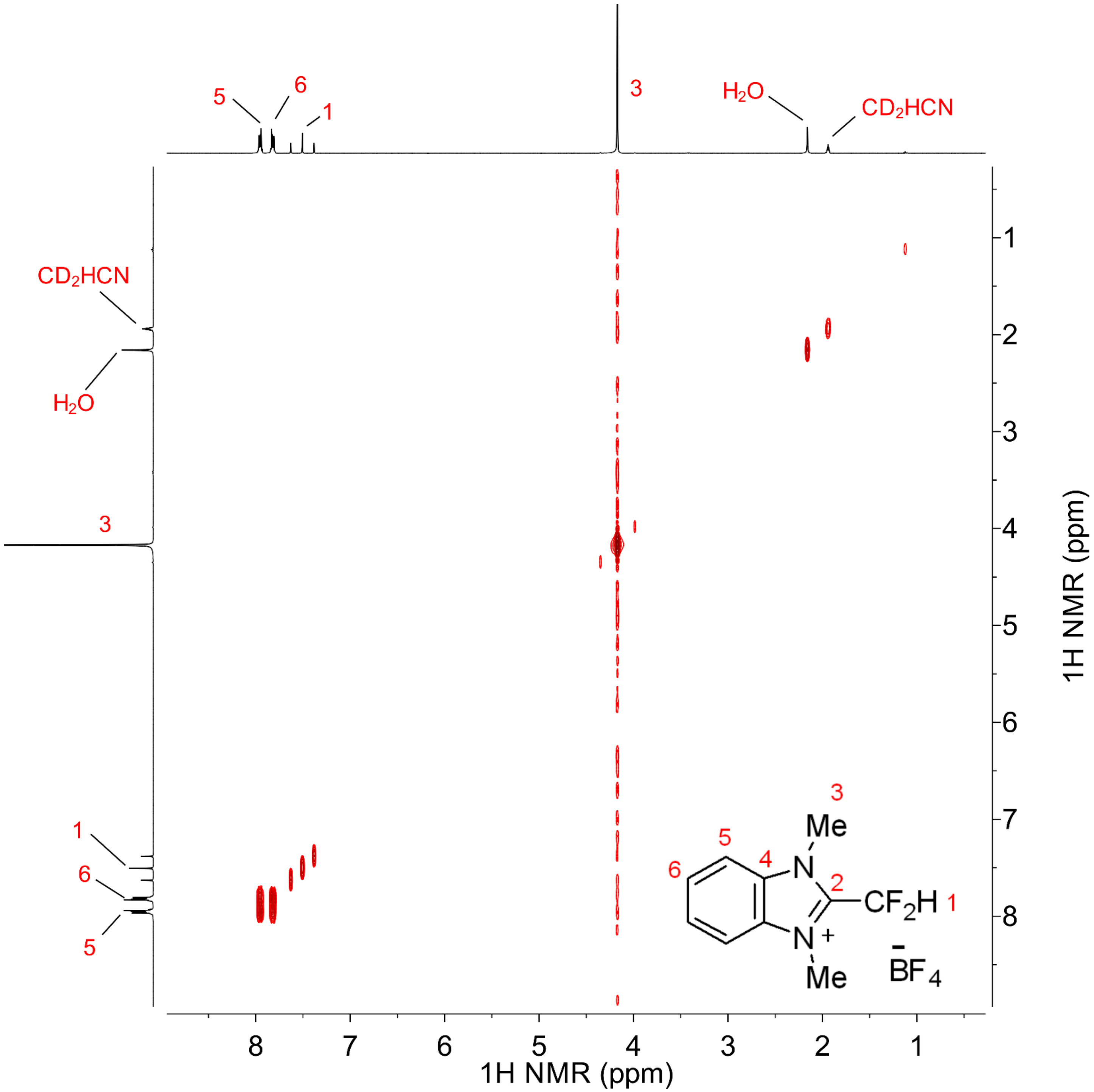

Figure S72. ^{1}H-^{1}H COSY spectrum of 2-(difluoromethyl)-1,3-dimethyl-benzoimidazolium tetrafluoroborate (**3b**).

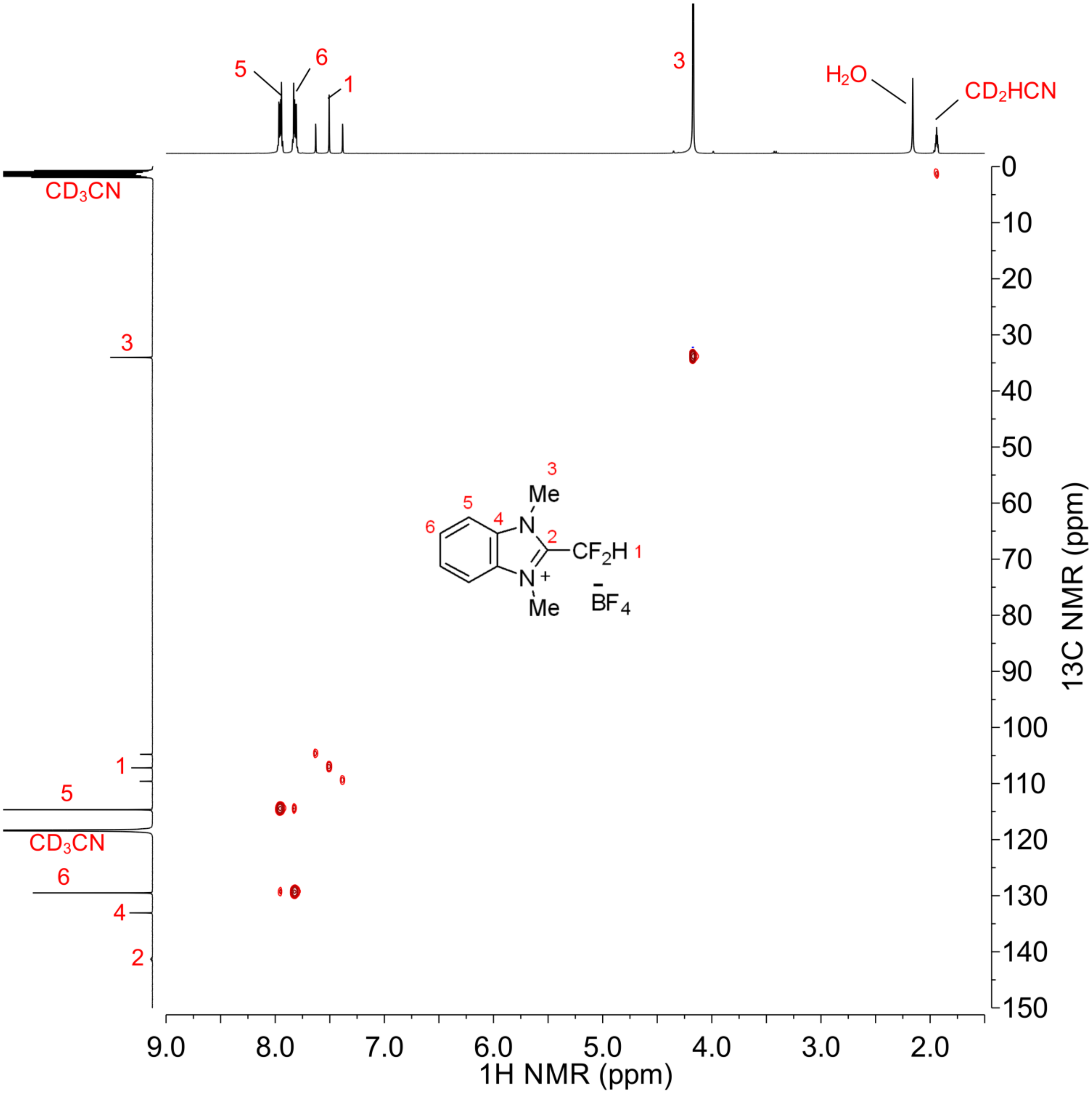

Figure S73. ^{1}H-^{13}C HSQC spectrum of 2-(difluoromethyl)-1,3-dimethyl-benzoimidazolium tetrafluoroborate (**3b**).

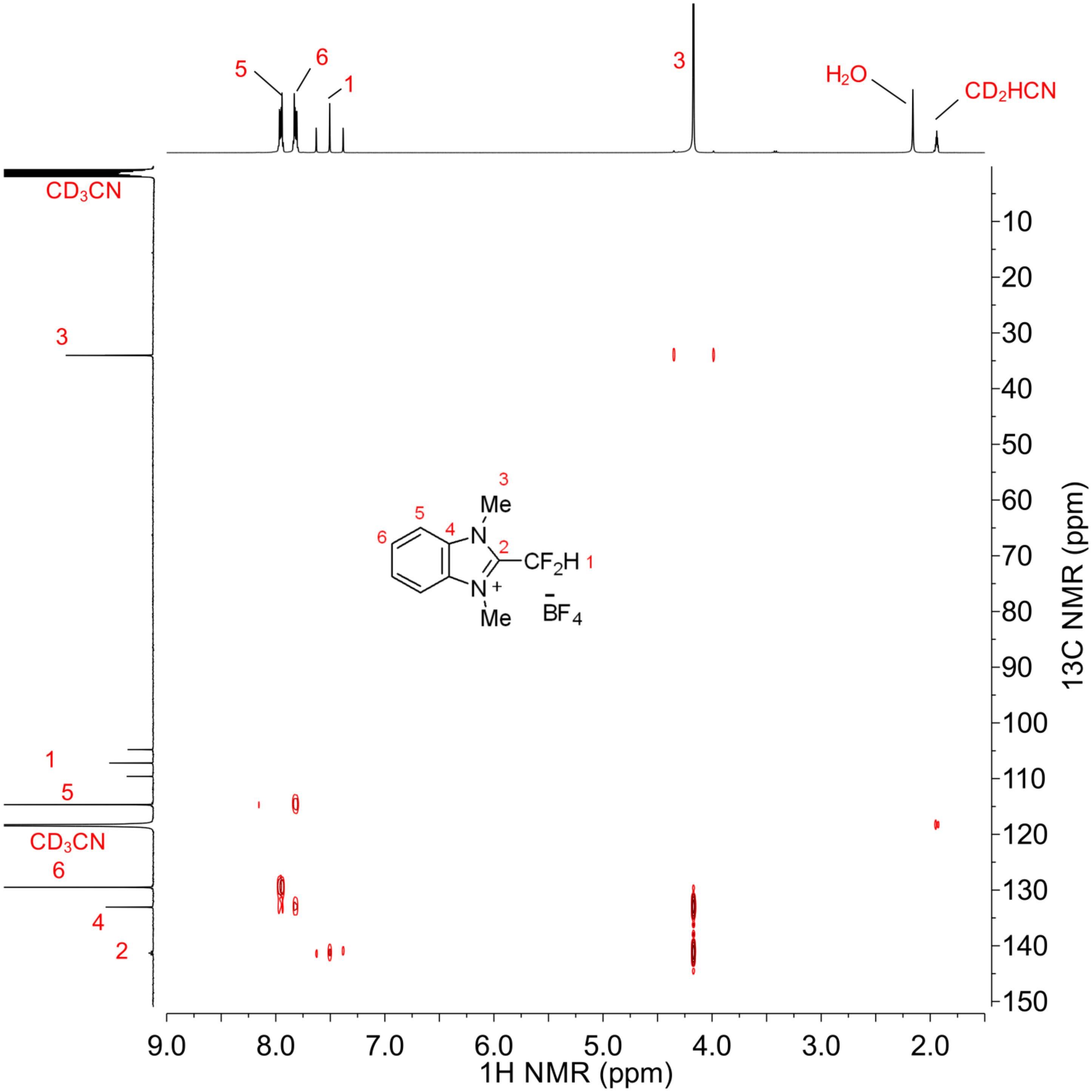

Figure S74. ^{1}H-^{13}C HMBC spectrum of 2-(difluoromethyl)-1,3-dimethyl-benzoimidazolium tetrafluoroborate (**3b**).

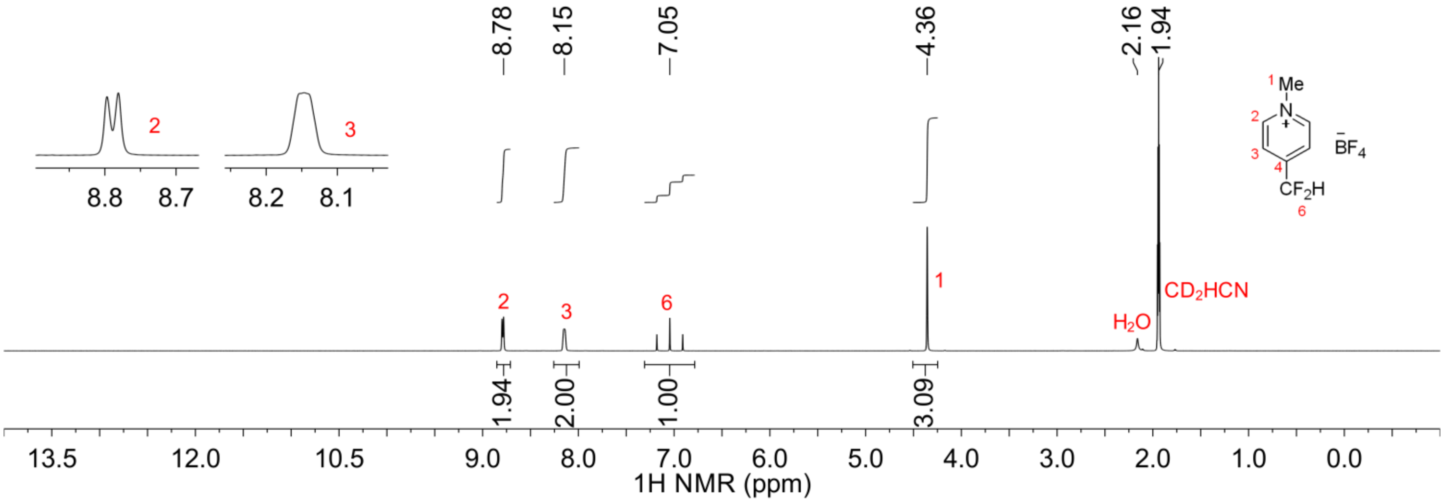

Figure S75. ^{1}H NMR spectrum of 4-(difluoromethyl)-*N*-methylpyridinium tetrafluoroborate (**4b**).

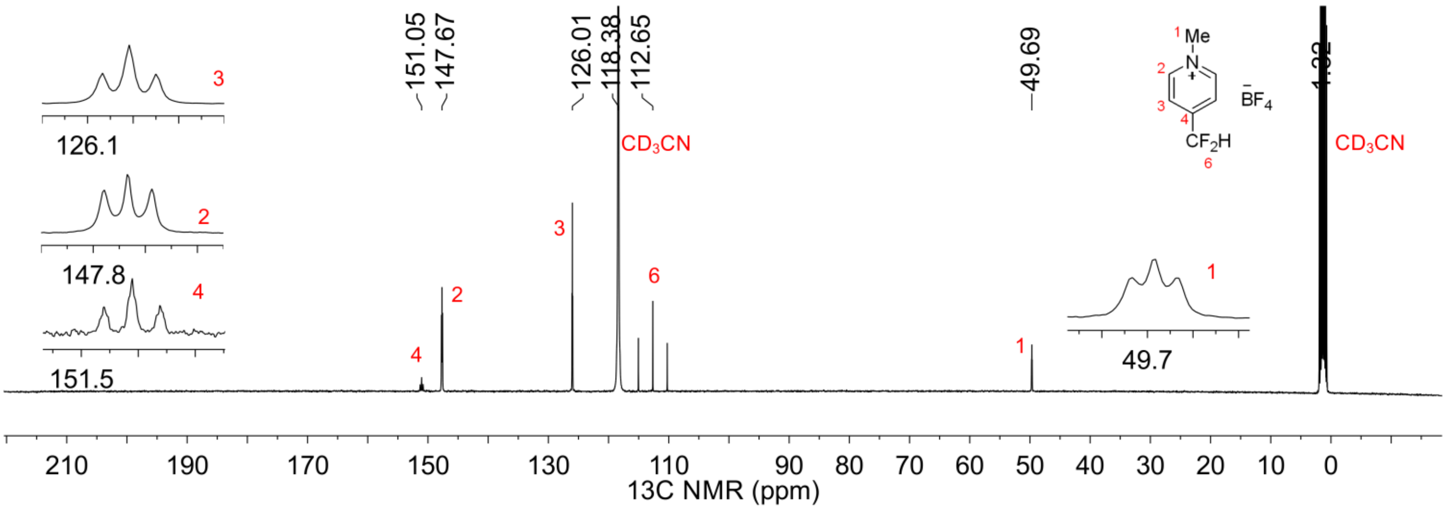

Figure S76. $^{13}C\{^{1}H\}$ NMR spectrum of 4-(difluoromethyl)-*N*-methylpyridinium tetrafluoroborate (**4b**).

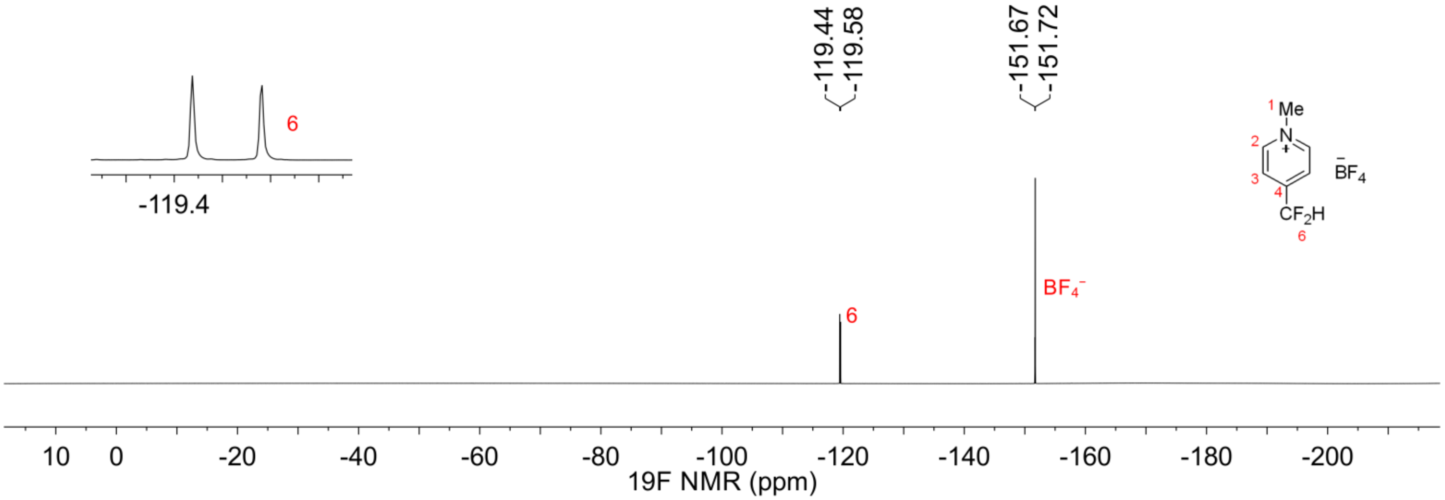

Figure S77. ^{19}F NMR spectrum of 4-(difluoromethyl)-*N*-methylpyridinium tetrafluoroborate (**4b**).

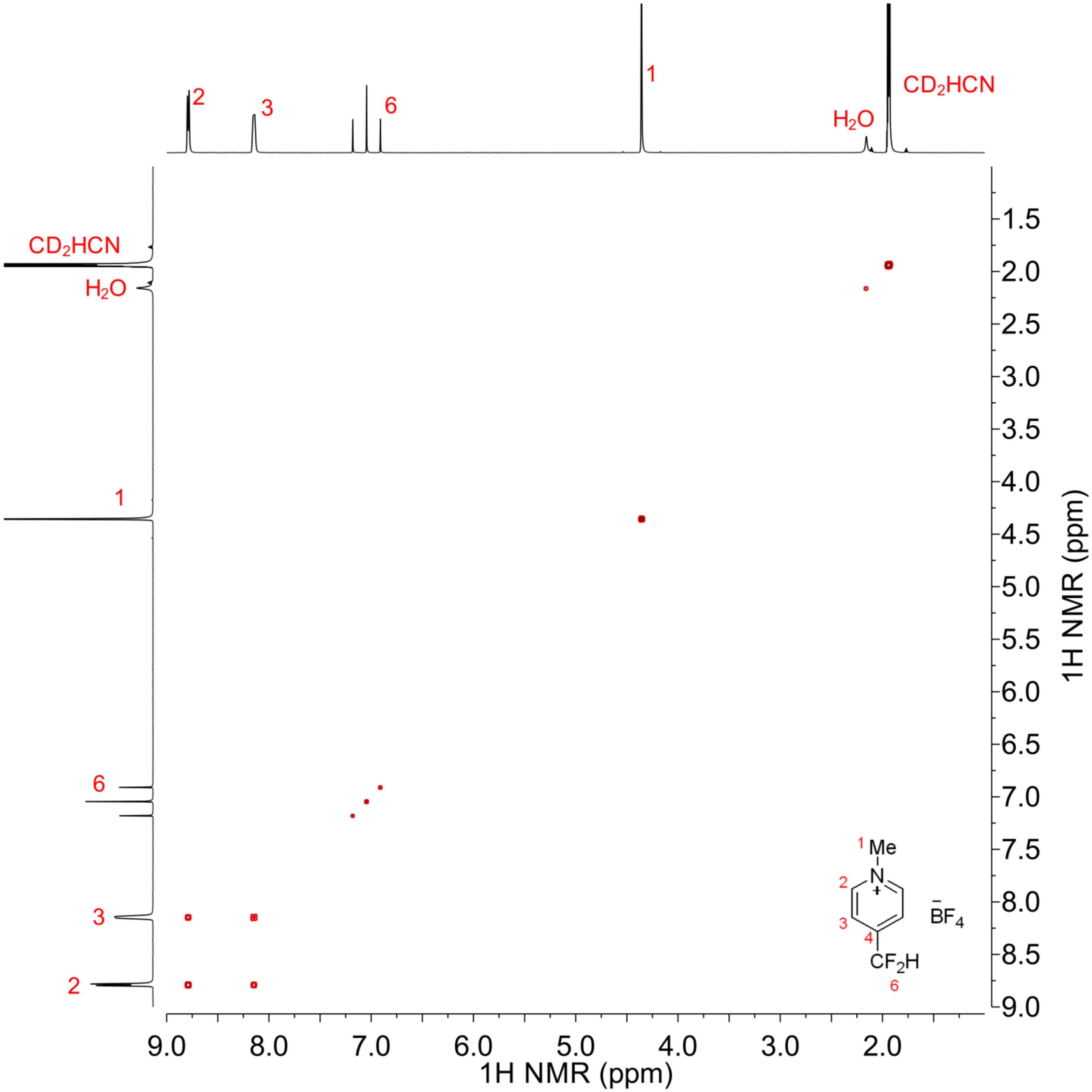

Figure S78. ^{1}H-^{1}H COSY spectrum of 4-(difluoromethyl)-*N*-methylpyridinium tetrafluoroborate (**4b**).

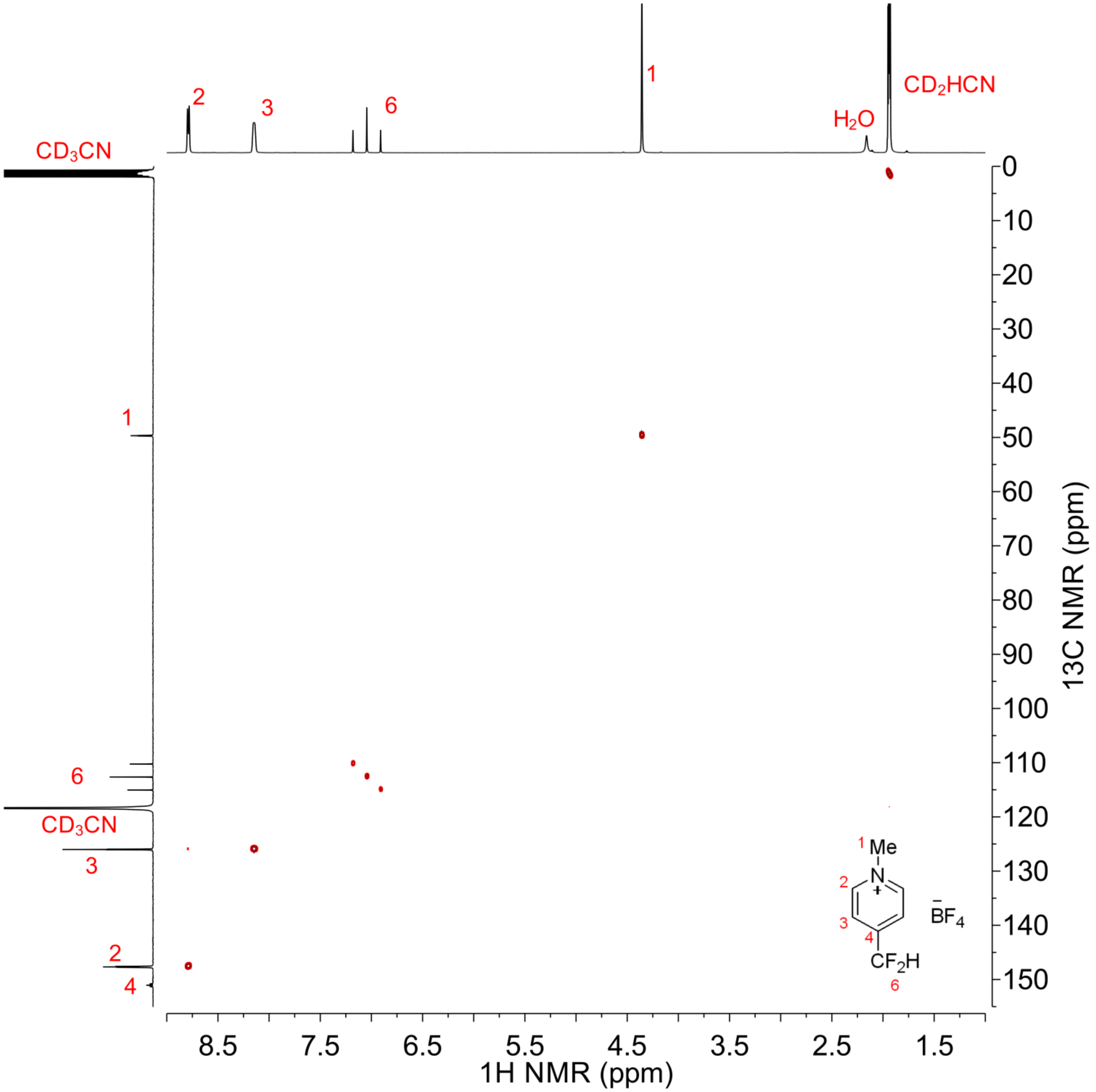

Figure S79. ^{1}H-^{13}C HSQC spectrum of 4-(difluoromethyl)-*N*-methylpyridinium tetrafluoroborate (**4b**).

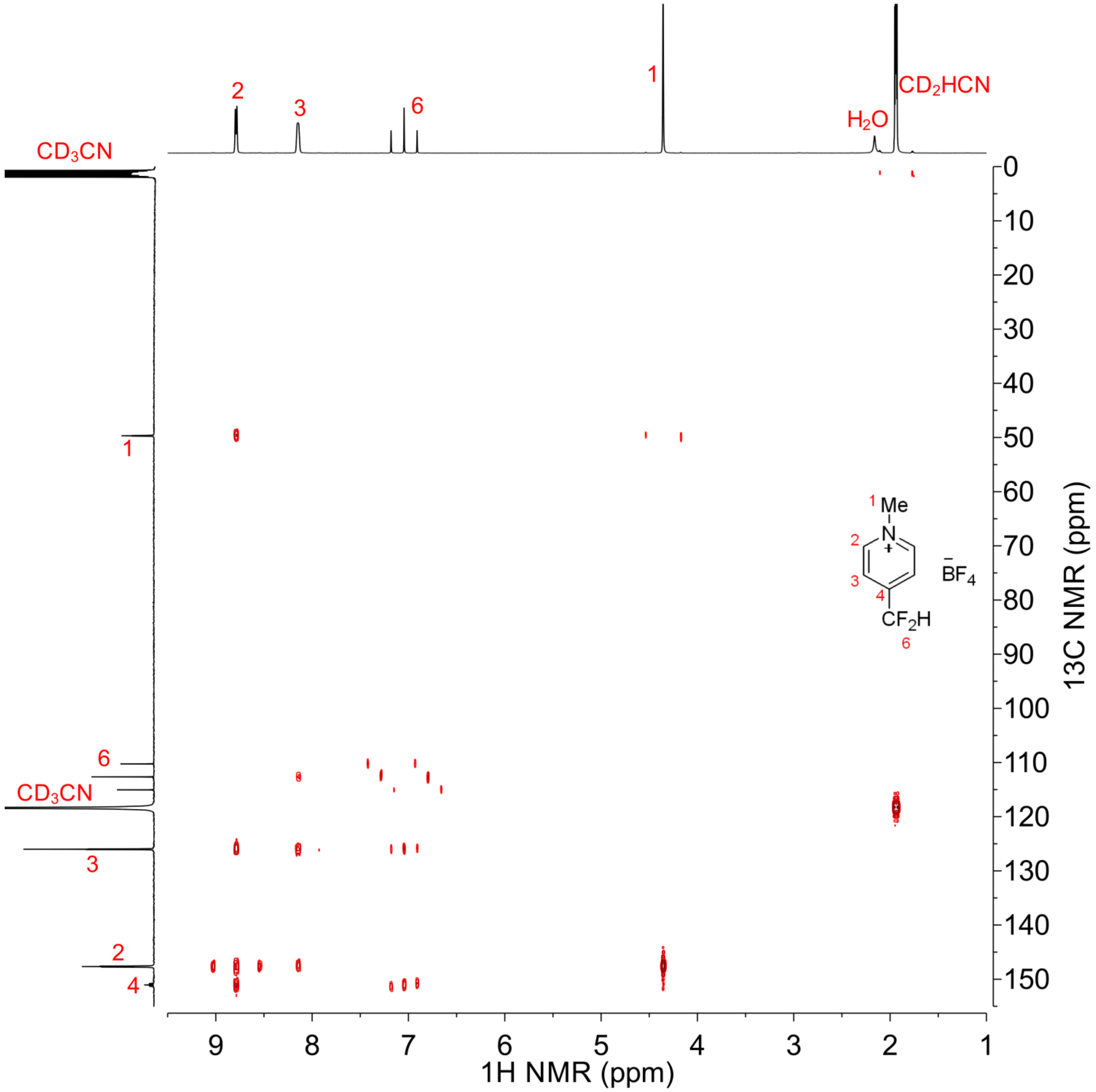

Figure S80. ^{1}H-^{13}C HMBC spectrum of 4-(difluoromethyl)-*N*-methylpyridinium tetrafluoroborate (**4b**).

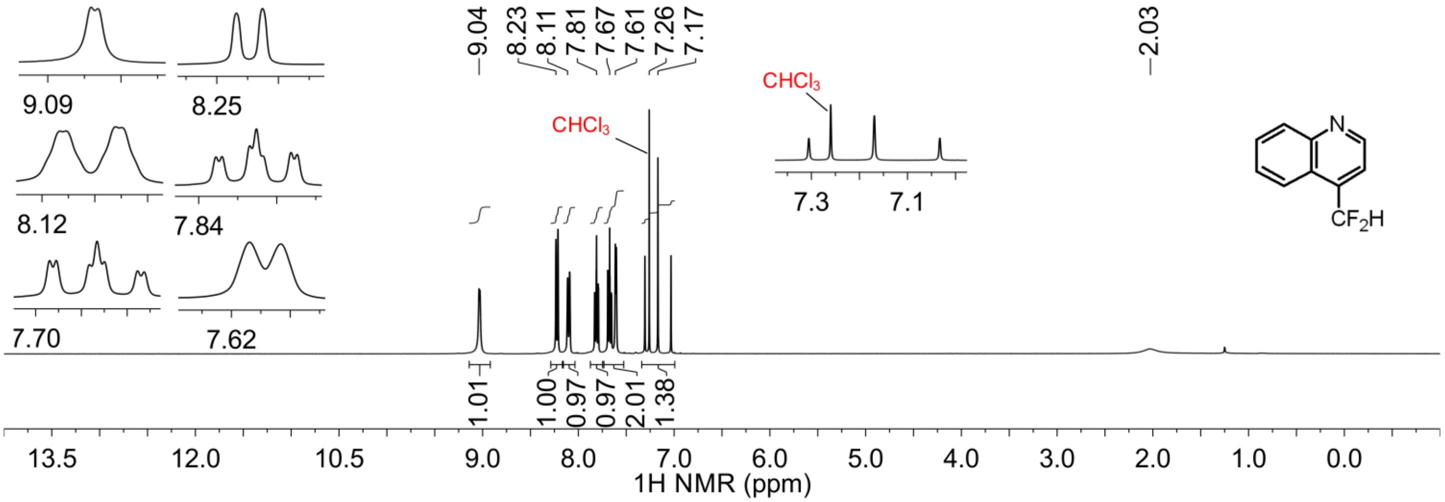

Figure S81. ^{1}H NMR spectrum of 4-(difluoromethyl)-*N*-methylquinolinium tetrafluorobor (**5a**).

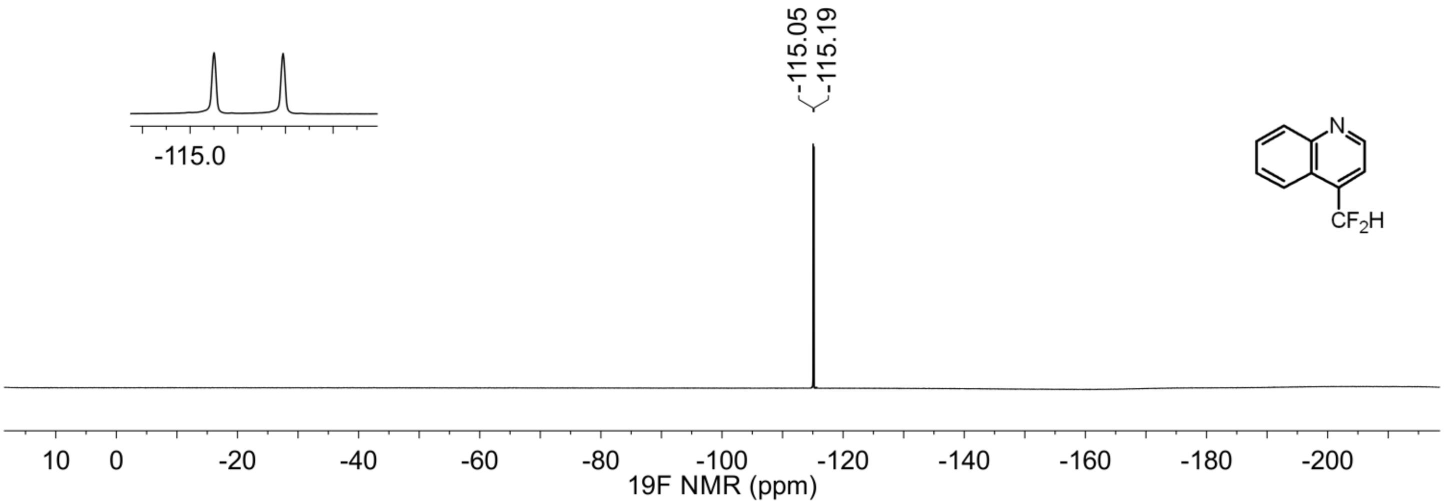

Figure S82. ^{19}F NMR spectrum of 4-(difluoromethyl)-*N*-methylquinolinium tetrafluorobor (**5a**).

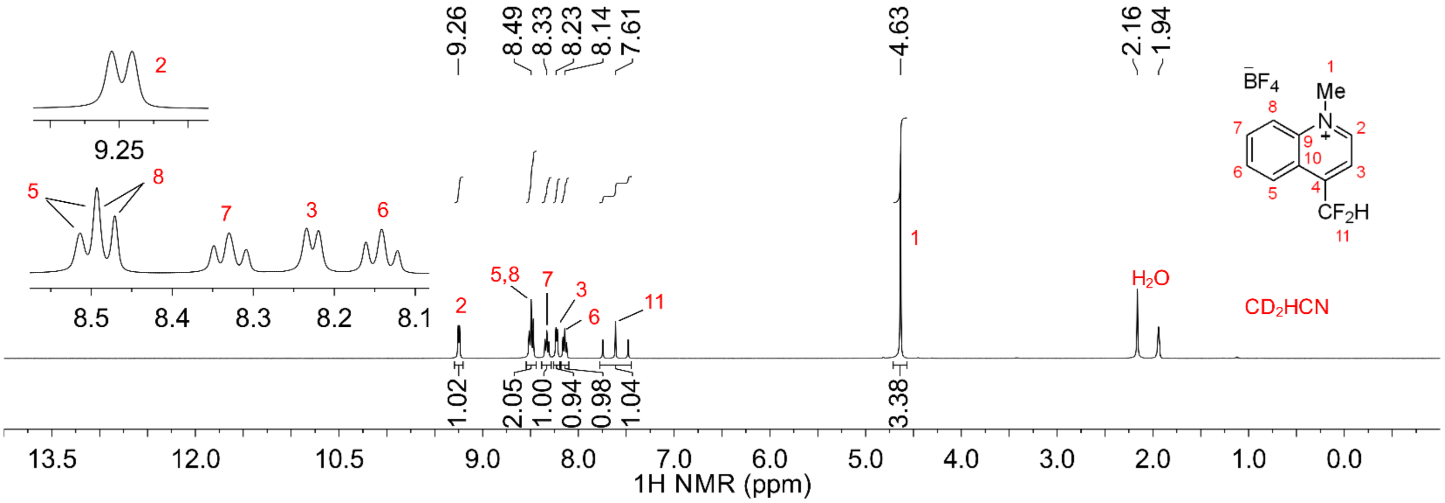

Figure S83. ^{1}H NMR spectrum of 4-(difluoromethyl)-*N*-methylquinolinium tetrafluoroborate (**5b**).

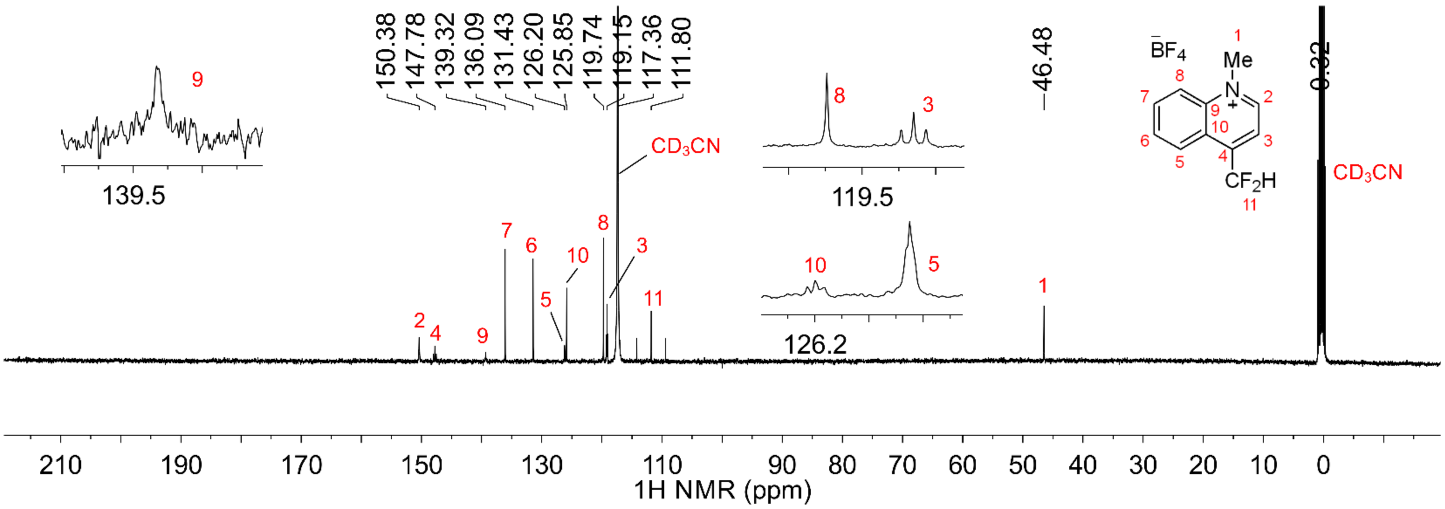

Figure S84. ^{13}C{^{1}H} NMR spectrum of 4-(difluoromethyl)-*N*-methylquinolinium tetrafluoroborate (**5b**).

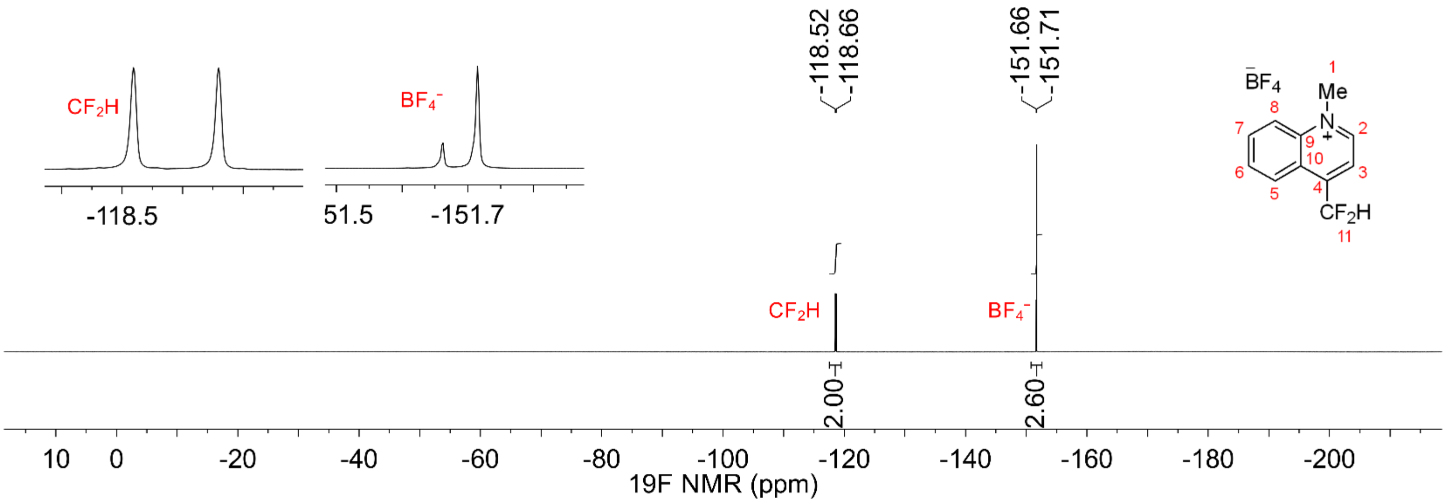

Figure S85. ^{19}F NMR spectrum of 4-(difluoromethyl)-*N*-methylquinolinium tetrafluoroborate (**5b**).

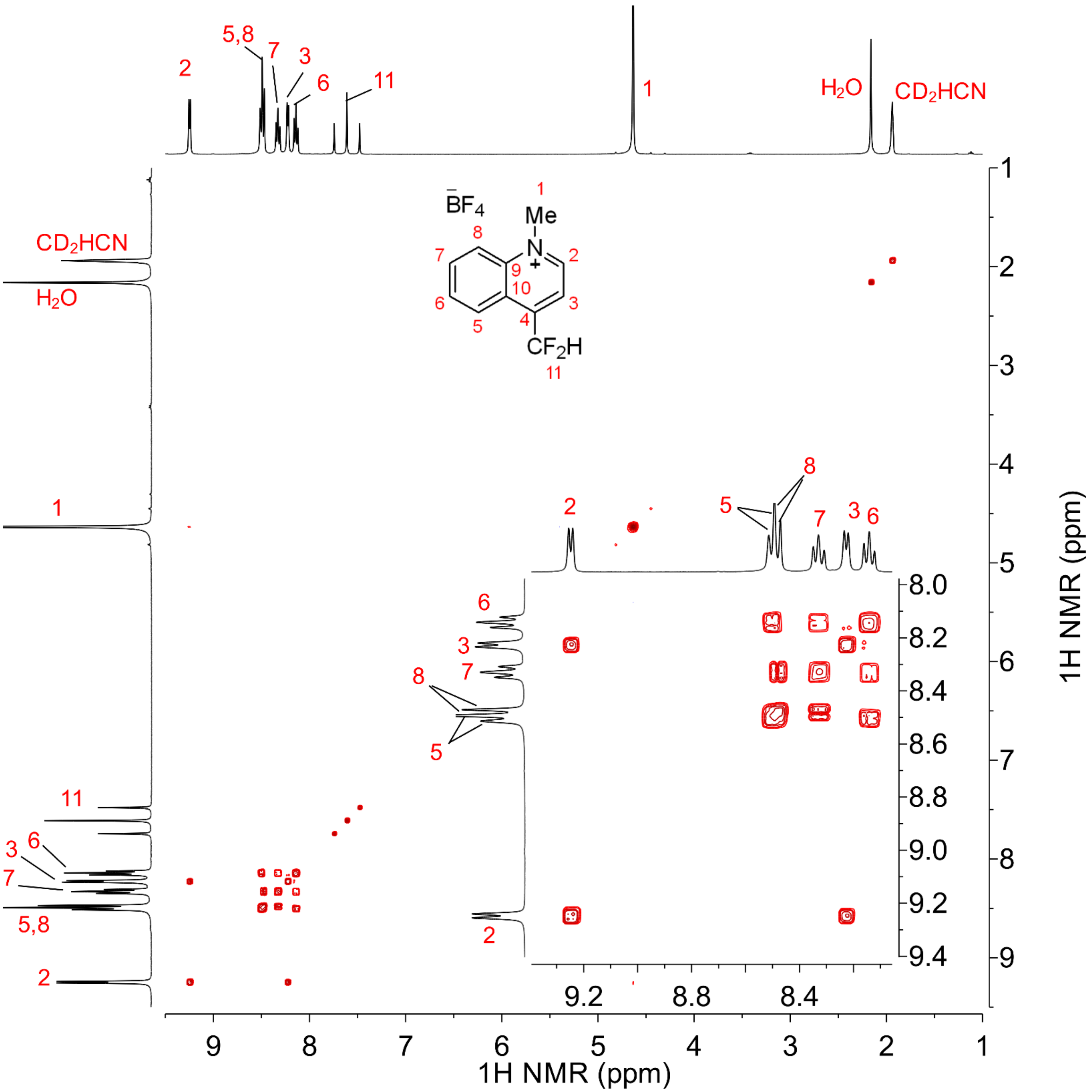

Figure S86. ^{1}H-^{1}H COSY spectrum of 4-(difluoromethyl)-*N*-methylquinolinium tetrafluoroborate (**5b**).

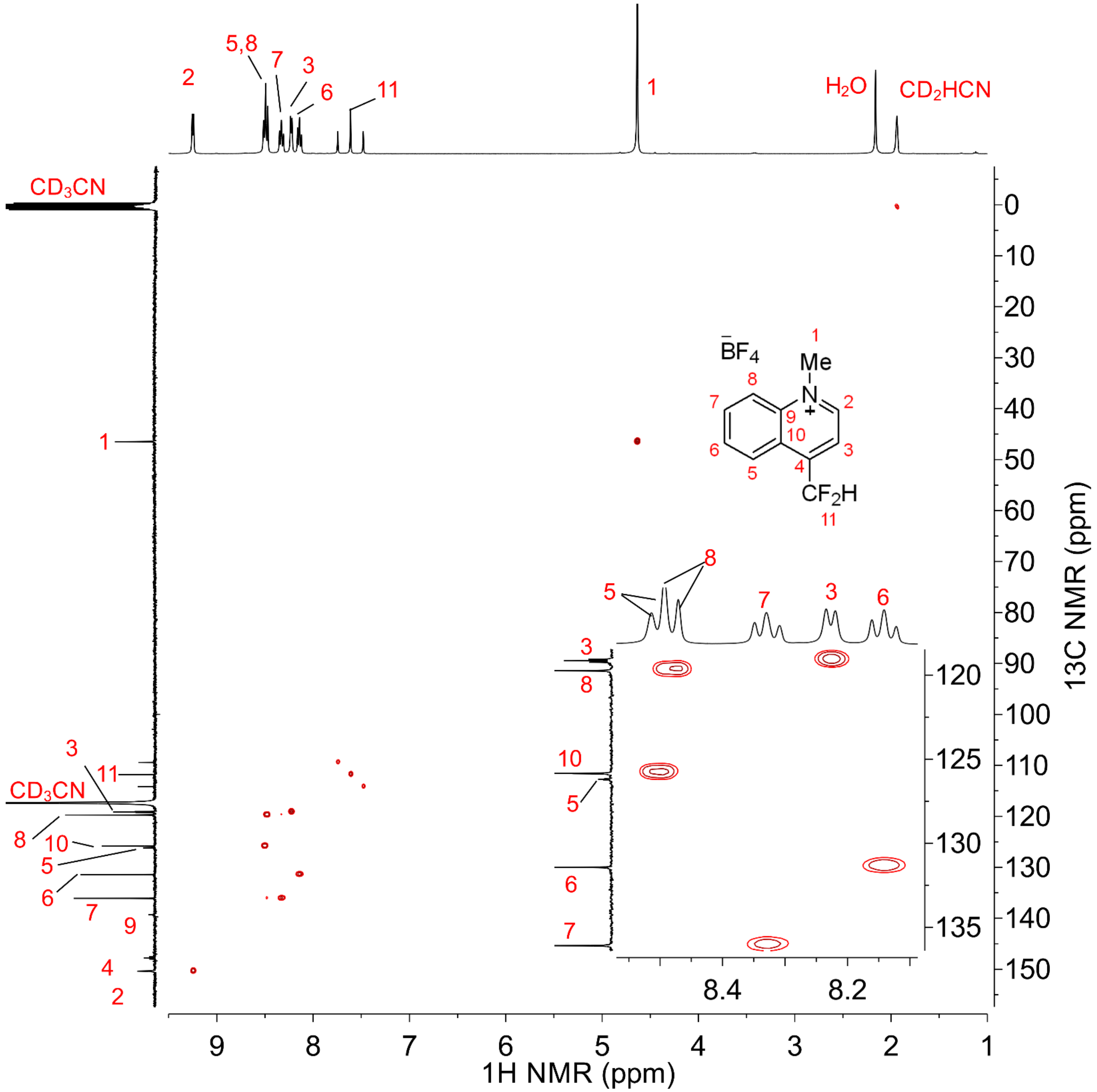

Figure S87. ^1H-^{13}C HSQC spectrum of 4-(difluoromethyl)-*N*-methylquinolinium tetrafluoroborate (**5b**).

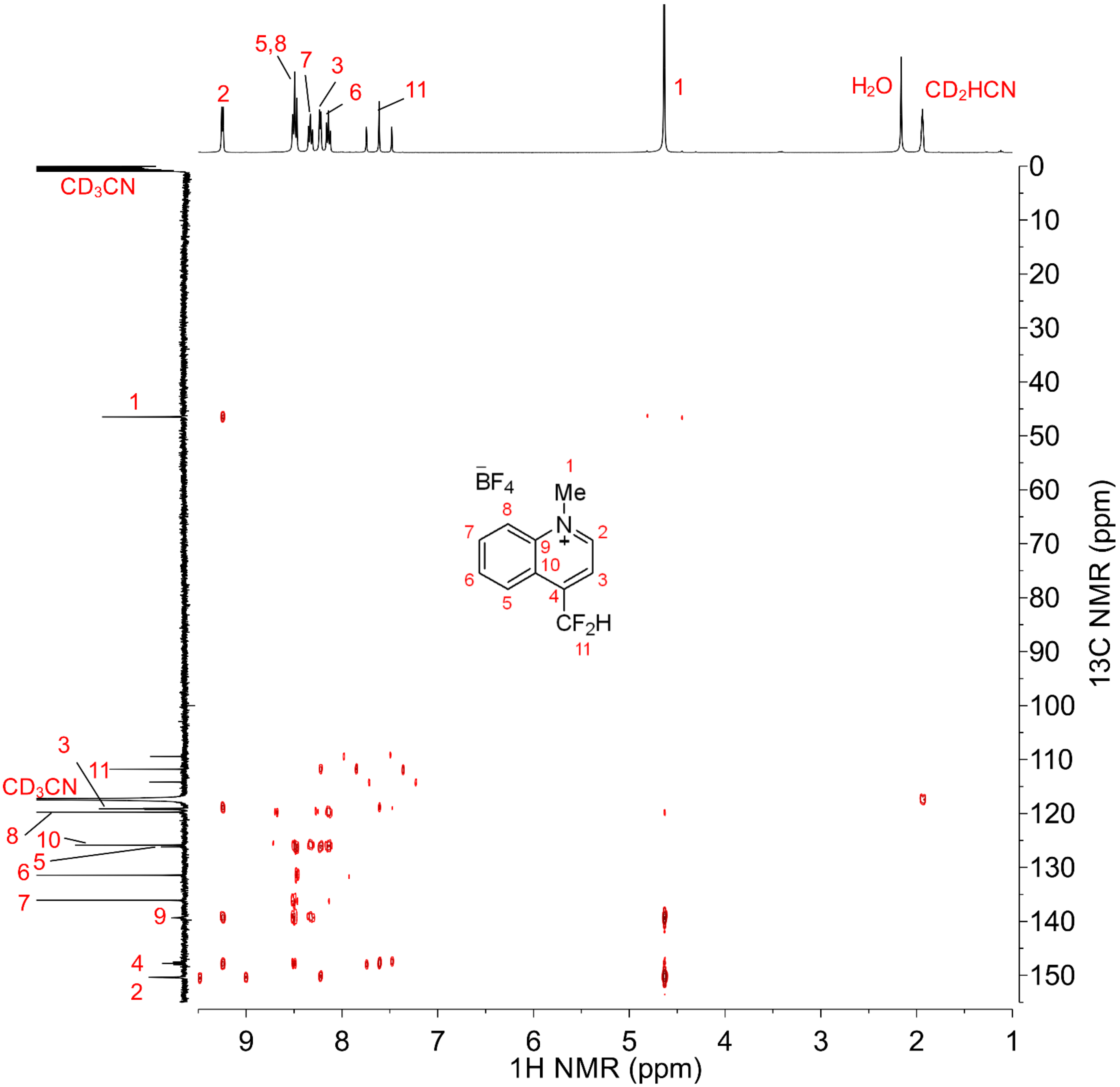

Figure S88. ^{1}H-^{13}C HMBC spectrum of 4-(difluoromethyl)-*N*-methylquinolinium tetrafluoroborate (**5b**).

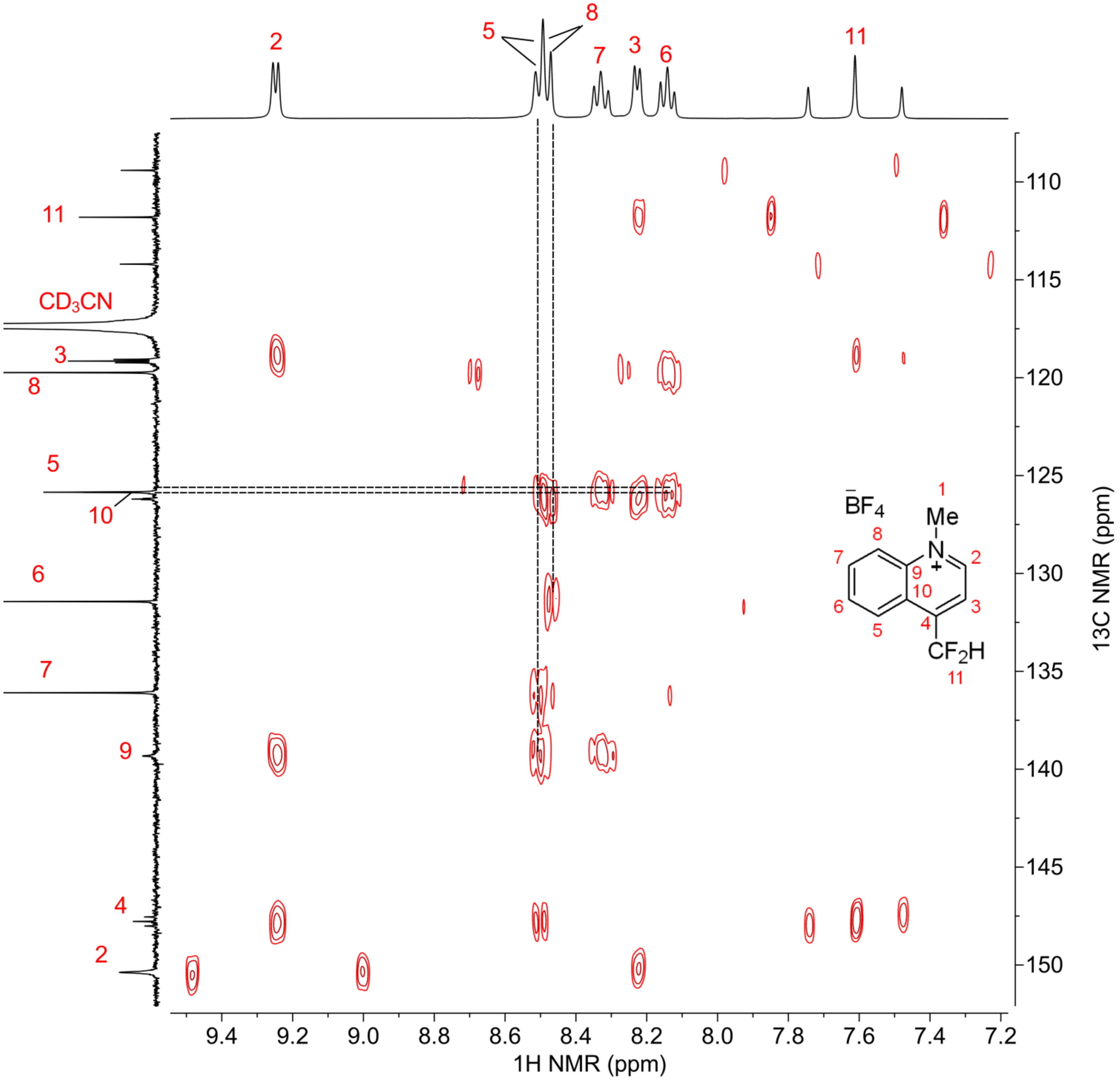

Figure S89. Expansion of ^{1}H-^{13}C HMBC spectrum of 4-(difluoromethyl)-*N*-methylquinolinium tetrafluoroborate (**5b**) from 7.8 to 8.7 ppm (^{1}H) and 128 to 144 ppm (^{13}C).

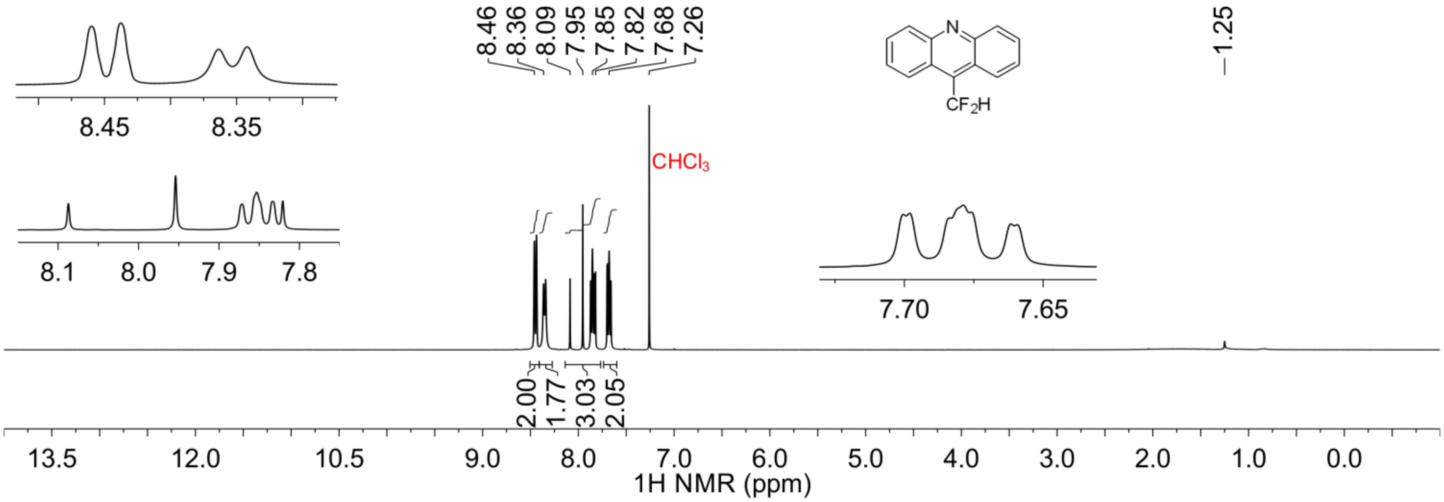

Figure S90. ^{1}H NMR spectrum of 9-(difluoromethyl)acridine (**6a**).

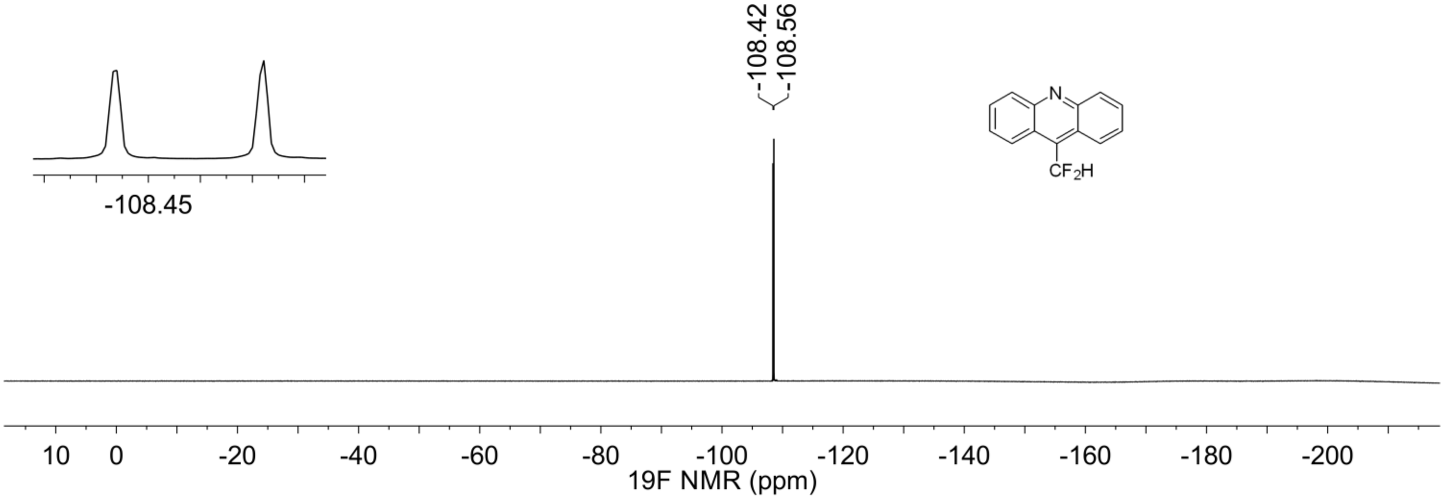

Figure S91. ^{19}F NMR spectrum of 9-(difluoromethyl)acridine (**6a**).

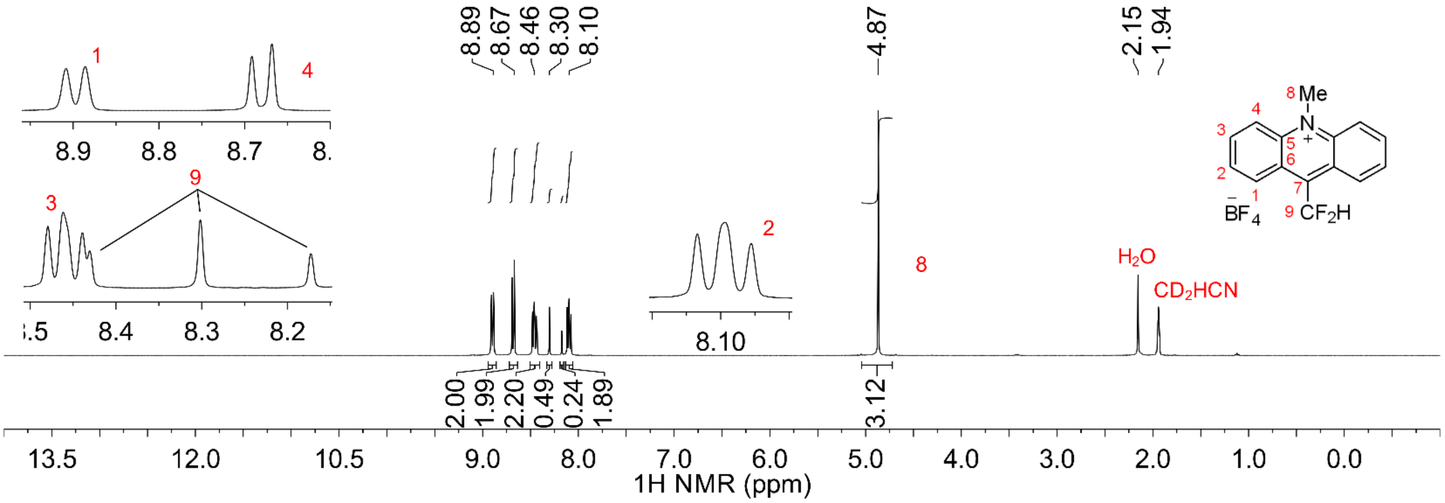

Figure S92. ^{1}H NMR spectrum of *N*-methyl-9-(difluoromethyl)acridinium tetrafluoroborate (**6b**).

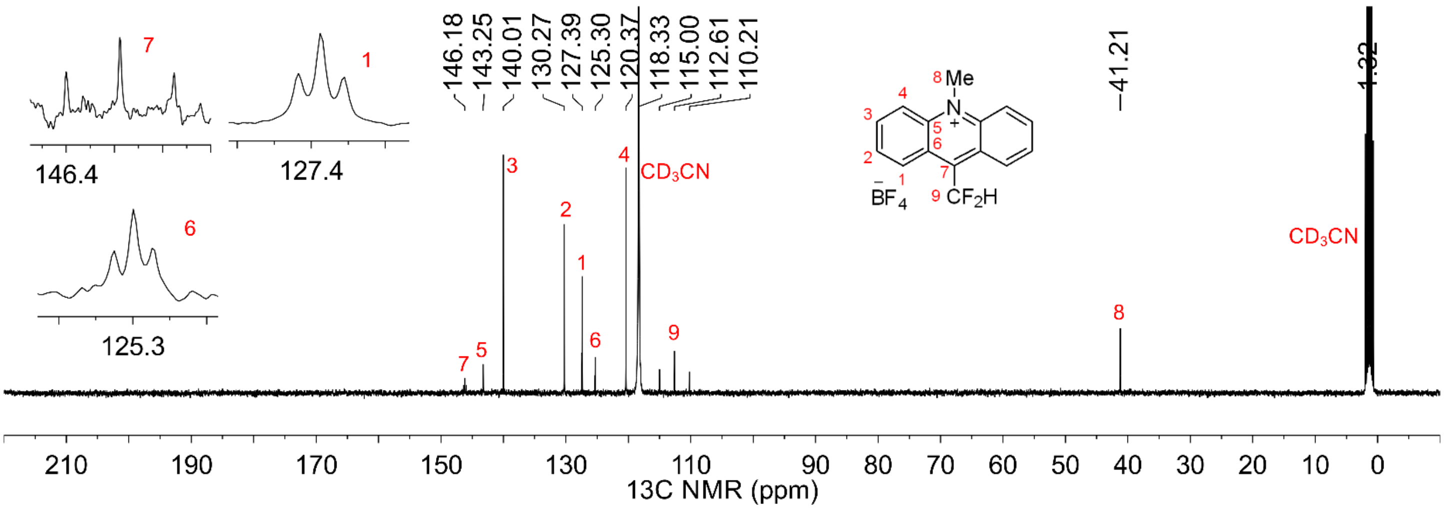

Figure S93. $^{13}C\{^{1}H\}$ NMR spectrum of *N*-methyl-9-(difluoromethyl)acridinium tetrafluoroborate (**6b**).

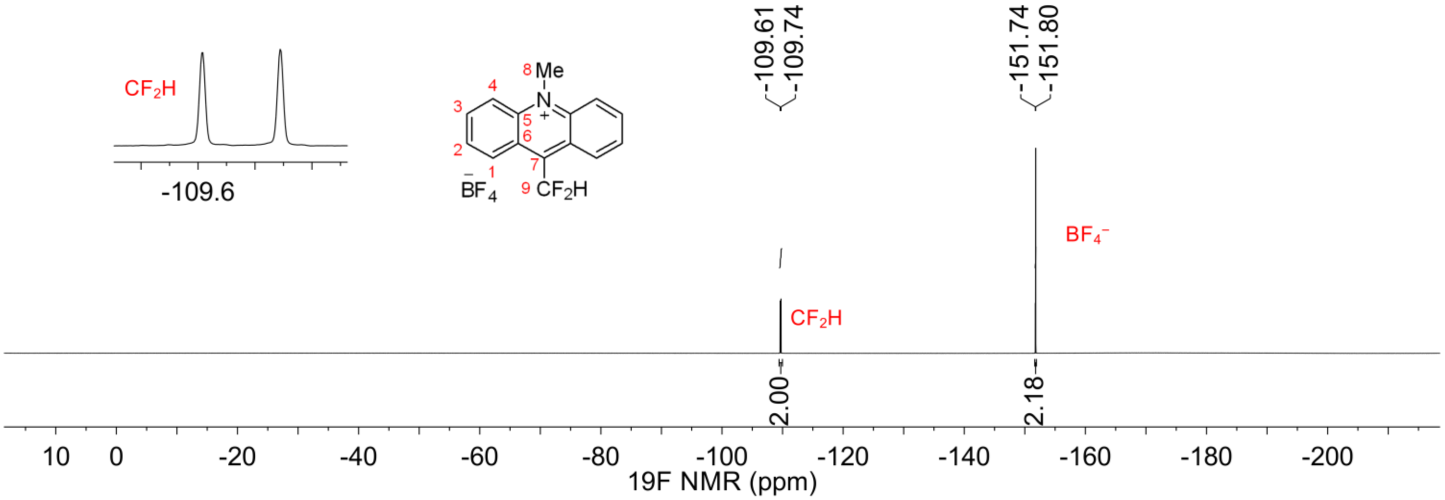

Figure S94. ^{19}F NMR spectrum of *N*-methyl-9-(difluoromethyl)acridinium tetrafluoroborate (**6b**).

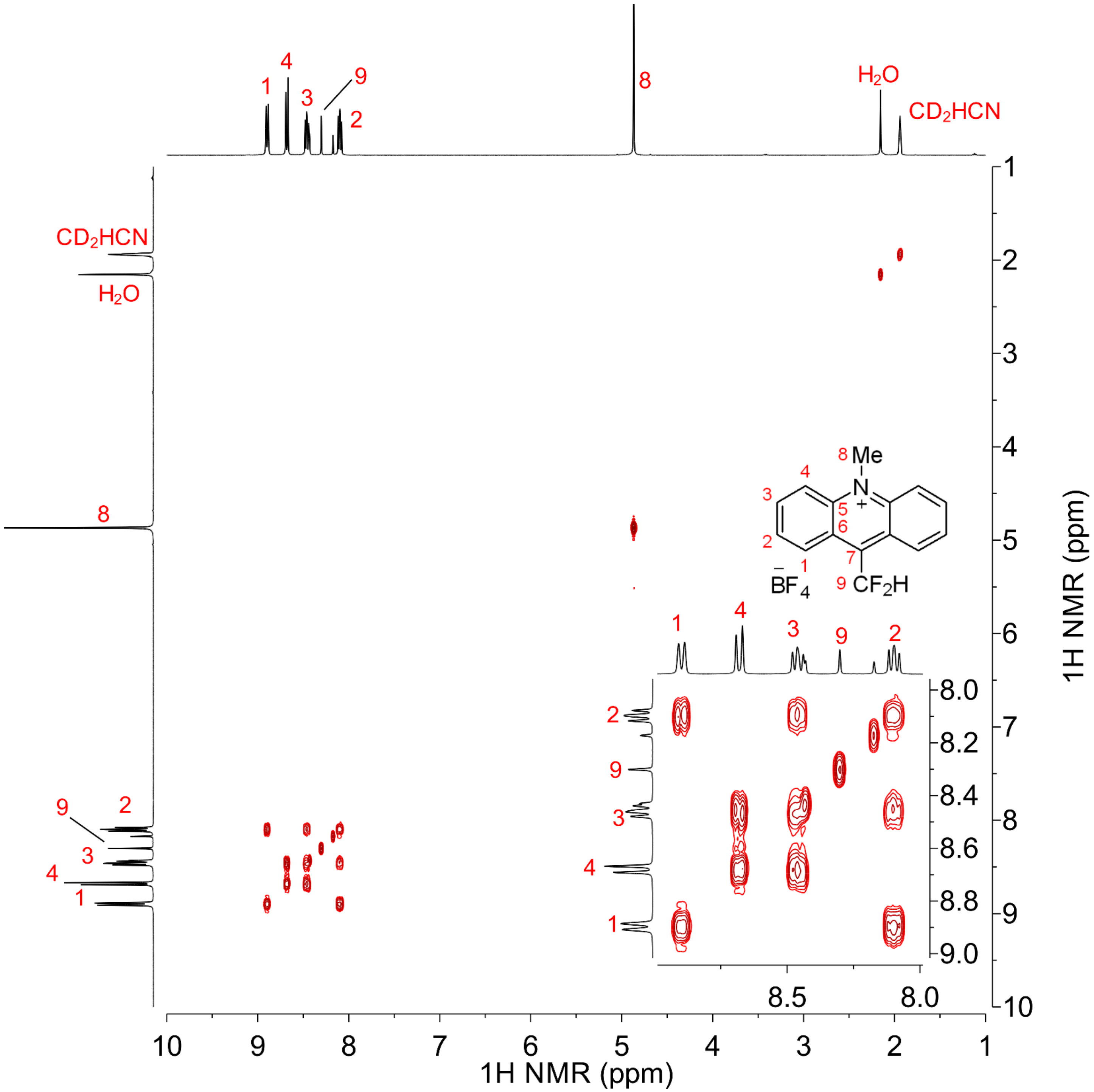

Figure S95. ^1H-^1H COSY spectrum of *N*-methyl-9-(difluoromethyl)acridinium tetrafluoroborate (**6b**).

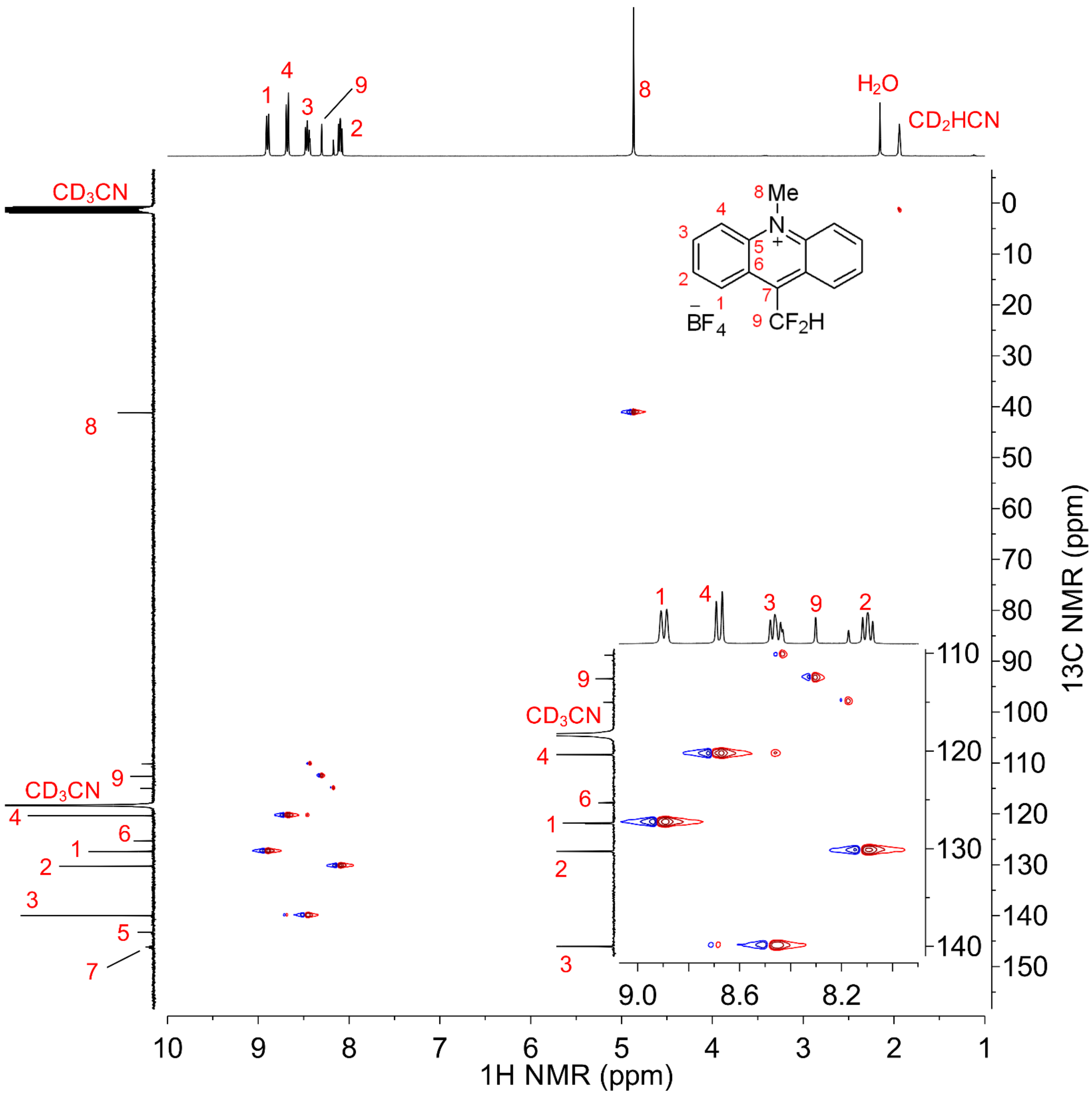

Figure S96. ^{1}H-^{13}C HSQC spectrum of *N*-methyl-9-(difluoromethyl)acridinium tetrafluoroborate (**6b**).

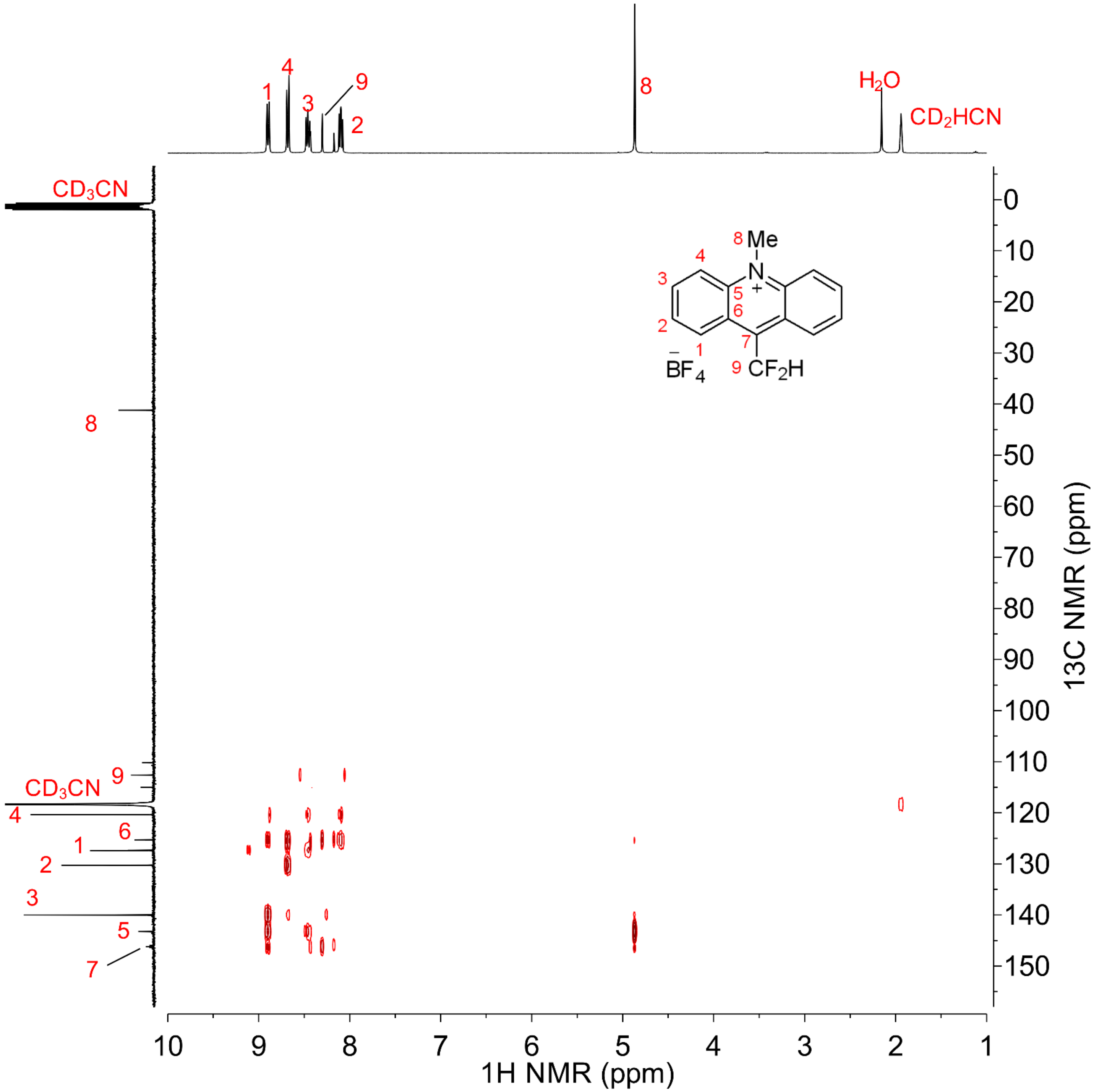

Figure S97. ^{1}H-^{13}C HMBC spectrum of *N*-methyl-9-(difluoromethyl)acridinium tetrafluoroborate (**6b**).

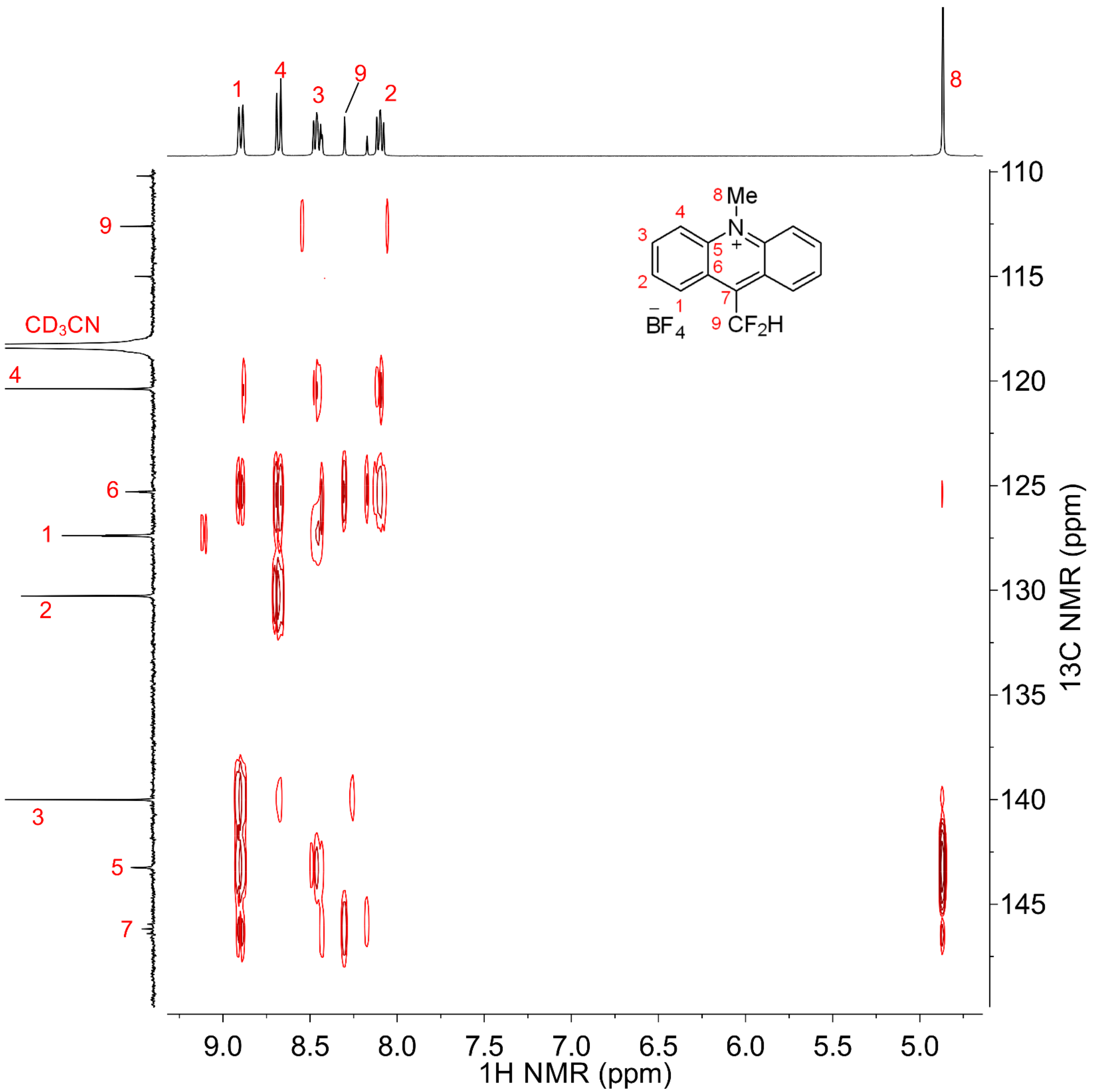

Figure S98. Expansion of ^{1}H-^{13}C HMBC spectrum of *N*-methyl-9-(difluoromethyl)acridinium tetrafluoroborate (**6b**) from 5.2 to 9.3 ppm (^{1}H) and 110 to 155 ppm (^{13}C).

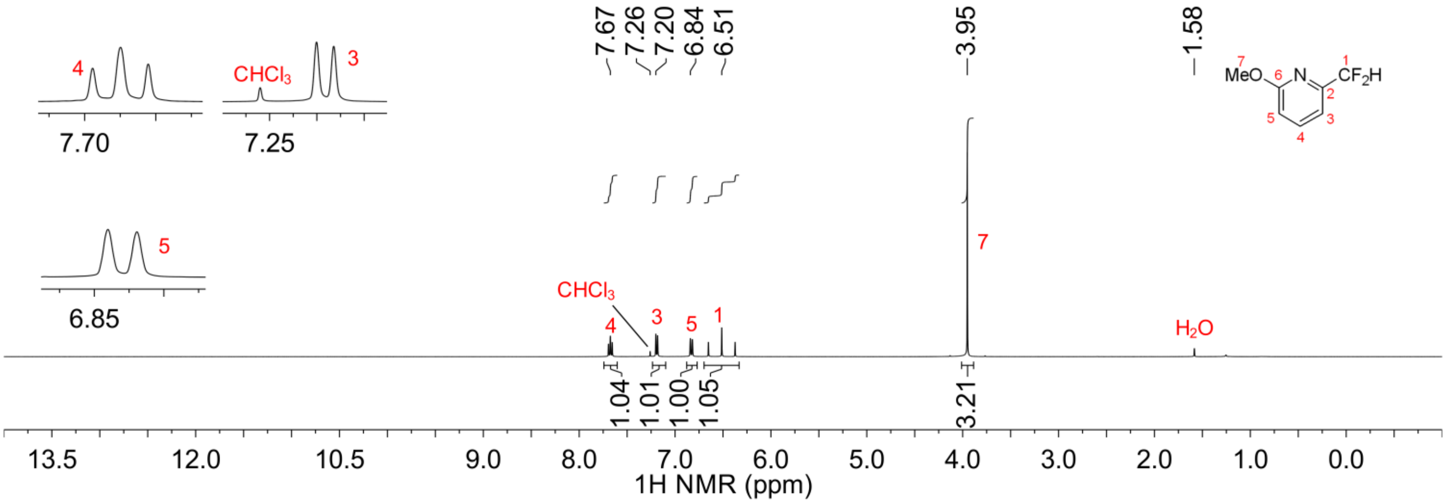

Figure S99. ^{1}H NMR spectrum of 2-(difluoromethyl)-6-methoxy-pyridine (**7a**).

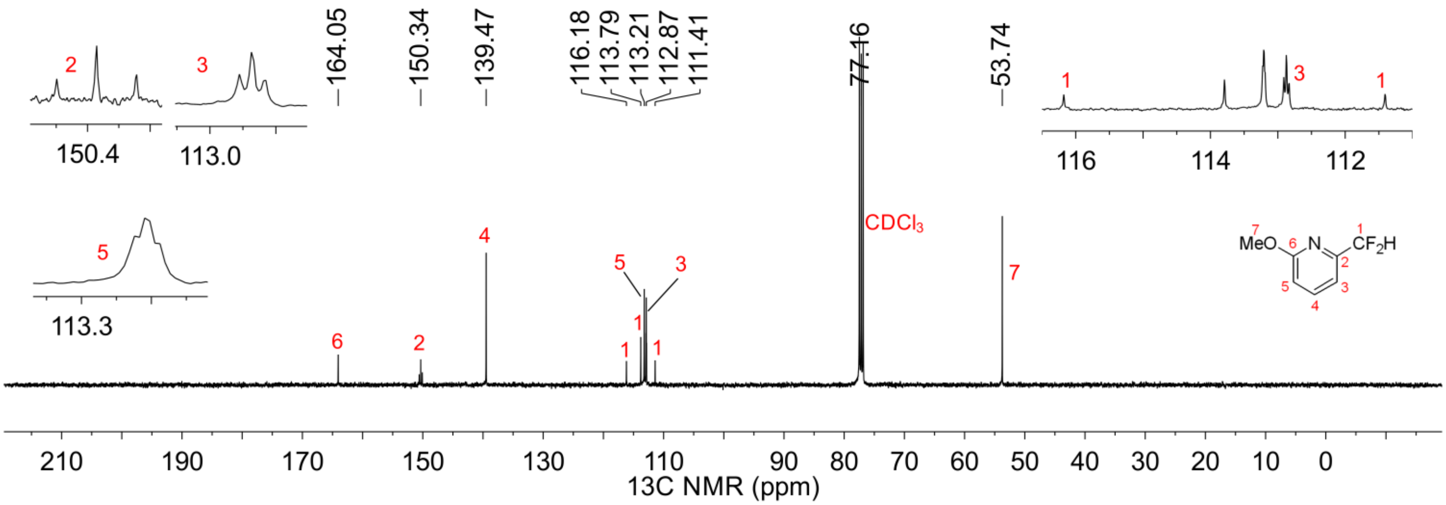

Figure S100. ^{13}C{^{1}H} NMR spectrum of 2-(difluoromethyl)-6-methoxy-pyridine (**7a**).

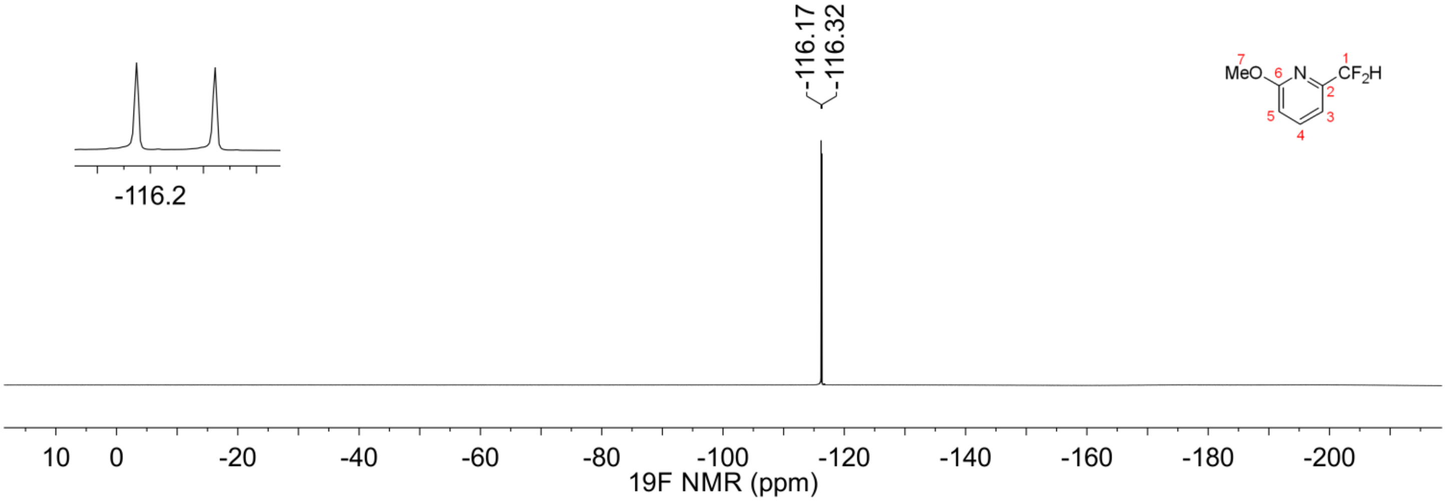

Figure S101. ^{19}F NMR spectrum of 2-(difluoromethyl)-6-methoxy-pyridine (**7a**).

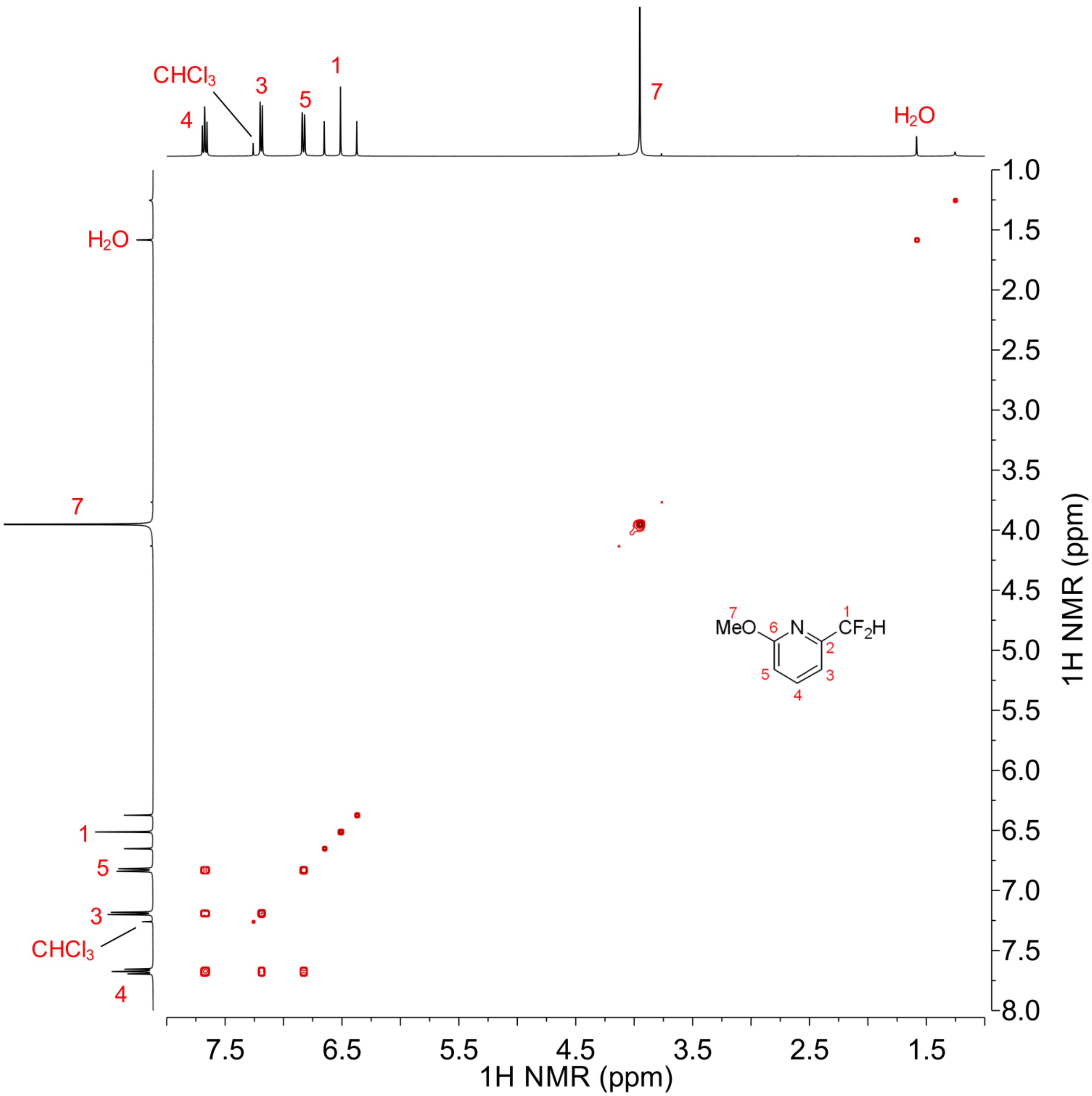

Figure S102. ^{1}H-^{1}H COSY spectrum of 2-(difluoromethyl)-6-methoxy-pyridine (**7a**).

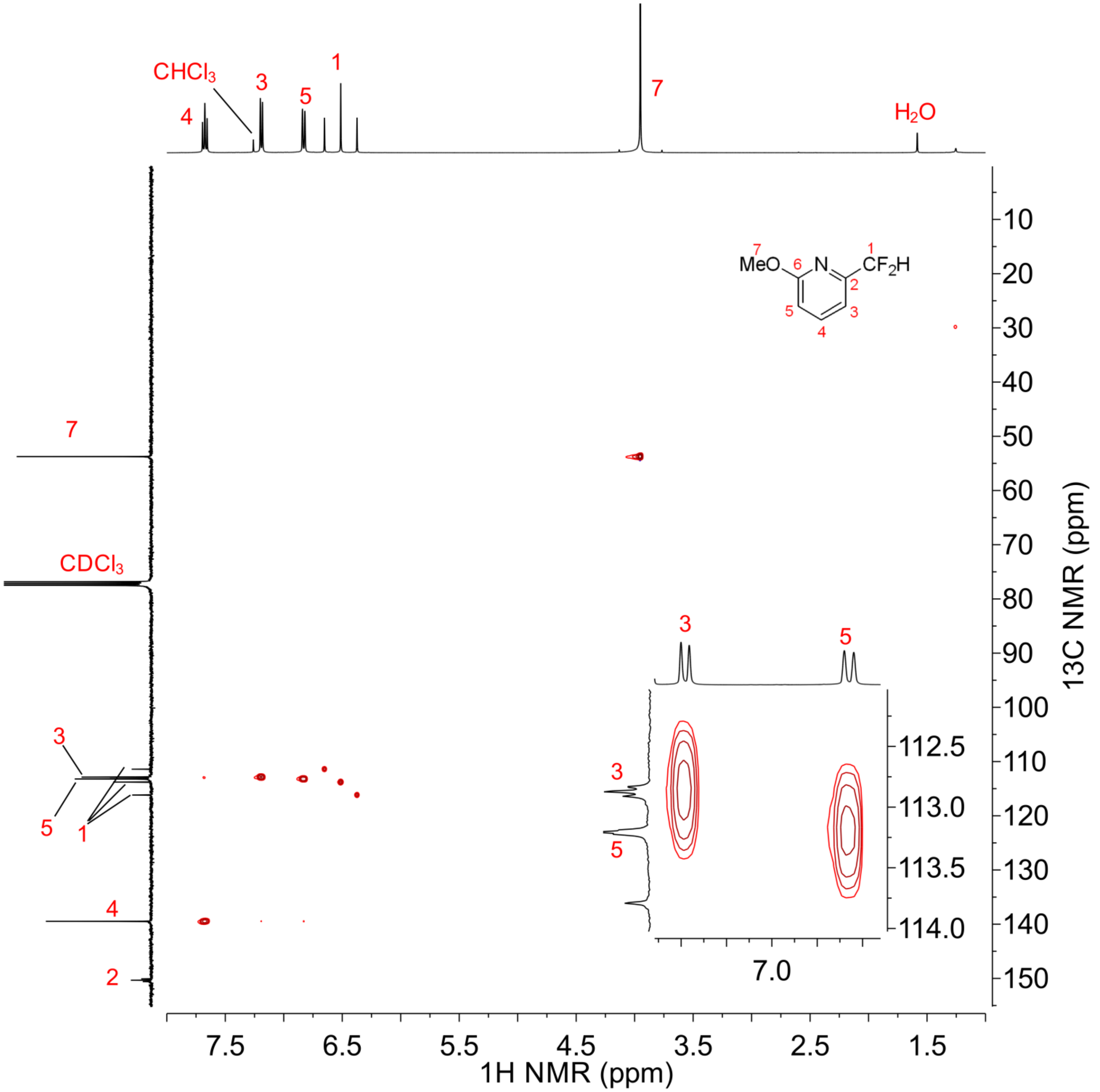

Figure S103. ${}^{1}H$-${}^{13}C$ HSQC spectrum of 2-(difluoromethyl)-6-methoxy-pyridine (**7a**).

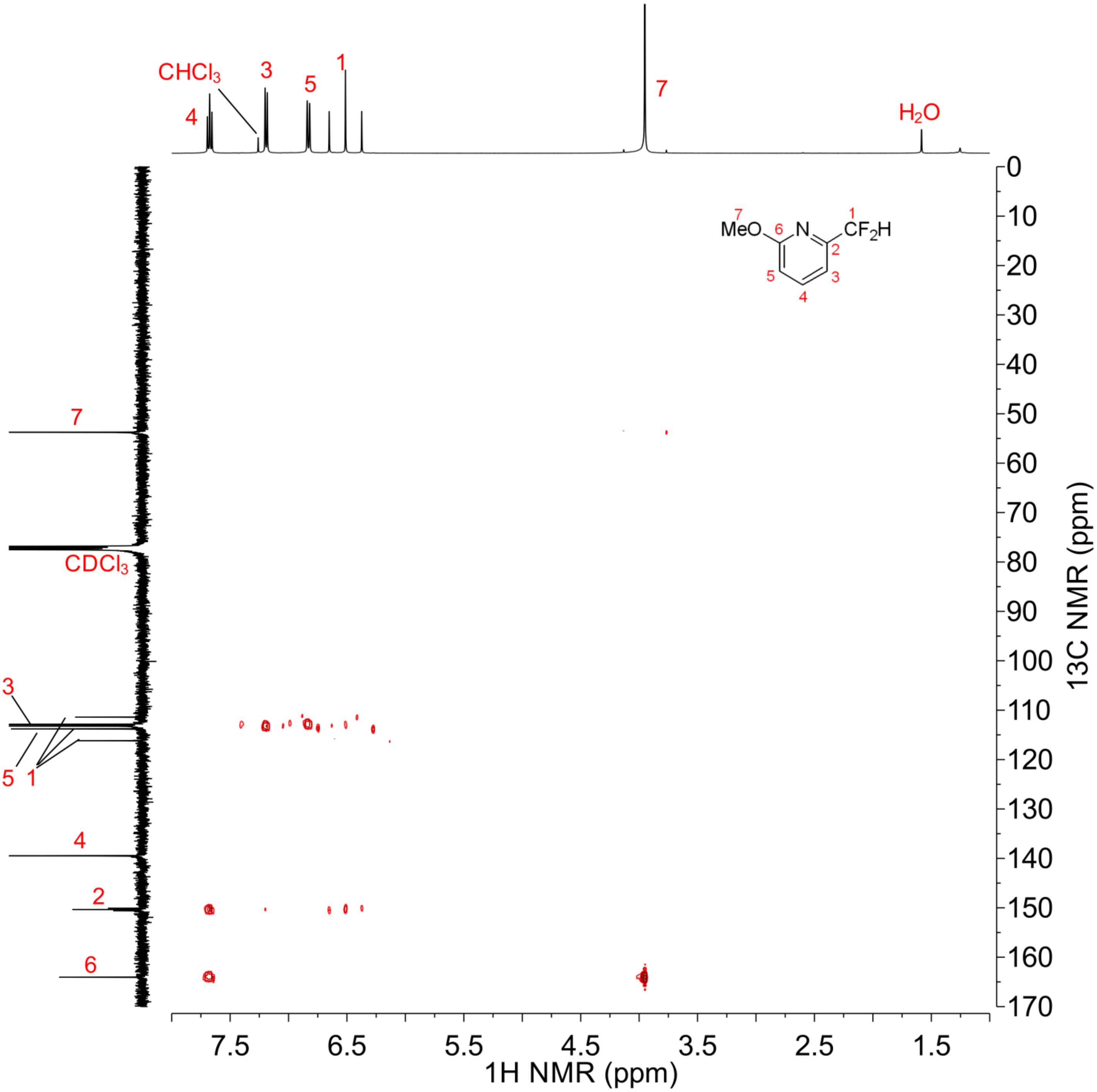

Figure S104. ^{1}H-^{13}C HMBC spectrum of 2-(difluoromethyl)-6-methoxy-pyridine (**7a**).

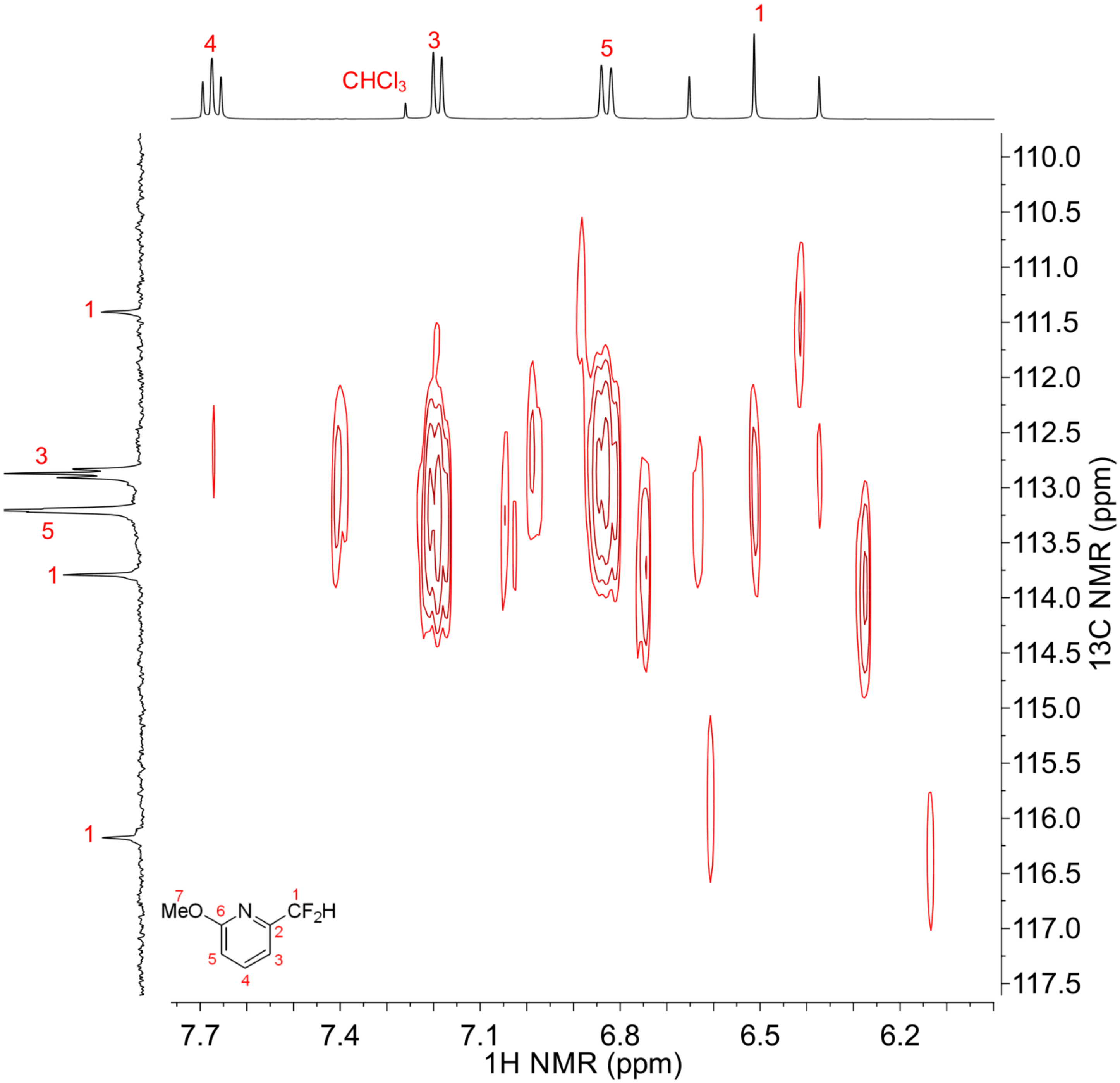

Figure S105. Expansion of ^{1}H-^{13}C HMBC spectrum of 2-(difluoromethyl)-6-methoxy-pyridine (**7a**) from 6.0 to 7.8 ppm (^{1}H) and 110 to 118 ppm (^{13}C).

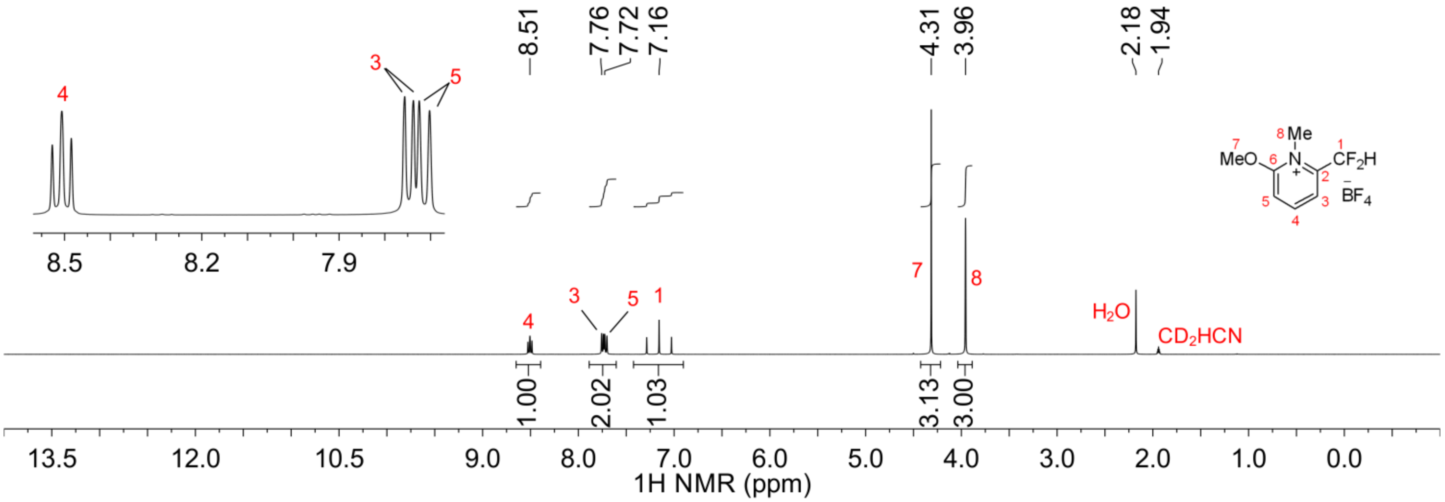

Figure S106. ^{1}H NMR spectrum of 2-(difluoromethyl)-6-methoxy-*N*-methylpyridinium tetrafluoroborate (**7b**).

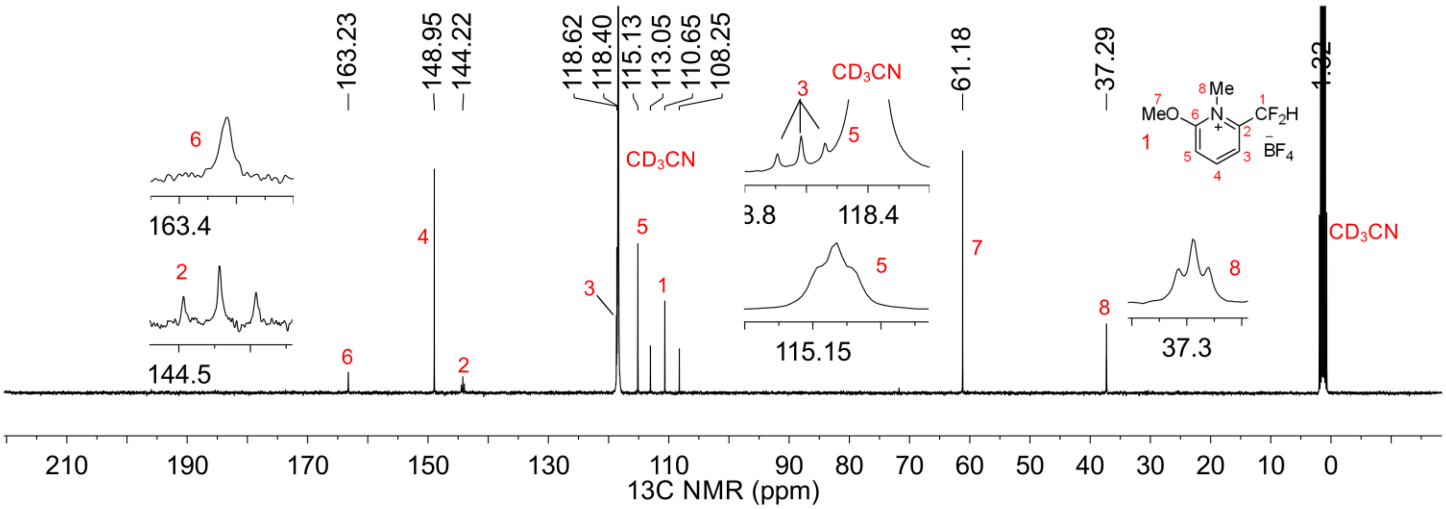

Figure S107. ^{13}C{^{1}H} NMR spectrum of 2-(difluoromethyl)-6-methoxy-*N*-methylpyridinium tetrafluoroborate (**7b**).

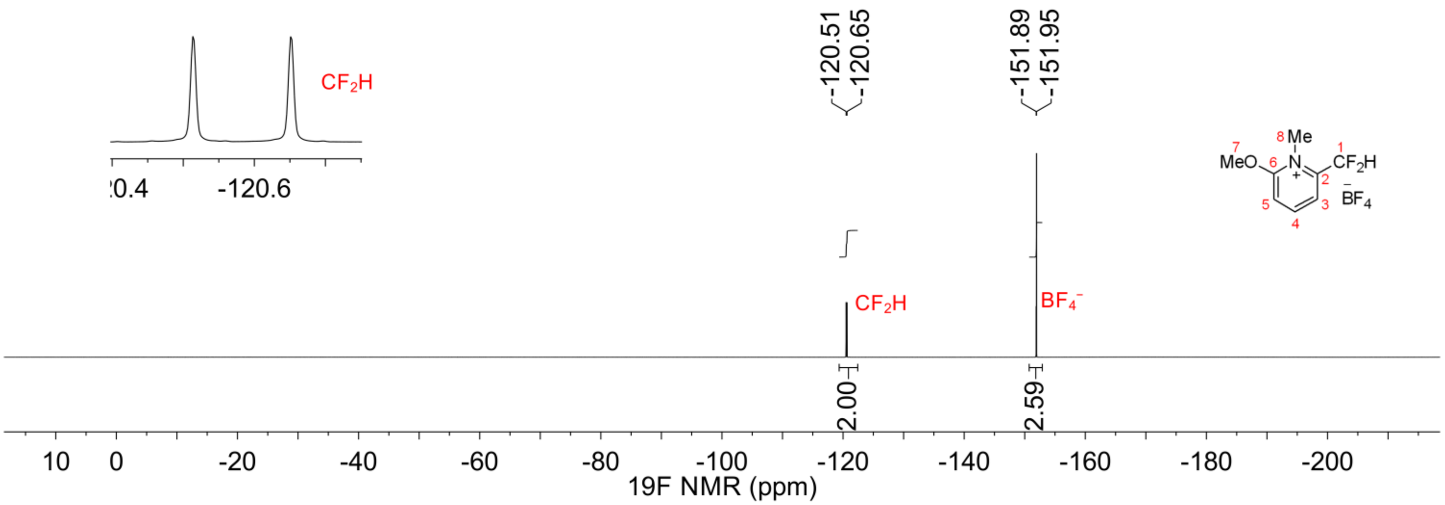

Figure S108. ^{19}F NMR spectrum of 2-(difluoromethyl)-6-methoxy-*N*-methylpyridinium tetrafluoroborate (**7b**).

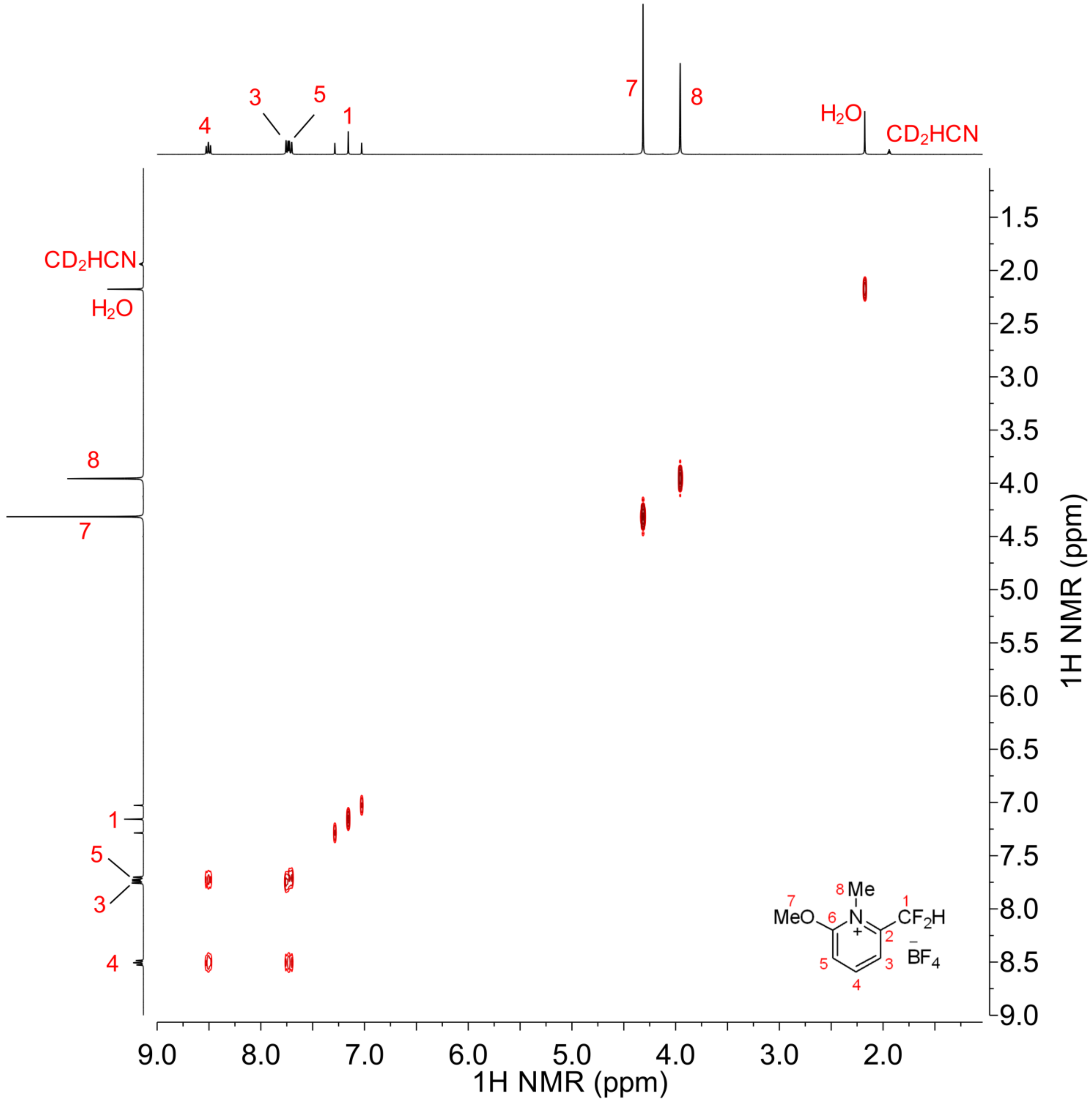

Figure S109. ^{1}H-^{1}H COSY spectrum of 2-(difluoromethyl)-6-methoxy-*N*-methylpyridinium tetrafluoroborate (**7b**).

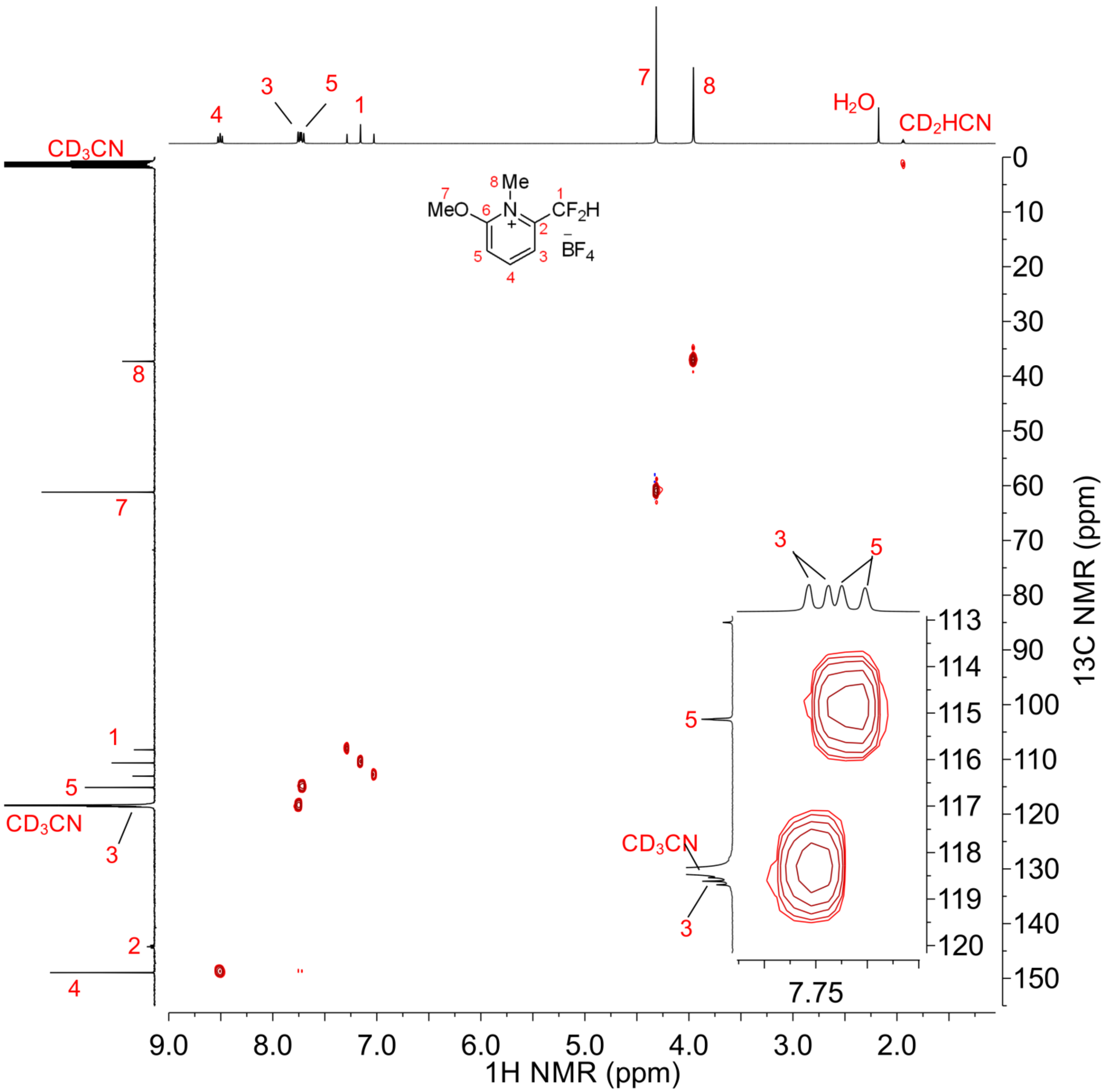

Figure S110. ^{1}H-^{13}C HSQC spectrum of 2-(difluoromethyl)-6-methoxy-*N*-methylpyridinium tetrafluoroborate (**7b**).

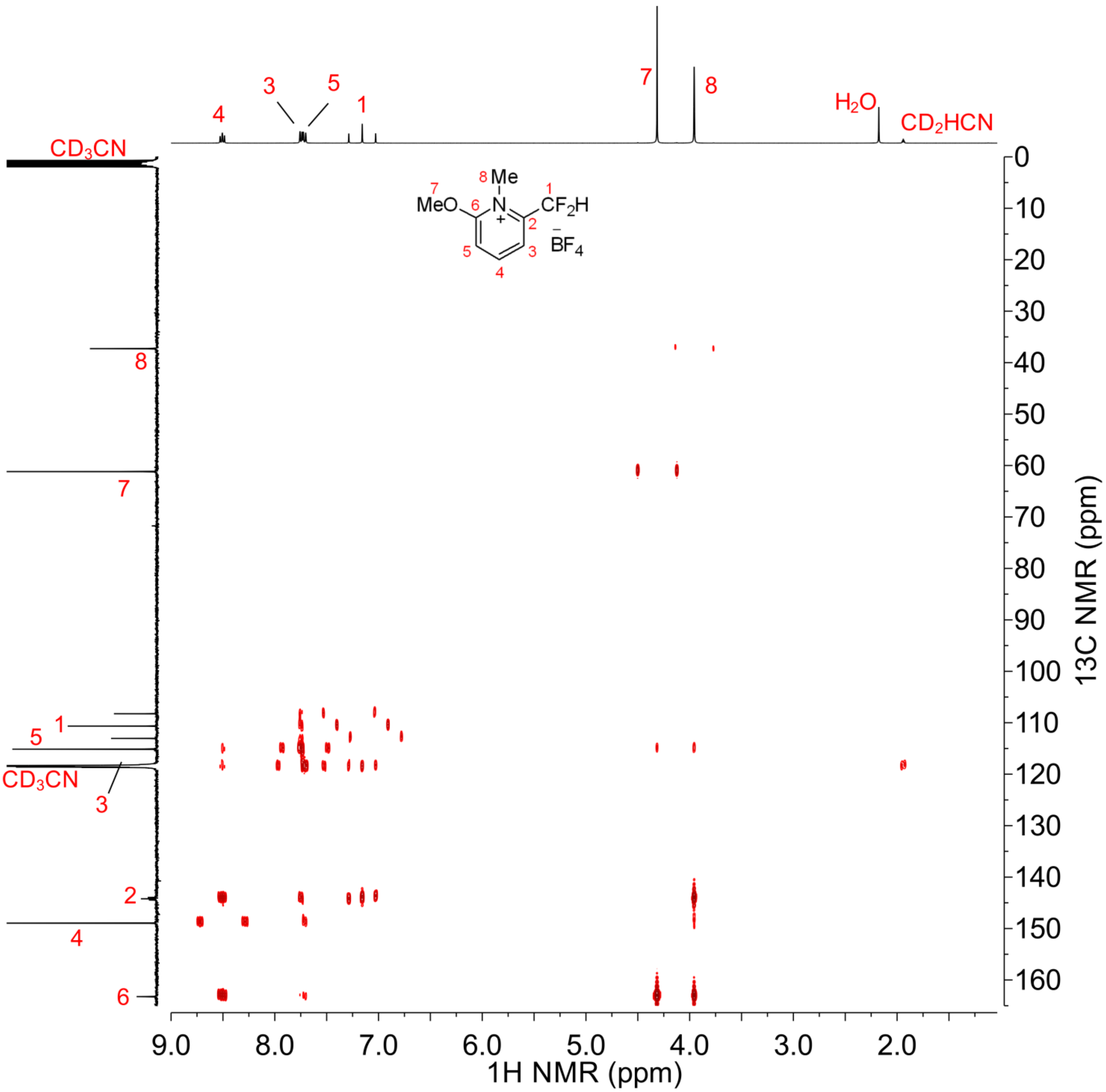

Figure S111. ^{1}H-^{13}C HMBC spectrum of 2-(difluoromethyl)-6-methoxy-*N*-methylpyridinium tetrafluoroborate (**7b**).

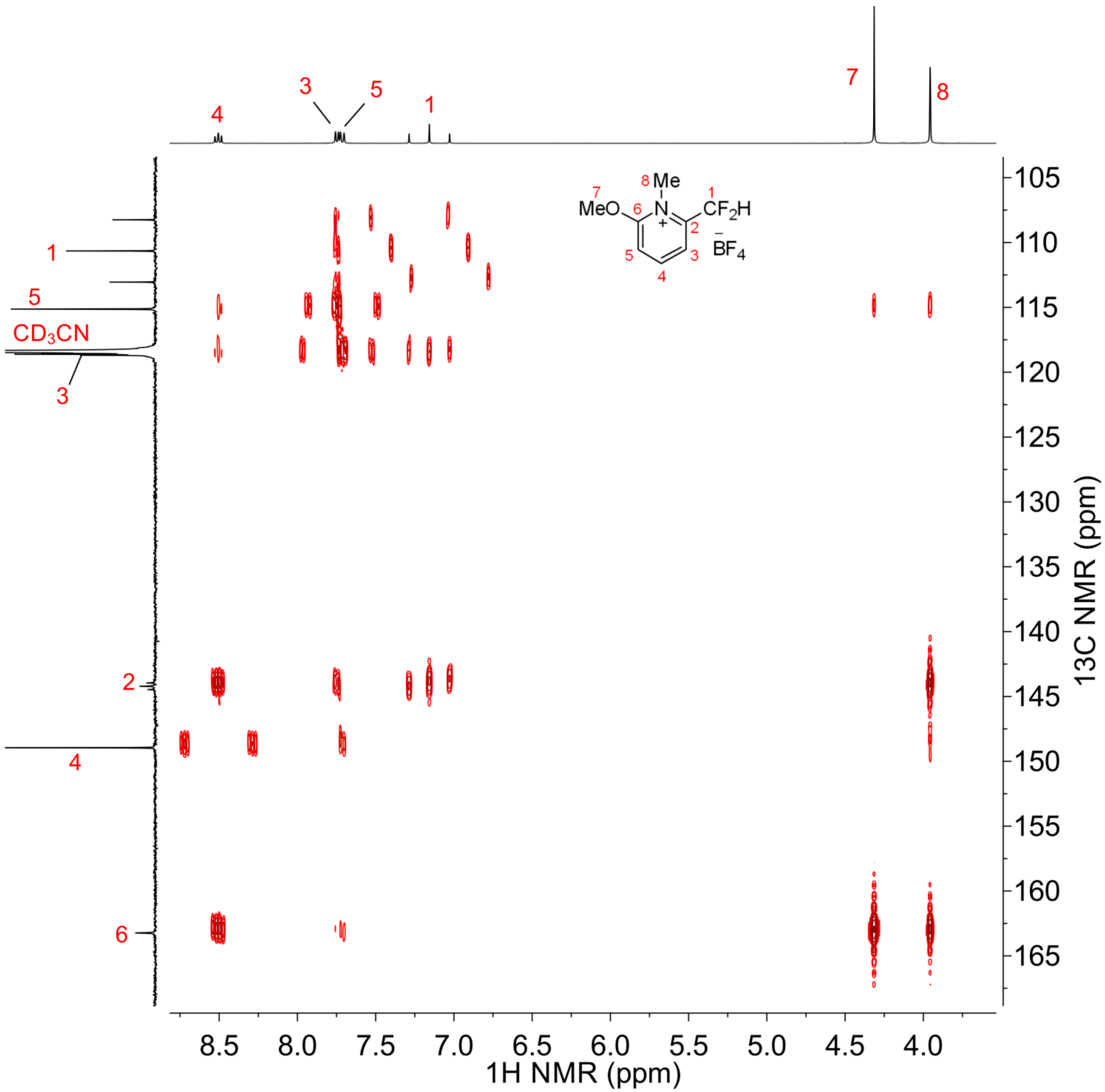

Figure S112. Expansion of ^{1}H-^{13}C HMBC spectrum of 2-(difluoromethyl)-6-methoxy-*N*-methylpyridinium tetrafluoroborate (**7b**) from 3.5 to 8.8 ppm (^{1}H) and 105 to 168 ppm (^{13}C).

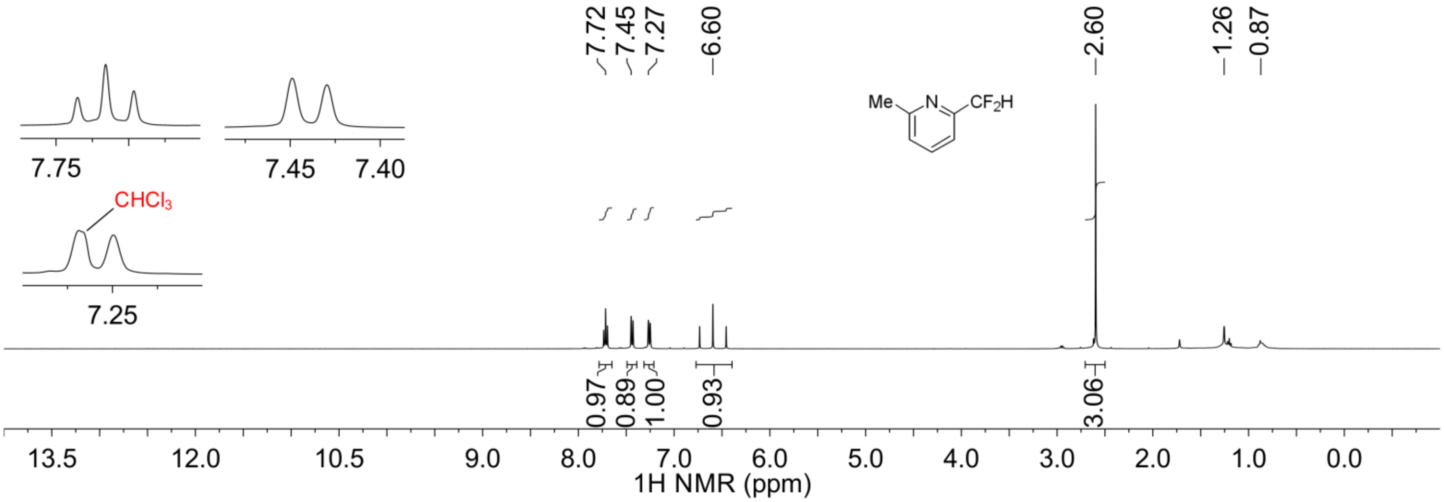

Figure S113. ^{1}H NMR spectrum of 2-(difluoromethyl)-6-methyl-pyridine (**8a**).

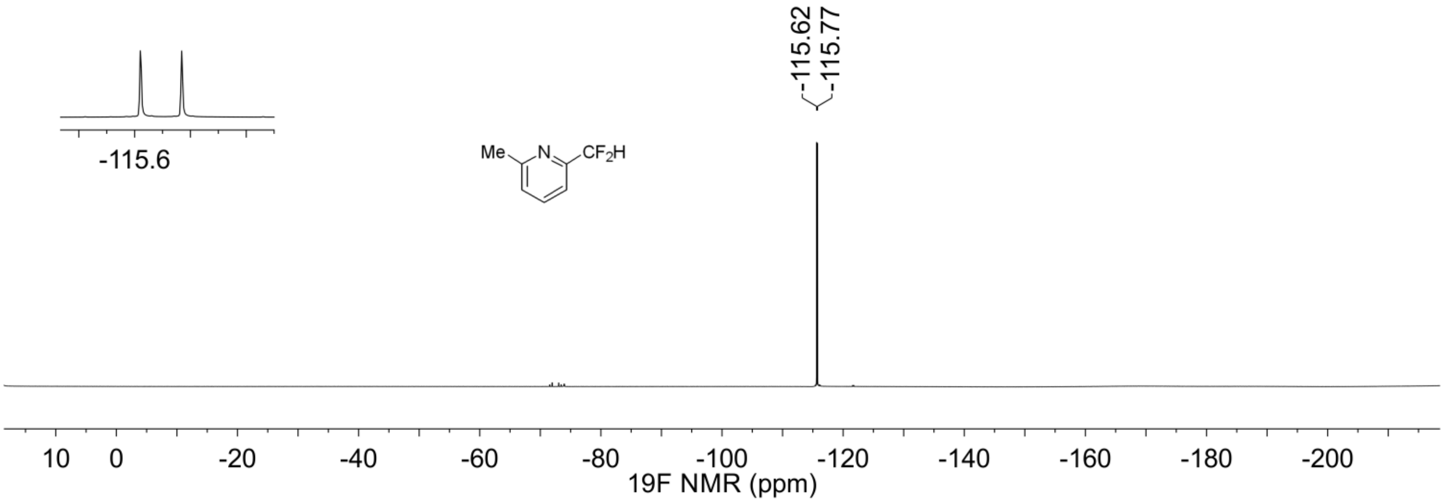

Figure S114. ^{19}F NMR spectrum of 2-(difluoromethyl)-6-methyl-pyridine (**8a**).

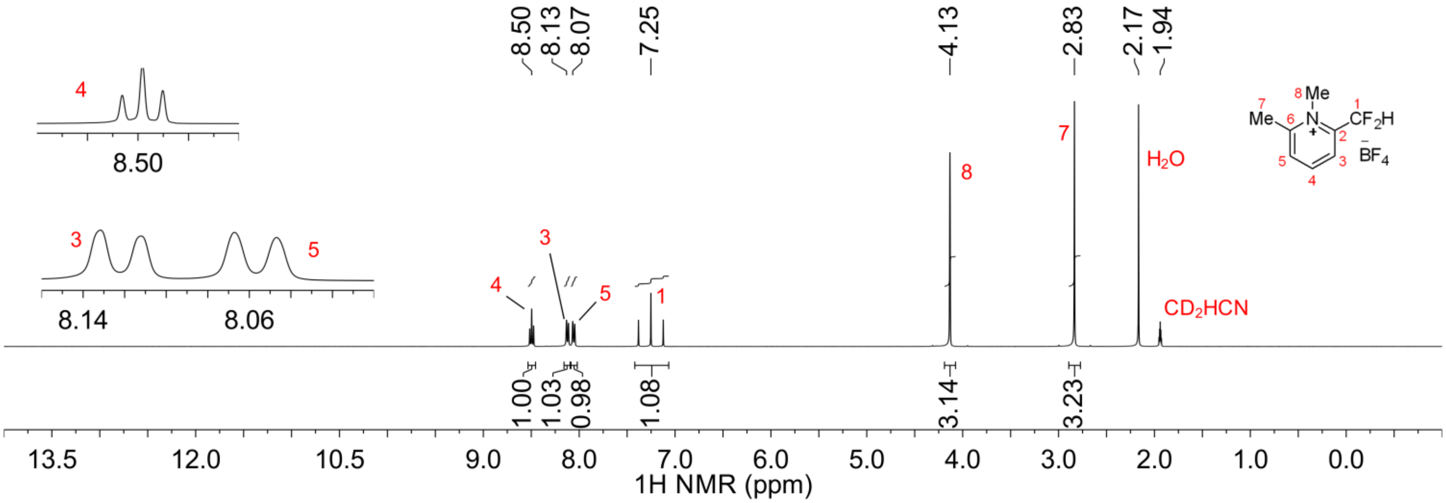

Figure S115. ^{1}H NMR spectrum of 2-(difluoromethyl)-6-methyl-*N*-methylpyridinium tetrafluoroborate (**8b**).

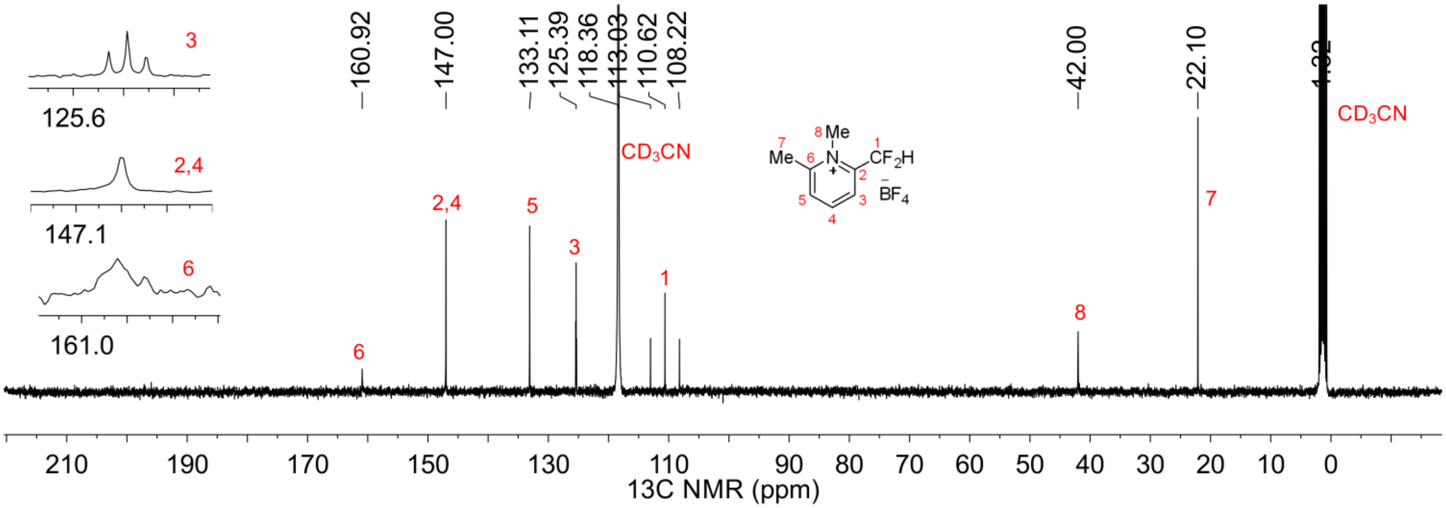

Figure S116. ^{13}C NMR spectrum of 2-(difluoromethyl)-6-methyl-*N*-methylpyridinium tetrafluoroborate (**8b**).

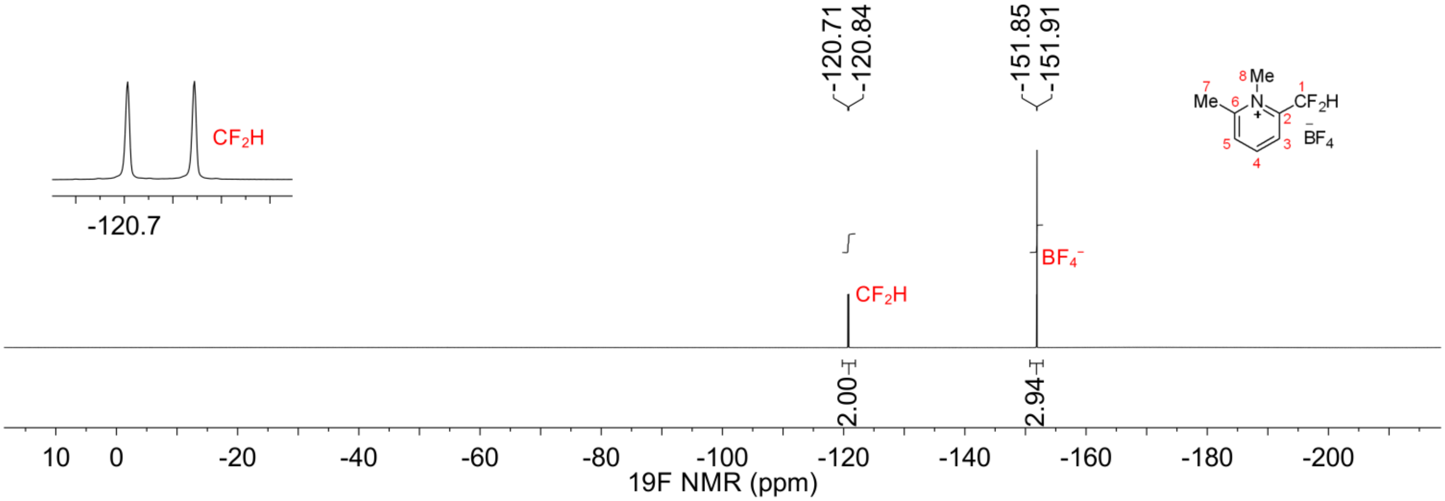

Figure S117. ^{19}F NMR spectrum of 2-(difluoromethyl)-6-methyl-*N*-methylpyridinium tetrafluoroborate (**8b**).

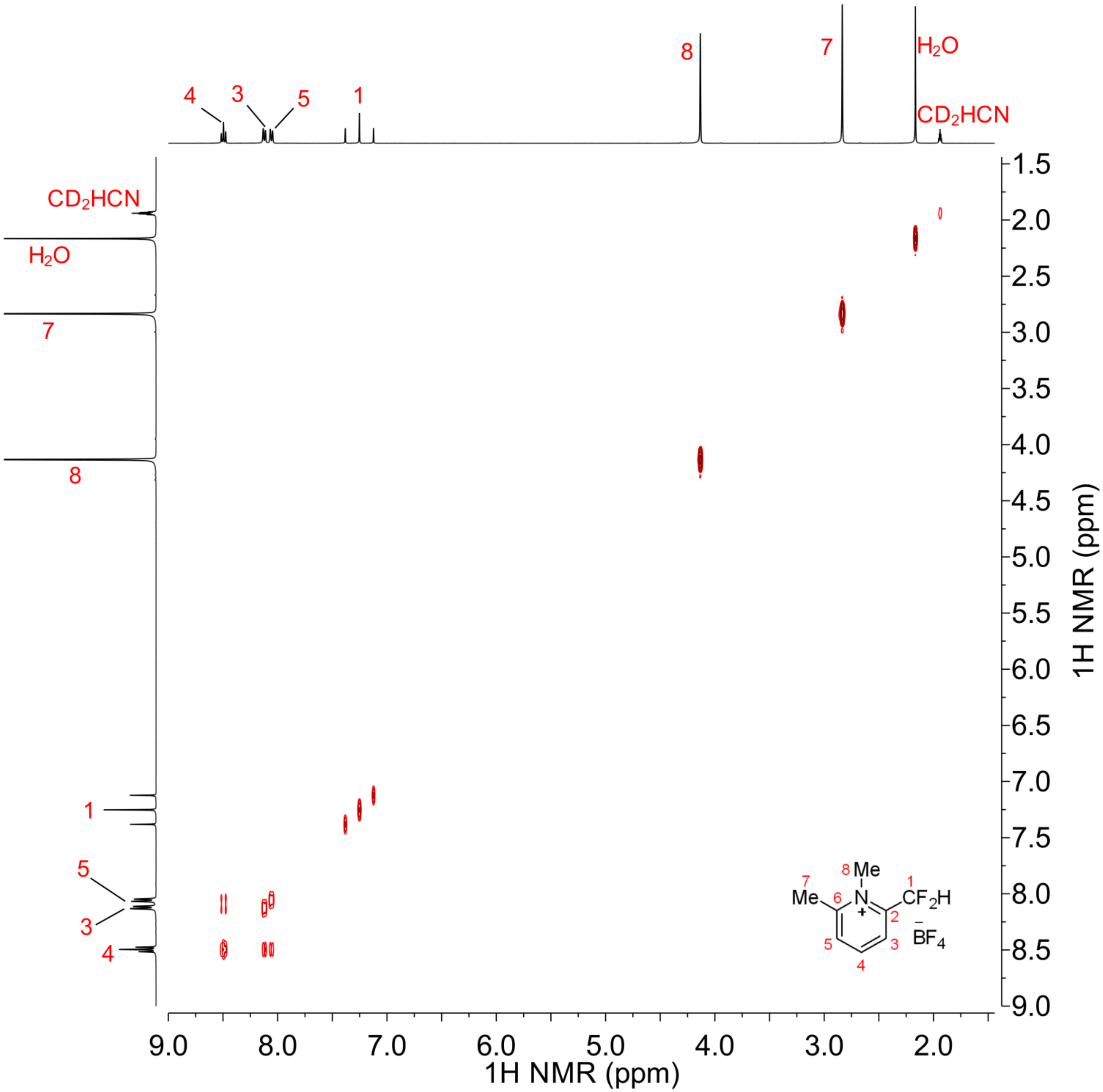

Figure S118. ^{1}H-^{1}H COSY spectrum of 2-(difluoromethyl)-6-methyl-*N*-methylpyridinium tetrafluoroborate (**8b**).

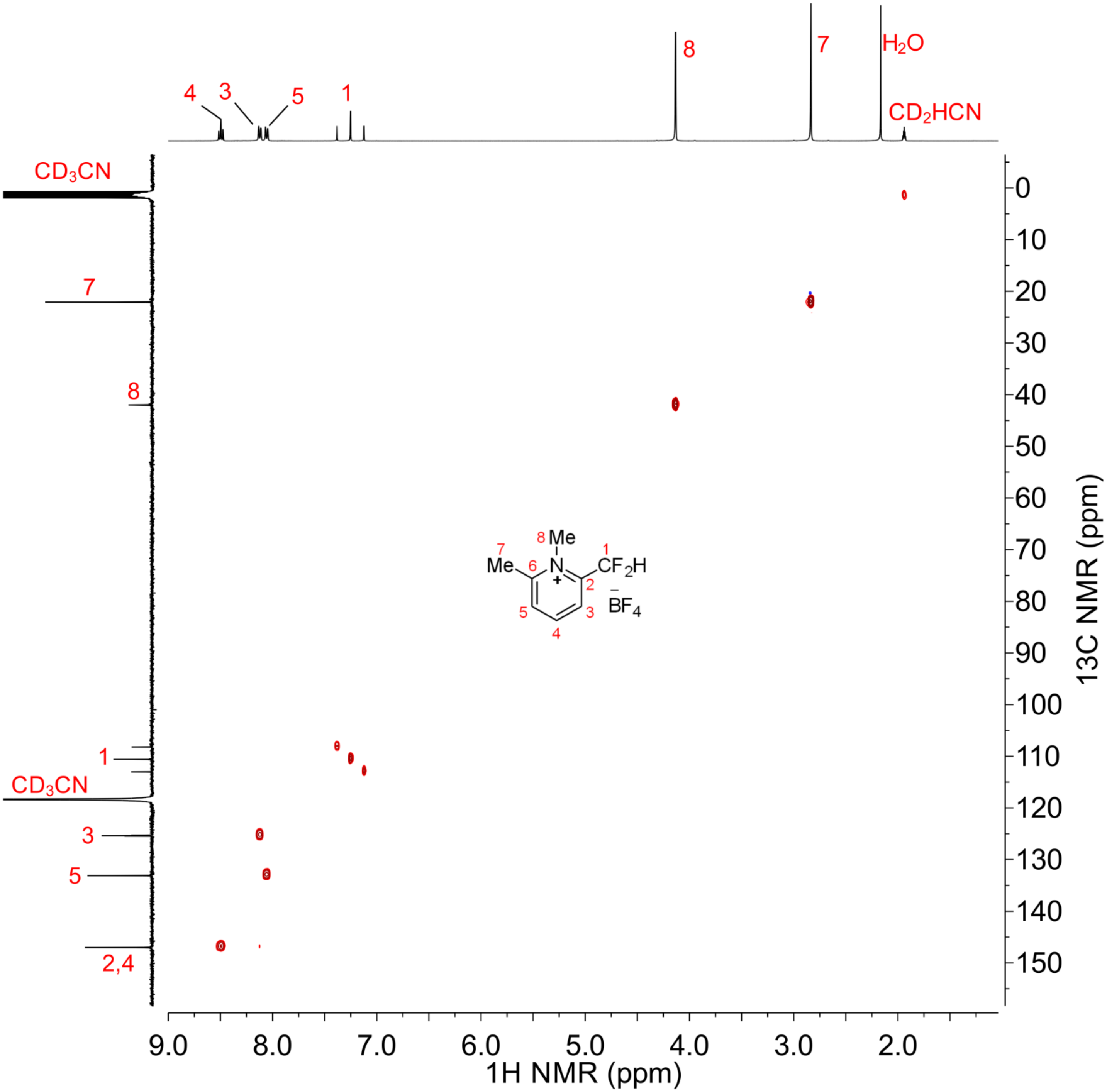

Figure S119. ^{1}H-^{13}C HSQC spectrum of 2-(difluoromethyl)-6-methyl-*N*-methylpyridinium tetrafluoroborate (**8b**).

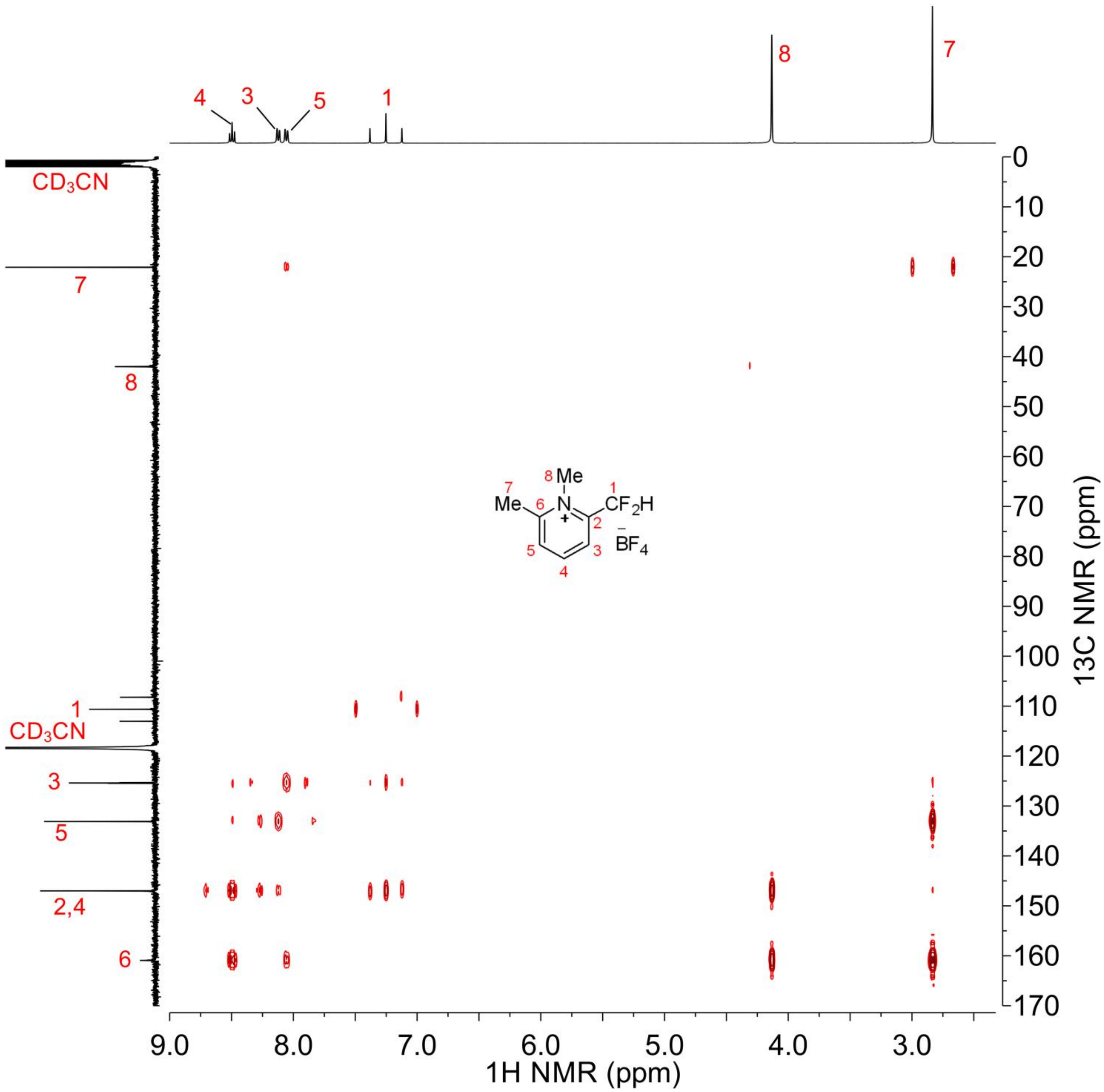

Figure S120. ^{1}H-^{13}C HMBC spectrum of 2-(difluoromethyl)-6-methyl-*N*-methylpyridinium tetrafluoroborate (**8b**).

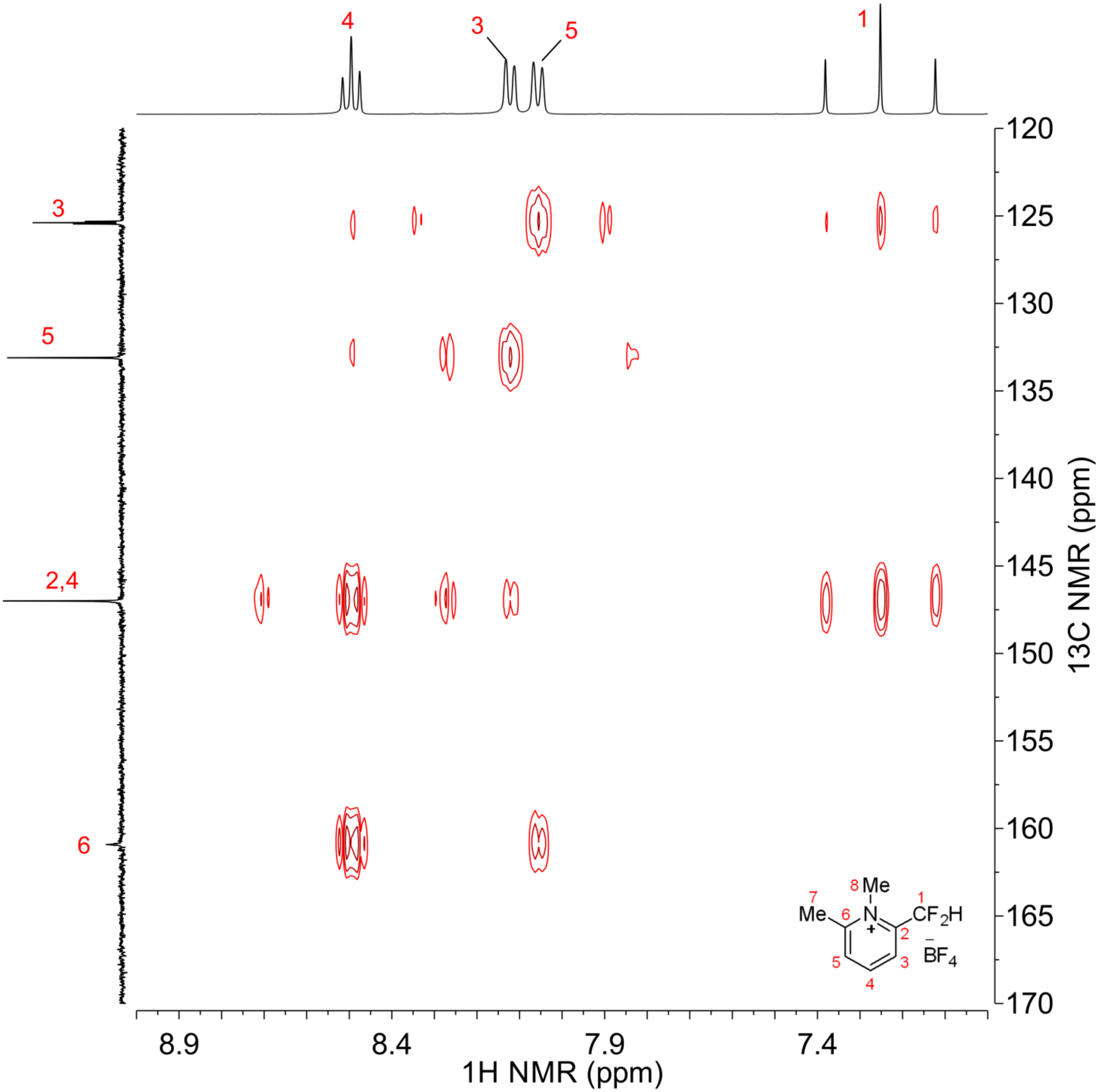

Figure S121. Expansion of ^{1}H-^{13}C HMBC spectrum of 2-(difluoromethyl)-6-methyl-*N*-methylpyridinium tetrafluoroborate (**8b**) from 7.0 to 9.0 ppm (^{1}H) and 120 to 170 ppm (^{13}C).

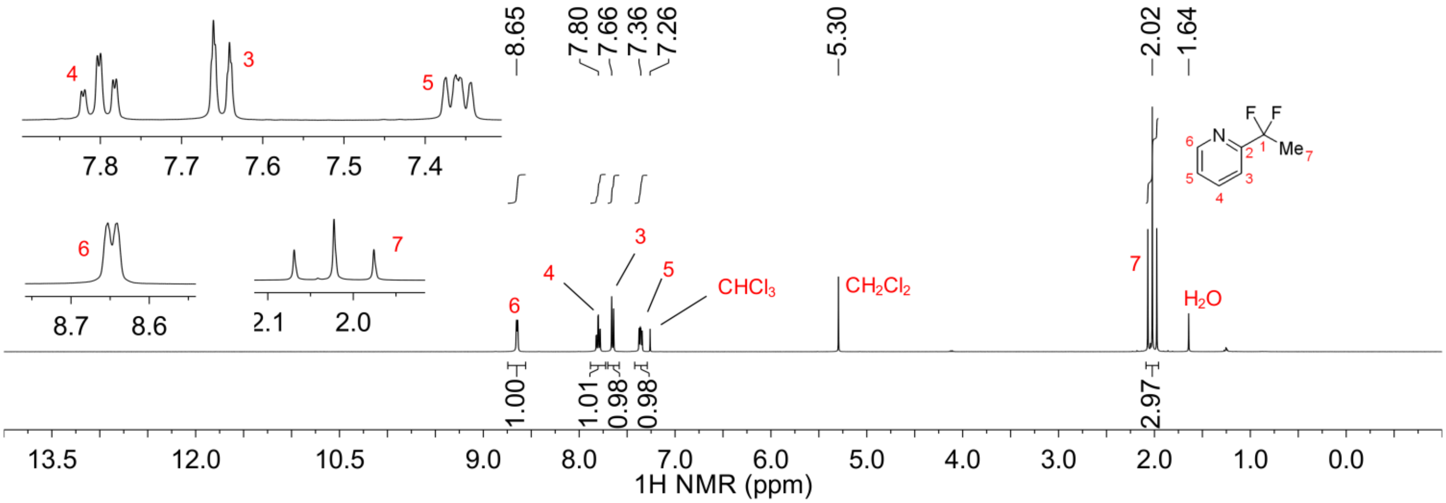

Figure S122. ^{1}H NMR spectrum of 2-(1,1-difluoroethyl)-pyridine (**9a**).

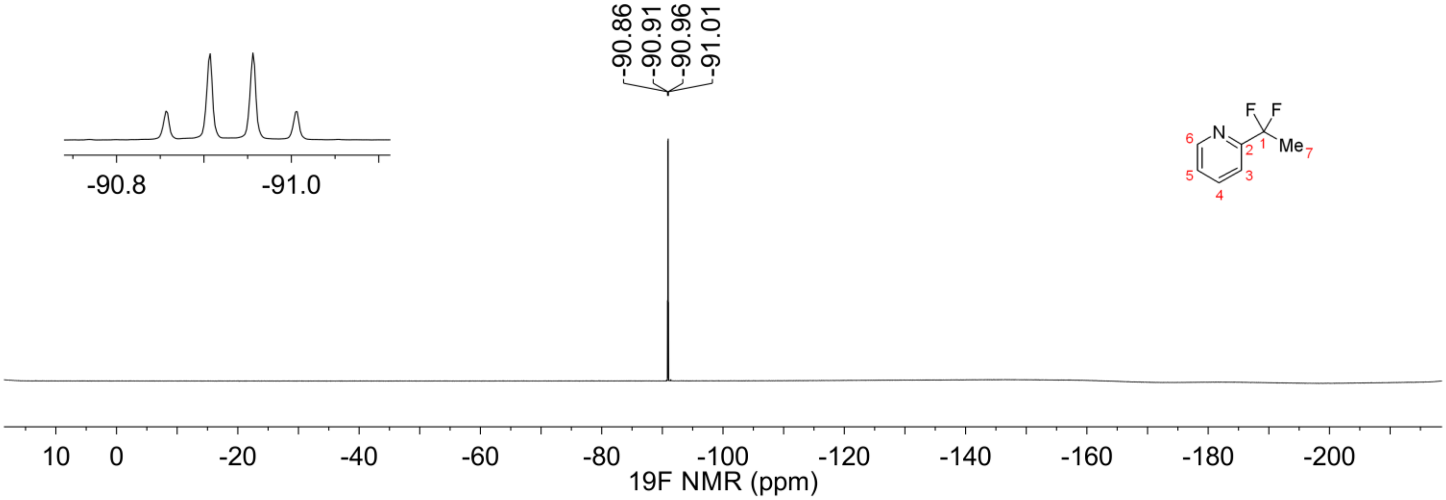

Figure S123. ^{19}F NMR spectrum of 2-(1,1-difluoroethyl)-pyridine (**9a**).

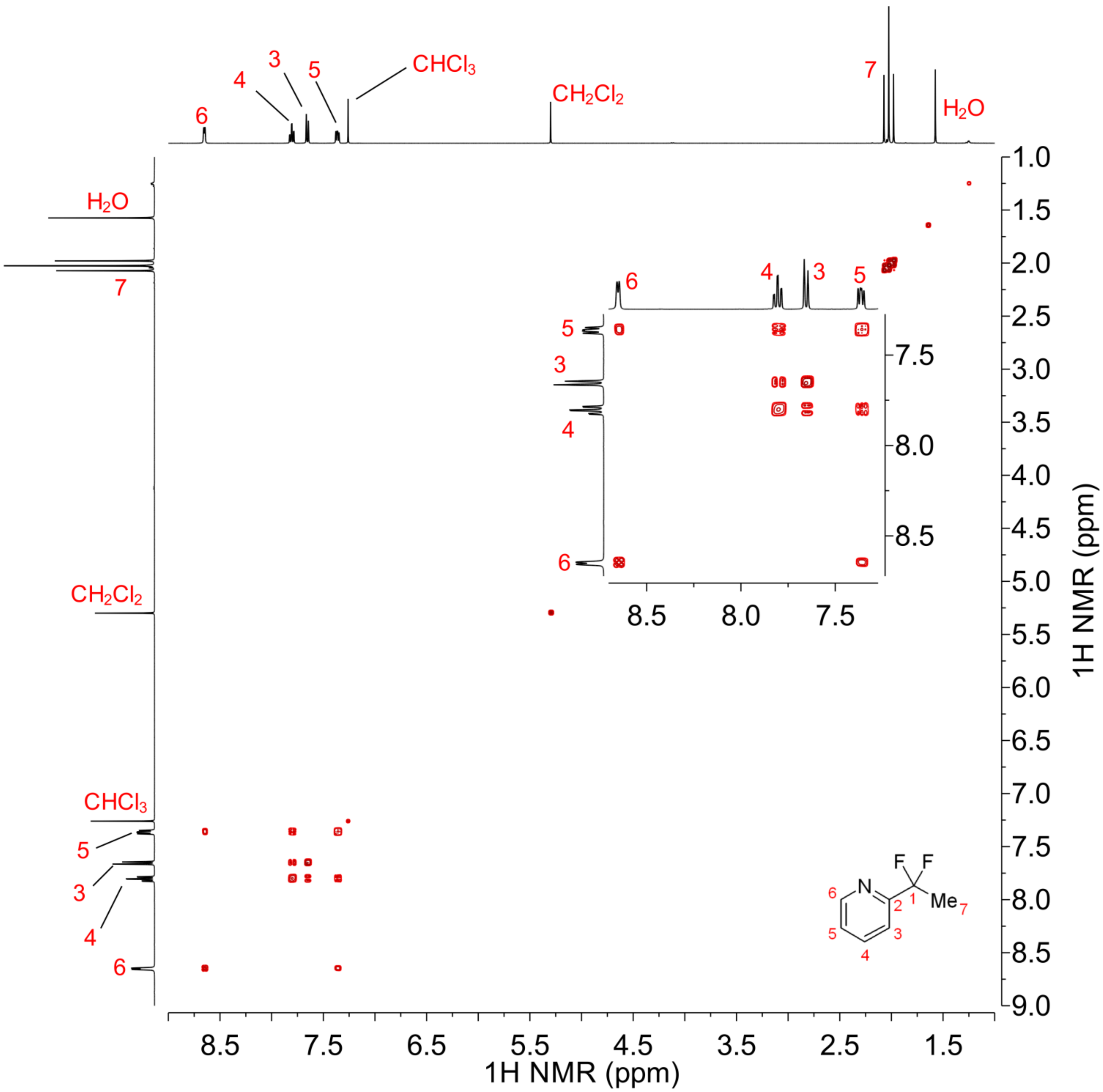

Figure S124. ^{1}H-^{1}H COSY spectrum of 2-(1,1-difluoroethyl)-pyridine (**9a**).

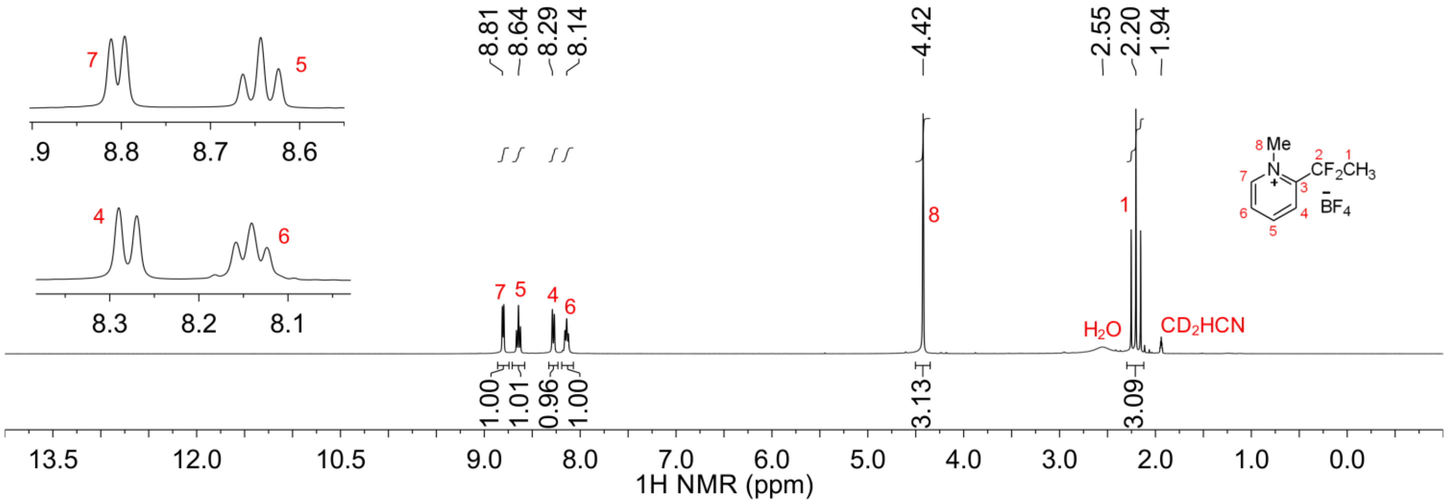

Figure S125. ^{1}H NMR spectrum of 2-(1,1-difluoroethyl)-*N*-methylpyridinium tetrafluoroborate (**9b**).

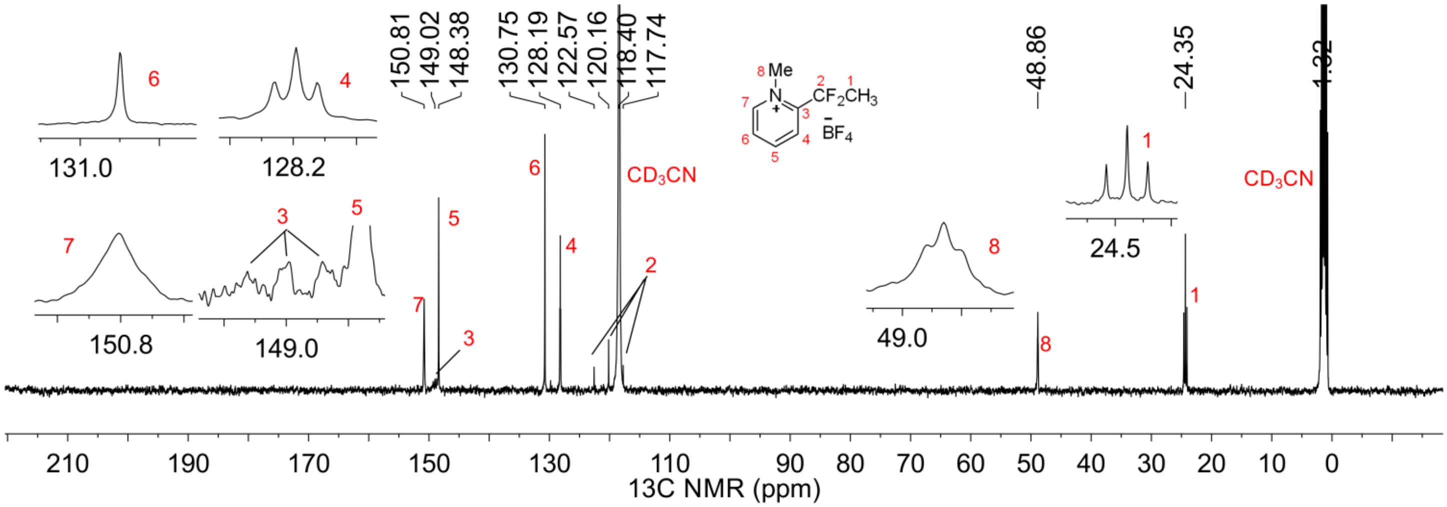

Figure S126. ^{13}C{^{1}H} NMR spectrum of 2-(1,1-difluoroethyl)-*N*-methylpyridinium tetrafluoroborate (**9b**).

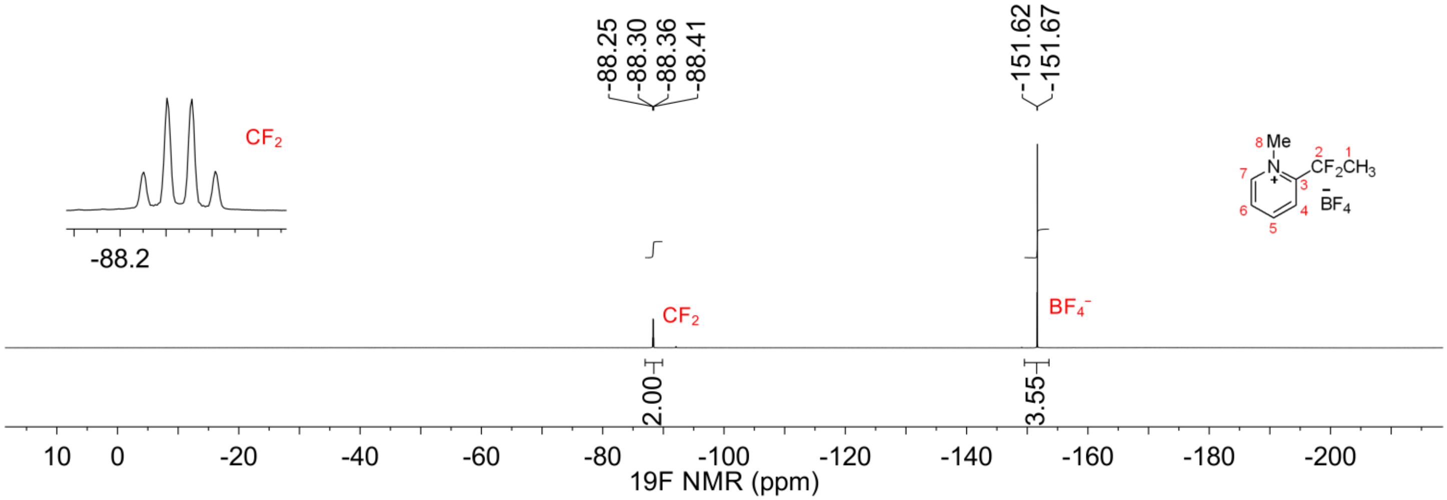

Figure S127. ^{19}F NMR spectrum of 2-(1,1-difluoroethyl)-*N*-methylpyridinium tetrafluoroborate (**9b**).

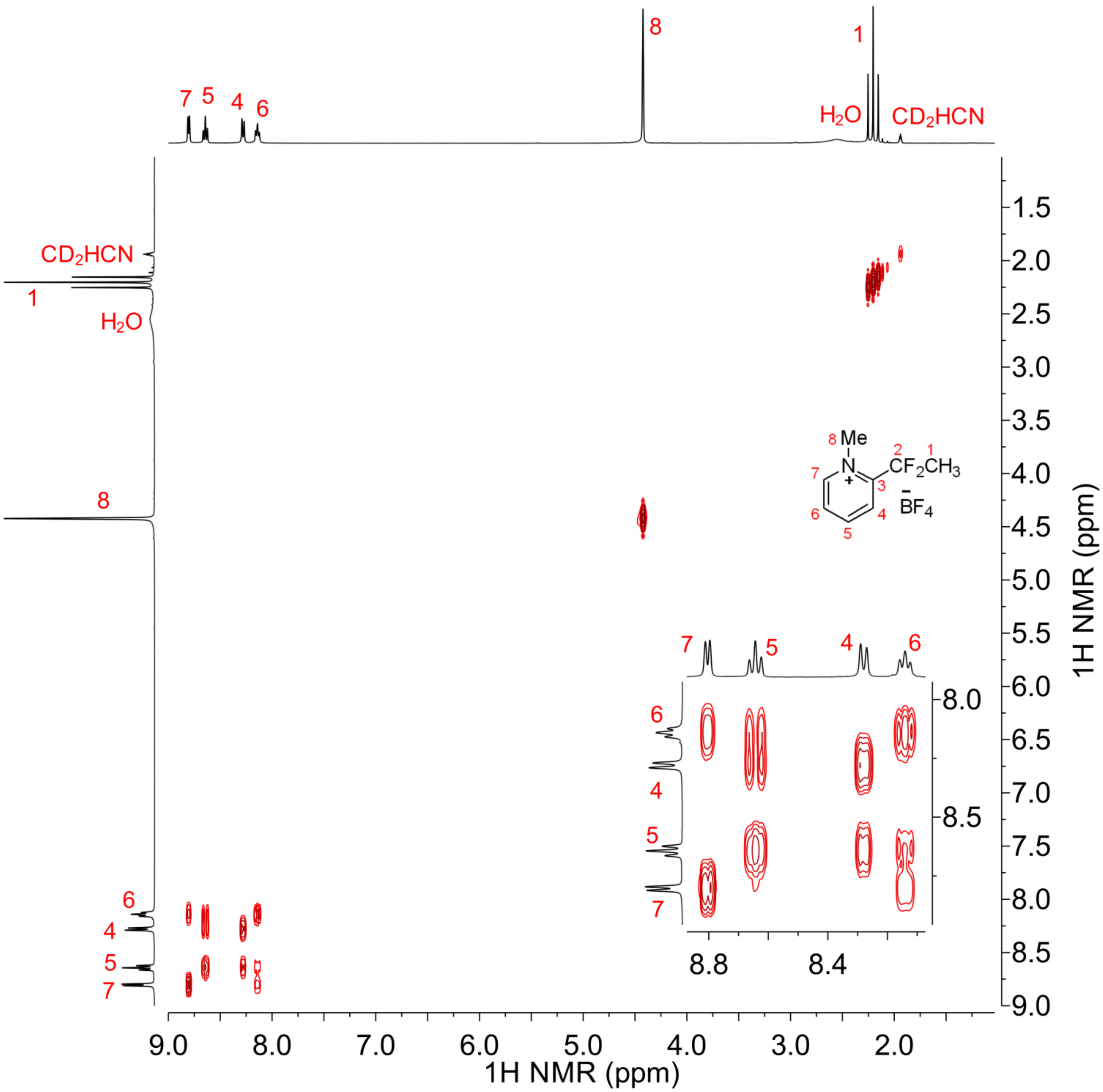

Figure S128. ^{1}H-^{1}H COSY spectrum of 2-(1,1-difluoroethyl)-*N*-methylpyridinium tetrafluoroborate (**9b**).

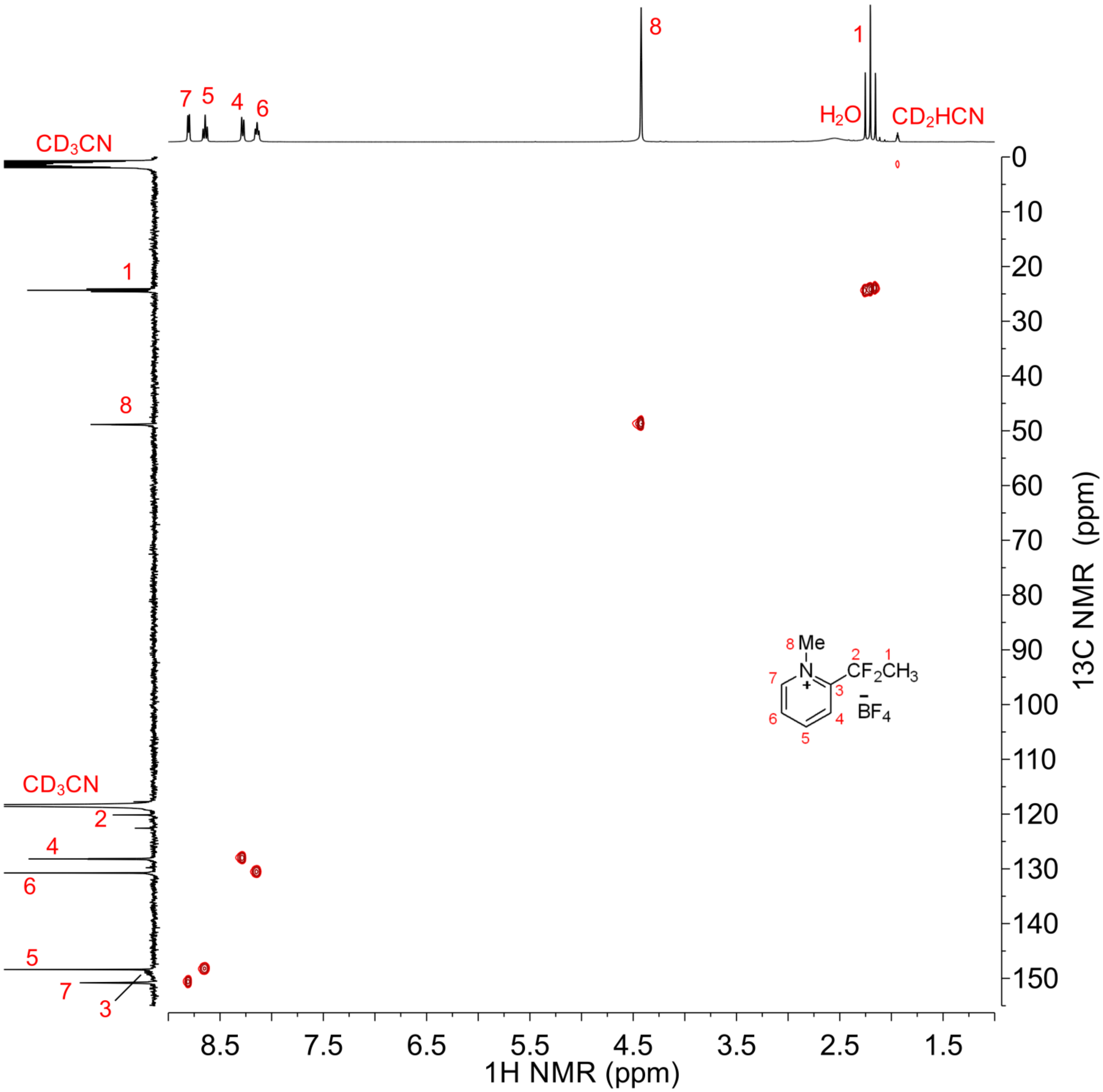

Figure S129. ^{1}H-^{13}C HSQC spectrum of 2-(1,1-difluoroethyl)-*N*-methylpyridinium tetrafluoroborate (**9b**).

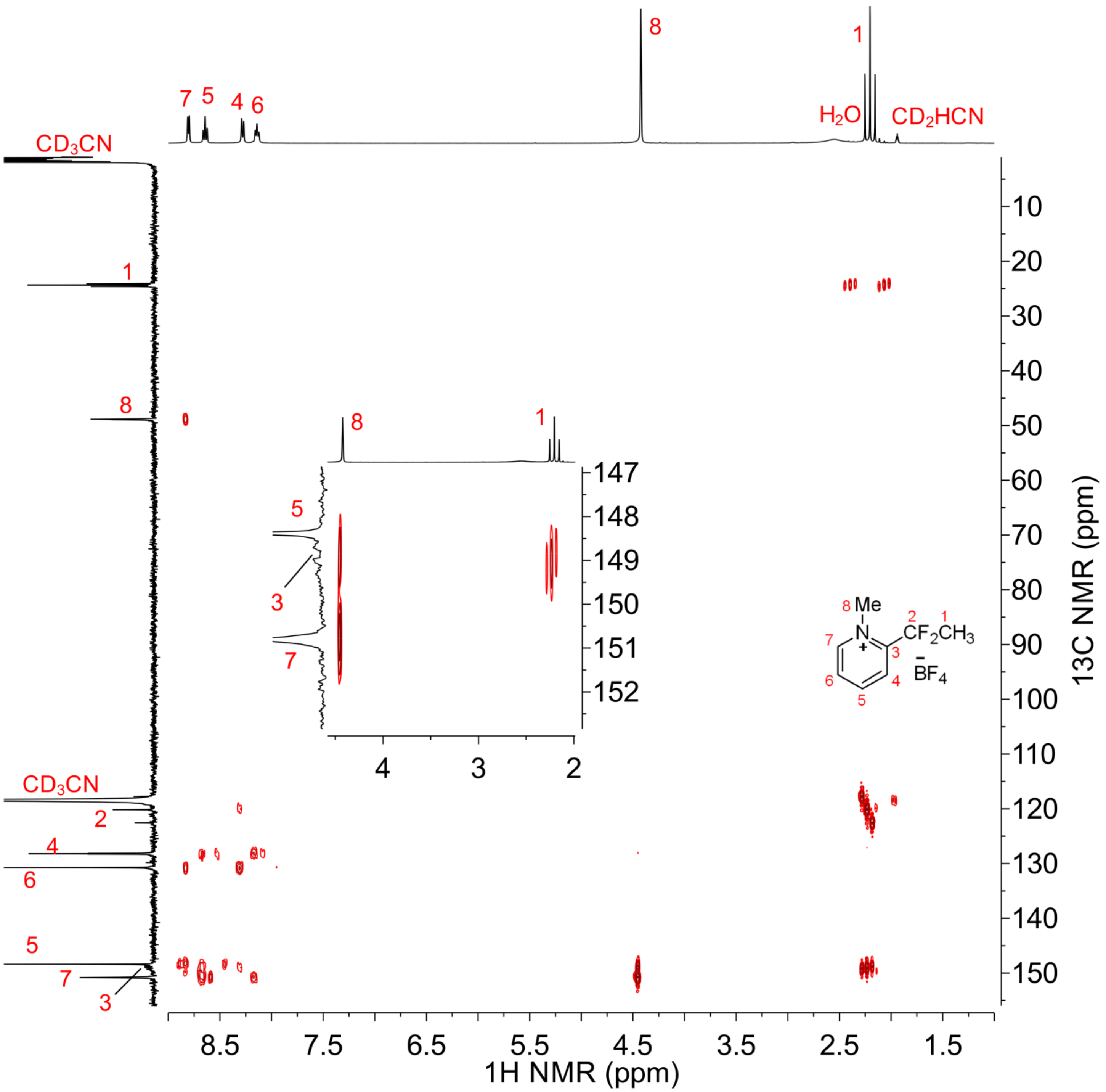

Figure S130. ^{1}H-^{13}C HMBC spectrum of 2-(1,1-difluoroethyl)-*N*-methylpyridinium tetrafluoroborate (**9b**).

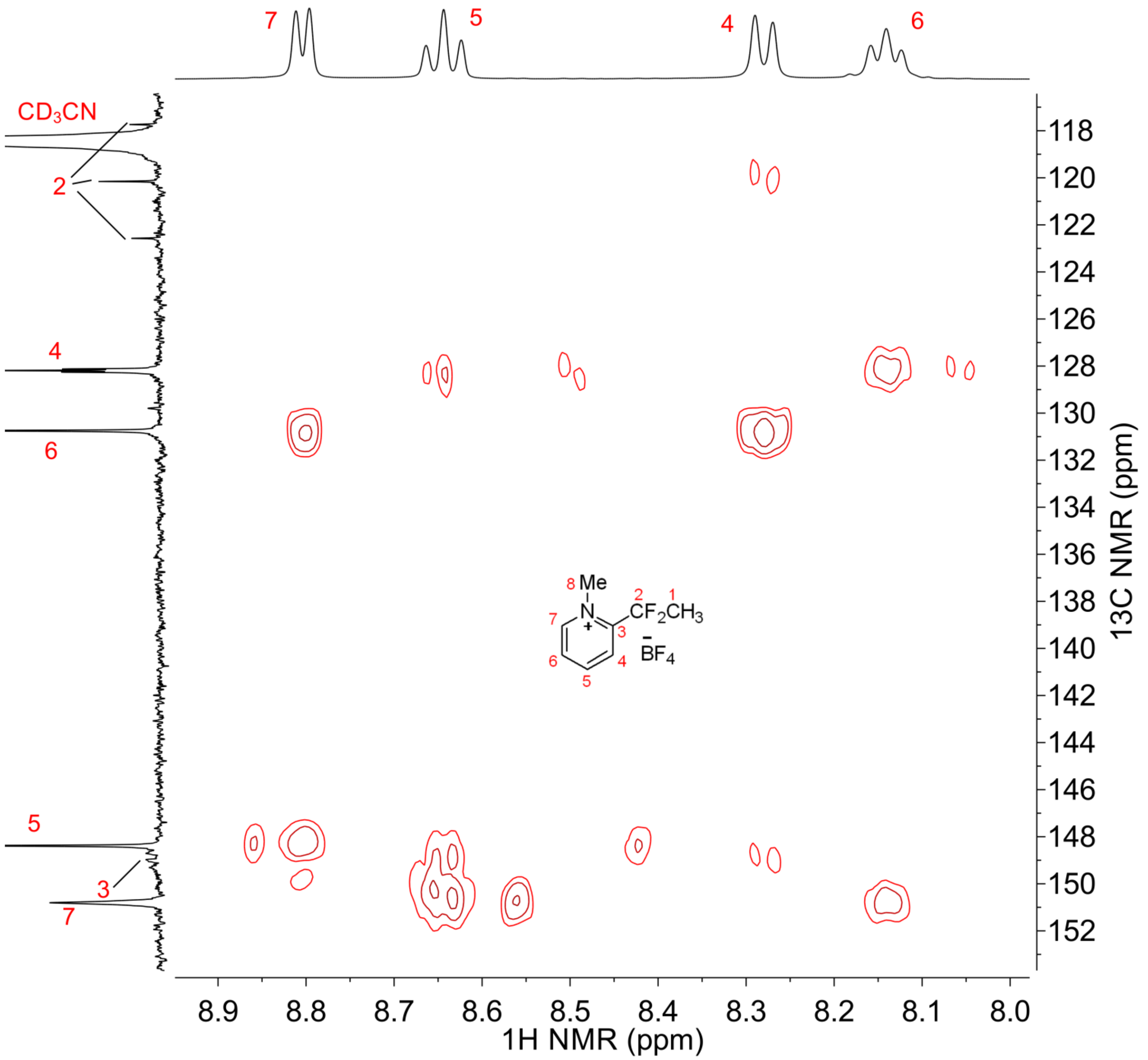

Figure S131. Expansion of ^{1}H-^{13}C HMBC spectrum of 2-(1,1-difluoroethyl)-*N*-methylpyridinium tetrafluoroborate (**9b**) from 8.0 to 9.0 ppm (^{1}H) and 116 to 154 ppm (^{13}C).

References

1. L. Huang, W. Liu, L.-L. Zhao, Z. Zhang, X. Yan, *J. Org. Chem.* **2021**, *86*, 3981.
2. A. Adachi, T. Hashimoto, K. Aikawa, K. Nozaki, T. Okazoe, *Org. Chem. Front.* **2023**, *10*, 5362.
3. A. Haas, M. Spitzer, M. Lieb, *Chem. Ber.* **1988**, *121*, 1329.
4. S. J. Pike, E. Lavagnini, L. M. Varley, J. L. Cook, C. A. Hunter, *Chem. Sci.* **2019**, *10*, 5943.
5. Y. Zhao, D. G. Truhlar, *Theor. Chem. Acc.* **2008**, *120*, 215.
6. M. J. Frisch, G. W. Trucks, H. B. Schlegel, G. E. Scuseria, M. A. Robb, J. R. Cheeseman, G. Scalmani, V. Barone, G. A. Petersson, H. Nakatsuji, X. Li, M. Caricato, A. V. Marenich, J. Bloino, B. G. Janesko, R. Gomperts, B. Mennucci, H. P. Hratchian, J. V. Ortiz, A. F. Izmaylov, J. L. Sonnenberg, Williams, F. Ding, F. Lipparini, F. Egidi, J. Goings, B. Peng, A. Petrone, T. Henderson, D. Ranasinghe, V. G. Zakrzewski, J. Gao, N. Rega, G. Zheng, W. Liang, M. Hada, M. Ehara, K. Toyota, R. Fukuda, J. Hasegawa, M. Ishida, T. Nakajima, Y. Honda, O. Kitao, H. Nakai, T. Vreven, K. Throssell, J. A. Montgomery Jr., J. E. Peralta, F. Ogliaro, M. J. Bearpark, J. J. Heyd, E. N. Brothers, K. N. Kudin, V. N. Staroverov, T. A. Keith, R. Kobayashi, J. Normand, K. Raghavachari, A. P. Rendell, J. C. Burant, S. S. Iyengar, J. Tomasi, M. Cossi, J. M. Millam, M. Klene, C. Adamo, R. Cammi, J. W. Ochterski, R. L. Martin, K. Morokuma, O. Farkas, J. B. Foresman, D. J. Fox, Wallingford, CT, **2016**.
7. M. Cossi, N. Rega, G. Scalmani, V. Barone, *J. Comput. Chem.* **2003**, *24*, 669.
8. V. Barone, M. Cossi, *J. Phys. Chem. A* **1998**, *102*, 1995.
9. A. E. Reed, L. A. Curtiss, F. Weinhold, *Chem. Rev.* **1988**, *88*, 899.
